# Synthesis and Biological Evaluation of 1,3-Dideazapurine-Like 7-Amino-5-Hydroxymethyl-Benzimidazole Ribonucleoside Analogues as Aminoacyl-tRNA Synthetase Inhibitors

**DOI:** 10.3390/molecules25204751

**Published:** 2020-10-16

**Authors:** Baole Zhang, Luping Pang, Manesh Nautiyal, Steff De Graef, Bharat Gadakh, Eveline Lescrinier, Jef Rozenski, Sergei V. Strelkov, Stephen D. Weeks, Arthur Van Aerschot

**Affiliations:** 1 Rega Institute for Medical Research, Medicinal Chemistry, KU Leuven, Herestraat 49–box 1041, 3000 Leuven, Belgium; zhangbaole.2008@163.com (B.Z.); Luping.Pang@kuleuven.be (L.P.); manesh2006@gmail.com (M.N.); bkgadakh@gmail.com (B.G.); Eveline.Lescrinier@kuleuven.be (E.L.); Jef.Rozenski@kuleuven.be (J.R.); 2 Laboratory for Biocrystallography, Department of Pharmaceutical and Pharmacological Sciences, KU Leuven, Herestraat 49–box 822, 3000 Leuven, Belgium; Steff.Degraef@kuleuven.be (S.D.G.); Sergei.Strelkov@kuleuven.be (S.V.S.); sweeks@orthogontherapeutics.com (S.D.W.)

**Keywords:** aminoacyl sulfamoylated nucleosides, aaRS inhibition, structure-activity relationship, sugar-base condensation, heterocycle glycosylation

## Abstract

Aminoacyl-tRNA synthetases (aaRSs) have become viable targets for the development of antimicrobial agents due to their crucial role in protein translation. A series of six amino acids were coupled to the purine-like 7-amino-5-hydroxymethylbenzimidazole nucleoside analogue following an optimized synthetic pathway. These compounds were designed as aaRS inhibitors and can be considered as 1,3-dideazaadenine analogues carrying a 2-hydroxymethyl substituent. Despite our intentions to obtain *N*^1^-glycosylated 4-aminobenzimidazole congeners, resembling the natural purine nucleosides glycosylated at the *N*^9^-position, we obtained the *N*^3^-glycosylated benzimidazole derivatives as the major products, resembling the respective purine *N*^7^-glycosylated nucleosides. A series of X-ray crystal structures of class I and II aaRSs in complex with newly synthesized compounds revealed interesting interactions of these “base-flipped” analogues with their targets. While the exocyclic amine of the flipped base mimics the reciprocal interaction of the *N*^3^-purine atom of aminoacyl-sulfamoyl adenosine (aaSA) congeners, the hydroxymethyl substituent of the flipped base apparently loses part of the standard interactions of the adenine *N*^1^ and the *N*^6^-amine as seen with aaSA analogues. Upon the evaluation of the inhibitory potency of the newly obtained analogues, nanomolar inhibitory activities were noted for the leucine and isoleucine analogues targeting class I aaRS enzymes, while rather weak inhibitory activity against the corresponding class II aaRSs was observed. This class bias could be further explained by detailed structural analysis.

## 1. Introduction

**Figure 1:**
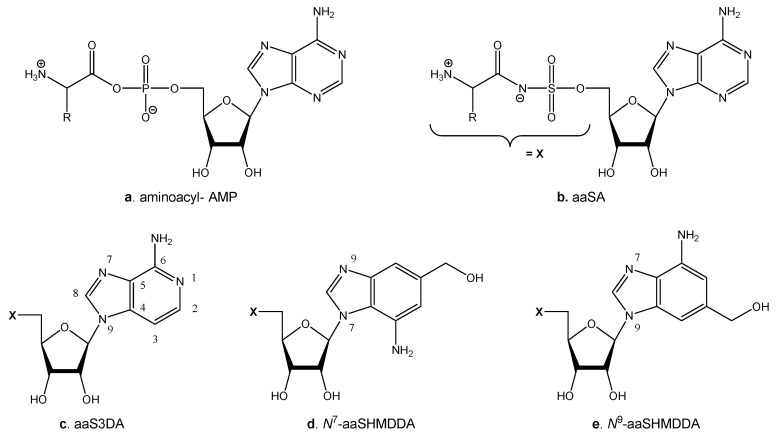
Chemical structures for (**a**) aminoacyl-adenylate; (**b**) aminoacyl-sulfamoyl adenosines; (**c**) aminoacyl-sulfamoyl 3-deazaadenosines; (**d**) the obtained *N*^7^-glycosylated 1,3-dideaza-2-hydroxy-methyl-adenosine (aaS7HMDDA), and (**e**) the desired *N*^9^-glycosylated analogues of 1,3-dideaza-2-hydroxymethyladenine (aaS9HMDDA). Atom numbering according to nucleoside nomenclature.

The aminoacyl-tRNA synthetases (aaRSs) are essential enzymes playing a central role in the translation of the genetic code and hence constitute a viable target for the development of antibiotics [1]. Based on the structure of the catalytic site, two distinct classes of tRNA synthetases can be discerned [2,3]. Both classes activate an amino acid in forming the respective aminoacyl-adenylate (aa-AMP, Figure 1a) as natural reaction intermediates. In view of their resemblance and irrespective of the class, aminoacyl-sulfamoyl adenosine (aaSA, Figure 1b) derivatives are well-known high-affinity aaRS inhibitors, but they lack antibacterial activity due to their low uptake potential [4]. By introducing various linkers or base substitutions on the aaSA scaffold, our group has already made large biochemical and structural efforts for the evaluation of various series of modified aaSA analogues. More recently, this resulted in the synthesis of 3-deazaadenosine derivatives (aaS3DA, Figure 1c) [3]. These proved less prone to cyclonucleoside formation via the attack of the *N^3^*-position on C5′ with the concomitant release of the sulfamate moiety [5]. 

In addition, our group in the past already evaluated several alternative bases, among which was 4-amino-benzimidazole as a possible substitution for adenine [6]. The former proved less potent, resulting from the loss of *N*^3^-hydrogen bonding as seen with adenine. Moreover, upon evaluation of the above-mentioned 3-deazaadenosine analogues, we observed a clear class bias for aaRS enzymes belonging preferentially to class I versus class II. Careful analysis of the available crystallographic structures pointed to the presence of a conserved water molecule promoting base recognition within class II enzymes via H-bonding with *N*^3^ [3]. Therefore, various substituents have been examined by molecular docking, which potentially could restore the enzymatic affinity. 

J. Lee et al. [7] reported a small cavity around the C2-position of adenine from their docking study of IleSA’s interaction with the IleRS enzyme. Preliminary modeling indicated to us that the introduction of a 2-hydroxymethyl group on the 1,3-dideaza-adenine base could afford improved interaction and affinity for the enzyme. The thus-proposed scaffold can be considered as either 4-amino-6-hydroxymethyl-benzimidazole or a 1,3-dideaza-2-hydroxymethyl-adenine ring structure. Moreover, purine ribonucleosides carrying a 2-hydroxymethyl moiety have been prepared in the past by hetero-cyclizations [8,9] or transformations of 2-vinylpurines [10,11]. 2-(hydroxymethyl)-nebularine was reported to exhibit antiviral activity [12], 2-(hydroxymethyl)inosine monophosphate to inhibit IMP dehydrogenase [8], and protected 2-(hydroxymethyl)inosine was used in the construction of inhibitors of GMP synthetase [9]. Interestingly, a recent report of the group of Roper discussed the beneficial effects of, in particular, C2-arylated adenosine congeners as inhibitors of bacterial seryl-tRNA synthetase, attaining selective inhibition with greater than two orders of magnitude compared to that for their human homologue [13].

The proposed deviation of the adenine structure should not deter us, as already in the past, compounds carrying highly modified base moieties have been reported to be highly efficient aaRS inhibitory compounds, as briefly exemplified by the high selectivity of aryl-tetrazole analogues as inhibitors of IleRS [14], and the excellent efficacy of the warhead metabolite of albomycin as a SerRS inhibitor [15]. Both findings proved that altering the adenine base structure while preserving the aaRS inhibitory activity is feasible within both aaRS classes. All this led us to investigate 1,3-dideaza-2-hydroxymethyl-adenosine (HMDDA) as a nucleoside scaffold to further study the structure–activity relationships of these non-hydrolysable aa-AMP mimics for aaRS inhibition. 

However, a series of flipped base derivatives were obtained (resembling an *N*^7^-glycosylated purine, Figure 1d) despite our efforts to generate the resembling *N*^9^-glycosylated purine analogues (Figure 1e). X-ray analysis further confirmed the structures for the flipped-base analogues and showed interesting interactions with the respective aaRS, with a water molecule now H-bonding with the flipped exocyclic amine moiety and substituting for the adenine *N*^3^-water interaction as seen with regular nucleosides interacting with class II enzymes. This series of 5′-aminoacylated sulfamoyl-7-amino-5-hydroxymethyl-benzimidazol-1-yl ribosides (changed numbering as of glycosylation of the alternative nitrogen atom) can be considered as well as close nucleoside analogues being aminoacylated, *N*^7^-glycosylated 2-hydroxymethyl-1,3-dideazaadenine (aaS7HMDDA, Figure 1d) congeners, in contrast with the expected *N*^9^-glycosylated 2-hydroxymethyl-1,3-dideazaadenine (aaS9HMDDA, Figure 1e) analogues. In this study, we describe the synthesis of these new aaRS inhibitors, and the evaluation of their inhibitory potency, and provide details on their 3D enzyme interactions as obtained via X-ray crystallography. 

## 2. Results

### 2.1. Synthesis of aaSHMDDA Analogues

**Scheme 1:**
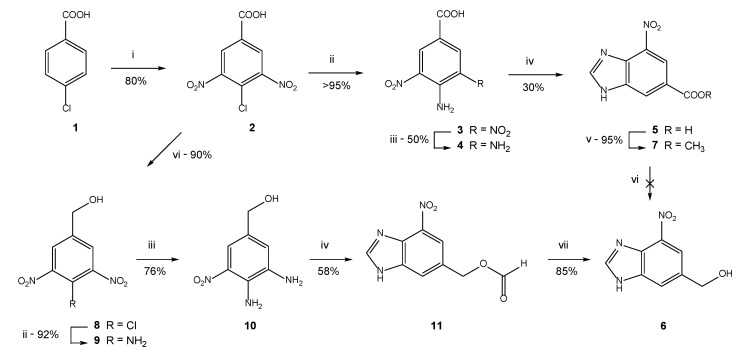
Synthesis of 6-hydroxymethyl-4-nitro-benzimidazole. Reagents and conditions: (i) conc. H_2_SO_4_, KNO_3_, 130 °C; (ii) methanol, aqueous ammonia, 65 °C; (iii) methanol/water (3:1), sodium sulfide, sodium hydrogen carbonate, 75 °C; (iv) formic acid, 115 °C; (v) methanol, SOCl_2_, conc. H_2_SO_4_, reflux; (vi) tetrahydrofuran (THF), boron trifluoride diethyl etherate, borane-THF complex, 50 °C; (vii) 7 N ammonia in methanol.

The initially planned scheme to achieve the synthesis of **6** from cheap and readily available 4-chlorobenzoic acid **1** is shown in Scheme 1. Compound **2** was obtained by the nitration of **1**, followed by treatment with ammonia and substituting an amine for the chlorine atom to afford **3** [16]. The selective reduction of one nitro group using the method described by V. Milata et al. [17] led to the synthesis of **4** with a high yield, using only water as the solvent and inexpensive reagents. The cyclization step to obtain **5** was performed under reflux conditions in formic acid [16]. The yield proved relatively low, most likely because of the presence of the carboxylic acid moiety. An attempt to obtain **6** by reducing the carboxylic acid of compound **5** following the method introduced by Y. Ida et al. [18] was not successful. The esterification of **5** was carried out in methanol in the presence of SOCl_2_ to afford **7** [16]. The same reduction procedure as used for **5** was applied to **7**, but we were still unable to obtain the target compound **6,** and only trace amounts were detected upon mass spectrometric analysis. The failure of the reduction step and the low yields can be ascribed most probably to the acidic moiety, causing mainly problems for work-up and purification.

In view of the perceived difficulties in obtaining **6**, we turned to another route where the carboxylic group was reduced at an earlier stage. The synthesis of **8** was achieved by applying the previous reduction protocol on **2** with a very good yield. Compounds **9**, **10** and **11** were synthesized in analogy with the synthesis of **3** to **5**, respectively, but with improved yields. Unexpectedly, **11** was obtained as the formate, which can be easily hydrolyzed to the desired compound **6** (Scheme 1).

**Scheme 2:**
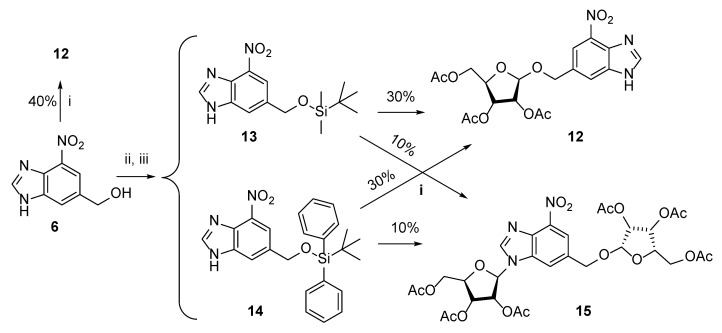
Glycosylation reactions using the unprotected or protected heterocyclic base. Reagents and conditions: (i) dry acetonitrile, 1,2,3,5-tetra-*O*-acetyl-*β-*D-ribofuranose, tin (IV) chloride (1 M in dichloromethane (DCM)), room temperature (rt); (ii) dry acetonitrile, tert-butyldimethylsilyl chloride (TBDMSCl), imidazole, rt; (iii) dry acetonitrile, tert-butyldiphenylsilyl chloride (TBDPSCl), imidazole, rt.

**Scheme 3:**
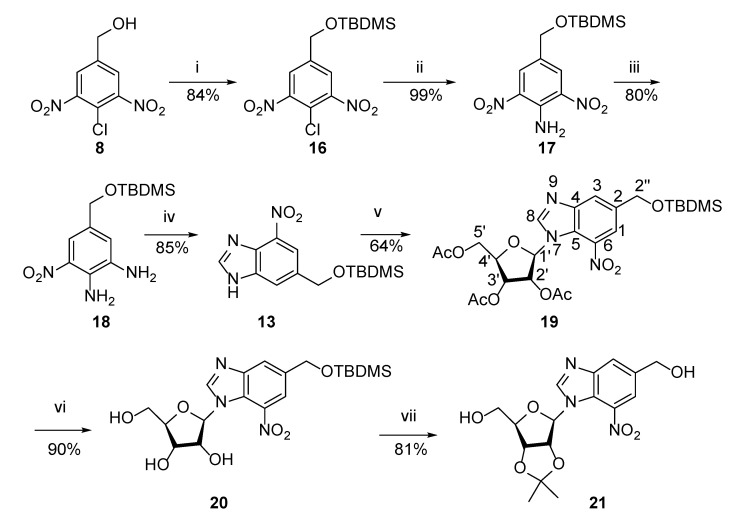
Sugar-base coupling using a TBDMS-protected heterocycle. Reagents and conditions: (i) dry acetonitrile, TBDMSCl, imidazole, rt; (ii) methanol, aqueous ammonia, 65 °C; (iii) methanol/water (3:1), sodium sulfide, sodium hydrogen carbonate, 75 °C; (iv) triethyl orthoformate, formic acid, 145 °C, 6 h; (v) 1,2-dichloroethane, 1,2,3,5-tetra-*O*-acetyl-*β-*D-ribofuranose, trimethylsilyl triflate (TMSOTf), *N*-methylmorpholine, rt.; (vi) 7 N ammonia in methanol, 0 °C to rt 1 h; (vii) *p*-toluenesulphonic acid (TSA), dry acetone, 2,2-dimethoxypropane (DMP), rt, 3 h.

The glycosylation of unprotected **6** with 1,2,3,5-tetra-*O*-acetyl-*β-*D-ribofuranose (Scheme 2) was carried out according to published procedures using stannic chloride [19]. Although the major product was thought to be the desired compound based on mass spectroscopic analysis, extensive 2D NMR analysis confirmed the product to be compound **12** (correlation signals were found for H^1′^ with C^2′’^ and C^1′^ with H^2′’^, following the atom numbering as found in Scheme 3). Likewise, when using tert-butyldimethylsilyl (TBDMS)-protected **13** or tert-butyldiphenylsilyl (TBDPS)-protected **14** under the same conditions, compound **12** remained the major product. Increasing the reaction time afforded the double glycosylated product **15** with a low yield (ratio 3:1 for **12** versus **15**) as confirmed by NMR.

**Figure 2:**
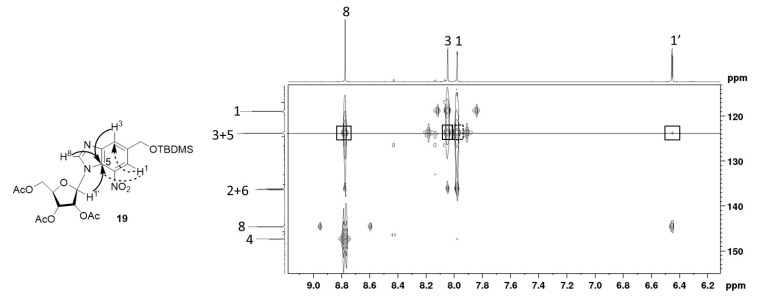
Decisive correlations with C5 on the N7-linked base in compound **19** are indicated by bold arrows in the structure on the left and boxed in the HMBC spectrum. Correlations indicated with a dashed arrow are overlapping (box in dashed lines). Labels correspond to assignments in proton and carbon dimensions.

We next considered whether using the milder glycosylation procedures as described in the silyl-Hilbert-Johnson or Vörbruggen reaction [20] would be advantageous, since the 2-hydroxymethyl protecting group was cleaved in the presence of stannic chloride, leading to the synthesis of **12**. Using trimethylsilyl triflate (TMSOTf) as a Lewis acid for the glycosylation as described by C.P. Ashcroft [21] successfully afforded **19** (Scheme 3). We anticipated compound **19** to be the *N*^9^-glycosylated dideazapurine derivative, but following careful corroboration using 2D NMR at different cut-offs (the correlation signals of H^8^ in the Heteronuclear Multiple Bond Correlation (HMBC) spectrum were missing in the original analysis), we confirmed the assignment as the *N*^7^-glycosylated product. We hereto first identified the base C^5^ having three strong correlation signals with *H*^1^, *H*^3^ and *H*^8^ in HMBC (Figure 2, purine-like numbering is used to highlight the analogy with adenine, see also Scheme 3; IUPAC nomenclature is used in the experimental part). This in turn allowed us to identify the *N*^7^-glycosylation site (*N*^3^-imidazole-coupled sugar if considered as a benzimidazole derivative) via the 2D correlated signal of *H*^1′^ with *C*^5^. X-ray crystallographic data afterwards (see Section 2.3) also confirmed the proposed *N^7^*-dideaza-adenyl glycosylation site, with the electron density maps clearly indicating a flipped base having the exocyclic amine residing at the normal *N^3^*-position. 

Improved solubility of compound **6** was observed following silylation prior to the sugar–base condensation step, which brought us to an improved synthetic route (Scheme 3) for the larger scale synthesis. Hence, TBDMS was introduced on **8** followed by amination, the selective reduction of one of the nitro functionalities, cyclization and glycosylation to finally obtain **19**. The acetate protection of **19** was removed using 7 *N* ammonia solution in methanol to afford **20** with an almost quantitative yield. Having coupled the sugar and the heterocyclic moiety, the next phase of the synthesis required the elaboration of the primary hydroxyl of the sugar part for sulfamoylation. The formation of the acetonide **21** was accomplished with *p*-toluenesulphonic acid in a mixture of acetone in the presence of two equiv. of 2,2-dimethoxypropane (DMP) [5]. However, the undesired concomitant cleavage of the silyl protecting group of the base moiety obviously generates a selectivity problem for the next sulfamoylation reaction, catapulting us back to the start.

**Figure 3:**
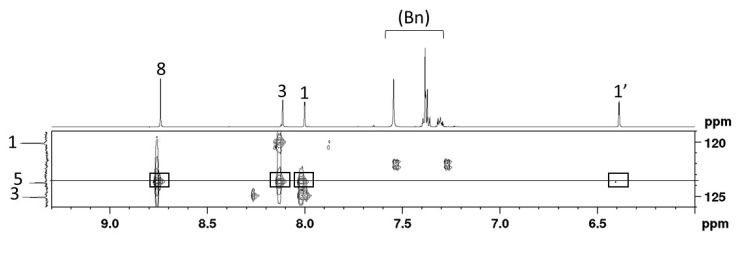
Decisive correlations with C5 on the N7-linked base in compound **29** are boxed in the HMBC spectrum. Labels correspond to assignments in proton and carbon dimensions.

**Scheme 4:**
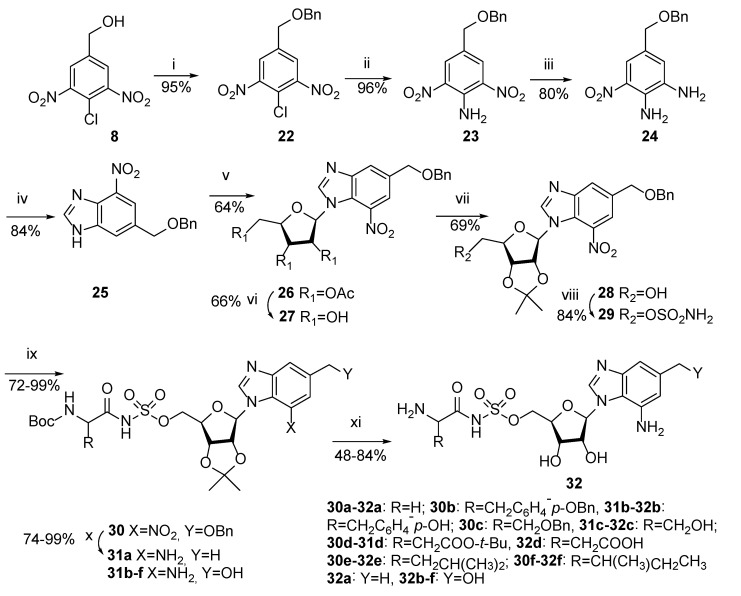
Final optimized route leading to the aaS7HMDDA analogues. Reagents and conditions: (i) dry 1,4-dixone, benzyl 2,2,2-trichloroacetimidate, trifluoromethane-sulfonate, rt; (ii) methanol, aqueous ammonia, 65 °C; (iii) methanol/water (3:1), sodium sulfide, sodium hydrogen carbonate, 75 °C; (iv) triethyl orthoformate, formic acid, 145 °C, 6 h; (v) 1,2-dichloroethane, 1,2,3,5-tetra-*O*-acetyl-*β-*D-ribofuranose, TMSOTf, *N*-methylmorpholine, rt; (vi) 7 N ammonia in methanol, 0 °C to rt, 1 h; (vii) *p*-TSA, dry acetone, DMP, rt, 3 h; (viii) NH_2_SO_2_Cl, acetonitrile, rt, overnight; (ix) Boc-aa-(tBu/Bn)-OSu, 1,8-Diazabicyclo[5.4.0]undec-7-ene (DBU), dry dimethylformamide (DMF), rt, overnight; (x) Pd/C, methanol, H_2_ atm. rt, overnight; (xi) trifluoroacetic acid (TFA)/H_2_O, 5:2 (*v/v*).

The encountered incompatibility finally led us to the use of the more stable benzyl group for the protection of the base moiety, and the synthesis of benzyl-protected 6-hydroxymethyl-4-nitro-benzimidazole (**25**) starting from **8** (Scheme 4). The benzyl protection of the primary alcohol of **8** using established procedures [22] to afford **22** was followed by amination, the selective reduction of one nitro group, cyclization, glycosylation and the hydrolysis of the acetate groups to give compounds **23**, **24**, **25, 26** and **27**, respectively. The acetonide protection of **27** was performed in analogy to **21** while preserving the benzyl protecting group. The direct sulfamoylation of **28** with sulfamoyl chloride (prepared in situ by the reaction of chlorosulfonyl isocyanate with formic acid) led to the synthesis of **29** [23]. The 2D NMR of compound **29** (Figure 3) clearly showed an interaction between *H*^1′^ and *C*^5^ in HMBC, indicating the *N*^7^-glycosylated **26** (using purine numbering) to be the major product upon glycosylation under Hilbert-Johnson conditions. No minor products were isolated.

The aminoacyl-sulfamate analogues **30a**–**f** were obtained by the coupling of the respective *N*-hydroxysuccinimide ester of appropriately protected amino acids with the key sulfamoyl compound **29** using DBU as a base in DMF (Scheme 4). The removal of the 2-hydroxymethyl-protecting benzyl moiety concomitant with the deprotection of the amino acid (serine and tyrosine specifically), as well as the reduction of the 6-nitro group, was accomplished by reductive hydrogenation with 10% Degussa-type Pd/C. Surprisingly, via the NMR and mass spectrometric analysis of compound **31a**, we unexpectedly observed a dehydroxylated compound (with concomitant reduction to a *C*2-methylated base moiety). Benzylic alcohols can be reduced to the corresponding alkyl groups under simple hydrogenolysis conditions, for instance, with hydrogen gas using Pd/C as a catalyst. This happens in a limited number of cases upon the cleavage of benzyl ethers, a common protecting group. Of all six Pd/C-treated compounds **31a**–**f**, only the glycine analogue **31a** proved to be reduced. Tert-butyloxycarbonyl and acetonide groups were cleaved using a TFA/water (5:2) mixture to afford the final compounds **32a**–**f** (aaS7HMDDA and GlyS7MDDA) [6]. All the compounds synthesized in this project were unambiguously characterized by one or more means of ^1^H, ^13^C NMR spectroscopy and electrospray ionization (ESI)-MS.

### 2.2. Measurement of In Vitro Enzymatic Inhibitory Activity with Purified E. coli aaRSs

**Figure 4:**
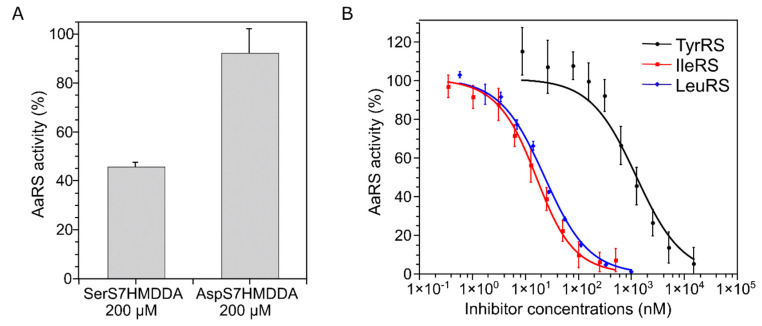
In vitro enzymatic inhibitory activity. (**A**): Inhibitory activity of HMDDA derivatives targeting SerRS and AspRS (class II enzymes) at high concentration. (**B**): Dose-response curves of HMDDA derivatives targeting class I enzymes IleRS, LeuRS and TyrRS. The activity of each enzyme is reported as a percentage value relative to that measured in the absence of inhibitor. The presented fit of the measured points was calculated using the Greco-Hakala equation [24]. Averages of three experiments with SD error bars are shown.

The five aaS7HMDDA analogues (the glycine derivative was left out) were evaluated for their ability to inhibit the aaRS-catalyzed aminoacylation of tRNA using the corresponding isolated *E. coli* aaRS (Figure 4). In the case of the class I-targeting compounds, low-nanomolar $K_{i}^{app}$ were obtained for the 7HMDDA derivatives **32e**–**f** and 1.2 µM for **32b** targeting TyrRS. While these values reflect high affinity for the target enzymes, the inhibition is 5 to 420-fold lower compared to that with the original aaSA analogue (Table 1). This contrasts with the inhibitory activity noted for the congeners targeting class II AspRS and SerRS, as only 9% and 54% inhibitory activity, respectively, was observed at a 200 µM inhibitor concentration (Figure 4A). 

### 2.3. Crystallographic Analysis

**Figure 5:**
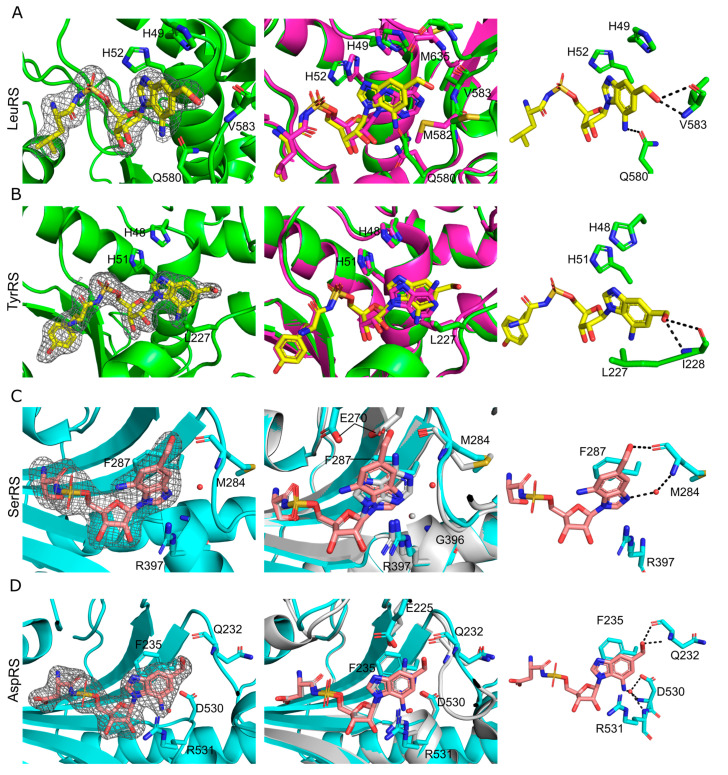
X-ray structures of aaS7HMDDA bound to the corresponding aaRS of class I (green) and class II (cyan). (**A**) *N. gonorrhoeae* LeuRS in complex with LeuS7HMDDA; (**B**) *E. coli* TyrRS in complex with TyrS7HMDDA; (**C**) *K. pneumoniae* SerRS in complex with SerS7HMDDA; (**D**) *T. thermophilus* AspRS in complex with AspS7HMDDA. Left: electron density map for the ligand; Middle: superposition of aaS7HMDDA and aaSA bound structures; Right: protein–aaS7HMDDA interactions. Protein structures are presented as cartoon representations. The ligand and interacting residues are shown in stick representations. A conserved structured water molecule in SerRS and AspRS is shown as a sphere.

To further investigate the structure-activity relationship (SAR), X-ray crystal structures of an aaRS in complex with the corresponding synthesized HMDDA analogues were determined (Figure 5 and Table 2). As shown in Figure 5, the compound was unambiguously built inside the active site of tRNA synthetase according to the electron density map, which confirmed the conformation of the flipped base. With the present modification, in most cases, the amine group occupies the place where the *N*^3^ of adenine is normally residing and mimics its interaction with the protein. This results in the HMDDA base rotating towards the ribose when compared to the conformation of the canonical bound adenine (Figure 5). 

In our previous work, we discussed in detail the interactions between the adenine base and the respective class I and class II enzymes [3]. In the case of class I aaRSs, only two polar interactions with the base are consistently observed for the different aaRS:aaSA complex structures, mediated by the interaction of the protein backbone atoms with the *N*^1^ and *N*^6^ atoms of adenine. In this work, the flipped base loses part of these important interactions, providing a good rationale for their lower activity compared with aaSA (Figure 5A,B). As seen in LeuRS, the amine group of the HMDDA base is involved in a H-bond with the side chain of Gln580, which originally interacts with N^3^ of adenine, but loses a stacking interaction with the side chain of Met582 and the polar contact with the backbone of Met635 as observed in the equivalent adenine congener (Figure 5A). In addition, the hydroxymethyl group of the base makes H-bonds with backbone atoms of Val583 in LeuRS or Ile228 in TyrRS, respectively. 

For class II aaRSs, the adenine makes numerous interactions with active site residues, with the *N*^1^, *N*^3^ and *N*^6^ all making H-bond interactions with conserved features shared amongst all class II aaRS members. The interaction between the N^3^ of adenine with the conserved water molecule for class II aaRS enzymes is crucial for the recognition of aaSAs by the corresponding enzymes [25]. It was hypothesized that the flipped exocyclic amine moiety of HMDDA should be able to mimic this specific interaction. However, in the case of SerRS, the base moiety adopts the *syn*-conformation relative to the ribose defined by the torsion angle (*O^4′^*-*C^1′^*-*N^7^*-*C^8^*) of −124°, while the base bound in the active site of class I LeuRS and TyrRS and class II AspRS remains in the *anti*-conformation (Figure 5). In this specific *syn* conformation in SerRS, the hydroxymethyl group of the base forms a direct H-bond with the carbonyl oxygen of Met284, and N9 makes an indirect contact with the backbone nitrogen of Met284 via a water bridge (Figure 5C). Despite the HMDDA base making some interactions with surrounding protein residues, compared with the adenine congener, the lack of H-bonds mediated by *N^6^* and *N^3^* of adenine with the conserved Glu270 and structural water molecule leads to a detrimental effect on its inhibitory activity. Although in the case of AspRS, the position of the HMDDA base overlaps with the natural adenine base, all the original interactions of the *N*^6^ as seen for the equivalent aaSA are still lost with AspS7HMDDA, similarly to what is seen with SerRS (Figure 5D). Furthermore, the N^6^ amine group creates an unfavorable bond with the class II conserved structured water. This could explain the more significant decrease in inhibitory activity compared with that seen for class I aaRSs.

**Table 2:** Data collection and refinement statistics for the crystal structures of aaRS in complex with aaS7HMDDA.

	SerRS-SerS7HMDDA	LeuRS-LeuS7HMDDA	AspRS-AspS7HMDDA	TyrRS-TyrS7HMDDA
**Data collection**				
Resolution range (Å)	19.96–2.18 (2.26–2.18)	56.25–2.25 (2.33–2.25)	79.53–2.15 (2.23–2.15)	52.6–1.998 (2.07–1.998)
Space group	*P* 4_3_ 2_1_ 2	*P* 2_1_ 2_1_ 2_1_	*P* 1 2_1_ 1	*P* 1 2_1_ 1
Unit cell	84.7 84.7 229.9 90 90 90	49.2 81.0 225.0 90 90 90	82.3 112.5 88.3 90 104.9 90	82.2 65.1 91.0 90 101.3 90
Total reflections	537,775 (53,341)	565,139 (56,028)	311,929 (31,077)	229,644 (22,423)
Unique reflections	44,514 (4326)	43,755 (4274)	83,442 (8289)	63,719 (6342)
Multiplicity	12.1 (12.3)	12.9 (13.1)	3.7 (3.7)	3.6 (3.5)
Completeness (%)	99.50 (99.49)	99.99 (100.00)	98.84 (98.57)	99.21 (99.15)
Mean *I*/σ (*I*)	15.45 (2.01)	10.91 (2.00)	10.57 (2.37)	7.39 (1.28)
Wilson B factor (Å^2^)	50.0	41.6	46.49	29.66
*R*_merge_	0.0828 (1.198)	0.1624 (1.407)	0.06744 (0.535)	0.1068 (0.971)
*R*_meas_	0.0866 (1.25)	0.1692 (1.465)	0.07833 (0.62)	0.1253 (1.14)
*R*_pim_	0.02507 (0.3546)	0.04709 (0.4034)	0.03934 (0.3139)	0.06464 (0.5966)
CC_1/2_	0.999 (0.941)	0.998 (0.713)	0.996 (0.837)	0.997 (0.686)
**Refinement**				
Reflections used for R_free_	2044 (206)	2006 (198)	2055 (217)	2465 (245)
*R*_work_	0.1889 (0.2867)	0.1873 (0.2827)	0.1831 (0.2553)	0.2028 (0.3036) (0.3014)
*R*_free_	0.2163 (0.2789)	0.2355 (0.3466)	0.2304 (0.2927)	0.2531 (0.3343) (0.3307)
Number of non-H atoms	3511	6732	9374	6985
Macromolecules	3347	6476	8998	6428
Ligands	31	33	76	74
Solvent	118	213	300	481
Protein residues	421	843	1153	826
RMS bonds (Å)	0.012	0.004	0.012	0.007
RMS angles (°)	1.56	0.65	1.16	0.82
Ramachandran favored (%)	97.11	96.65	96.41	97.44
Ramachandran allowed (%)	2.89	3.23	3.32	2.46
Rotamer outliers (%)	1.4	0.16	2.67	0.12
Clashscore	1.34	4.28	5.52	4.56
Average B-factor (Å^2^)	61.19	49.49	69.85	41.46
Protein	61.22	49.69	70.08	41.61
Inhibitor	52.88	36.44	59.50	30.65
Solvent	60.56	45.51	55.33	41.20

Statistics were generated using Phenix [26]; values in parenthesis correspond to the highest resolution shell.

## 3. Discussion

There is a clear precedent in the synthesis and application of deazapurines in medicinal chemistry [5,27,28]. Within our research group, Gadakh. et al. [6] reported one deazapurine 5′-*O*-(*N*-isoleucyl)sulfamoyl-1,3-dideazaadenosine as an IleRS inhibitor, but unfortunately, there was a significant loss of inhibitory activity compared with the IleSA compound. However, Lee et al. [7] reported a small cavity around the C2-position of adenine based on their docking study of IleSA’s interaction with the IleRS enzyme, suggesting to us that introducing a 2-hydroxymethyl group probably could improve the interaction with the targeted enzymes. Based on this assumption, we planned to synthesize a series of six compounds using this 1,3-dideaza-2-hydroxymethyladenine scaffold and targeting representatives of both classes of aaRSs. The removal of the *N*^3^ atom as in 3-deazaadenine should enhance the chemical stability, and according to modelling, the inhibitory activity should improve via interactions of the Supplementary Materials hydroxymethyl moiety with the target enzyme. In combination with 3-deazaadenosine [3], this scaffold should provide us with a more comprehensive understanding of the SAR properties of the base moiety.

The synthesis of the target compounds proved to be not straightforward. At first, we tried to carry out the reactions without protecting the carboxylic acid group of 4-chloro-benzoic acid but failed to yield the desired compound **6** (as shown in Scheme 1), while also, the yield for the cyclization step was low. Secondly, we applied another synthetic route where the reduction of the carboxylic group was carried out at the second step, and we successfully obtained the desired compound **6** with a moderate yield. However, when applying the standard glycosylation procedure, the sugar moiety preferentially coupled to the hydroxymethyl group as shown in Scheme 2. Upon the protection of the hydroxyl moiety with tert-butyldimethylsilyl chloride (TBDMSCl) immediately following the reduction of the carboxylic group (Scheme 3), we managed to synthesize the base with an increased overall yield and also succeeded in the glycosylation of the desired heterocycle. Unfortunately, this TBDMS protection was cleaved when trying to protect the 2′,3′-hydroxyl moieties with the isopropylidene group, and obviously, the resulting presence of two primary alcohols provides a selectivity issue in the following sulfamoylation step. We finally opted for the more stable benzyl group for the protection of the base moiety and successfully obtained the targeted final compounds (Scheme 4). The benzyl group was cleaved simultaneously with the reduction of the nitro group using hydrogenation with a Pd/C catalyst. Surprisingly, only for **32a**, the anomalous reduction of the benzyl ether afforded a methyl group instead of the expected hydroxymethyl moiety, for which we have no explanation but which seems to be related to the unbranched glycine.

The *N*^7^-1,3-dideazapurine glycosylation site was unexpected but was firmly corroborated via extensive HMBC correlation studies, and further confirmed by the X-ray structures of aaRSs in complex with their corresponding inhibitors. The biological activity of aaSHMDDA analogues showed that all the newly synthesized compounds were less potent than the respective aaSAs. However, while inhibitors targeting class I aaRSs still exhibit lower nanomolar inhibitory activity, especially for class I IleRS and LeuRS, the respective compounds targeting class II enzymes lost their inhibitory activity even at the higher concentration of 200 µM. 

To provide further insight into the SAR of the molecules, the X-ray crystal structures of the compounds with their respective aaRSs were solved. When comparing the structures of the aaRSs in complex with their corresponding aaS7HMDDA analogues, it is clear that this flipped base only can replicate the interactions of *N^3^* in adenine by the amine in aaS7HMDDA but loses almost all the important interactions generated by *N^1^* and *N^6^* in adenine due to the flipped orientation of the base. The Supplementary Materials hydroxymethyl moiety obviously is not located at the originally intended position but makes polar interactions with the backbone of contacting protein residues. For both LeuRS and TyrRS, we note these H-bonds mediated by the hydroxymethyl moiety, and for IleRS, crystallographic data are not available.

Nevertheless, IleS7HMDDA (**32f**) is only five times less inhibitory than the well-known inhibitor IleSA, which surprisingly is 10-fold better compared to the 3-deaza derivative IleS3DA [3]. The compound also outperforms the pyrimidine analogues previously reported [25]. By contrast, the inhibitory activity for the leucine analogue **32e** is analogous to its pyrimidine congeners, but for LeuRS, the LeuS3DA congener almost matched the strong activity of LeuSA. While TyrS7HMDDA was about 400 times less inhibitory compared to TyrSA, the 3DA and the uracil analogue only gave 10-fold and 15-fold reductions in activity, respectively, while the cytosine congener also suffered a 400-fold decreased inhibitory activity. It is clear that the subtle differences between these class I aaRSs structures can have significantly different effects on the inhibitory activity of the corresponding inhibitors sharing similar scaffolds. However, in class II aaRSs, two different base conformations were observed, where the base adopts an *anti*-conformation in AspRS as seen in class I aaRSs but a *syn* conformation in SerRS. This is likely the result of the distinct flexibility of their motif-2 loop regions. A comparison of ligand-free and ligand-bound states shows that the motif-2 loop is in a relative fixed position (a closed conformation) in SerRS independently of inhibitors binding, while this loop region in AspRS is more dynamic, which has been described in detail in our previous work [29]. In the holo-enzyme of AspRS, the motif-2 loop region adopts an open conformation and only changes to the closed conformation upon the binding of adenine-containing compounds. Both HMDDA base conformations in SerRS and AspRS either lose the additional conserved structural water-mediated H-bonds as observed for the *N^3^* of adenine or make unfavorable interactions with structured water due to the presence of protons at the *N^6^*-amine moiety. This could further explain why this scaffold is more disruptive for binding for class II than for class I inhibitors. This highlights again that the extensive modification of the heterocyclic base can be tolerated, especially for the inhibition of class I aaRS enzymes, as highlighted as well with the strongly inhibiting pyrimidine nucleosides reported by Nautiyal et al. [25].

## 4. Conclusions

A series of six *N*^7^-glycosylated aminoacyl sulfamoyl-2-hydroxymethyl-1,3-dideazaadenosine analogues were synthesized with three compounds, each targeting both classes of aaRSs. An optimized chemical route hereto was elaborated to obtain the desired heterocyclic base, and the glycosylation site was firmly established via extensive 2D NMR and crystallographic studies. In vitro evaluation using purified aaRS enzymes showed, especially, class I-targeting compounds to be active, with, in particular, **32e** and **32f** displaying nanomolar inhibitory activity against LeuRS and IleRS, respectively. Structural analysis showed the flipped 2-hydroxymethyl-1,3-dideazaadenine base is partially mimicking the interactions of adenine within both series of aaRS enzymes. 

## 5. Experimental Section

### 5.1. Reagents and Methods

The reagent qualities and sources and general methods were as described before [3,25].

#### 5.1.1. 4-Chloro-3,5-Dinitrobenzoic Acid (**2**) 

4-chloro-benzoic acid **1** (20 g, 0.128 mol) was dissolved in H_2_SO_4_ (d = 1.835 g/mL, 300 mL) at 80 °C, and KNO_3_ (66 g, 0.65 mol) was added. The reaction mixture was heated to 125 °C using a high-pressure flask and kept for 2 h, after which the reaction was cooled to rt and poured onto ice. The yield of title compound **2** was 28 g (89%) [16]. ^1^H NMR (300 MHz, DMSO-*d*_6_) δ = 14.29 (s, 1H), 8.76 (s, 2H). ^13^C NMR (75 MHz, DMSO-d6) δ 163.61, 149.06, 132.20, 128.70, 122.80. HRMS (ESI): *m*/*z* calcd. for C_7_H_2_ClN_2_O_6_ [M−H]^−^: 244.9607; found, 244.9607.

#### 5.1.2. 4-Amino-3,5-Dinitrobenzoic Acid (**3**) 

Compound **2** (20 g, 81 mmol) was dissolved in methanol (100 mL), and aqueous 24% NH_3_ (120 mL) was gradually added. The reaction mixture was stirred at rt for 2.5 h, refluxed for 3 h and left at rt for around 14 h. The precipitate that formed was filtered off, and the filtrate was evaporated to dryness. Water (10 mL) and HCl (10 mL) were added to the solid residue, which was combined with the precipitate. After stirring for 10 min, the precipitate was filtered off and washed with water till washings showed neutral. The yield of 4-amino-3,5-dinitrobenzoic acid **3** was 18 g (98%) [16]. ^1^H NMR (300 MHz, Acetone-*d*_6_) δ = 9.16–9.01 (m, 3H), 9.01–8.84 (s, 2H). ^13^C NMR (75 MHz, Acetone-*d*_6_) δ 163.49, 143.33, 134.76, 133.75, 128.17, 115.39. HRMS (ESI): *m*/*z* calcd. for C_7_H_4_N_3_O_6_ [M−H]^−^: 226.0105; found, 226.0100.

#### 5.1.3. 3,4-Diamino-5-Nitrobenzoic Acid (**4**)

A suspension of freshly made compound **3** (10 g, 44 mmol) in water (200 mL) was heated to 60 °C with stirring for about 10 min. To this suspension was then added a solution of sodium sulfide (11.2 g, anhydrous) and sodium bicarbonate (11.5 g) in water (250 mL). The reaction mixture was stirred at 75 °C for 1 h and left to cool overnight, after which it was concentrated and subjected to silica gel column chromatography to give a deep red solid compound **4** with a yield of 74% [17]. ^1^H NMR (300 MHz, DMSO-*d*_6_) δ 12.56 (s, 1H), 7.98 (d, *J* = 1.9 Hz, 1H), 7.40 (s, 2H), 7.30 (d, *J* = 1.9 Hz, 1H), 5.47 (s, 2H). ^13^C NMR (75 MHz, DMSO-*d*_6_) δ 166.71, 138.09, 137.74, 130.25, 117.66, 116.01, 115.78. HRMS (ESI): *m*/*z* calcd. for C_7_H_6_N_3_O_4_ [M−H]^−^: 196.0364; found, 196.0367.

#### 5.1.4. 4-Nitro-1H-Benzo[d]imidazole-6-Carboxylic Acid (**5**)

Compound **4** (2 g, 10.15 mmol) was refluxed for 6 h in 50 mL of formic acid. The reaction mixture was concentrated, and then, 10 mL of 30% aqueous NaOH was slowly added to the residue. The resulting suspension was portioned between 30 mL of ethyl acetate and 20 mL of water 3 times; the combined ethyl acetate layer was concentrated and subjected to a silica gel column to obtain 600 mg of **5** with a 29% yield [30]. ^1^H NMR (300 MHz, DMSO-*d*_6_) δ = 13.54 (s, 2H), 8.64–8.57 (m, 2H), 8.56 (d, *J* = 1.5 Hz, 1H). ^13^C NMR (75 MHz, DMSO-*d*_6_) δ 166.25, 147.16, 133.43, 126.95, 124.45, 119.75. HRMS (ESI): *m*/*z* calcd. for C_8_H_4_N_3_O_4_ [M−H]^−^: 206.0207; found, 206.0208.

#### 5.1.5. (4-Nitro-1H-Benzo[d]imidazol-6-yl)-Methanol (**6**)

Compound **11** (160 mg, 0.72 mmol) was dissolved in methanolic ammonia (7N, 15 mL) and stirred at 25 °C for 1.5 h. After TLC showed the completion of the reaction, the mixture was concentrated, and 10 mL of DCM was added. Undissolved solid compound was filtered, and 123 mg of the title product **6** was collected with a yield of 88%. ^1^H NMR (300 MHz, DMSO-*d*_6_) δ 8.43 (s, 1H), 8.14 (d, *J* = 1.3 Hz, 1H), 8.06 (d, *J* = 1.3 Hz, 1H), 4.70 (s, 2H). ^13^C NMR (75 MHz, DMSO-*d*_6_) δ 145.57, 136.64, 133.41, 131.48, 127.20, 124.24, 117.34, 62.28. HRMS (ESI): *m*/*z* calcd. for C_9_H_8_N_3_O_4_ [M + H]^+^: 194.0560; found, 194.0565.

#### 5.1.6. Methyl 4-Nitro-1H-Benzo[d]imidazole-6-Carboxylate (**7**)

To a mixture of 100 mg of **5** in 10 mL of methanol was added 3 drops of concentrated sulfuric acid, and the reaction was refluxed for 2 h until TLC indicated the reaction to be completed. A solution of 20 mL of saturated sodium bicarbonate was added to the concentrated reaction mixture, which was extracted with 30 mL of ethyl acetate. The title compound **7** was separated by silica gel chromatography to afford 97 mg (92% yield). ^1^H NMR (300 MHz, DMSO-*d*_6_) δ 8.58 (d, *J* = 15.1 Hz, 3H), 3.93 (s, 3H). ^13^C NMR (75 MHz, DMSO-*d*_6_) δ 165.20, 147.55, 145.51, 133.53, 131.11, 126.61, 122.94, 119.45, 52.75. HRMS (ESI): *m*/*z* calcd. for C_9_H_8_N_3_O_4_ [M + H]^+^: 222.0509; found, 222.0513.

#### 5.1.7. (4-Chloro-3,5-Dinitrophenyl)-Methanol (**8**)

To a solution of compound **2** (3 g, 12.2 mmol) in dry THF (30 mL) at ambient temperature and under a nitrogen atmosphere was added boron trifluoride diethyl etherate (12 mL), followed by the addition of borane tetrahydrofuran complex (1.0 M solution in THF, 24 mL) over 5 min. The mixture was stirred at ambient temperature overnight, and the reaction was quenched by carefully adding 10 mL of methanol until the gas evolution ceased, followed by 20 mL of water. The mixture was concentrated, the residue was extracted with ethyl acetate (3 × 30 mL), and the combined organic extracts were washed with brine. The title compound **8** was separated with silica gel, affording 2.48 g (87%). ^1^H NMR (300 MHz, DMSO-*d*_6_) δ 8.31 (d, *J* = 0.9 Hz, 2H), 4.65 (s, 2H). ^13^C NMR (75 MHz, DMSO-*d*_6_) δ 148.75, 145.89, 128.71, 125.89 (two carbons), 116.11, 60.96. HRMS (ESI): *m*/*z* calcd. for C_7_H_6_ClN_2_O_5_ [M + H]^+^: 232.9960; found, 232.9959.

#### 5.1.8. (4-Amino-3,5-Dinitrophenyl)-Methanol (**9**)

Compound **8** (2.3 g, 9.95 mmol) was dissolved in methanol (100 mL), and aqueous NH_3_ (20 mL) was gradually added. The reaction mixture was stirred at rt for 2.5 h, refluxed for 3 h and left at ambient temperature overnight. Following concentration, the reaction mixture was subjected to a silica gel column, affording 1.8 g (92%) of the title compound **9**. ^1^H NMR (300 MHz, DMSO-*d*_6_) δ 8.43 (s, 2H), 8.35 (s, 2H), 5.49 (t, *J* = 5.8 Hz, 1H), 4.48 (d, *J* = 5.7 Hz, 2H). ^13^C NMR (75 MHz, DMSO-*d*_6_) δ 139.95, 134.68, 131.80 (two carbons), 128.45, 125.87, 60.85. HRMS (ESI): *m*/*z* calcd. for C_7_H_6_N_3_O_5_ [M−H]^−^: 212.0313; found, 212.0316.

#### 5.1.9. (3,4-Diamino-5-Nitrophenyl)-Methanol (**10**)

A suspension of **9** (2.1 g, 9.85 mmol) in MeOH (40 mL) was heated to 60 °C with stirring for about 10 min. To this suspension was added a solution of sodium sulfide (5.4 g of the nonahydrate, 22.14 mmol) and sodium bicarbonate (3.5 g, 41.7 mmol) in 60 mL of water. The reaction mixture was stirred at 70–75 °C for 3 h. The reaction was concentrated and subjected to silica gel chromatography, giving 1.34 g (76%) of the title compound **10**. ^1^H NMR (300 MHz, DMSO-*d*_6_) δ 7.29 (d, *J* = 1.7 Hz, 1H), 6.91 (s, 2H), 6.77 (d, *J* = 1.8 Hz, 1H), 5.24 (s, 2H), 5.07 (t, *J* = 5.7 Hz, 1H), 4.30 (d, *J* = 5.6 Hz, 2H). ^13^C NMR (75 MHz, DMSO-*d*_6_) δ 137.74, 134.60, 130.67, 130.09, 116.41, 110.56, 62.52. HRMS (ESI): *m*/*z* calcd. for C_7_H_8_N_3_O_3_ [M−H]^−^: 182.0571; found, 182.0571.

#### 5.1.10. (4-Nitro-1H-Benzo[d]imidazol-6-yl)-Methyl Formate (**11**)

A suspension of **10** (100 mg, 0.55 mmol) in 10 mL of formic acid was heated to 115 °C for 3 h, after which the reaction was concentrated, followed by adding 10 mL of ethyl acetate and 1 mL of triethylamine (TEA). The title compound was purified by silica gel column chromatography, and 70 mg of white compound **11** was collected with a yield of 58%. ^1^H NMR (300 MHz, DMSO-*d*_6_) δ 13.36 (s, 1H), 8.49 (s, 1H), 8.36 (s, 1H), 8.20 (d, *J* = 5.0 Hz, 2H), 5.38 (s, 2H). ^13^C NMR (75 MHz, DMSO-*d*_6_) δ 162.06, 146.13, 129.42, 119.21, 64.46. HRMS (ESI): *m*/*z* calcd. for C_9_H_8_N_3_O_4_ [M + H]^+^: 222.0509; found, 222.0513.

#### 5.1.11. (2R,3R,4R,5R)-2-(Acetoxymethyl)-5-((4-Nitro-1H-Benzo[d]imidazol-6-yl)methoxy)tetrahydrofuran-3,4-Diyl Diacetate (**12**)

Compound **6** (110 mg, 0.57 mmol) and 1,2,3,5-tetra-*O*-acetyl-*β*–D-ribofuranose (280 mg, 0.63 mmol) were dissolved in 15 mL of dry acetonitrile, followed by adding a solution of stannic chloride (1 M in DCM, 3 mL) into the reaction mixture. After stirring at 25 °C for 16 h, the reaction mixture was diluted with DCM and then poured under stirring into 60 mL of ice-cooled saturated sodium bicarbonate. The resulting suspension was filtered through celite, and the layers were separated. The organic layer was further washed with brine and was dried over Na_2_SO_4_, filtered off and evaporated. The residue was purified by silica gel column chromatography to yield 100 mg (40%) of the title compound **12**. ^1^H NMR (300 MHz, DMSO-*d*_6_) δ 13.32 (s, 1H), 8.47 (s, 1H), 8.14 (d, *J* = 4.5 Hz, 2H), 5.34–5.15 (m, 3H), 4.89 (d, *J* = 11.9 Hz, 1H), 4.74 (d, *J* = 11.8 Hz, 1H), 4.30 (dt, *J* = 10.2, 3.7 Hz, 2H), 4.18–3.96 (m, 1H), 2.17–1.92 (m, 9H). ^13^C NMR (75 MHz, DMSO-*d*_6_) δ 170.18, 169.70, 169.53, 145.92, 131.14, 118.74, 104.32, 78.36, 74.20, 71.11, 68.48, 63.92, 20.62, 20.48, 20.44. HRMS (ESI): *m*/*z* calcd. for C_19_H_22_N_3_O_10_ [M + H]^+^: 452.1300; found, 452.1303.

#### 5.1.12. 6-((Tert-Butyldimethylsilyloxy)methyl)-4-Nitro-1H-Benzo[d]imidazole (**13**)

The alcohol derivative **6** (100 mg, 0.52 mmol) and TBDMSCl (94 mg, 0.63 mmol) were dissolved in 15 mL of dry acetonitrile, and the reaction mixture was stirred at 25 °C for 20 h. After the completion of the reaction, the mixture was extracted with ethyl acetate (EA) (20 mL) directly, the organic layer was further washed with brine, and the organic layer was subjected to a silica gel column to give 117 mg of title compound **13** with a 74% yield. ^1^H NMR (300 MHz, DMSO-*d*_6_) δ 13.26 (s, 1H), 8.45 (d, *J* = 2.0 Hz, 1H), 8.10 (d, *J* = 20.1 Hz, 2H), 4.92 (d, *J* = 2.1 Hz, 2H), 0.93 (d, *J* = 2.2 Hz, 9H), 0.12 (d, *J* = 2.4 Hz, 6H). ^13^C NMR (75 MHz, DMSO-*d*_6_) δ 145.71, 135.26, 116.85, 63.61, 25.94, 18.13, −5.12. HRMS (ESI): *m*/*z* calcd. for C_14_H_22_N_3_O_3_Si [M + H]^+^: 308.1425; found, 308.1428.

#### 5.1.13. 6-((Tert-Butyldiphenylsilyloxy)methyl)-4-Nitro-1H-Benzo[d]imidazole (**14**)

This compound was synthesized in analogy to **13** using TBDPSCl from 100 mg (0.52 mmol) of **8**. Yield: 89%. ^1^H NMR (300 MHz, DMSO-*d*_6_) δ 8.46 (s, 1H), 8.18 (s, 1H), 8.10 (s, 1H), 7.66 (dt, *J* = 6.1, 1.7 Hz, 4H), 7.57–7.34 (m, 7H), 4.99 (d, *J* = 1.8 Hz, 2H). ^13^C NMR (75 MHz, DMSO-*d*_6_) δ 146.38, 145.72, 135.15, 134.52, 132.82, 130.15, 128.13, 124.47, 116.89, 64.48, 26.80, 19.01. HRMS (ESI): *m*/*z* calcd. for C_24_H_26_N_3_O_3_Si [M + H]^+^: 432.1739; found, 432.1740.

#### 5.1.14. (2R,3R,4R,5R)-2-(Acetoxymethyl)-5-((1-((2R,3R,4R,5R)-3,4-Diacetoxy-5-(Acetoxymethyl)tetrahydrofuran-2-yl)-4-Nitro-1H-Benzo[d]imidazol-6-yl)methoxy)tetrahydrofuran-3,4-Diyl Diacetate (**15**)

The double sugar-containing compound was obtained using the procedure for **12** by changing the reaction temperature to 60 °C. However, compound **12** remained the major product in this reaction, no matter whether we started from the protected compound **13** or **14.** The ratio for compounds **12** and **15** was 3:1, with an overall yield of 40%. ^13^C NMR (75 MHz, DMSO-*d*_6_) δ 170.19, 169.68, 169.62, 169.52, 169.40, 146.09, 138.70, 136.45, 135.39, 132.96, 119.09, 117.32, 104.30, 86.55, 79.92, 78.41, 74.18, 72.40, 71.10, 69.67, 68.33, 63.90, 63.00, 20.62, 20.56, 20.51, 20.46, 20.42, 20.29. HRMS (ESI): *m*/*z* calcd. for C_30_H_36_N_3_O_17_ [M + H]^+^: 710.2039; found, 710.2040.

#### 5.1.15. Tert-Butyl(4-chloro-3,5-Dinitrobenzyloxy)dimethylsilane (**16**)

This compound was synthesized in analogy to **13** (which was synthesized from **6**) starting from 500 mg (2.59 mmol) of **8**. Yield: 84%. ^1^H NMR (300 MHz, CDCl_3_) δ 8.57 (d, *J* = 1.0 Hz, 2H), 5.44 (t, *J* = 1.0 Hz, 2H), 1.58 (s, 9H), 0.77 (s, 6H). ^13^C NMR (75 MHz, CDCl_3_) δ 149.76, 144.05, 124.99, 120.66, 118.50, 63.19, 62.94, 26.08, 18.59, −5.11. HRMS (ESI): *m*/*z* calcd. for C_13_H_18_ClN_2_O_5_Si [M-H]^-^: 345.0679; found, 345.0674.

#### 5.1.16. 4-((Tert-Butyldimethylsilyloxy)methyl)-2,6-Dinitroaniline (**17**)

This compound was synthesized from **16** (640 mg 1.85 mmol) in analogy to **9**. Yield: 99%. ^1^H NMR (300 MHz, DMSO-*d*_6_) δ 8.45 (s, 1H), 8.37 (s, 1H), 7.33 (s, 2H), 3.35 (s, 2H), 0.92 (s, 5H), 0.10 (s, 3H). ^13^C NMR (75 MHz, DMSO-*d*_6_) δ 140.08, 134.79, 131.32, 127.05, 62.21, 25.88, 18.06, −5.18. HRMS (ESI): *m*/*z* calcd. for C_13_H_20_N_3_O_5_Si [M−H]^−^: 326.1178; found, 326.1185. 

#### 5.1.17. 5-((Tert-Butyldimethylsilyloxy)methyl)-3-Nitrobenzene-1,2-Diamine (**18**)

This compound was synthesized in analogy to **10** starting from 600 mg (1.83 mmol) of **17**. Yield: 80%. ^1^H NMR (300 MHz, DMSO-*d*_6_) δ 7.28 (d, *J* = 1.8 Hz, 1H), 6.94 (s, 2H), 6.74 (d, *J* = 1.9 Hz, 1H), 5.28 (s, 2H), 4.50 (s, 2H), 0.89 (s, 9H), 0.07 (s, 6H). ^13^C NMR (75 MHz, DMSO-*d*_6_) δ 137.91, 134.76, 130.57, 128.64, 115.80, 110.35, 64.04, 25.98, 18.15, −5.06. HRMS (ESI): *m*/*z* calcd. for C_13_H_24_N_3_O_3_Si [M + H]^+^: 298.1581; found, 298.1571.

#### 5.1.18. 6-((Tert-Butyldimethylsilyloxy)methyl)-4-Nitro-1H-Benzo[d]imidazole (**13**)

*Alternative procedure*: A mixture of **18** (410 mg, 1.38 mmol) and triethyl orthoformate (25 mL) was refluxed for about 3 h at 145 °C. After the completion of the reaction as monitored by TLC, 1.5 mL of formic acid was added, and the mixture was refluxed at the same temperature for another 2 h. The solution was evaporated to dryness at reduced pressure, and the residue was dissolved in methanol and stirred at rt, overnight, in the presence of charcoal. Following the removal of the charcoal by vacuum filtration through celite-545, the filtrate was evaporated to give 300 mg of title compound **13** as an off-white solid with a 85% yield without further purification [31]. ^1^H NMR (300 MHz, DMSO-*d*_6_) δ 13.28 (s, 1H), 8.45 (s, 1H), 8.11 (d, *J* = 19.8 Hz, 2H), 4.92 (s, 2H), 0.93 (s, 9H), 0.12 (s, 6H). ^13^C NMR (75 MHz, DMSO-*d*_6_) δ 146.40, 145.70, 135.20, 133.00, 126.52, 124.53, 116.90, 63.60, 25.93, 18.13, −5.12. HRMS (ESI): *m*/*z* calcd. for C_14_H_22_N_3_O_3_Si [M + H]^+^: 308.1425; found, 308.1415.

#### 5.1.19. (2R,3S,4S,5R)-2-(Acetoxymethyl)-5-(5-(((Tert-Butyldimethylsilyl)oxy)methyl)-7-Nitro-1H-Benzo[d]imidazol-1-yl)tetrahydrofuran-3,4-Diyl Diacetate (**19**) 

To a solution of compound **13** (280 mg, 0.9 mmol) in dry 1,2-dichlorethane, an amount of Bis(trimethylsilyl)acetamide (BSA) (366 mg, 1.8 mmol) was added, and the reaction was stirred at 60 °C for 40 min. Hereafter, 1,2,3,5-tetra-*O*-acetyl-D-ribofuranose (561 mg, 1.8 mM, dissolved in 8 mL of dry 1,2-dichlorethane), TMSOTf (300 mg, 1.35 mmol) and *N*-methylmorpholine (127 mg, 1.08 mmol) were added, and the reaction mixture was stirred at 65 °C for another 2.5 h. After completion, the reaction mixture was concentrated and portioned between EA and saturated NaHCO_3_. The residue was subjected to silica gel chromatography, and the title compound **19** was obtained with 330 mg (64% yield) [30]. 1H NMR (300 MHz, DMSO-*d*_6_) δ 8.78 (s, 1H), 8.05 (dd, *J* = 1.6, 0.7 Hz, 1H), 7.99 (d, *J* = 1.4 Hz, 1H), 6.46 (d, *J* = 3.5 Hz, 1H), 5.78 (dd, *J* = 5.6, 3.6 Hz, 1H), 5.41 (t, *J* = 6.0 Hz, 1H), 4.97–4.85 (m, 2H), 4.40 (ddd, *J* = 6.8, 4.6, 3.1 Hz, 1H), 4.34–4.14 (m, 2H), 2.12 (d, *J* = 0.6 Hz, 3H), 2.11–2.05 (m, 3H), 1.91 (d, *J* = 0.6 Hz, 3H), 0.92 (d, *J* = 0.6 Hz, 9H), 0.11 (d, *J* = 0.6 Hz, 6H). ^13^C NMR (75 MHz, DMSO-*d*_6_) δ 169.89, 169.44, 169.23, 147.29, 144.56, 136.30, 136.14, 123.73, 118.85, 88.50, 79.13, 73.83, 69.31, 63.25, 62.49, 20.40, 20.33, 20.30, 18.09, −5.21. HRMS (ESI): *m*/*z* calcd. for C_25_H_36_N_3_O_10_Si [M + H]+: 566.2164; found, 566.2168.

#### 5.1.20. (2R,3S,4S,5R)-2-(Hydroxymethyl)-5-(5-(((Tert-Butyldimethylsilyl)oxy)methyl)-7-Nitro-1H-Benzo[d]imidazol-1-yl)tetrahydrofuran-3,4-Diol (**20**)

A solution of compound **19** (327 mg, 0.58 mmol) in methanolic ammonia (7 N, 15 mL) was stirred at 0 °C for 4 h. The reaction was concentrated in the presence of silica gel, and the title compound **20** was purified by silica gel chromatography using EA/methanol (8:2) to afford 254 mg (90% yield). ^1^H NMR (300 MHz, DMSO-*d*_6_) δ 8.69 (s, 1H), 8.02 (d, *J* = 1.4 Hz, 1H), 7.91 (d, *J* = 1.4 Hz, 1H), 6.05 (d, *J* = 3.9 Hz, 1H), 5.70 (d, *J* = 5.8 Hz, 1H), 5.41 (d, *J* = 5.6 Hz, 1H), 4.90 (s, 2H), 4.56 (q, *J* = 4.8 Hz, 1H), 4.18–3.92 (m, 5H), 0.93 (s, 9H), 0.12 (s, 6H). ^13^C NMR (75 MHz, DMSO-*d*_6_) δ 147.17, 144.44, 136.49, 135.95, 123.91, 123.12, 118.36, 89.75, 81.59, 73.83, 70.09, 63.33, 63.12, 25.92, 18.12, −5.14. HRMS (ESI): *m*/*z* calcd. for C_21_H_32_N_3_O_8_Si [M + H]^+^: 482.1953; found, 482.1962.

#### 5.1.21. (1-(2. R,3S,4S,5R)-(6-(Hydroxymethyl)-2,2-Dimethyltetrahydrofuro[3,4-d][1,3]dioxol-4-yl)-7-Nitro-1H-Benzo[d]imidazol-5-yl)methanol (**21**)

To a solution of compound **20** (1.66 g, 3.77 mmol), suspended in dry acetone (500 mL), was added 37 mM dry *p*-toluenesulfonic acid (6.5 g, 10 eq.) in one portion. The mixture was stirred under a nitrogen atmosphere and turned into a yellow solution upon dissolution. After 3 h, a saturated NaHCO_3_ solution (500 mL) cooled to 0 °C was added with stirring over 5 min. The solvents were removed under reduced pressure, and the residue was portioned between ethyl acetate (200 mL) and water [32]. The solvent was evaporated, and the title compound **21** was purified by silica gel chromatography (EA/MeOH 9:1) with an 81% yield (1.08 g, 2.95 mM). ^1^H NMR (300 MHz, DMSO-*d*_6_) δ 8.76 (s, 1H), 8.00 (d, *J* = 1.4 Hz, 1H), 7.90 (d, *J* = 1.4 Hz, 1H), 6.26 (d, *J* = 1.8 Hz, 1H), 5.38 (dd, *J* = 5.9, 2.0 Hz, 1H), 4.83 (dd, *J* = 5.9, 2.2 Hz, 1H), 4.66 (s, 2H), 4.09 (ddd, *J* = 6.2, 4.2, 2.1 Hz, 1H), 3.08 (dd, *J* = 11.6, 4.3 Hz, 1H), 2.88 (dd, *J* = 11.6, 5.9 Hz, 1H), 1.49 (s, 3H), 1.32 (s, 3H). ^13^C NMR (75 MHz, DMSO-*d*_6_) δ 147.20, 144.82, 137.44, 136.55, 123.62, 123.41, 118.83, 112.64, 91.20, 86.81, 83.34, 81.11, 62.00, 60.72, 26.79, 25.19. HRMS (ESI): *m*/*z* calcd. for C_16_H_20_N_3_O_7_ [M + H]^+^: 366.1296; found, 366.1299.

#### 5.1.22. 5-(Benzyloxy)methyl-2-Chloro-1,3-Dinitrobenzene (**22**)

To a solution of compound **8** (500 mg, 2.59 mmol) in 20 mL of 1,4-dioxane was added benzyl 2,2,2-trichloroacetimidate (1.3 g, 5.2 mmol) and trifluoromethanesulfonic acid (200 mg, 1.3 mmol), and the reaction mixture was stirred at rt for 1 h. The reaction was portioned between EA and saturated NaHCO_3_; the organic layer was concentrated and subjected to a silica gel column. The title compound **22** was separated with a 95% yield (680 mg). ^1^H NMR (300 MHz, CDCl_3_) δ 8.59 (d, *J* = 0.9 Hz, 2H), 8.11–7.91 (m, 5H), 5.29 (s, 2H), 5.25 (d, *J* = 0.9 Hz, 2H). ^13^C NMR (75 MHz, CDCl_3_) δ 141.13, 136.99, 129.29, 129.00, 128.64, 128.46, 128.24, 128.04, 126.14, 119.09, 73.70, 69.25. HRMS (ESI): *m*/*z* calcd. for C_14_H_12_ClN_2_O_5_ [M + H]^+^: 323.0429; found, 323.0424.

#### 5.1.23. 4-(Benzyloxy)methyl-2,6-Dinitroaniline (**23**)

This compound was synthesized in analogy to **9** starting from 680 mg (2.11 mmol) of **22**. Yield: 96%. ^1^H NMR (300 MHz, DMSO-*d*_6_) δ 8.48 (s, 2H), 8.40 (s, 2H), 7.40–7.31 (m, 5H), 4.57 (s, 2H), 4.54 (s, 2H). ^13^C NMR (75 MHz, DMSO-*d*_6_) δ 140.33, 134.84, 132.99, 128.54, 128.45, 127.80, 127.71, 127.26, 127.16, 124.22, 71.75, 69.32. HRMS (ESI): *m*/*z* calcd. for C_14_H_14_N_3_O_5_ [M + H]^+^: 304.0928; found, 304.0930.

#### 5.1.24. 5-(Benzyloxy)methyl-3-Nitrobenzene-1,2-Diamine (**24**)

This compound was synthesized in analogy to **10** starting from 630 mg (2.08 mmol) of **23**. Yield: 80%. ^1^H NMR (300 MHz, DMSO-*d*_6_) δ = 7.50–7.16 (m, 6H), 7.01 (s, 2H), 6.81 (d, *J* = 1.8, 1H), 5.34 (s, 2H), 4.47 (s, 2H), 4.34 (s, 2H). HRMS (ESI): *m*/*z* calcd. for C_14_H_16_N_3_O_3_ [M + H]^+^: 274.1186; found, 274.1188.

#### 5.1.25. 6-(Benzyloxy)methyl-4-Nitro-1H-benzo[d]imidazole (**25**)

This compound was synthesized in analogy to **13** (which was synthesized from **18**) starting from 550 mg (2.0 mmol) of **24**. Yield: 84%. ^1^H NMR (300 MHz, DMSO-*d*_6_) δ 13.31 (s, 1H), 8.47 (s, 1H), 8.16 (s, 2H), 7.53–7.09 (m, 5H), 4.75 (s, 2H), 4.60 (s, 2H). ^13^C NMR (75 MHz, DMSO-*d*_6_) δ 145.81, 138.35, 132.31, 128.45, 127.72, 127.66, 126.23, 118.39, 71.69, 70.76. HRMS (ESI): *m*/*z* calcd. For C_15_H_14_N_3_O_3_ [M + H]^+^: 284.1030; found, 284.1021.

#### 5.1.26. (2R,3S,4S,5R)-2-(Acetoxymethyl)-5-(5-((Benzyloxy)methyl)-7-Nitro-1H-Benzo[d]imidazol-1-yl)tetrahydrofuran-3,4-Diyl Diacetate (**26**)

This compound was synthesized in analogy to **19** starting from 1.44 g (5.09 mmol) of **25**. Yield: 64%. ^1^H NMR (300 MHz, DMSO-*d*_6_) δ 8.80 (s, 1H), 8.13 (d, *J* = 1.4 Hz, 1H), 8.03 (d, *J* = 1.4 Hz, 1H), 7.50–7.22 (m, 5H), 6.46 (d, *J* = 3.6 Hz, 1H), 5.79 (dd, *J* = 5.6, 3.6 Hz, 1H), 5.40 (t, *J* = 6.0 Hz, 1H), 4.74 (s, 2H), 4.60 (s, 2H), 4.40 (dq, *J* = 6.4, 3.1 Hz, 1H), 4.34–4.15 (m, 2H), 2.12 (s, 3H), 2.08 (s, 3H), 1.91 (s, 3H). ^13^C NMR (75 MHz, DMSO-*d*_6_) δ 169.93, 169.46, 169.26, 147.25, 144.66, 138.29, 136.24, 133.42, 128.45, 127.71, 127.67, 125.42, 124.06, 120.28, 88.52, 79.14, 73.81, 71.75, 70.33, 69.32, 62.49, 20.43, 20.37, 20.33. HRMS (ESI): *m*/*z* calcd. For C_26_H_27_N_3_O_10_Na [M + Na]^+^: 564.1589; found, 564.1607.

#### 5.1.27. (2R,3S,4S,5R)-2-(5-((Benzyloxy)methyl)-7-Nitro-1H-Benzo[d]imidazol-1-yl)-5-(Hydroxymethyl)tetrahydrofuran-3,4-Diol (**27**)

This compound was synthesized in analogy to **20** starting from 1.55 g (2.86 mmol) of **26**. Yield: 66%. ^1^H NMR (300 MHz, DMSO-*d*_6_) δ 8.86 (s, 1H), 8.10 (d, *J* = 1.4 Hz, 1H), 7.98 (d, *J* = 1.4 Hz, 1H), 7.47–7.25 (m, 5H), 6.08 (d, *J* = 5.0 Hz, 1H), 5.51 (d, *J* = 6.2 Hz, 1H), 5.23 (d, *J* = 5.0 Hz, 1H), 5.03 (t, *J* = 5.2 Hz, 1H), 4.73 (s, 2H), 4.60 (s, 2H), 4.29 (q, *J* = 5.3 Hz, 1H), 4.15–3.98 (m, 1H), 3.95 (d, *J* = 3.8 Hz, 1H), 3.70–3.42 (m, 2H). ^13^C NMR (75 MHz, DMSO-*d*_6_) δ 147.33, 144.92, 138.33, 136.24, 132.83, 128.46, 127.71, 127.67, 125.01, 124.36, 119.82, 89.52, 85.34, 75.19, 71.73, 70.45, 69.96, 60.71. HRMS (ESI): *m*/*z* calcd. For C_20_H_22_N_3_O_7_[M + H]^+^: 416.1452; found, 416.1447.

#### 5.1.28. (2R,3S,4S,5R)-(6-(5-((Benzyloxy)methyl)-7-Nitro-1H-Benzo[d]imidazol-1-yl)-2,2-Dimethyltetrahydrofuro[3,4-d][1,3]dioxol-4-yl)Methanol (**28**)

This compound was synthesized in analogy to **21** starting from 680 mg (1.64 mmol) of **27**. Yield: 78%. ^1^H NMR (300 MHz, CDCl_3_) δ 9.27 (s, 1H), 8.50 (dd, *J* = 10.1, 1.5 Hz, 2H), 8.11–7.85 (m, 5H), 7.09 (d, *J* = 3.2 Hz, 1H), 5.59 (dd, *J* = 6.1, 2.6 Hz, 1H), 5.50 (dd, *J* = 6.1, 3.2 Hz, 1H), 5.23 (d, *J* = 7.5 Hz, 4H), 5.06 (t, *J* = 2.5 Hz, 1H), 4.55 (dd, *J* = 12.0, 2.5 Hz, 1H), 4.43 (dd, *J* = 12.1, 2.7 Hz, 1H), 2.27 (s, 3H), 1.99 (s, 3H). ^13^C NMR (75 MHz, CDCl_3_) δ 146.93, 144.57, 137.92, 136.84, 133.52, 128.80, 128.19, 128.13, 125.05, 124.30, 120.77, 114.75, 92.94, 86.61, 85.55, 81.21, 72.91, 71.03, 62.12, 27.45, 25.73. HRMS (ESI): *m*/*z* calcd. For C_23_H_26_N_3_O_7_[M + H]^+^: 456.1765; found, 456.1759.

#### 5.1.29. (2R,3S,4S,5R)-(6-(5-((Benzyloxy)methyl)-7-Nitro-1H-Benzo[d]imidazol-1-yl)-2,2-Dimethyltetrahydrofuro[3,4-d][1,3]dioxol-4-yl)methyl Sulfamate (**29**)

Chlorosulfonyl isocyanate (630 mg, 4.5 mmol) was taken into a 10 mL flask, and after cooling to 0 °C, formic acid (210 mg, 4.5 mmol) was added and the mixture was stirred for 5 min. The resulting solid was dissolved in dry acetonitrile (6 mL), and the solution was cooled to 0 °C and stirred for another 5 h, gradually reaching rt. Compound **28** (580 mg, 1.27 mmol) was dissolved in 30 mL of dimethylacetamide (DMA) and cooled to 0 °C, followed by adding the obtained sulfamoyl chloride, and the mixture was stirred overnight. The next morning, 3 mL of TEA was added, and the reaction was stirred for 10 min, followed by adding 6 mL of methanol and further stirring for 15 min. The reaction was concentrated, and the residue was partitioned between EA and saturated NaHCO_3_. The organic layer was further washed with water and brine, and the title compound **29** was purified by silica gel chromatography with a 84% yield (570 mg). ^1^H NMR (300 MHz, DMSO-*d*_6_) δ 8.75 (s, 1H), 8.19–8.07 (m, 1H), 8.01 (d, *J* = 1.4 Hz, 1H), 7.56 (s, 2H), 7.47–7.25 (m, 5H), 6.39 (d, *J* = 1.9 Hz, 1H), 5.48 (dd, *J* = 6.0, 2.0 Hz, 1H), 4.94 (dd, *J* = 6.0, 3.0 Hz, 1H), 4.74 (s, 2H), 4.60 (s, 2H), 4.36 (m, 1H), 3.75 (m, 2H), 1.55 (s, 3H), 1.37 (s, 3H). ^13^C NMR (75 MHz, DMSO-*d*_6_) δ 147.16, 144.73, 138.28, 136.50, 133.41, 128.46, 127.75, 127.69, 125.12, 123.80, 120.12, 113.45, 90.92, 83.36, 83.32, 80.50, 71.76, 70.37, 67.44, 26.83, 25.27. HRMS (ESI): *m*/*z* calcd. for C_23_H_27_N_4_O_9_S [M + H]^+^: 535.1493; found, 535.1503.

#### 5.1.30. (2R,3S,4S,5R)-(6-(5-((Benzyloxy)methyl)-7-Nitro-1H-Benzo[d]imidazol-1-yl)-2,2-Dimethyltetrahydrofuro[3,4-d][1,3]dioxol-4-yl)methyl ((Tert-Butoxycarbonyl)glycyl)sulfamate (**30a**)

Compound **29** (140 mg, 0.26 mM) and Boc-Gly-OSu (140 mg, 0.52 mmol) were dissolved in 16 mL of dry DMF, followed by adding DBU (60 mg, 0.39 mmol), and the reaction was stirred at rt overnight. The reaction was concentrated under vacuum, and the residue was dissolved in EA and washed with saturated NaHCO_3_. The title compound **30a** was purified by silica gel chromatography with a 77% yield (EA:hexane, 2:1). ^1^H NMR (300 MHz, DMSO-*d*_6_) δ 8.81 (s, 1H), 8.10 (d, *J* = 1.5 Hz, 1H), 7.98 (d, *J* = 1.4 Hz, 1H), 7.51–7.19 (m, 6H), 6.33 (d, *J* = 2.1 Hz, 1H), 5.41 (dd, *J* = 5.9, 2.2 Hz, 1H), 4.95 (dd, *J* = 5.9, 2.3 Hz, 1H), 4.74 (s, 2H), 4.60 (d, *J* = 3.1 Hz, 2H), 4.31 (s, 1H), 3.80–3.59 (m, 1H), 3.40 (d, *J* = 5.8 Hz, 1H), 1.53 (s, 3H), 1.46–1.28 (m, 12H). ^13^C NMR (75 MHz, DMSO-*d*_6_) δ 173.55, 155.61, 147.20, 144.95, 138.31, 136.46, 133.31, 128.44, 127.74, 127.65, 125.00, 123.75, 119.92, 112.94, 91.40, 83.83, 83.41, 81.15, 77.67, 71.75, 70.39, 66.17, 45.63, 28.38, 26.86, 25.29. HRMS (ESI): *m*/*z* calcd. for C_30_H_36_N_5_O_12_S [M−H]^−^: 690.2086; found, 690.2086.

#### 5.1.31. (2R,3S,4S,5R)-(6-(5-((Benzyloxy)methyl)-7-Nitro-1H-Benzo[d]imidazol-1-yl)-2,2-Dimethyltetrahydrofuro[3,4-d][1,3]dioxol-4-yl)methyl (3-(4-(benzyloxy)phenyl)-2-((Tert-Butoxycarbonyl)amino)propanoyl)sulfamate (**30b**)

This compound was synthesized in analogy to **30a** using Boc-Tyr(OBn)-OSu, using 300 mg (0.56 mmol) of starting compound **29**. Yield: 99%. ^1^H NMR (300 MHz, DMSO-*d*_6_) δ 8.80 (s, 1H), 8.07 (d, *J* = 1.4 Hz, 1H), 7.97 (d, *J* = 1.4 Hz, 1H), 7.50–7.23 (m, 10H), 6.98 (dd, *J* = 67.8, 8.1 Hz, 5H), 6.35 (d, *J* = 2.2 Hz, 1H), 6.32–6.06 (m, 1H), 5.47–5.31 (m, 1H), 5.12–4.87 (m, 4H), 4.70 (s, 2H), 4.58 (s, 2H), 4.34 (dt, *J* = 6.9, 3.5 Hz, 1H), 3.76 (m, 3H), 2.91 (d, *J* = 13.7 Hz, 1H), 2.69 (dd, *J* = 13.6, 8.5 Hz, 1H), 1.53 (s, 3H), 1.32 (d, *J* = 10.5 Hz, 12H). ^13^C NMR (75 MHz, DMSO-*d*_6_) δ 156.89, 155.01, 147.22, 144.93, 138.30, 137.41, 136.41, 133.29, 130.43, 128.50, 128.43, 128.40, 127.83, 127.72, 127.69, 127.64, 125.05, 123.79, 119.95, 114.30, 113.12, 91.32, 83.59, 80.95, 77.78, 71.73, 70.37, 69.27, 57.55, 28.32, 26.89, 25.29. HRMS (ESI): *m*/*z* calcd. for C_44_H_48_N_5_O_13_S [M−H]^−^: 886.2975; found, 886.3018.

#### 5.1.32. (2R,3S,4S,5R)-(6-(5-((Benzyloxy)methyl)-7-Nitro-1H-Benzo[d]imidazol-1-yl)-2,2-Dimethyltetrahydrofuro[3,4-d][1,3]dioxol-4-yl)methyl (O-Benzyl-N-(Tert-Butoxycarbonyl)seryl)sulfamate (**30c**)

This compound was synthesized in analogy to **30a** using the appropriate protected serine, using 347 mg (0.65 mmol) of starting compound **29**. Yield: 95%. ^1^H NMR (300 MHz, DMSO-*d*_6_) δ 8.79 (s, 1H), 8.09 (d, *J* = 1.4 Hz, 1H), 7.98 (d, *J* = 1.4 Hz, 1H), 7.45–7.18 (m, 10H), 6.33 (d, *J* = 2.3 Hz, 1H), 6.10 (d, *J* = 8.1 Hz, 1H), 5.32 (dd, *J* = 6.1, 2.4 Hz, 1H), 4.92 (dd, *J* = 5.9, 2.3 Hz, 1H), 4.72 (s, 2H), 4.59 (s, 2H), 4.42 (d, *J* = 2.1 Hz, 2H), 4.30 (d, *J* = 6.1 Hz, 1H), 3.94 (s, 2H), 3.83–3.46 (m, 4H), 1.52 (s, 3H), 1.35 (d, *J* = 11.5 Hz, 12H). ^13^C NMR (75 MHz, DMSO-*d*_6_) δ 173.90, 155.00, 147.22, 145.00, 138.72, 138.30, 136.37, 133.27, 128.44, 128.18, 127.81, 127.73, 127.65, 127.48, 127.31, 125.05, 123.78, 119.94, 113.01, 91.38, 83.74, 83.61, 81.09, 77.78, 71.93, 71.73, 71.24, 70.37, 66.32, 56.62, 28.35, 26.91, 25.29. HRMS (ESI): *m*/*z* calcd. for C_38_H_44_N_5_O_13_S [M−H]^−^: 810.2662; found, 810.2668.

#### 5.1.33. Tert-Butyl 4-((((6-(5-((Benzyloxy)methyl)-7-Nitro-1H-Benzo[d]imidazol-1-yl)-2,2-Dimethyltetrahydrofuro[3,4-d][1,3]dioxol-4-yl)methoxy)sulfonyl)amino)-3-((Tert-Butoxycarbonyl)amino)-4-oxobutanoate (**30d**)

This compound was synthesized in analogy to **30a** with the aid of the appropriately protected aspartic acid, using 300 mg (0.56 mmol) of starting compound **29**. Yield: 99%. ^1^H NMR (300 MHz, DMSO-*d*_6_) δ 8.77 (s, 1H), 8.10 (d, *J* = 1.4 Hz, 1H), 7.98 (d, *J* = 1.4 Hz, 1H), 7.48–7.22 (m, 5H), 6.34 (d, *J* = 2.3 Hz, 1H), 5.37 (dd, *J* = 6.2, 2.3 Hz, 1H), 4.93 (dd, *J* = 5.9, 2.4 Hz, 1H), 4.74 (s, 2H), 4.60 (s, 2H), 4.41–4.26 (m, 1H), 4.19–4.04 (m, 2H), 3.78 (dd, *J* = 10.9, 4.3 Hz, 1H), 3.63 (s, 0H), 3.18 (s, 2H), 2.70–2.54 (m, 1H), 2.35 (dd, *J* = 15.3, 8.4 Hz, 1H), 1.53 (s, 3H), 1.46–1.27 (m, 21H). ^13^C NMR (75 MHz, DMSO-*d*_6_) δ 170.02, 154.96, 147.20, 144.95, 138.30, 136.40, 133.28, 128.43, 127.72, 127.65, 125.05, 123.78, 119.97, 113.08, 91.33, 83.62, 83.53, 81.00, 79.73, 77.86, 71.73, 70.37, 48.73, 29.72, 28.31, 27.81, 27.78, 26.89, 25.28. HRMS (ESI): *m*/*z* calcd. for C_36_H_46_N_5_O_14_S [M−H]^−^: 804.2767; found, 804.2786.

#### 5.1.34. (2R,3S,4S,5R)-(6-(5-((Benzyloxy)methyl)-7-Nitro-1H-Benzo[d]imidazol-1-yl)-2,2-Dimethyltetrahydrofuro[3,4-d][1,3]dioxol-4-yl)methyl ((Tert-Butoxycarbonyl)leucyl)sulfamate (**30e**)

This compound was synthesized in analogy to **30a** using Boc-Leu-OSu, using 220 mg (0.41 mmol) of starting compound **29**. Yield: 72%. ^1^H NMR (300 MHz, DMSO-*d*_6_) δ 8.80 (s, 1H), 8.10 (d, *J* = 1.4 Hz, 1H), 7.98 (d, *J* = 1.4 Hz, 1H), 7.47–7.22 (m, 5H), 6.32 (d, *J* = 2.3 Hz, 1H), 6.05 (d, *J* = 8.6 Hz, 1H), 5.36 (d, *J* = 5.6 Hz, 1H), 4.93 (dd, *J* = 6.0, 2.2 Hz, 1H), 4.74 (s, 2H), 4.60 (s, 2H), 4.33 (s, 1H), 3.82–3.63 (m, 2H), 3.51 (dd, *J* = 11.0, 5.7 Hz, 1H), 1.67–1.23 (m, 18H), 0.83 (dd, *J* = 6.5, 1.5 Hz, 6H). ^13^C NMR (75 MHz, DMSO-*d*_6_) δ 177.14, 155.13, 147.20, 145.00, 138.30, 136.39, 133.26, 128.44, 127.72, 127.65, 125.01, 123.77, 119.92, 112.97, 91.36, 83.79, 83.58, 81.16, 77.47, 71.74, 70.37, 66.21, 54.90, 42.59, 28.36, 26.91, 25.31, 24.54, 23.29, 21.99. HRMS (ESI): *m*/*z* calcd. for C_34_H_44_N_5_O_12_S [M−H]^−^: 746.2712; found, 746.2708.

#### 5.1.35. (2R,3S,4S,5R)-(6-(5-((Benzyloxy)methyl)-7-Nitro-1H-benzo[d]imidazol-1-yl)-2,2-Dimethyltetrahydrofuro[3,4-d][1,3]dioxol-4-yl)methyl ((Tert-Butoxycarbonyl)isoleucyl)sulfamate (**30f**)

This compound was synthesized in analogy to **30a** using Boc-Ile-OSu, using 140 mg (0.26 mmol) of starting compound **29**. Yield: 82%. ^1^H NMR (300 MHz, DMSO-*d*_6_) δ 8.80 (s, 1H), 8.10 (d, *J* = 1.5 Hz, 1H), 7.98 (d, *J* = 1.4 Hz, 1H), 7.51–7.10 (m, 4H), 6.33 (d, *J* = 2.2 Hz, 1H), 5.80 (s, 1H), 5.38 (d, *J* = 5.8 Hz, 1H), 4.93 (dd, *J* = 5.8, 2.4 Hz, 1H), 4.74 (s, 2H), 4.60 (d, *J* = 2.8 Hz, 2H), 4.32 (s, 1H), 3.85–3.40 (m, 2H), 2.81 (s, 1H), 1.53 (s, 3H), 1.40 (s, 3H), 1.35 (d, *J* = 4.9 Hz, 12H), 0.92–0.68 (m, 6H). ^13^C NMR (75 MHz, DMSO-*d*_6_) δ 170.11, 155.15, 147.21, 144.93, 138.31, 136.41, 133.28, 128.44, 127.72, 127.65, 125.03, 123.75, 119.93, 113.01, 91.27, 83.71, 83.52, 81.08, 78.85, 77.71, 71.73, 70.36, 60.37, 28.30, 26.87, 25.62, 25.25, 24.46, 15.74, 11.71. HRMS (ESI): *m*/*z* calcd. for C_34_H_44_N_5_O_12_S [M−H]^−^: 746.2712; found, 746.2712.

#### 5.1.36. (2R,3S,4S,5R)-(6-(7-Amino-5-Methyl-1H-benzo[d]imidazol-1-yl)-2,2-Dimethyltetrahydrofuro[3,4-d][1,3]dioxol-4-yl)methyl ((Tert-Butoxycarbonyl)glycyl)sulfamate (**31a**)

To a solution of compound **30a** (140 mg, 0.187 mmol) in methanol (20 mL) was added 69 mg of Pd/C under argon, after which the argon was exchanged for hydrogen and the mixture was stirred at rt overnight. TLC analysis indicated the reaction to be completed, the Pd/C was filtered, and the filtrate was concentrated. The residue was adsorbed on silica, and title compound **31a** was purified by chromatography (MeOH/EA, 1:9) with an 84% yield (95 mg). ^1^H NMR (300 MHz, CD_3_OD) δ = 8.31 (s, 1H), 6.89 (dd, *J* = 1.5, 0.9 Hz, 1H), 6.59 (s, 1H), 6.20 (d, *J* = 4.2 Hz, 1H), 5.30–5.05 (m, 2H), 4.47 (t, *J* = 3.1 Hz, 1H), 4.28 (dd, *J* = 5.5, 3.2 Hz, 2H), 3.69 (s, 2H), 3.37 (s, 1H), 3.33 (p, *J* = 1.6 Hz, 2H), 2.36 (t, *J* = 0.6 Hz, 3H), 1.61 (s, 3H), 1.44 (d, *J* = 1.7 Hz, 10H), 1.39 (s, 3H). ^13^C NMR (75 MHz, CD_3_OD) δ 176.21, 156.48, 144.88, 141.33, 133.48, 133.31, 120.29, 114.92, 111.86, 108.59, 91.51, 83.37, 82.20, 79.86, 78.40, 66.53, 45.02, 27.05, 25.59, 23.81, 19.84. HRMS (ESI): *m*/*z* calcd. for C_23_H_32_N_5_O_9_S [M−H]^−^: 554.1926; found, 554.1924.

#### 5.1.37. (2R,3S,4S,5R)-(6-(7-Amino-5-(Hydroxymethyl)-1H-Benzo[d]imidazol-1-yl)-2,2-Dimethyltetrahydrofuro[3,4-d][1,3]dioxol-4-yl)methyl ((Tert-Butoxycarbonyl)tyrosyl)sulfamate (**31b**)

This compound was synthesized in analogy to **31a** starting from 400 mg (0.62 mmol) of **30b**. Yield: 98%. ^1^H NMR (300 MHz, DMSO-*d*_6_) δ 9.07 (d, *J* = 2.0 Hz, 1H), 8.31 (d, *J* = 2.0 Hz, 1H), 7.07–6.86 (m, 3H), 6.61 (dd, *J* = 8.5, 2.1 Hz, 3H), 6.24 (s, 1H), 6.04 (d, *J* = 8.2 Hz, 1H), 5.17 (s, 1H), 5.10–4.80 (m, 4H), 4.58–4.42 (m, 2H), 4.35 (s, 1H), 4.04–3.75 (m, 3H), 2.93 (d, *J* = 13.7 Hz, 1H), 2.81–2.60 (m, 1H), 1.55 (d, *J* = 2.0 Hz, 3H), 1.33 (d, *J* = 2.0 Hz, 12H). ^13^C NMR (75 MHz, DMSO-*d*_6_) δ 175.99, 155.57, 154.98, 145.87, 142.25, 137.98, 134.27, 130.30, 128.99, 121.60, 114.77, 114.32, 109.18, 107.24, 90.37, 83.38, 82.36, 80.25, 77.56, 66.24, 63.55, 57.94, 37.30, 28.37, 27.01, 25.33. HRMS (ESI): *m*/*z* calcd. for C_30_H_38_N_5_O_11_S [M−H]^−^: 676.2294; found, 676.2291.

#### 5.1.38. (2R,3S,4S,5R)-(6-(7-Amino-5-(Hydroxymethyl)-1H-Benzo[d]imidazol-1-yl)-2,2-Dimethyltetrahydrofuro[3,4-d][1,3]dioxol-4-yl)methyl ((Tert-Butoxycarbonyl)seryl)sulfamate (**31c**)

This compound was synthesized in analogy to **31a** starting from 500 mg (0.62 mmol) of **30c**. Yield: 80%. ^1^H NMR (300 MHz, DMSO-*d*_6_) δ 8.30 (s, 1H), 6.93 (d, *J* = 1.3 Hz, 1H), 6.61 (d, *J* = 1.3 Hz, 1H), 6.23 (d, *J* = 3.9 Hz, 1H), 6.05 (d, *J* = 7.9 Hz, 1H), 5.76 (s, 1H), 5.25–5.08 (m, 1H), 5.08–4.96 (m, 2H), 4.80 (d, 3H), 4.46 (d, *J* = 5.4 Hz, 2H), 4.34 (q, *J* = 4.0 Hz, 1H), 4.01–3.84 (m, 1H), 3.82–3.67 (m, 1H), 3.58 (d, *J* = 4.4 Hz, 2H), 2.60 (s, 1H), 1.55 (s, 3H), 1.35 (d, *J* = 17.8 Hz, 12H). ^13^C NMR (75 MHz, DMSO-*d*_6_) δ 174.73, 155.13, 145.87, 142.28, 137.97, 134.29, 121.60, 114.28, 109.15, 107.22, 90.40, 83.36, 82.33, 80.26, 77.80, 66.04, 63.55, 62.94, 58.57, 28.38, 27.01, 25.33. HRMS (ESI): *m*/*z* calcd. for C_24_H_34_N_5_O_11_S [M−H]^−^: 600.1981; found, 600.1983.

#### 5.1.39. Tert-Butyl 4-((((6-(7-Amino-5-(Hydroxymethyl)-1H-Benzo[d]imidazol-1-yl)-2,2-Dimethyltetrahydrofuro[3,4-d][1,3]dioxol-4-yl)methoxy)sulfonyl)amino)-3-((Tert-Butoxycarbonyl)amino)-4-Oxobutanoate (**31d**)

This compound was synthesized in analogy to **31a** starting from 460 mg (0.57 mmol) of **30d**. Yield: 68%. ^1^H NMR (300 MHz, DMSO-*d*_6_) δ 8.31 (s, 1H), 6.93 (s, 1H), 6.62 (s, 1H), 6.33 (d, *J* = 8.5 Hz, 1H), 6.23 (d, *J* = 4.0 Hz, 1H), 5.17 (dd, *J* = 6.6, 4.0 Hz, 1H), 5.00 (dd, *J* = 8.6, 5.6 Hz, 3H), 4.46 (d, *J* = 5.0 Hz, 2H), 4.34 (d, *J* = 3.8 Hz, 1H), 4.06 (d, *J* = 7.1 Hz, 1H), 3.91 (dd, *J* = 11.1, 4.6 Hz, 1H), 2.64 (dd, *J* = 15.0, 4.7 Hz, 1H), 2.36 (dd, *J* = 15.1, 8.5 Hz, 1H), 1.55 (s, 3H), 1.52–1.27 (m, 21H). ^13^C NMR (75 MHz, DMSO-*d*_6_) δ 175.05, 155.03, 145.72, 142.18, 138.03, 134.30, 121.58, 114.27, 109.19, 107.17, 99.65, 90.41, 83.35, 82.41, 80.30, 79.61, 77.72, 66.19, 63.53, 53.56, 28.37, 27.85, 27.01, 25.31. HRMS (ESI): *m*/*z* calcd for C_29_H_42_N_5_O_12_S [M−H]^−^: 684.2556; found, 684.2569.

#### 5.1.40. (2R,3S,4S,5R)-(6-(7-Amino-5-(Hydroxymethyl)-1H-Benzo[d]imidazol-1-yl)-2,2-Dimethyltetrahydrofuro[3,4-d][1,3]dioxol-4-yl)methyl ((Tert-Butoxycarbonyl)leucyl)sulfamate (**31e**)

This compound was synthesized in analogy to **31a** starting from 200 mg (0.27 mmol) of **30e**. Yield: 80%. ^1^H NMR (300 MHz, DMSO-*d*_6_) δ 11.97 (s, 1H), 8.30 (s, 1H), 6.92 (d, *J* = 1.3 Hz, 1H), 6.61 (s, 1H), 6.22 (d, *J* = 3.9 Hz, 1H), 6.14 (d, *J* = 8.5 Hz, 1H), 5.16 (dd, *J* = 6.7, 4.0 Hz, 1H), 5.10–4.75 (m, 2H), 4.46 (d, *J* = 5.6 Hz, 2H), 4.39–4.24 (m, 1H), 4.02–3.84 (m, 2H), 3.76 (d, *J* = 5.1 Hz, 1H), 3.18 (d, *J* = 4.4 Hz, 1H), 1.91 (s, 2H), 1.55 (s, 3H), 1.34 (d, *J* = 12.9 Hz, 12H), 0.85 (dd, *J* = 6.5, 1.5 Hz, 8H). ^13^C NMR (75 MHz, DMSO-*d*_6_) δ 177.25, 155.23, 145.89, 142.22, 137.95, 134.26, 121.61, 114.25, 109.14, 107.25, 90.35, 83.35, 82.37, 80.28, 77.50, 66.12, 63.54, 55.03, 42.50, 28.39, 27.01, 25.34, 24.56, 23.33, 21.96. HRMS (ESI): *m*/*z* calcd. for C_27_H_40_N_5_O_10_S [M + H]^+^: 628.2647; found, 628.2656.

#### 5.1.41. (2R,3S,4S,5R)-(6-(7-Amino-5-(Hydroxymethyl)-1H-Benzo[d]imidazol-1-yl)-2,2-Dimethyltetrahydrofuro[3,4-d][1,3]dioxol-4-yl)methyl ((Tert-Butoxycarbonyl)isoleucyl)sulfamate (**31f**)

This compound was synthesized in analogy to **31a** starting from 140 mg (0.176 mmol) of **30f**. Yield: 85%. ^1^H NMR (300 MHz, DMSO-*d*_6_) δ 8.31 (s, 1H), 6.93 (s, 1H), 6.62 (d, *J* = 1.3 Hz, 1H), 6.23 (d, *J* = 3.9 Hz, 1H), 5.87 (d, *J* = 8.4 Hz, 1H), 5.09 (dd, *J* = 6.7, 3.7 Hz, 3H), 4.46 (d, *J* = 4.8 Hz, 2H), 4.36 (t, *J* = 3.9 Hz, 1H), 3.96 (dd, *J* = 12.1, 7.4 Hz, 1H), 3.67 (dd, *J* = 8.5, 5.0 Hz, 1H), 1.72 (s, 1H), 1.55 (s, 3H), 1.35 (d, *J* = 15.0 Hz, 12H), 1.05 (dt, *J* = 15.3, 7.8 Hz, 1H), 0.79 (dt, *J* = 7.5, 4.0 Hz, 7H). ^13^C NMR (75 MHz, DMSO-*d*_6_) δ 175.79, 155.19, 145.86, 142.17, 137.96, 134.25, 121.61, 114.28, 109.16, 107.25, 99.66, 90.34, 83.37, 82.35, 80.27, 77.71, 66.33, 63.54, 60.58, 59.88, 38.14, 28.33, 26.98, 25.29, 24.45, 15.84, 11.78. HRMS (ESI): *m*/*z* calcd. for C_27_H_40_N_5_O_10_S [M−H]^−^: 626.2501; found, 626.2496.

#### 5.1.42. (2R,3S,4S,5R)-(5-(7-Amino-5-Methyl-1H-Benzo[d]imidazol-1-yl)-3,4-Dihydroxytetrahydrofuran-2-yl)methyl Glycylsulfamate (**32a**)

Compound **31a** (60 mg, 0.11 mmol) was dissolved in a mixture of TFA and H_2_O (7 mL, 5:2 *v/v*), and the reaction mixture was stirred for 40 min. After reaction, the volatiles were evaporated under reduced pressure, followed by co-evaporation with EtOH, and once more with EtOH + 1 mL Et_3_N to neutralize any remaining acid. The title compound **32a** was obtained by reversed phase-HPLC as a white solid with a 60% yield. ^1^H NMR (300 MHz, D_2_O) δ 8.20 (s, 1H), 6.87 (s, 1H), 6.50 (s, 1H), 6.00 (d, *J* = 6.0 Hz, 1H), 4.53 (t, *J* = 5.8 Hz, 1H), 4.32 (m, 5H), 3.54 (s, 2H), 2.22 (s, 3H). ^13^C NMR (75 MHz, D_2_O) δ 172.34, 143.71, 141.14, 134.52, 131.91, 120.76, 113.15, 109.54, 88.63, 81.94, 73.22, 69.30, 67.80, 42.43, 20.03. HRMS (ESI): *m*/*z* calcd. for C_15_H_22_N_5_O_7_S [M + H]^+^: 416.1234; found, 416.1223.

#### 5.1.43. (2R,3S,4S,5R)-(5-(7-Amino-5-(Hydroxymethyl)-1H-Benzo[d]imidazol-1-yl)-3,4-Dihydroxytetrahydrofuran-2-yl)methyl Tyrosylsulfamate (**32b**)

This compound was synthesized in analogy to **32a** starting from 300 mg (0.44 mmol) of **31b**. Yield: 80%. ^1^H NMR (300 MHz, D_2_O) δ 8.67 (s, 1H), 7.09–6.82 (m, 3H), 6.81–6.50 (m, 3H), 6.18 (d, *J* = 2.9 Hz, 1H), 4.61–4.08 (m, 7H), 3.84 (t, *J* = 6.6 Hz, 1H), 2.86 (qd, *J* = 14.4, 6.0 Hz, 2H). ^13^C NMR (75 MHz, D_2_O) δ 174.41, 154.49, 139.14, 138.81, 137.11, 133.45, 130.41, 125.58, 120.65, 115.21, 112.16, 105.07, 89.76, 82.61, 74.07, 69.49, 67.68, 63.14, 56.24, 35.47. HRMS (ESI): *m*/*z* calcd. for C_22_H_26_N_5_O_9_S [M−H]^−^: 536.1457; found, 536.1452.

#### 5.1.44. (2R,3S,4S,5R)-(5-(7-Amino-5-(Hydroxymethyl)-1H-Benzo[d]imidazol-1-yl)-3,4-Dihydroxytetrahydrofuran-2-yl)methyl Serylsulfamate (**32c**)

This compound was synthesized in analogy to **32a** starting from 115 mg (0.19 mmol) of **31c**. Yield: 62%. ^1^H NMR (300 MHz, D_2_O) δ 8.47 (s, 1H), 6.98 (d, *J* = 1.4 Hz, 1H), 6.65 (d, *J* = 1.4 Hz, 1H), 6.05 (dd, *J* = 5.8, 1.3 Hz, 1H), 4.52 (d, *J* = 8.4 Hz, 3H), 4.42–4.17 (m, 4H), 3.99–3.69 (m, 3H). ^13^C NMR (75 MHz, D_2_O) δ 172.83, 140.46, 140.26, 137.76, 132.93, 121.36, 111.56, 106.78, 89.12, 82.33, 73.59, 69.39, 67.80, 63.35, 59.94, 56.67. HRMS (ESI): *m*/*z* calcd. for C_16_H_22_N_5_O_9_S [M−H]^−^: 460.1144; found, 460.1146.

#### 5.1.45. 3-Amino-4-((((5-(7-Amino-5-(Hydroxymethyl)-1H-Benzo[d]imidazol-1-yl)-3,4-Dihydroxytetrahydrofuran-2-yl)methoxy)sulfonyl)amino)-4-Oxobutanoic Acid (**32d**)

This compound was synthesized in analogy to **32a** starting from 120 mg (0.18 mmol) of **31d**. Yield: 48%. ^1^H NMR (300 MHz, D_2_O) δ 9.19 (s, 1H), 7.13 (s, 1H), 6.89 (s, 1H), 6.33 (d, *J* = 4.9 Hz, 1H), 4.70–4.20 (m, 7H), 3.98 (t, *J* = 5.3 Hz, 1H), 2.90 (d, *J* = 5.4 Hz, 2H). ^13^C NMR (75 MHz, D_2_O) δ 173.71, 173.43, 140.87, 137.35, 134.06, 133.04, 119.85, 117.95, 114.08, 112.91, 103.02, 90.54, 83.20, 74.53, 69.74, 67.62, 62.90, 51.41, 34.36. HRMS (ESI): *m*/*z* calcd. for C_17_H_22_N_5_O_10_S [M−H]^−^: 488.1093; found, 488.1064.

#### 5.1.46. (2R,3S,4S,5R)-(5-(7-Amino-5-(Hydroxymethyl)-1H-Benzo[d]imidazol-1-yl)-3,4-Dihydroxytetrahydrofuran-2-yl)methyl Leucylsulfamate (**32e**)

This compound was synthesized in analogy to **32a** starting from 80 mg (0.13 mmol) of **31e**. Yield: 82%. ^1^H NMR (300 MHz, D_2_O) δ 8.26 (d, *J* = 2.3 Hz, 1H), 7.10 (d, *J* = 1.9 Hz, 1H), 6.70 (q, *J* = 1.4 Hz, 1H), 6.06 (dd, *J* = 6.6, 2.3 Hz, 1H), 4.64–4.19 (m, 7H), 3.59 (td, *J* = 5.4, 2.5 Hz, 1H), 1.66–1.21 (m, 3H), 0.71 (dd, *J* = 6.0, 2.1 Hz, 6H). ^13^C NMR (75 MHz, D_2_O) δ 176.02, 144.13, 141.89, 136.45, 132.51, 122.55, 111.30, 108.93, 88.19, 82.12, 73.10, 69.52, 68.03, 63.60, 53.80, 39.81, 23.62, 21.39, 20.41. HRMS (ESI): *m*/*z* calcd. for C_19_H_28_N_5_O_8_S [M−H]^−^: 486.1664; found, 486.1666.

#### 5.1.47. (2R,3S,4S,5R)-(5-(7-Amino-5-(Hydroxymethyl)-1H-Benzo[d]imidazol-1-yl)-3,4-Dihydroxytetrahydrofuran-2-yl)methyl Isoleucylsulfamate (**32f**)

This compound was synthesized in analogy to **32a** starting from 158 mg (0.25 mmol) of **31f**. Yield: 84%. ^1^H NMR (300 MHz, D_2_O) δ 8.35 (s, 1H), 7.12 (s, 1H), 6.74 (d, *J* = 1.3 Hz, 1H), 6.10 (d, *J* = 6.5 Hz, 1H), 4.67–4.19 (m, 7H), 3.54 (dd, *J* = 4.3, 1.2 Hz, 1H), 3.11 (d, *J* = 7.3 Hz, 1H), 1.76 (s, 1H), 1.37–1.09 (m, 2H), 1.09–0.54 (m, 6H). ^13^C NMR (75 MHz, D_2_O) δ 174.77, 144.16, 141.79, 136.43, 132.51, 122.59, 111.24, 108.92, 88.14, 82.12, 73.06, 69.59, 68.05, 63.61, 59.73, 35.97, 23.63, 14.02, 10.44. HRMS (ESI): *m*/*z* calcd. for C_19_H_28_N_5_O_8_S [M−H]^−^: 486.1664; found, 486.1664.

### 5.2. Preparation of Different Aminoacyl tRNA Synthetases

The cloning, expression and purification of all aaRSs were performed as previously described [3,25,29].

### 5.3. Radiolabel Aminoacyl Transfer Assay Using Purified E. coli aaRSs

The general method and procedures were carried out according to Zhang et al. [3] and Nautiyal et al. [25]. The aminoacylation assay was performed with following enzyme concentrations and reaction times: AspRS (2 nM; 3 min); SerRS (2 nM; 6 min); TyrRS (0.5 nM; 8 min); LeuRS (2.5 nM; 4 min); IleRS (10 nM; 6 min).

### 5.4. Crystallization, Data Collection and Structure Determination

The crystallization procedures for *Neisseria gonorrhoeae* LeuRS, *E. coli* TyrRS, *Klebsiella pneumoniae* SerRS and *Thermus thermophilus* AspRS were carried out as described in our previous work [25,29]. Suitable crystals were soaked with the respective synthesized aaS7HMDDA inhibitors in the corresponding cryo-protectant solution before being flash frozen in liquid nitrogen.

All the X-ray diffraction datasets of the aaRS–inhibitor complexes were collected at synchrotron facilities. The datasets were processed by applying the autoproc package [33]. The structures were initially solved by molecular replacement with PHASER using our previously published aaSA-bound aaRS structures as the search models. Briefly, Protein Data Bank (PDB) structures 6Q89, 6I5Y, 6H9X and 6SJC were used as the model for LeuRS, TyrRS, SerRS and AspRS, respectively. After initial refinement, the molecular replacement solution was completed manually in COOT [34] and refined in Phenix. Structure quality was analyzed by PDB validation. The crystallographic data collection and refinement statistics are presented in Table 2.

## Figures and Tables

**Table 1:** $K_{i}^{app}$ values of the aminoacylated sulfonamide nucleosides for the respective class I enzymes are given in nM.

Class I aaRSs	$K_{i}^{app}(\mathrm{aaSA})\;*$	$K_{i}^{app}(\mathrm{aaS}7\mathrm{HMDDA})$
IleRS	1.92 ± 4.0	10.8 ± 0.8
LeuRS	0.14 ± 0.10	21.5 ± 1.2
TyrRS	2.93 ± 1.2	1232.2 ± 239.1

* The $K_{i}^{app}$ values for the adenosine derivatives were taken from our prior work [3].

## Supplementary Materials

The following are available online. Molecules-960294-Supporting Information-final.pdf.

## References

[1] Vondenhoff G.H.M., Van Aerschot A. (2011). Aminoacyl-tRNA synthetase inhibitors as potential antibiotics. Eur. J. Med. Chem..

[2] Eriani G., Delarue M., Poch O., Gangloff J., Moras D. (1990). Partition of tRNA synthetases into two classes based on mutually exclusive sets of sequence motifs. Nature.

[3] Zhang B., De Graef S., Nautiyal M., Pang L., Gadakh B., Froeyen M., Van Mellaert L., Strelkov S.V., Weeks S.D., Van Aerschot A. (2018). Family-wide analysis of aminoacyl-sulfamoyl-3-deazaadenosine analogues as inhibitors of aminoacyl-tRNA synthetases. Eur. J. Med. Chem..

[4] Redwan I.N., Ljungdahl T., Grøtli M. (2012). Investigation, optimization and synthesis of sulfamoyloxy-linked aminoacyl-AMP analogues. Tetrahedron.

[5] Shi C., Tiwari D., Wilson D.J., Seiler C.L., Schnappinger D., Aldrich C.C. (2013). Bisubstrate Inhibitors of Biotin Protein Ligase in Mycobacterium tuberculosis Resistant to Cyclonucleoside Formation. ACS Med. Chem. Lett..

[6] Gadakh B., Vondenhoff G., Lescrinier E., Rozenski J., Froeyen M., Van Aerschot A. (2014). Base substituted 5′-*O*-(N-isoleucyl)sulfamoyl nucleoside analogues as potential antibacterial agents. Bioorg. Med. Chem..

[7] Lee J., Kim S.E., Lee J.Y., Kim S.Y., Kang S.U., Seo S.H., Chun M.W., Kang T., Choi S.Y., Kim H.O. (2003). *N*-Alkoxysulfamide, *N*-hydroxysulfamide, and sulfamate analogues of methionyl and isoleucyl adenylates as inhibitors of methionyl-tRNA and isoleucyl-tRNA synthetases. Bioorg. Med. Chem. Lett..

[8] Zhang H.-Z., Rao K., Carr S.F., Papp E., Straub K., Wu J.C., Fried J. (1997). Rationally Designed Inhibitors of Inosine Monophosphate Dehydrogenase. J. Med. Chem..

[9] Bonhôte P., Wrighton M.S. (1997). Facile Synthesis of Tetrapyrido[2,3-a:3’,2’-c:2”,3”-h:3’”,2’”-j]phenazine. Synlett.

[10] Nair V., Purdy D.F., Sells T.B. (1989). Synthesis of congeners of adenosine resistant to deamination by adenosine deaminase. J. Chem. Soc. Chem. Commun..

[11] Nair V., Lyons A.G. (1989). Novel unsaturated purine nucleosides. Tetrahedron.

[12] Nair V., Ussery M.A. (1992). New hypoxanthine nucleosides with RNA antiviral activity. Antivir. Res..

[13] Cain R., Salimraj R., Punekar A.S., Bellini D., Fishwick C.W.G., Czaplewski L., Scott D.J., Harris G., Dowson C.G., Lloyd A.J. (2019). Structure-Guided Enhancement of Selectivity of Chemical Probe Inhibitors Targeting Bacterial Seryl-tRNA Synthetase. J. Med. Chem..

[14] Schimmel P., Tao J., Hill J. (1998). Aminoacyl tRNA synthetases as targets for new anti-infectives. FASEB J..

[15] Zeng Y., Roy H., Patil P.B., Ibba M., Chen S. (2009). Characterization of two seryl-tRNA synthetases in albomycin-producing streptomyces sp. Strain ATCC 700974. Antimicrob. Agents Chemother..

[16] Elkin V.V., Tolkacheva L.N., Chernysheva N.B., Karmanova I.B., Konyushkin L.D., Semenov V.V. (2007). Hydrogenation on palladium containing granulated catalysts; 3. Synthesis of aminobenzimidazoles by catalytic hydrogenation of dinitroanilines. Russ. Chem. Bull. Int. Ed..

[17] Milata V., Saloň J. (1999). Simple, high yield preparation of 3-nitro-1,2-phenylenediamine. Org. Prep. Proced. Int..

[18] Ida Y., Matsubara A., Nemoto T., Saito M., Hirayama S., Fujii H., Nagase H. (2012). Synthesis of quinolinomorphinan derivatives as highly selective δ opioid receptor ligands. Bioorg. Med. Chem..

[19] Pathak A.K., El-Kattan Y.A., Bansal N., Maddry J.A., Reynolds R.C. (1998). Tin(IV) chloride mediated glycosylation in arabinofuranose, galactofuranose and rhamnopyranose. Tetrahedron Lett..

[20] Vorbrüggen H., Krolikiewicz K., Bennua B. (1981). Nucleoside syntheses, XXII1) Nucleoside synthesis with trimethylsilyl triflate and perchlorate as catalysts. Chem. Ber..

[21] Ashcroft C.P., Dessi Y., Entwistle D.A., Hesmondhalgh L.C., Longstaff A., Smith J.D. (2012). Route Selection and Process Development of a Multikilogram Route to the Inhaled A2a Agonist UK-432,097. Org. Process Res. Dev..

[22] Eckenberg P., Groth U., Huhn T., Richter N., Schmeck C. (1993). A useful application of benzyl trichloroacetimidate for the benzylation of alcohols. Tetrahedron.

[23] Ombouma J., Vullo D., Dumy P., Supuran C.T., Winum J.-Y. (2015). Carbonic Anhydrase Glycoinhibitors belonging to the Aminoxysulfonamide Series. ACS Med. Chem. Lett..

[24] Copeland R.A. (2013). Tight Binding Inhibition. Evaluation of Enzyme Inhibitors in Drug Discovery.

[25] Nautiyal M., De Graef S., Pang L., Gadakh B., Strelkov S.V., Weeks S.D., Van Aerschot A. (2019). Comparative analysis of pyrimidine substituted aminoacyl-sulfamoyl nucleosides as potential inhibitors targeting class I aminoacyl-tRNA synthetases. Eur. J. Med. Chem..

[26] Adams P.D., Afonine P.V., Bunkoczi G., Chen V.B., Davis I.W., Echols N., Headd J.J., Hung L.-W., Kapral G.J., Grosse-Kunstleve R.W. (2010). PHENIX: A comprehensive Python-based system for macromolecular structure solution. Acta Crystallogr. Sect. D Struct. Biol..

[27] Khoje A.D., Charnock C., Wan B., Franzblau S., Gundersen L.-L. (2011). Synthesis and antimycobacterial activities of non-purine analogs of 6-aryl-9-benzylpurines: Imidazopyridines, pyrrolopyridines, benzimidazoles, and indoles. Bioorg. Med. Chem..

[28] Krajczyk A., Zeidler J., Januszczyk P., Dawadi S., Boshoff H.I., Barry C.E., Ostrowski T., Aldrich C.C. (2016). 2-Aryl-8-aza-3-deazaadenosine analogues of 5’-O-[N-(salicyl)sulfamoyl]adenosine: Nucleoside antibiotics that block siderophore biosynthesis in Mycobacterium tuberculosis. Bioorg. Med. Chem..

[29] Pang L., Nautiyal M., De Graef S., Gadakh B., Zorzini V., Economou A., Strelkov S.V., Van Aerschot A., Weeks S.D. (2020). Structural Insights into the Binding of Natural Pyrimidine-Based Inhibitors of Class II Aminoacyl-tRNA Synthetases. ACS Chem. Biol..

[30] Devlin T.A., Jebaratnam D.J. (1995). An Improved Procedure for the Synthesis of 1,3-Dideazaadenosine. Synth. Commun..

[31] Boehr D.D., Farley A.R., LaRonde F.J., Murdock T.R., Wright G.D., Cox J.R. (2005). Establishing the Principles of Recognition in the Adenine-Binding Region of an Aminoglycoside Antibiotic Kinase [APH(3′)-IIIa]. Biochemistry.

[32] Townsend A.P., Roth S., Williams H.E., Stylianou E., Thomas N.R. (2009). New S-adenosyl-L-methionine analogues: Synthesis and reactivity studies. Org. Lett..

[33] Vonrhein C., Flensburg C., Keller P., Sharff A., Smart O., Paciorek W., Womack T., Bricogne G. (2011). Data processing and analysis with the autoPROC toolbox. Acta Crystallogr. Sect. D.

[34] Emsley P., Cowtan K. (2004). Coot: Model-building tools for molecular graphics. Acta Crystallogr. D Biol. Crystallogr..

