# Supplementary material for: Synthesis and Biological Evaluation of 1,3-Dideazapurine-Like 7-Amino-5-Hydroxymethyl-Benzimidazole Ribonucleoside Analogues as Aminoacyl-tRNA Synthetase Inhibitors
Source: Molecules. 2020 Oct 16;25(20):4751. doi: 10.3390/molecules25204751 (PMC7587597; doi:10.3390/molecules25204751)

Supporting Information

Synthesis and biological evaluation of 1,3-dideazapurine-like 7-amino-5-hydroxymethyl-benzimidazole ribonucleoside analogues as aminoacyl-tRNA synthetase inhibitors

Baole Zhang,[a,#,§] *Luping Pang,*[a,b,#] *Manesh Nautiyal,*[a] *Steff De Graef,*[b,¥] *Bharat Gadakh,*[a] *Eveline Lescrinier,*[a] *Jef Rozenski,*[a] *Sergei V. Strelkov,*[b] *Stephen D. Weeks,*[b,¥] *Arthur Van Aerschot*[a*]

[a] *KU Leuven, Rega Institute for Medical Research, Medicinal Chemistry, Herestraat 49 – box 1041, 3000 Leuven, Belgium*

[b] *KU Leuven, Laboratory of Biocristallography, Department of Pharmaceutical and Pharmacological Sciences, Herestraat 49 – box 822, 3000 Leuven, Belgium*

[#] These authors contributed equally to this work.

§ Present address: Hybio Medicine Park, No.37 Keji C, Str. 2nd, Shenzhen Hi-Tech Industrial Park ,P. R. China

¥ Present address: OrthogonX, Gaston Geenslaan 1, 3001 Leuven, Belgium

[*] *Corresponding author: Arthur.Vanaerschot@kuleuven.be*

Compound 2 in DMSO-d6 1H spectrum - 300 MHz

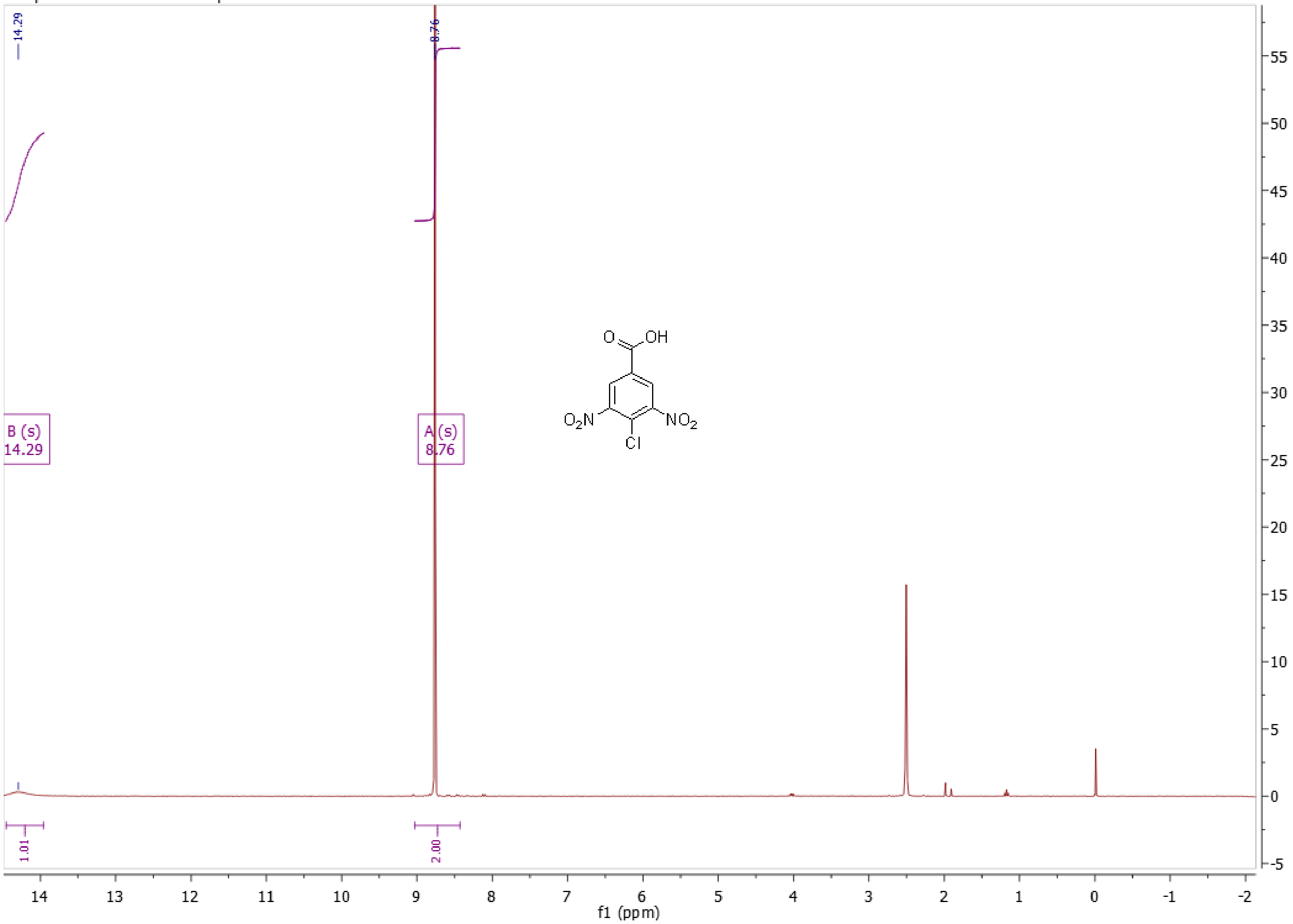

Compound 2 in DMSO-d6 13C spectrum - 75 MHz

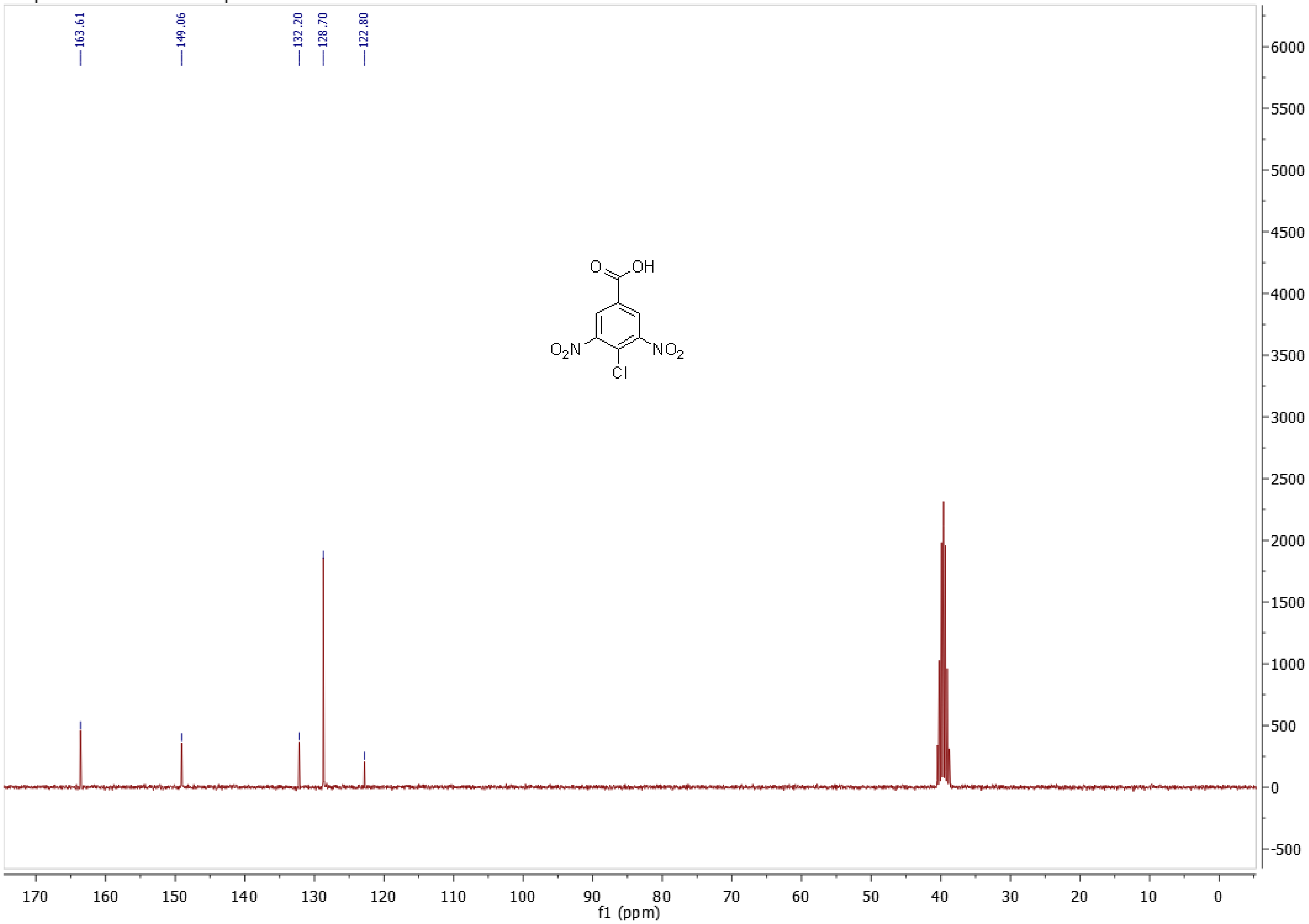

compound 2

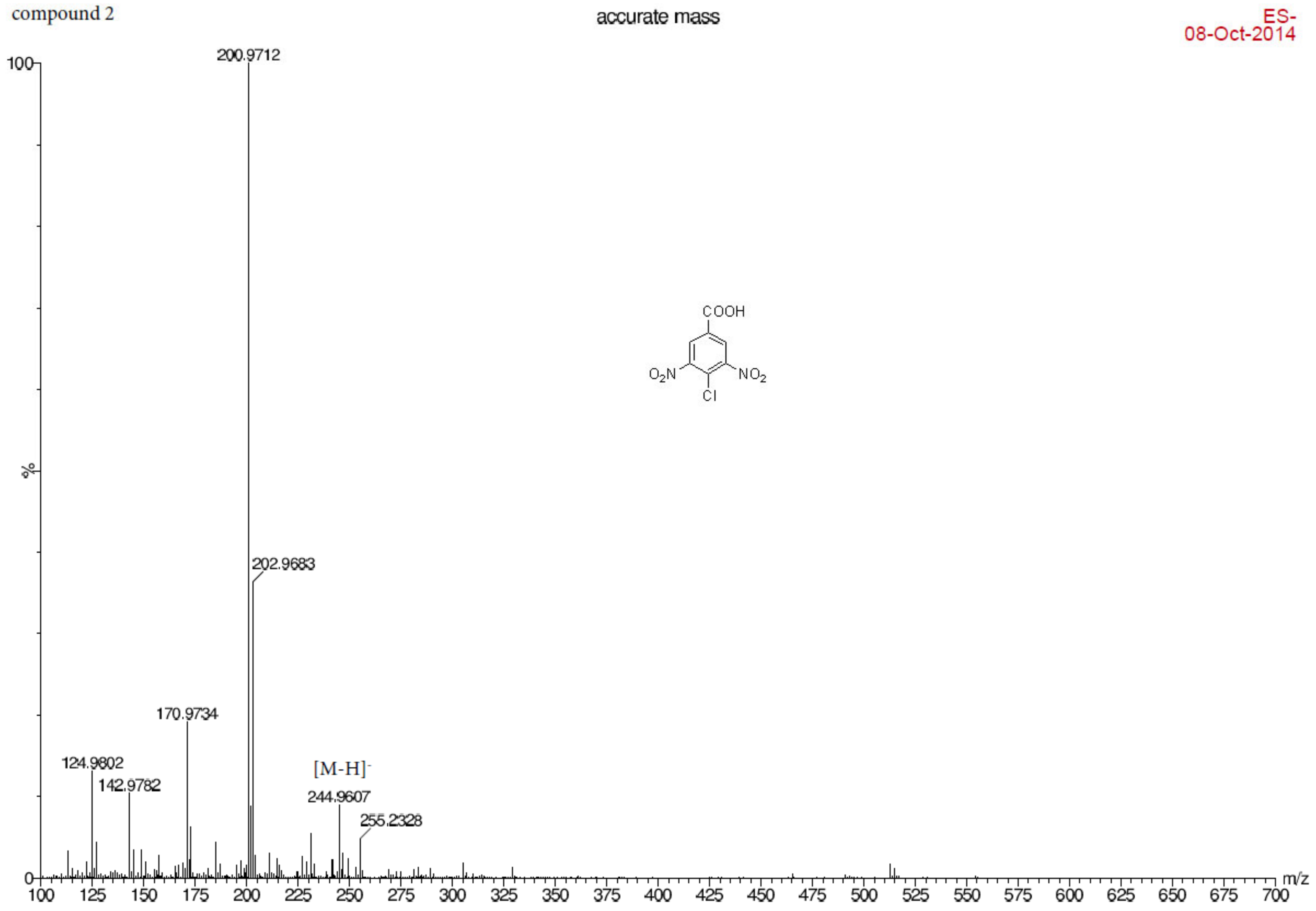
accurate mass
ES-
08-Oct-2014
100
%
0
200.9712
202.9683
170.9734
124.9802
142.9782
[M-H]-
244.9607
255.2328
100
125
150
175
200
225
250
275
300
325
350
375
400
425
450
475
500
525
550
575
600
625
650
675
700
m/z
COOH
O2N
NO2
Cl

Compound 3 in Acetone-d6 1H spectrum - 300 MHz

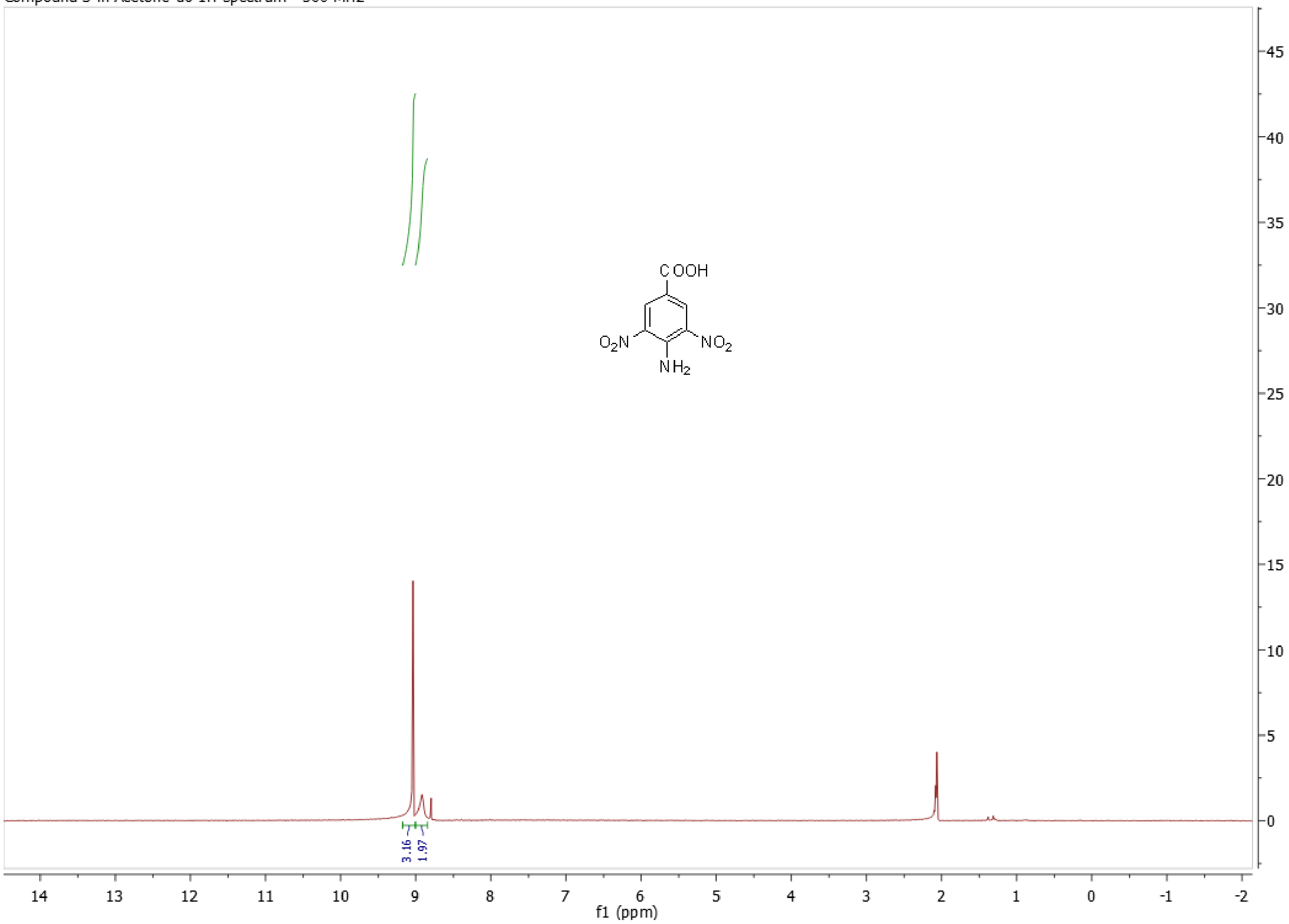

Compound 3 in Acetone-d6 13C spectrum - 75 MHz

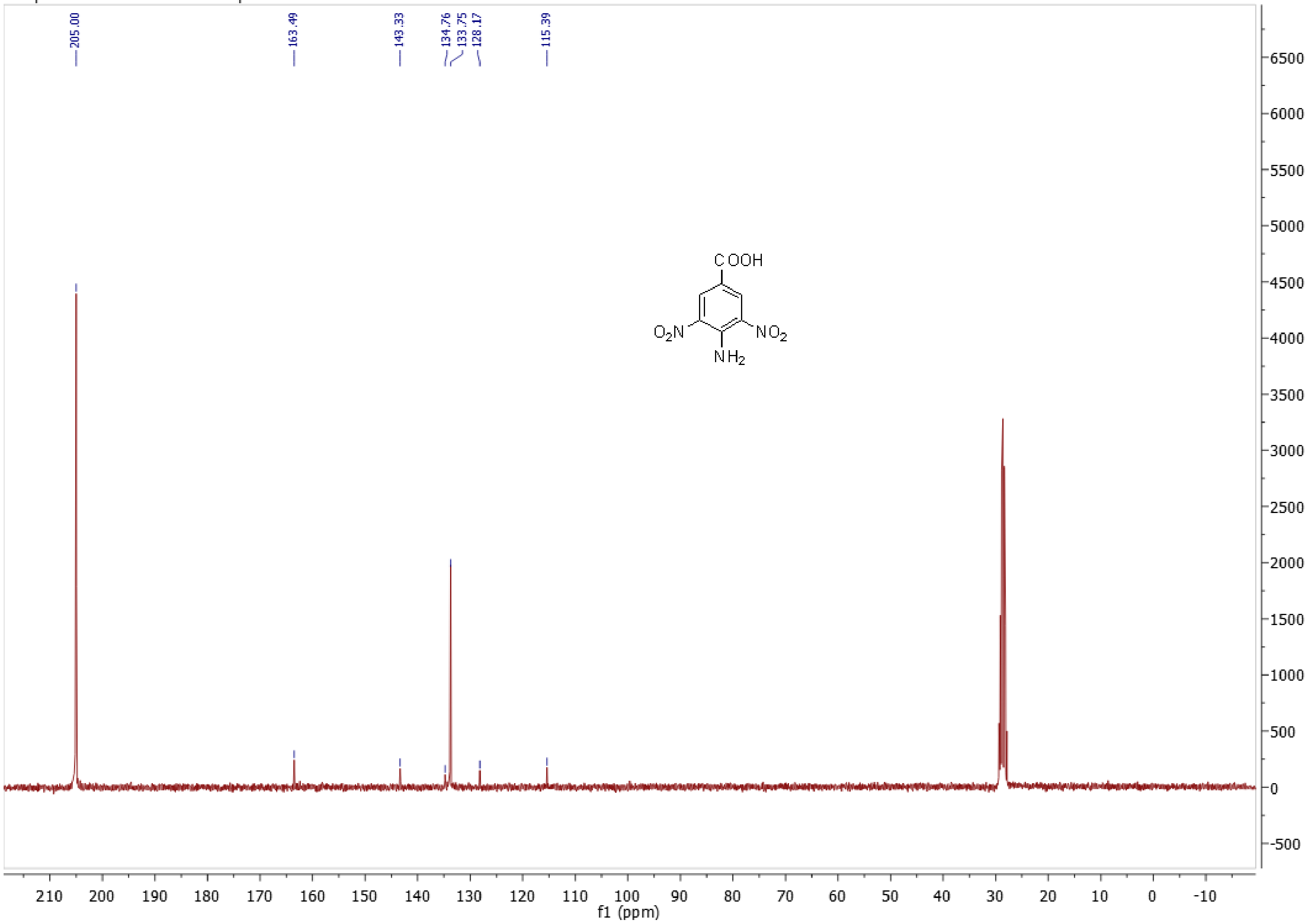

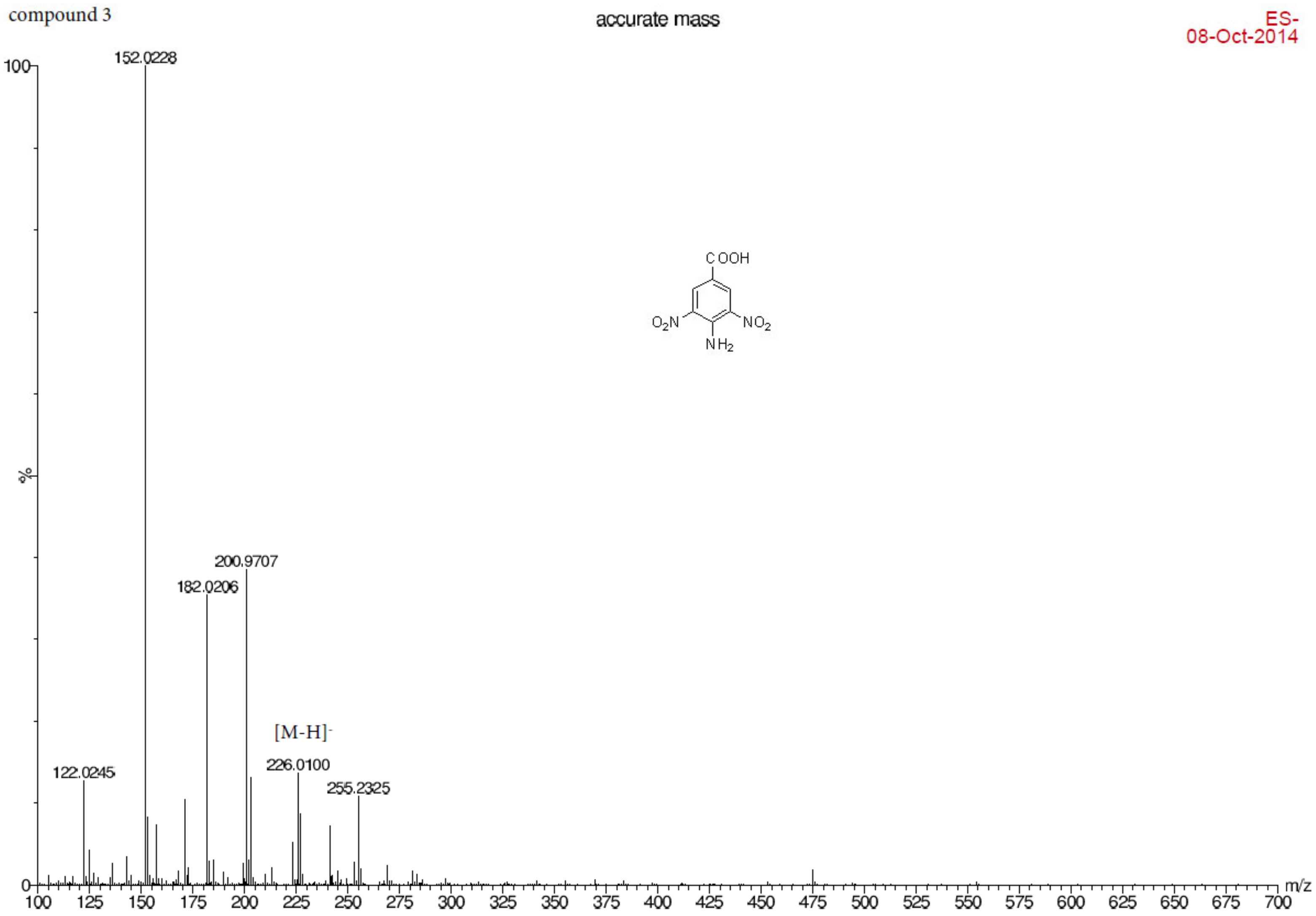
compound 3
accurate mass
ES-
08-Oct-2014
152.0228
200.9707
182.0206
[M-H]-
226.0100
122.0245
255.2325
COOH
O2N
NO2
NH2
%
m/z
100
0
100 125 150 175 200 225 250 275 300 325 350 375 400 425 450 475 500 525 550 575 600 625 650 675 700

Compound 4 in DMSO-d6 1H spectrum - 300 MHz

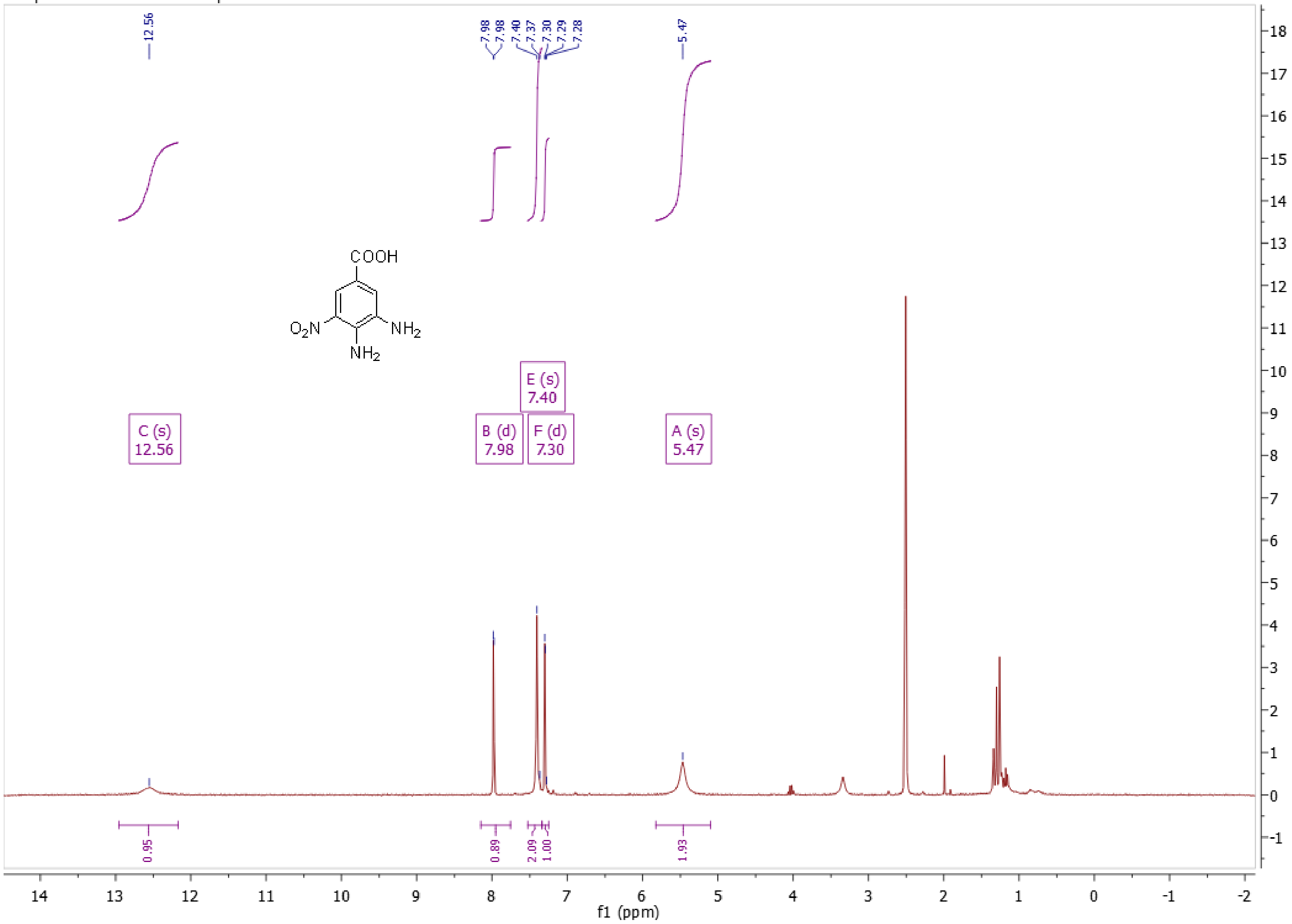

Compound 4 in DMSO-d6 13C spectrum - 75 MHz

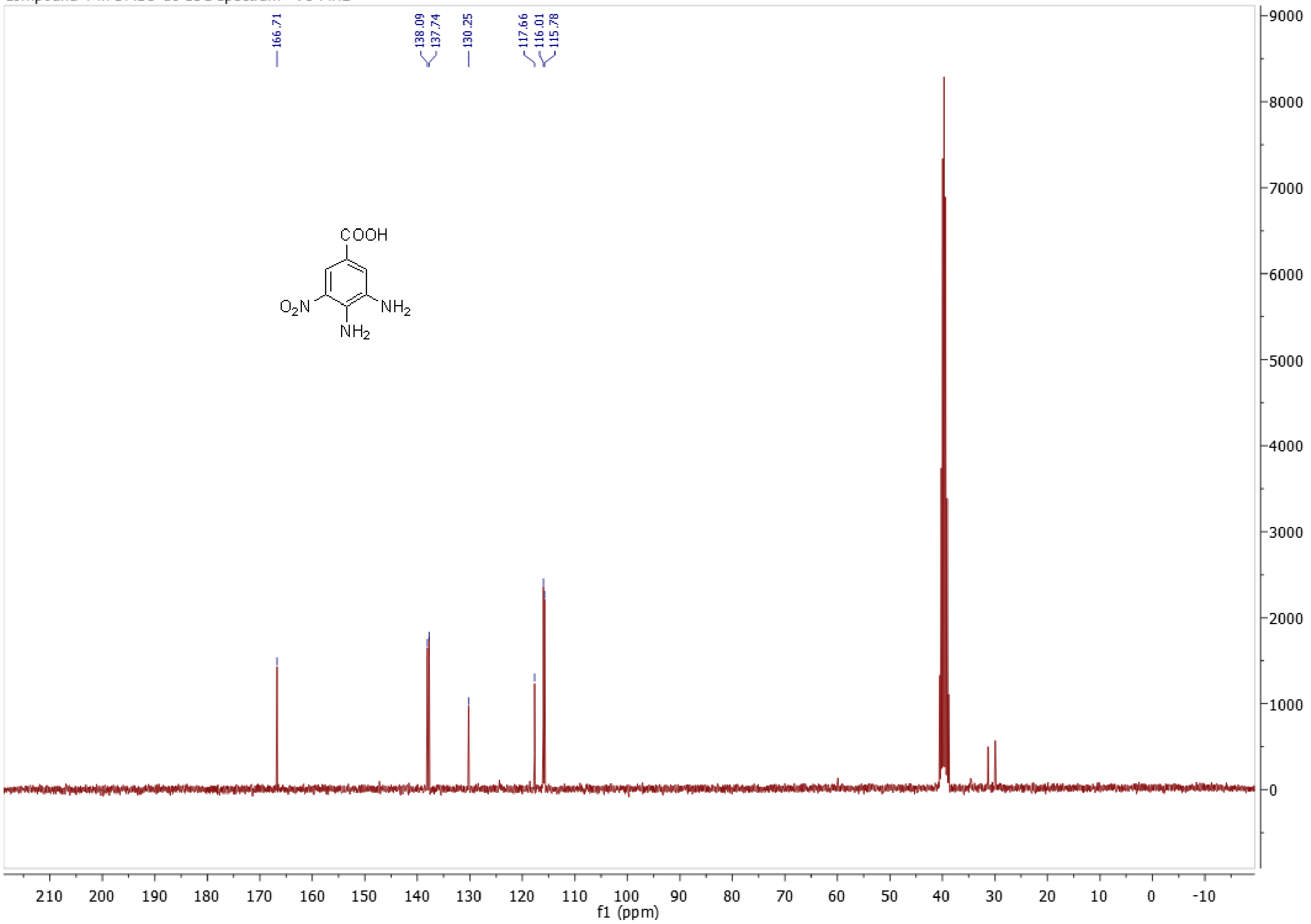

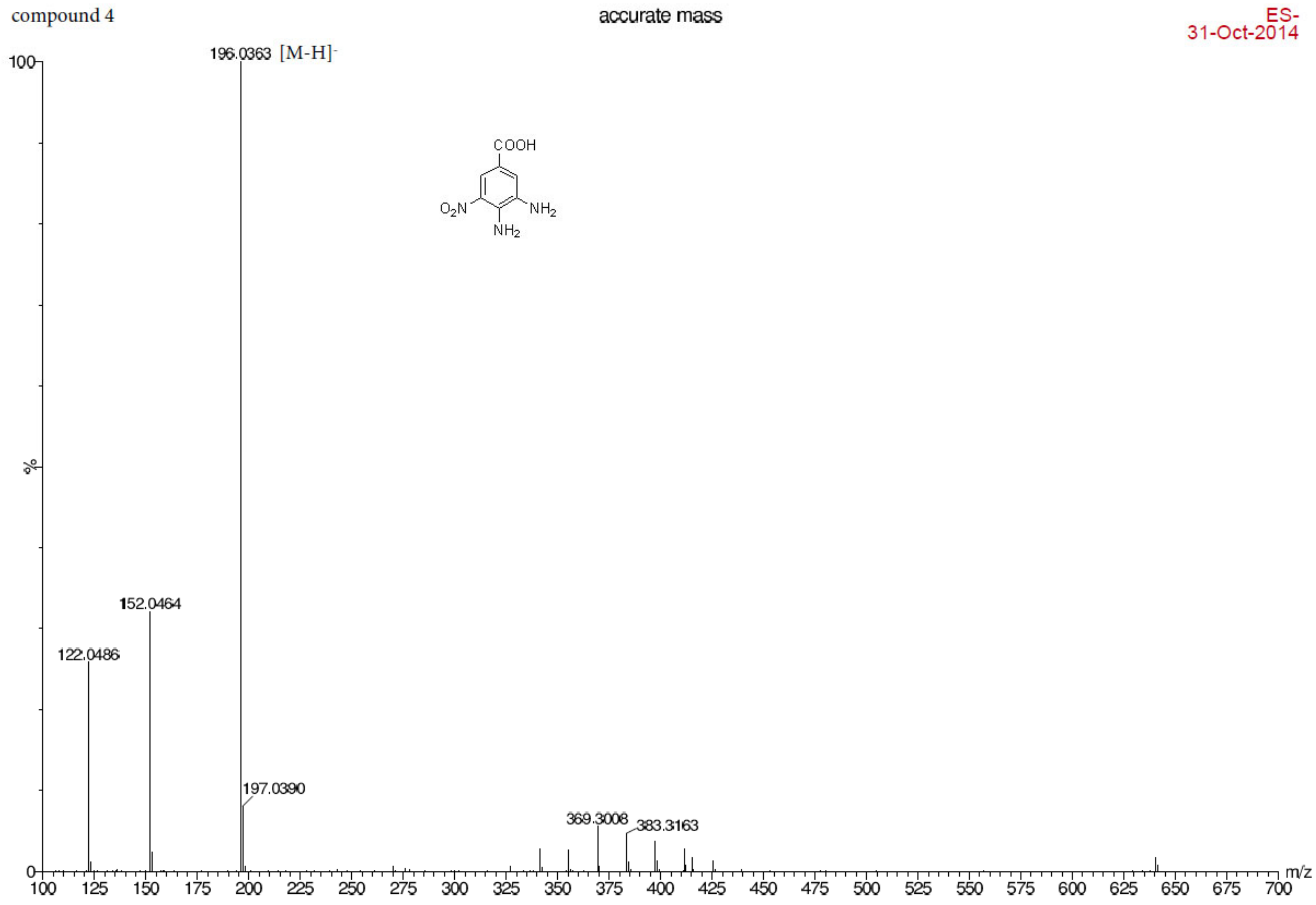

compound 4
accurate mass
ES-
31-Oct-2014
196.0363 [M-H]-
COOH
O_2N
NH_2
NH_2
152.0464
122.0486
197.0390
369.3008
383.3163
%
m/z

Compound 5 in DMSO-d6 1H spectrum - 300 MHz

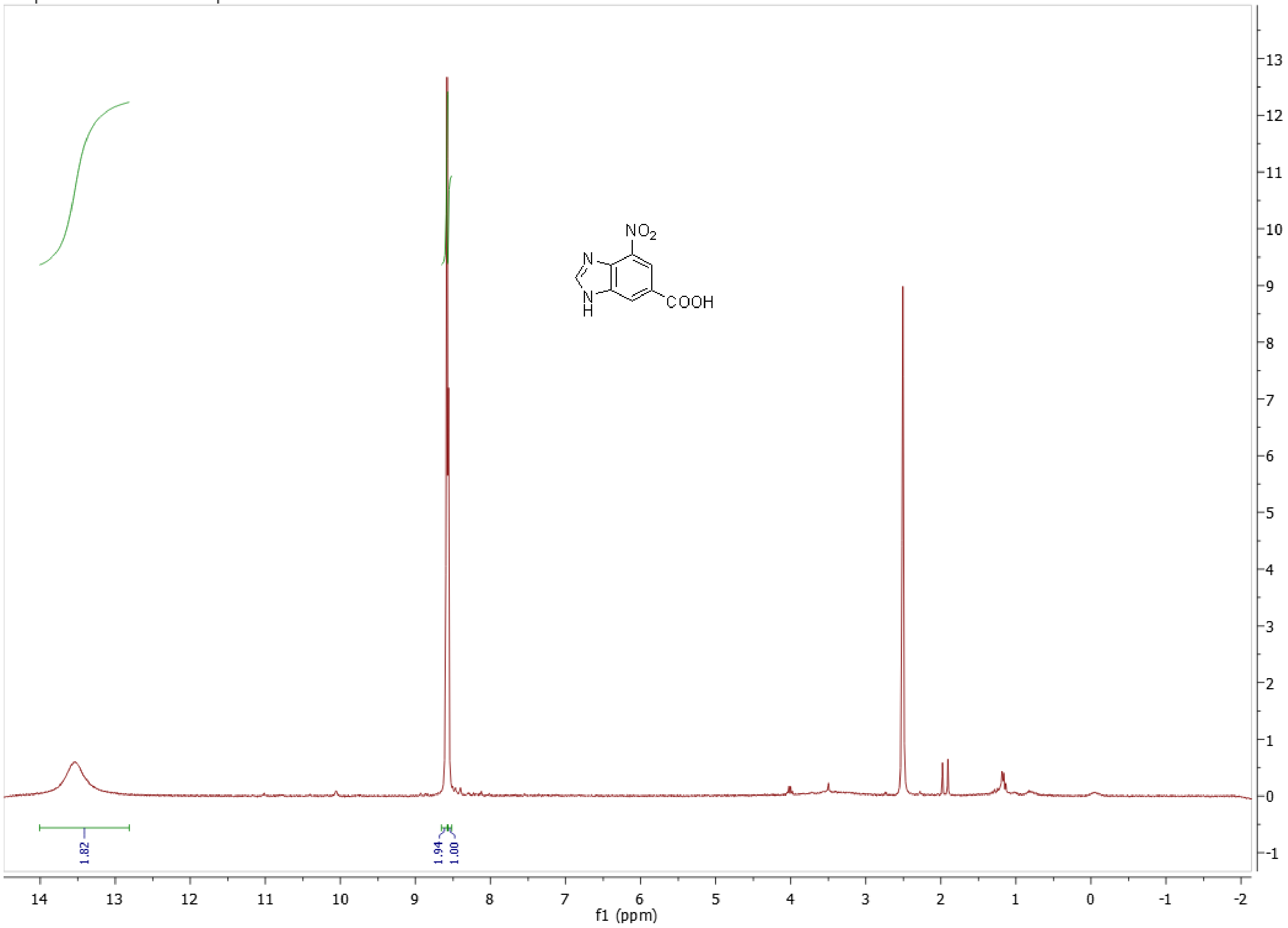

Compound 5 in DMSO-d6 13C spectrum - 75 MHz

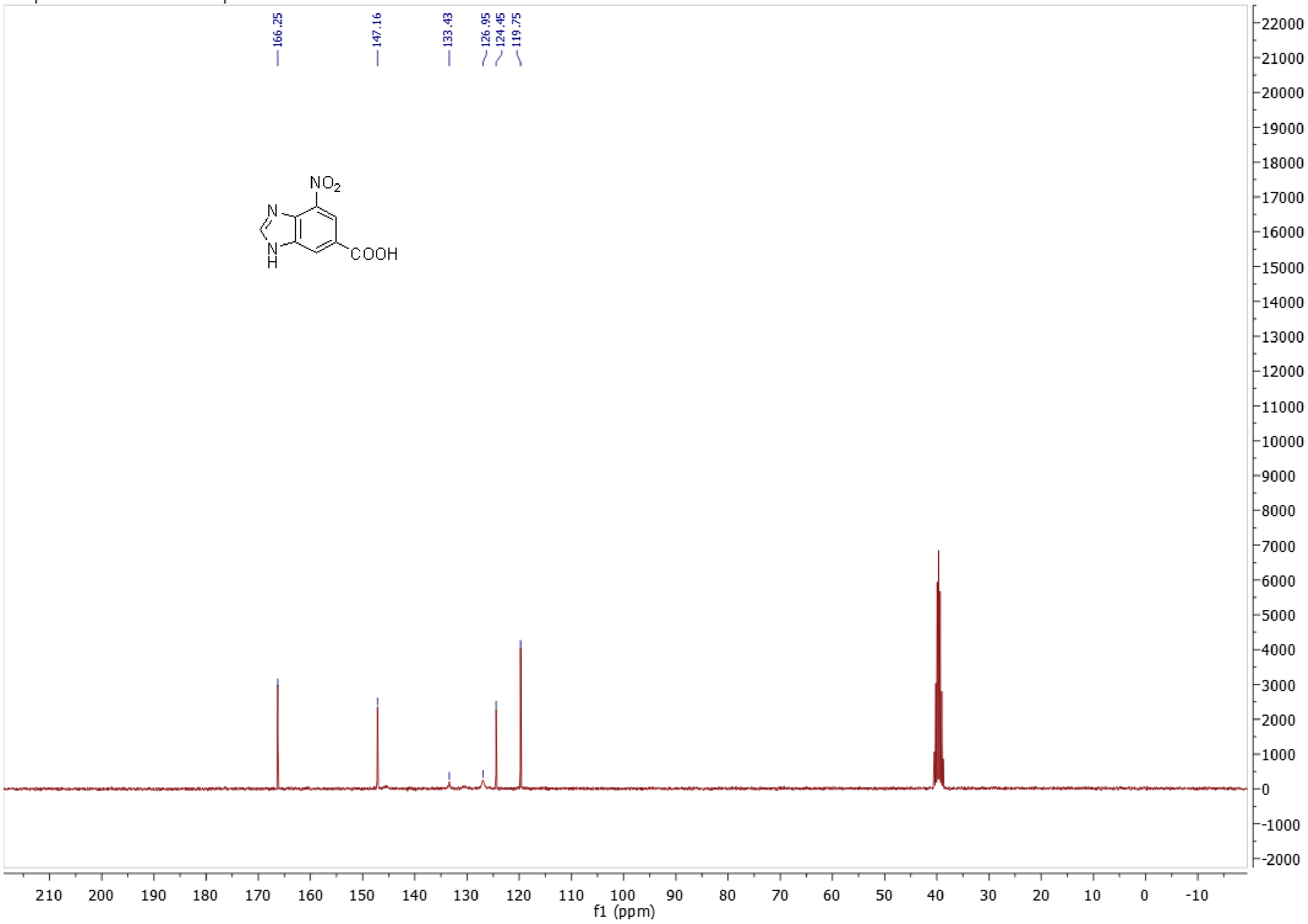

compound 5

accurate mass

ES-
31-Oct-2014

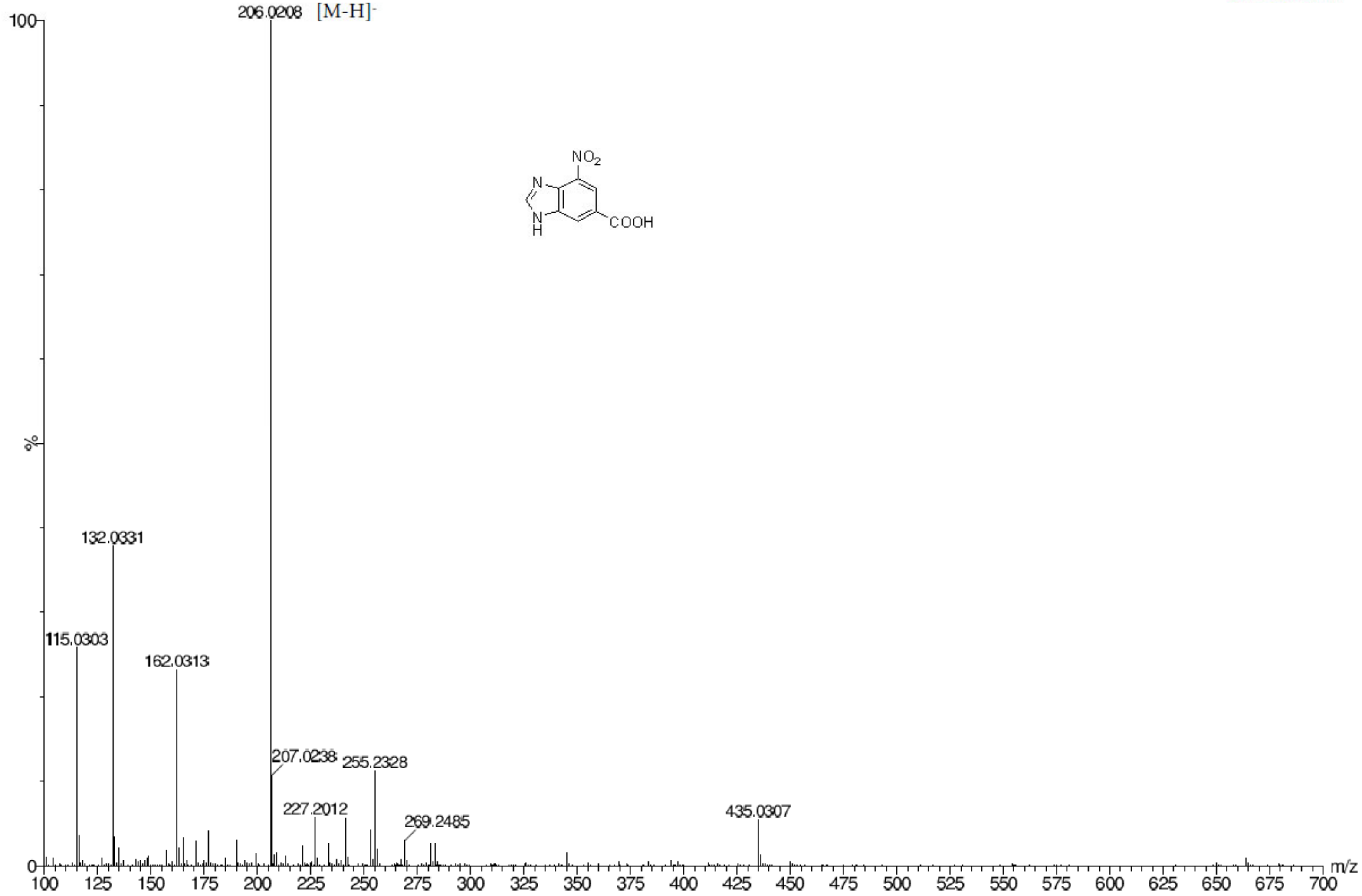

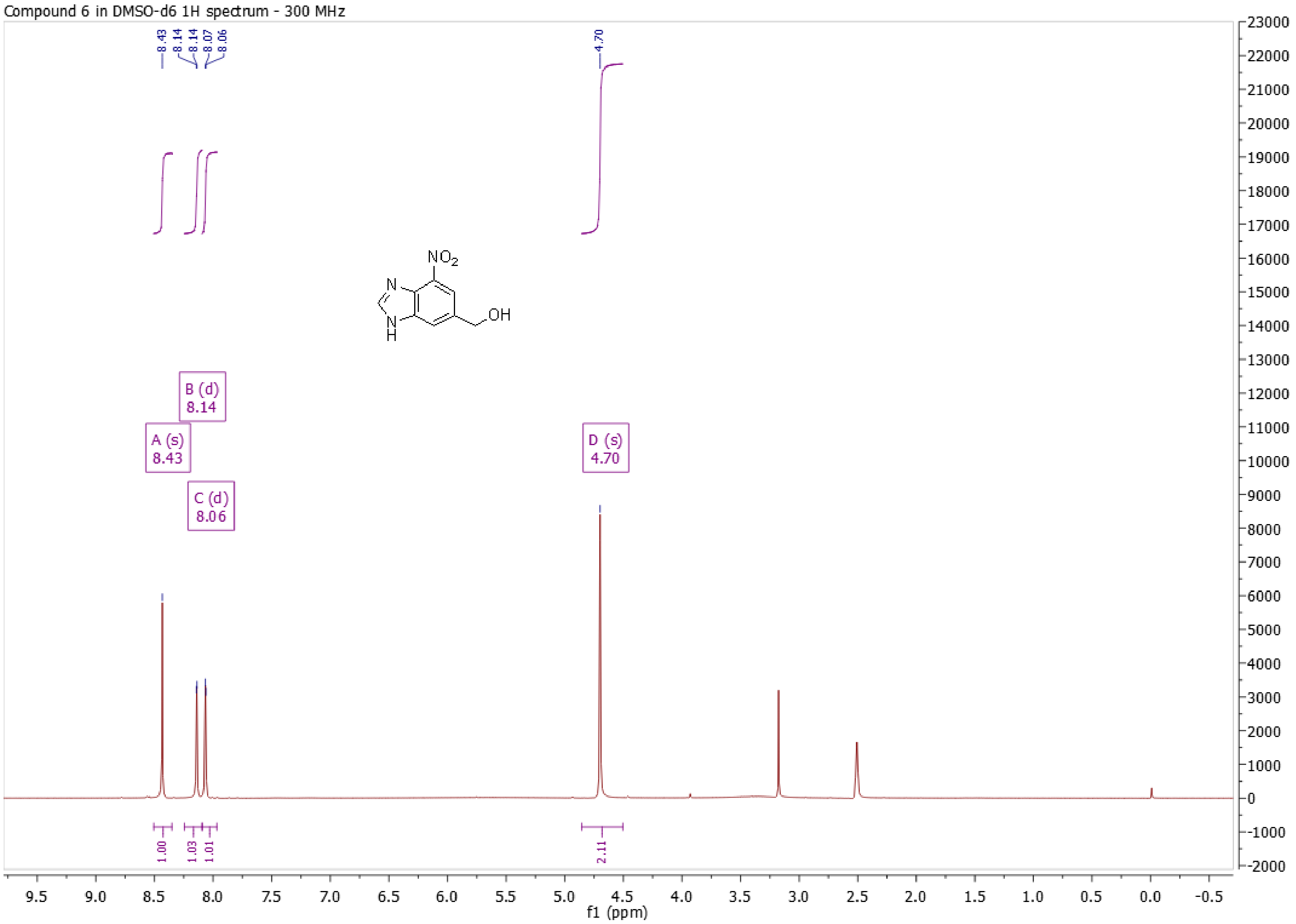
Compound 6 in DMSO-d6 1H spectrum - 300 MHz
8.43
8.14
8.14
8.07
8.06
4.70
NO2
N
N
H
OH
B (d)
8.14
A (s)
8.43
C (d)
8.06
D (s)
4.70
1.00
1.03
1.01
2.11
f1 (ppm)

Compound 6 in DMSO-d6 13C spectrum - 75 MHz

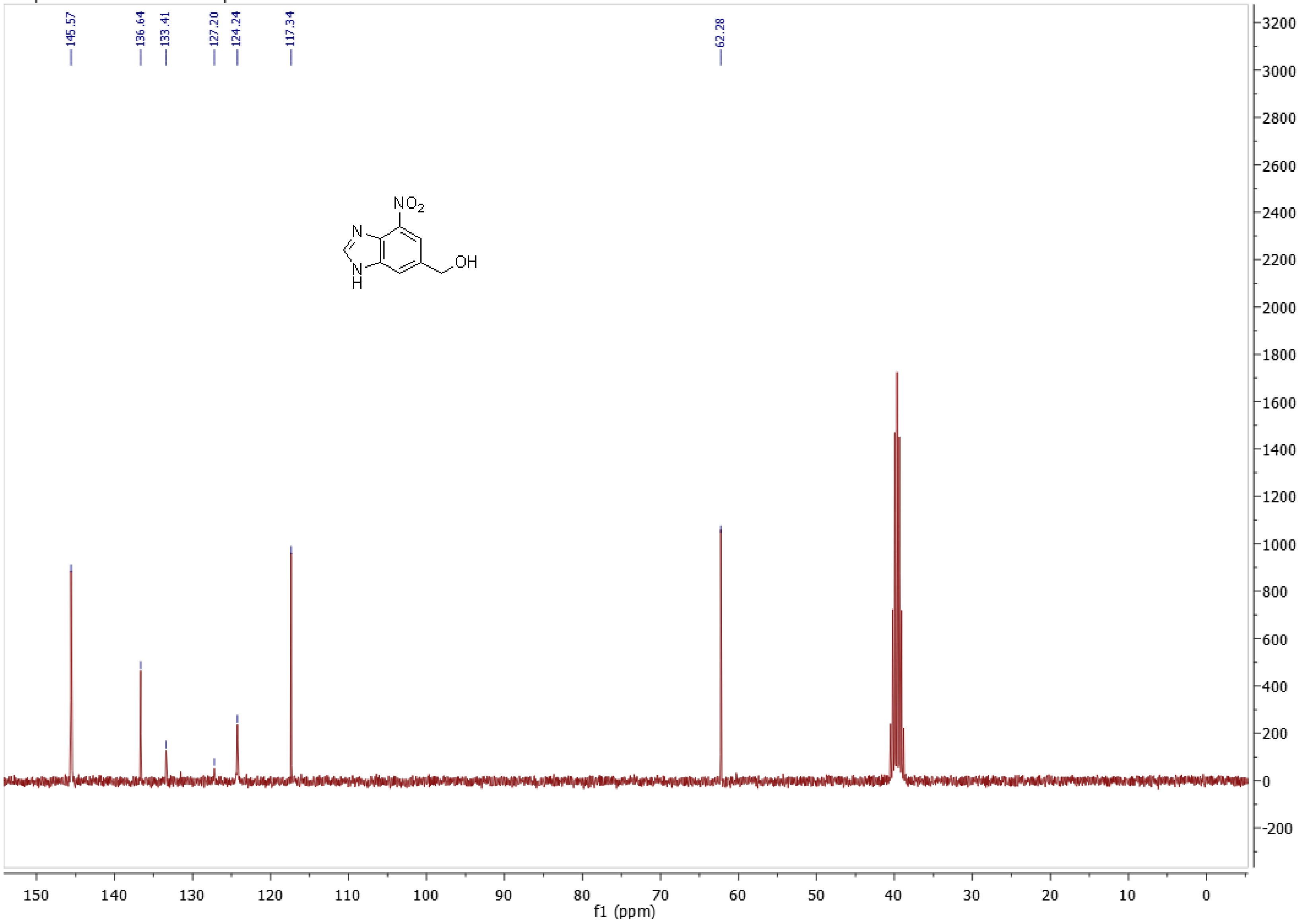

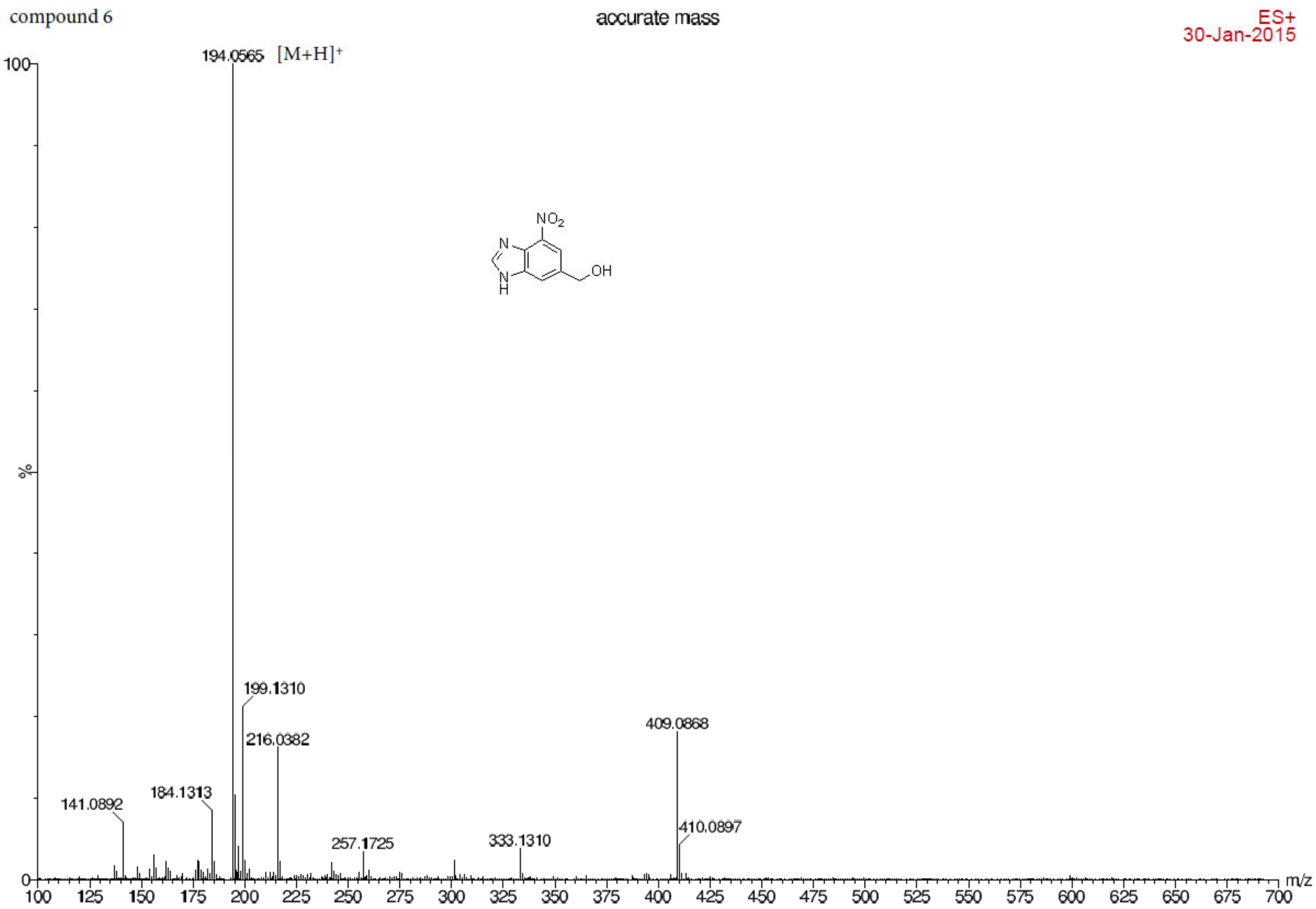
compound 6
accurate mass
ES+
30-Jan-2015
194.0565 [M+H]+
NO2
N
N
H
OH
199.1310
216.0382
409.0868
141.0892
184.1313
410.0897
257.1725
333.1310
%
100
0
100 125 150 175 200 225 250 275 300 325 350 375 400 425 450 475 500 525 550 575 600 625 650 675 700
m/z

Compound 7 in DMSO-d6 1H spectrum - 300 MHz

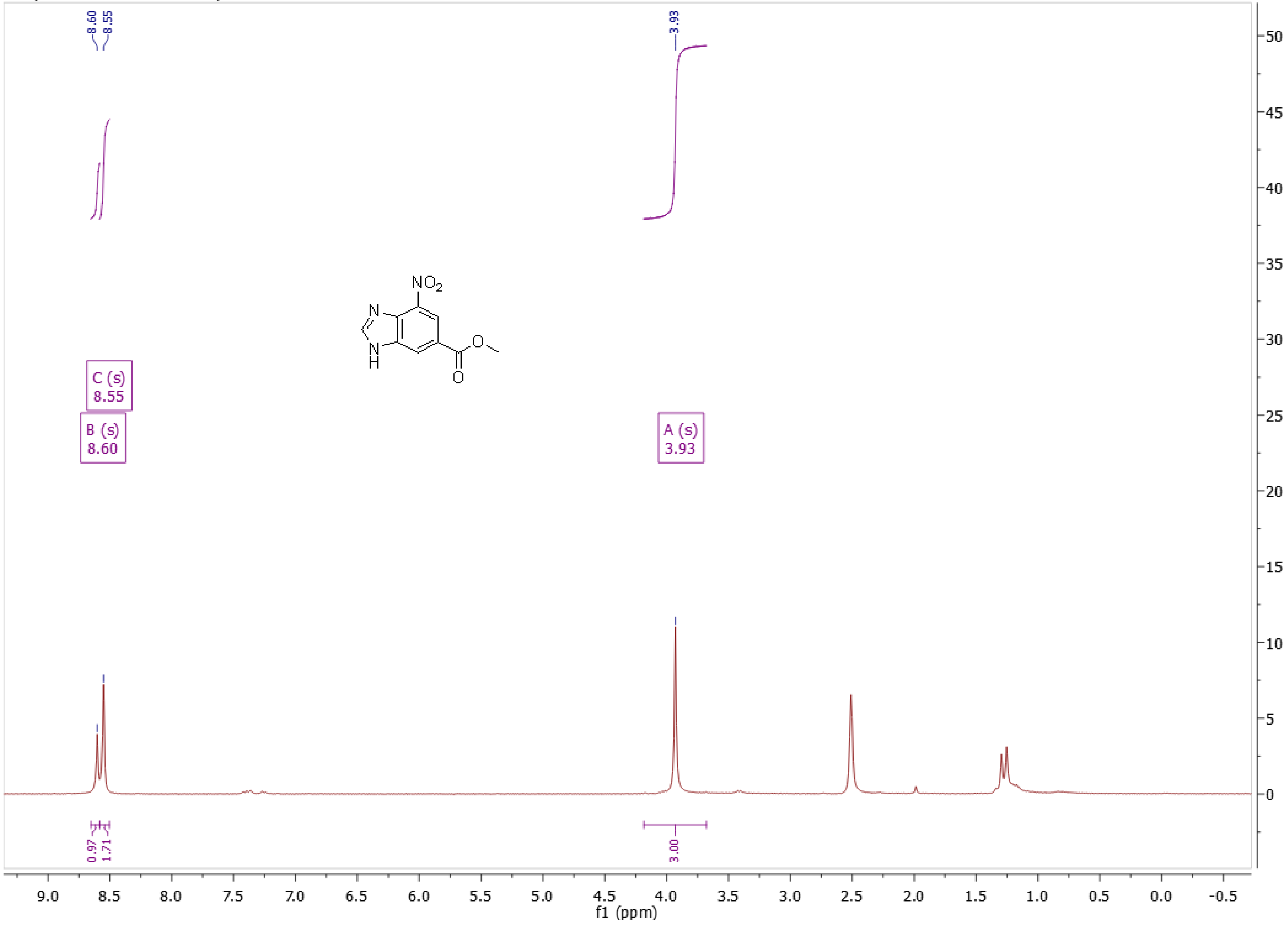

Compound 7 in DMSO-d6 13C spectrum - 75 MHz

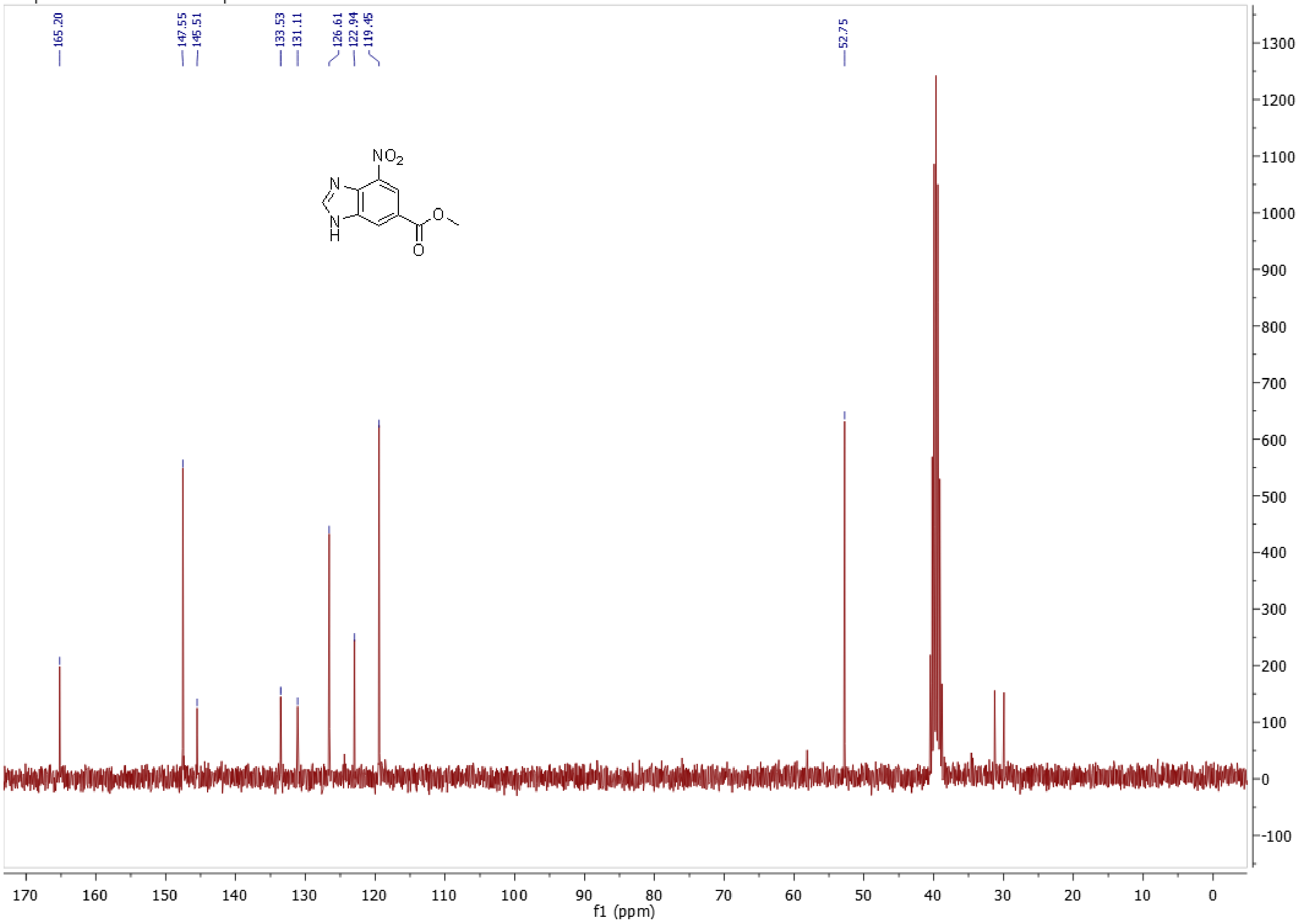

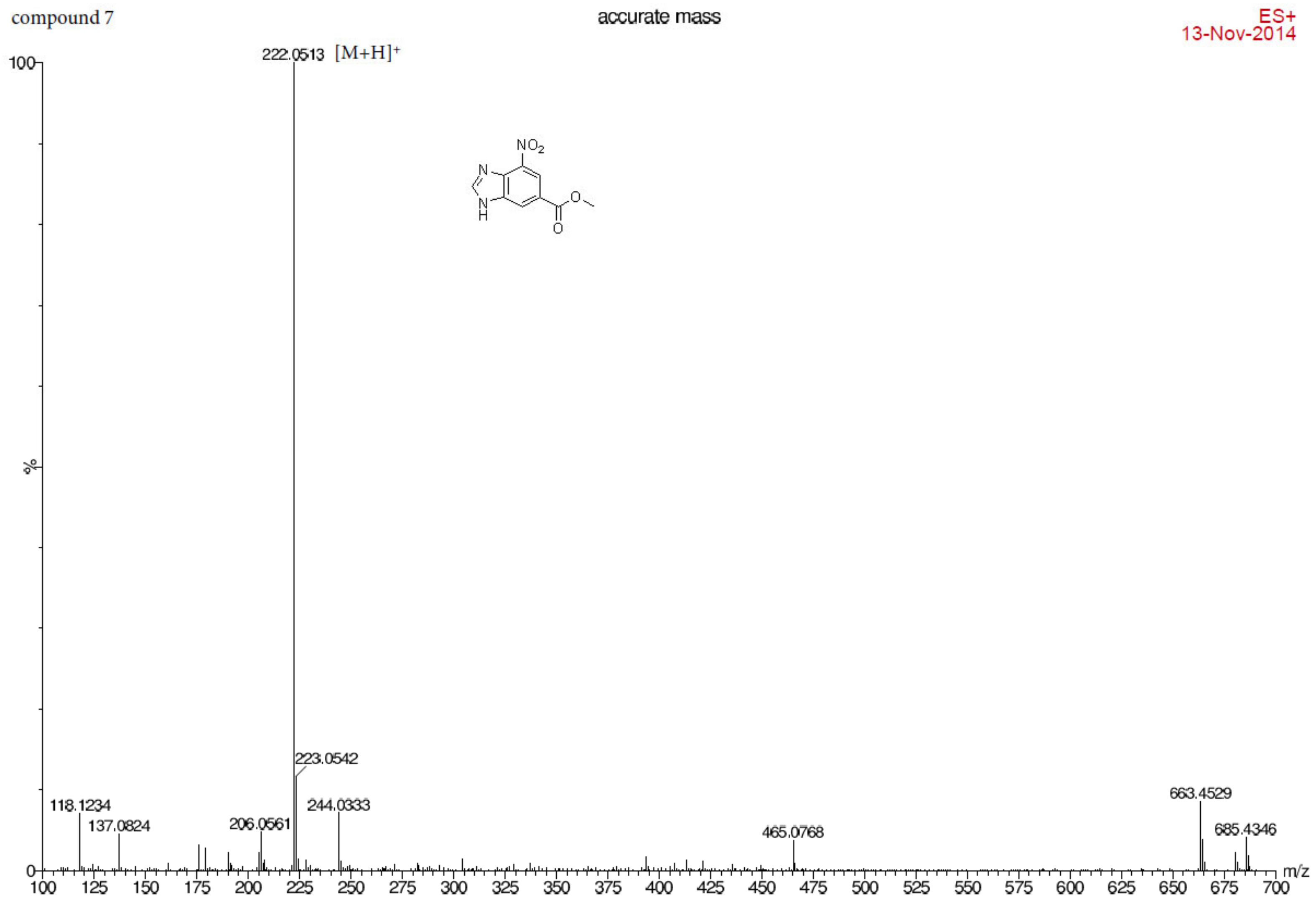
compound 7
accurate mass
ES+
13-Nov-2014
222.0513 [M+H]+
100
%
0
NO2
N
N
H
O
O
223.0542
118.1234
137.0824
206.0561
244.0333
465.0768
663.4529
685.4346
m/z
100 125 150 175 200 225 250 275 300 325 350 375 400 425 450 475 500 525 550 575 600 625 650 675 700

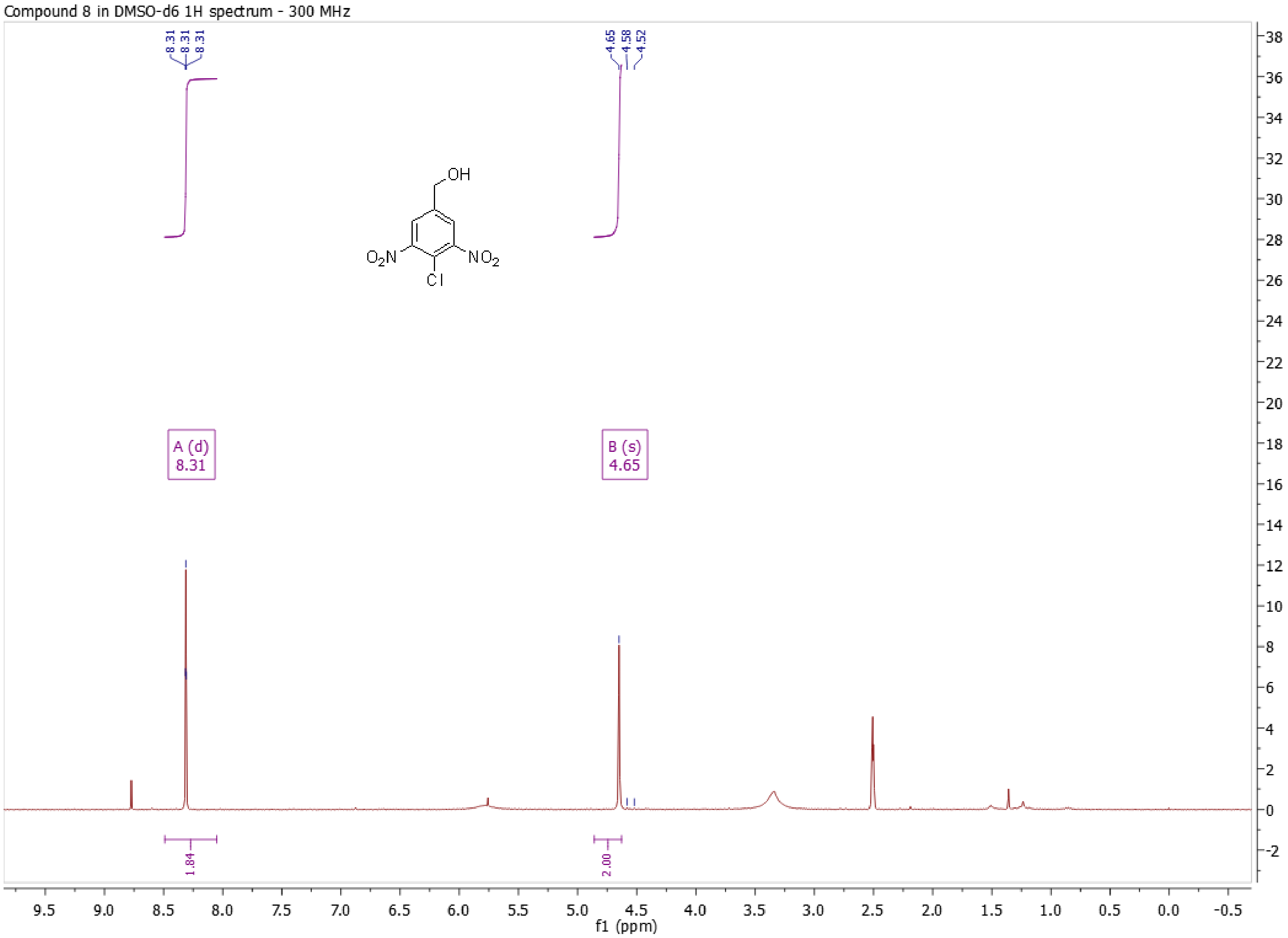
Compound 8 in DMSO-d6 1H spectrum - 300 MHz
8.31
8.31
8.31
4.65
4.58
4.52
OH
O_2N
NO_2
Cl
A (d)
8.31
B (s)
4.65
1.84
2.00
f1 (ppm)

Compound 8 in DMSO-d6 13C spectrum - 75 MHz

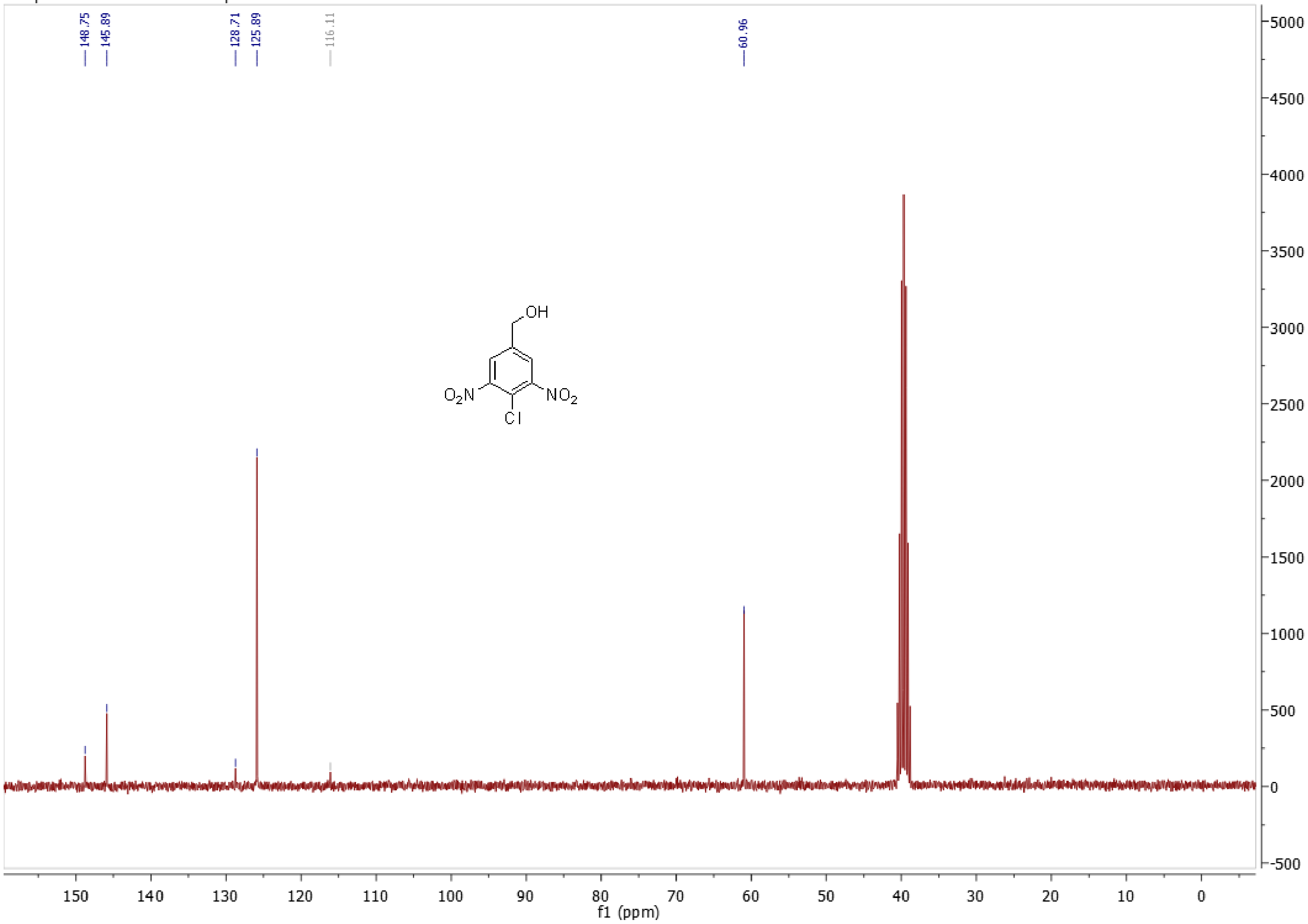

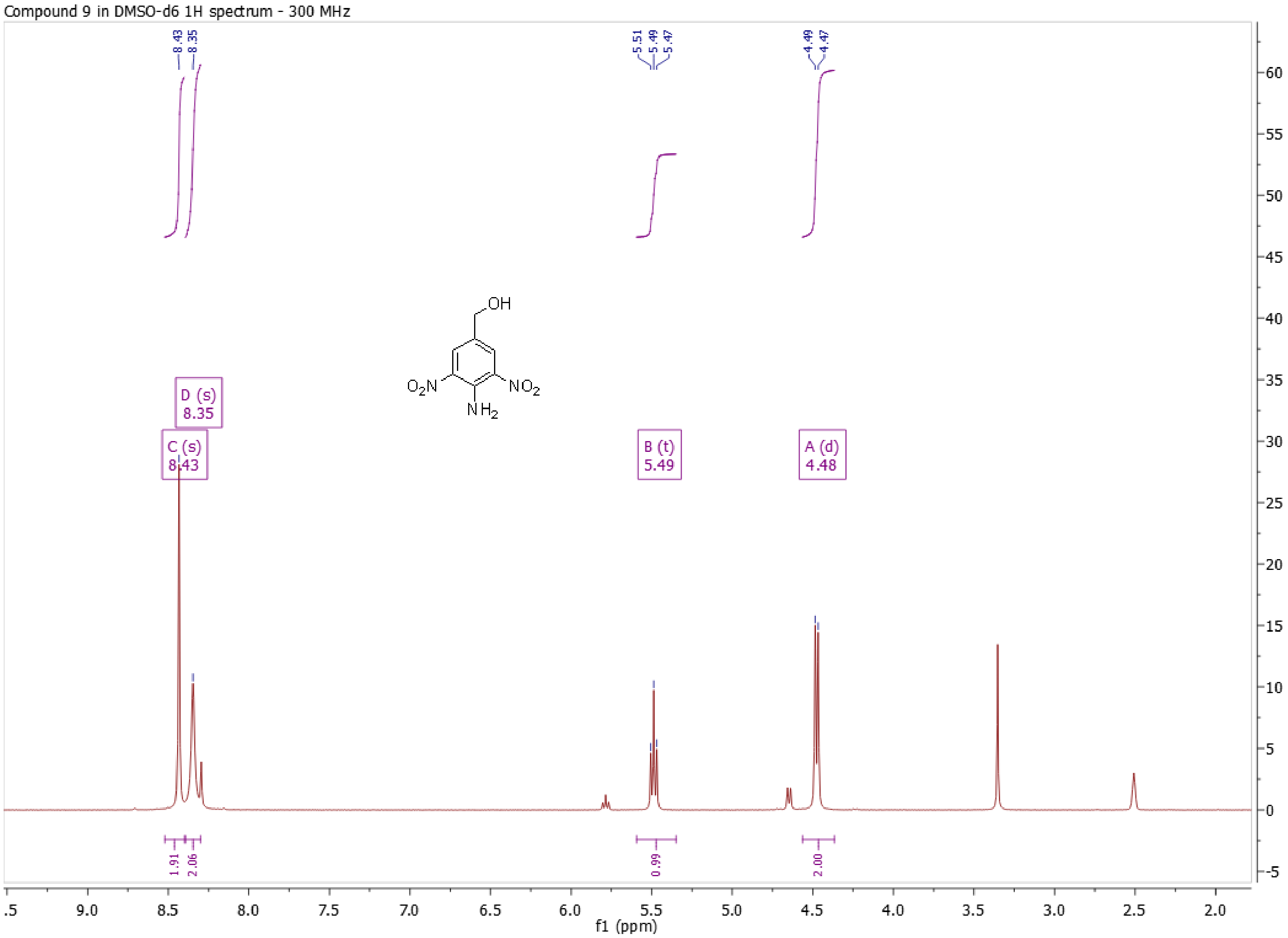
Compound 9 in DMSO-d6 1H spectrum - 300 MHz
8.43
8.35
5.51
5.49
5.47
4.49
4.47
OH
O_2N
NO_2
NH_2
D (s)
8.35
C (s)
8.43
B (t)
5.49
A (d)
4.48
1.91
2.06
0.99
2.00
f1 (ppm)

Compound 9 in DMSO-d6 13C spectrum - 75 MHz

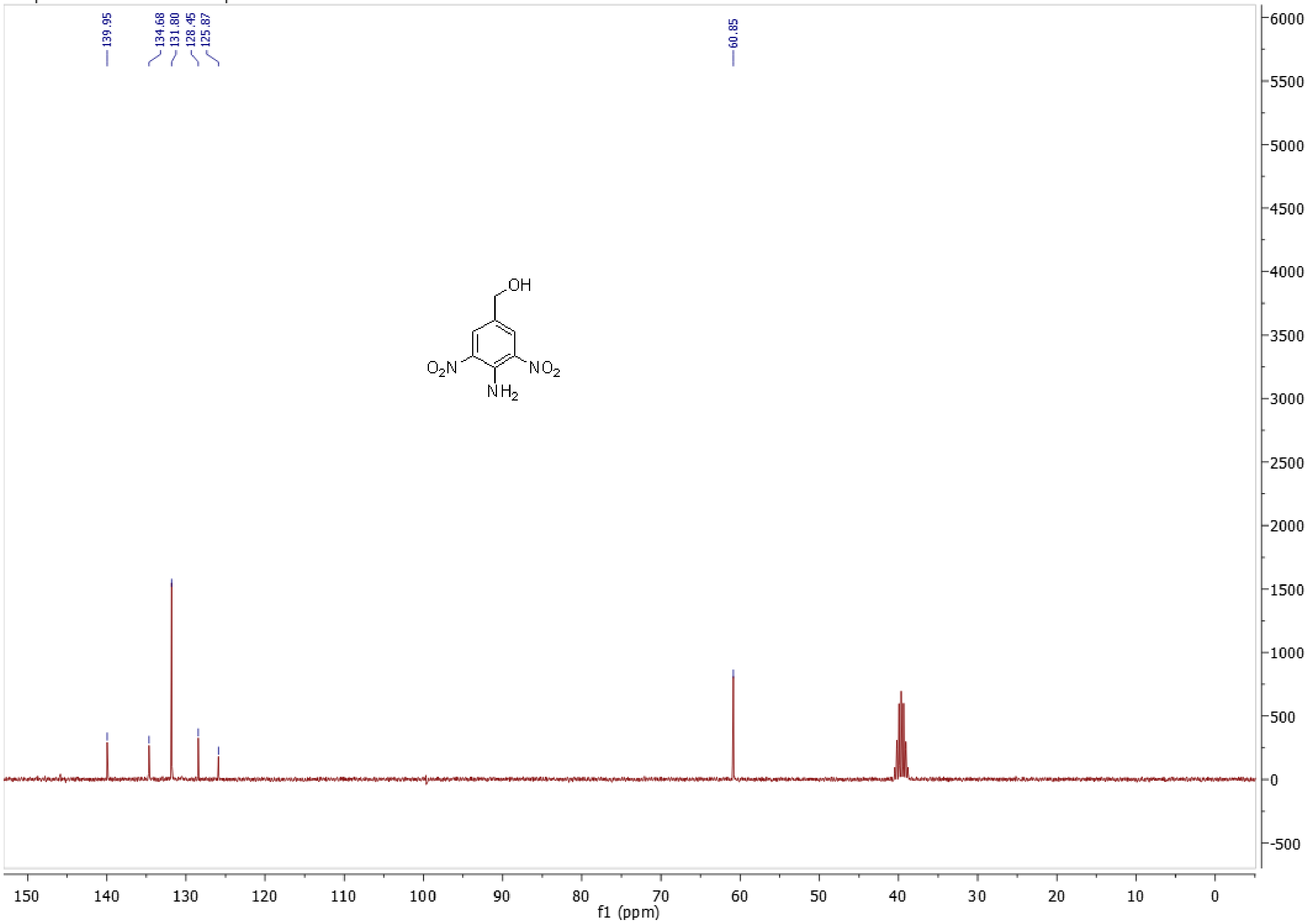

compound 9

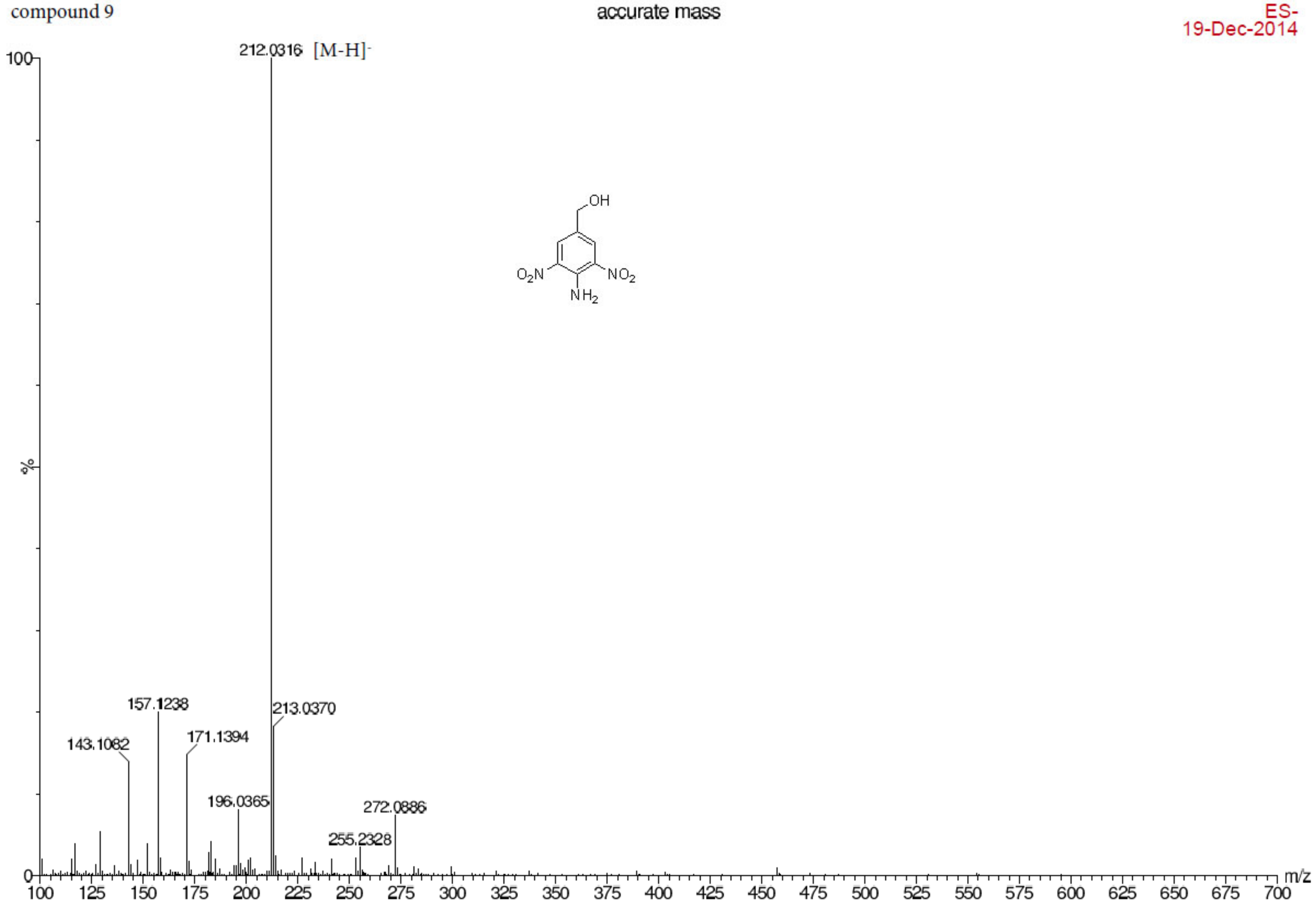

accurate mass
ES-
19-Dec-2014
212.0316 [M-H]-
100
%
0
157.1238
213.0370
143.1082
171.1394
196.0365
272.0886
255.2328
m/z
100 125 150 175 200 225 250 275 300 325 350 375 400 425 450 475 500 525 550 575 600 625 650 675 700
OH
O_2N
NO_2
NH_2

Compound 10 in DMSO-d6 1H spectrum - 300 MHz

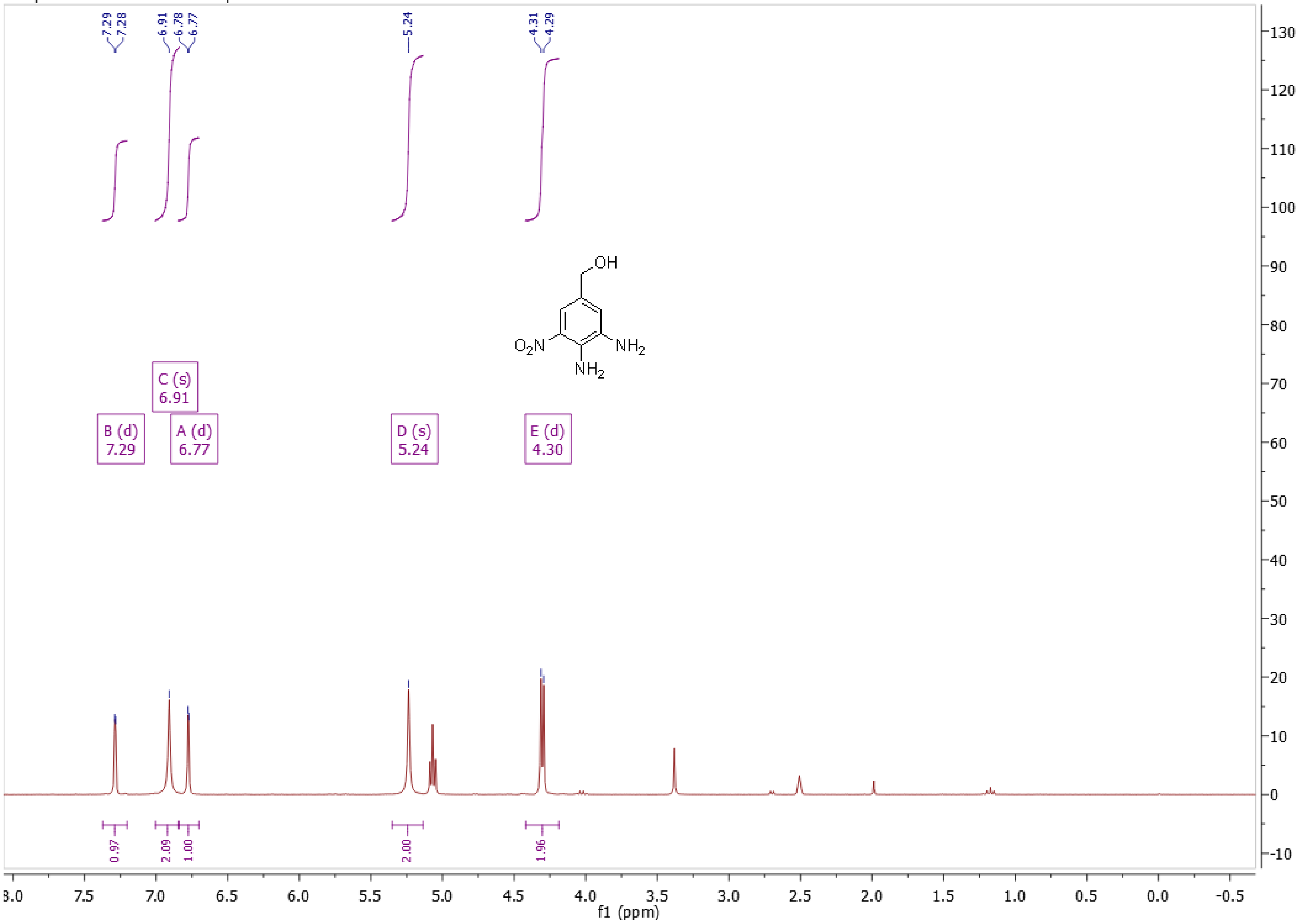

Compound 10 in DMSO-d6 13C spectrum - 75 MHz

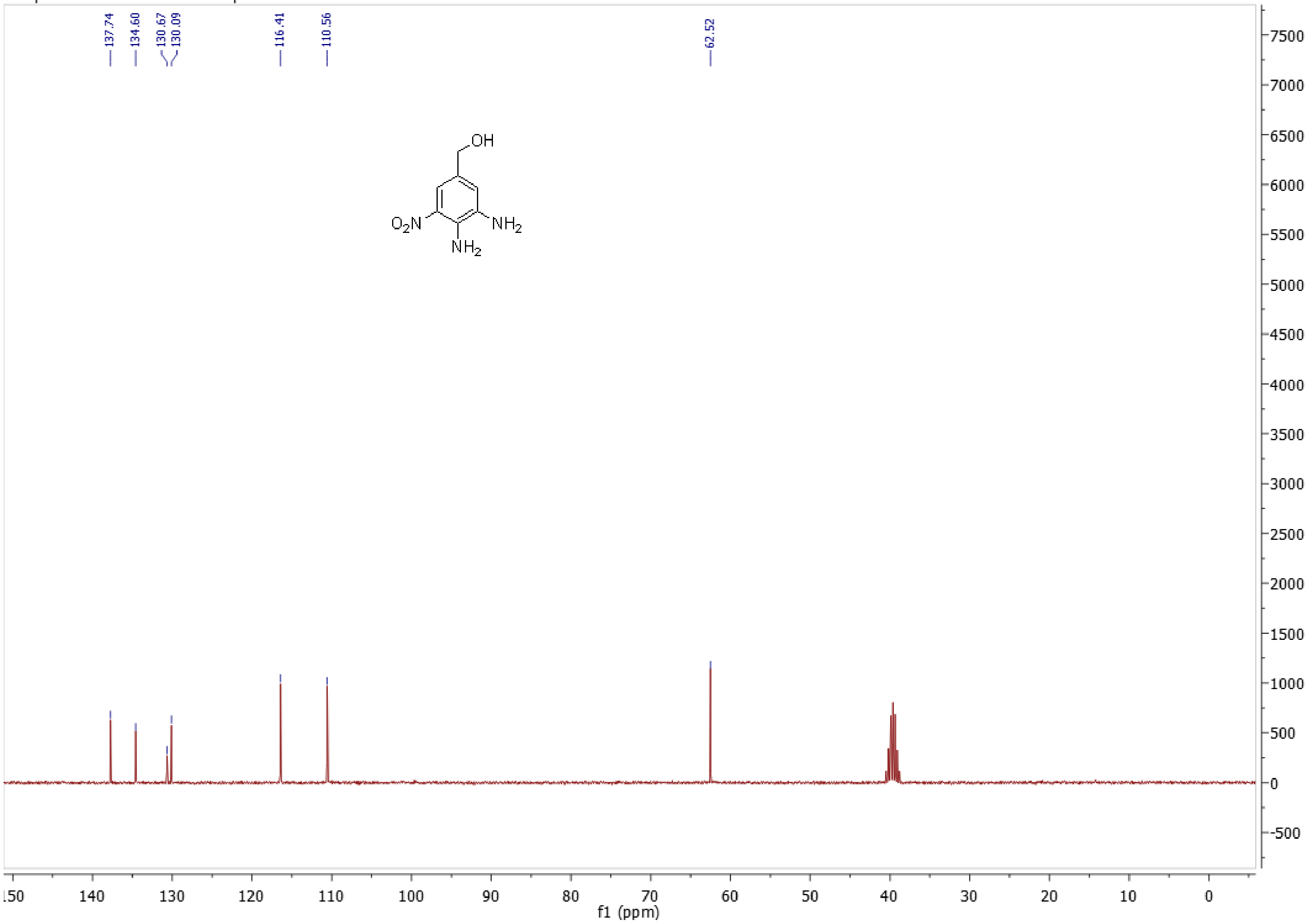

compound 10

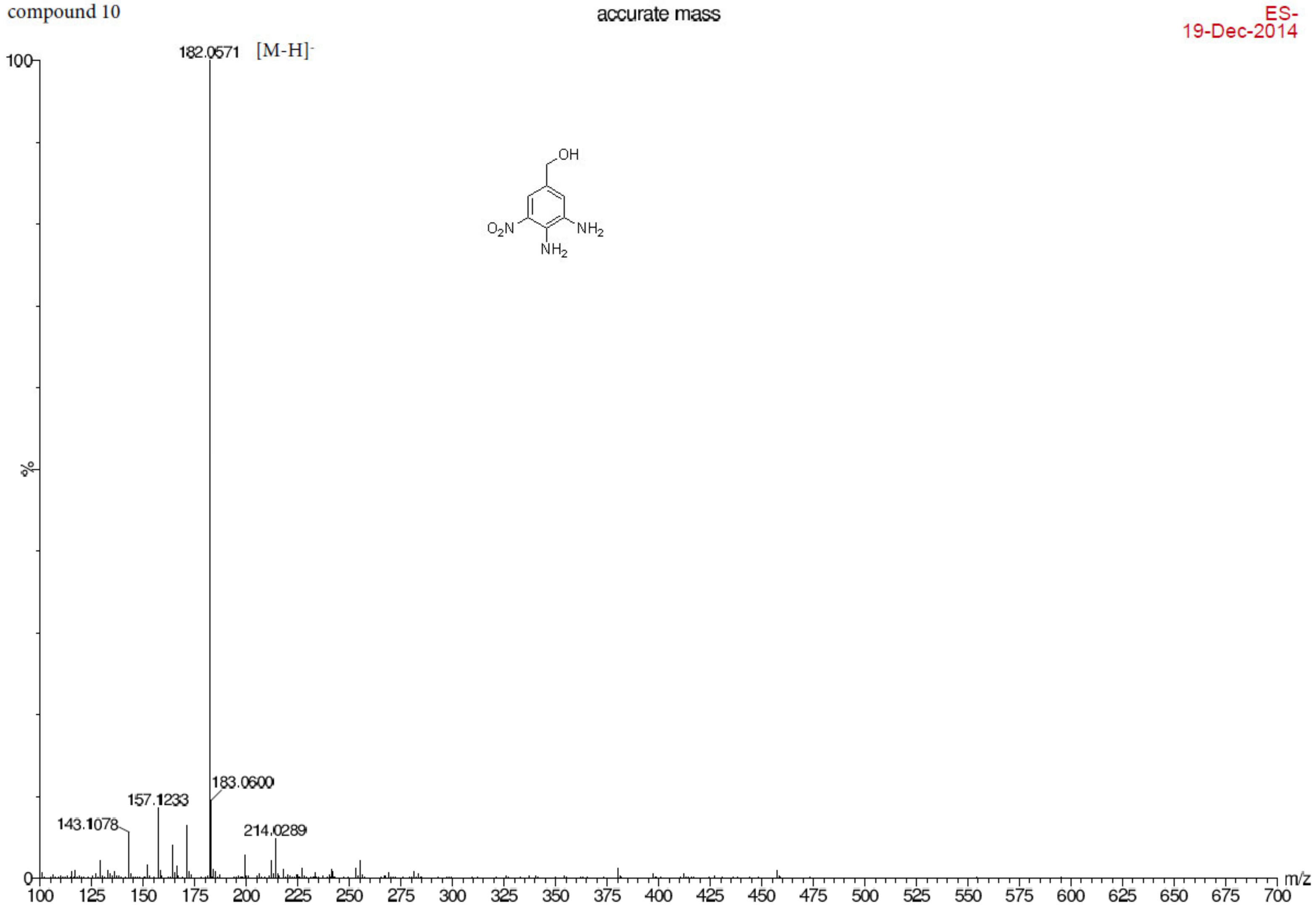

accurate mass
ES-
19-Dec-2014
182.0571
[M-H]-
100
%
0
183.0600
157.1233
143.1078
214.0289
OH
O_2N
NH_2
NH_2
100
125
150
175
200
225
250
275
300
325
350
375
400
425
450
475
500
525
550
575
600
625
650
675
700
m/z

Compound 11 in DMSO-d6 1H spectrum - 300 MHz

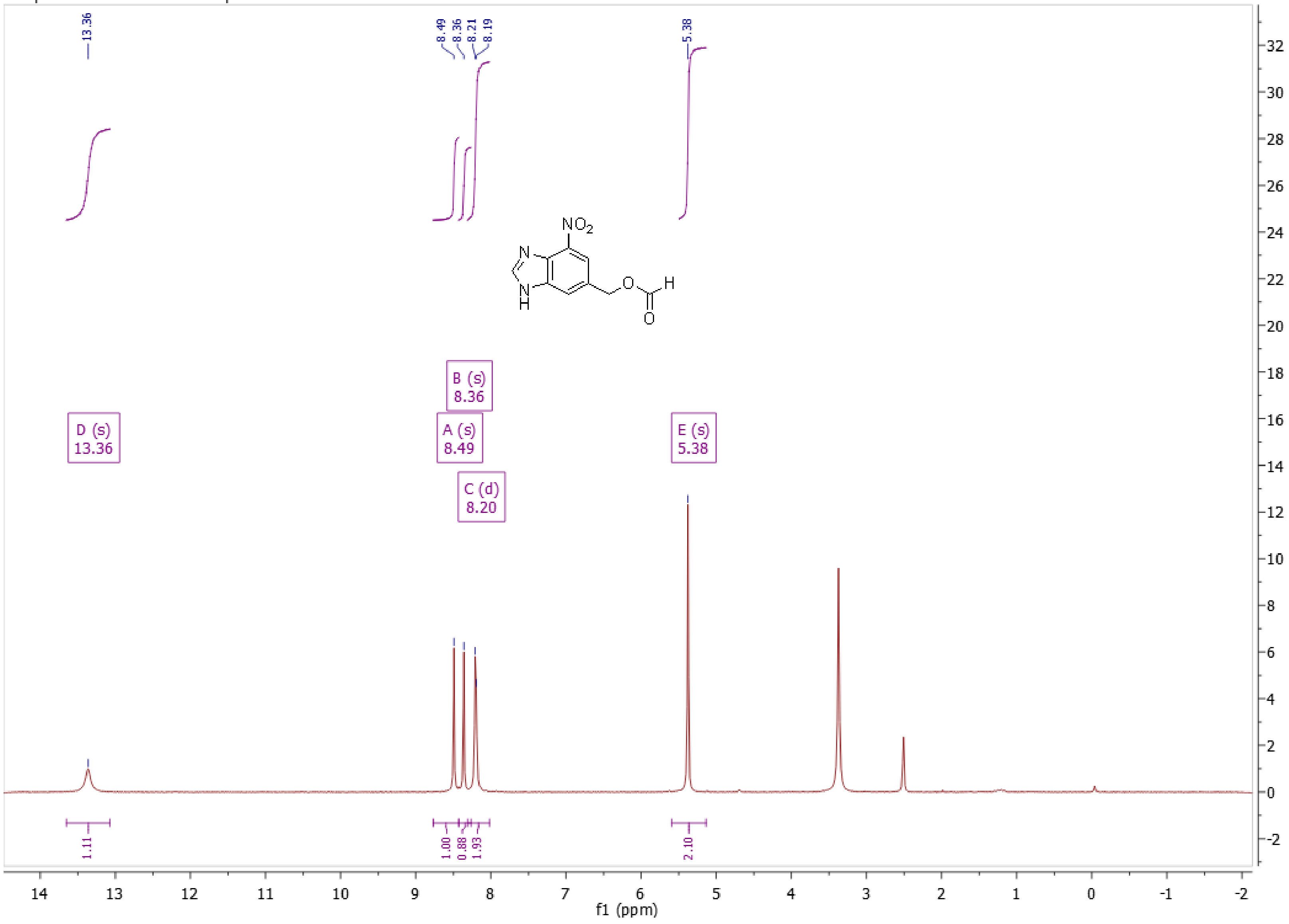

Compound 11 in DMSO-d6 13C spectrum - 75 MHz

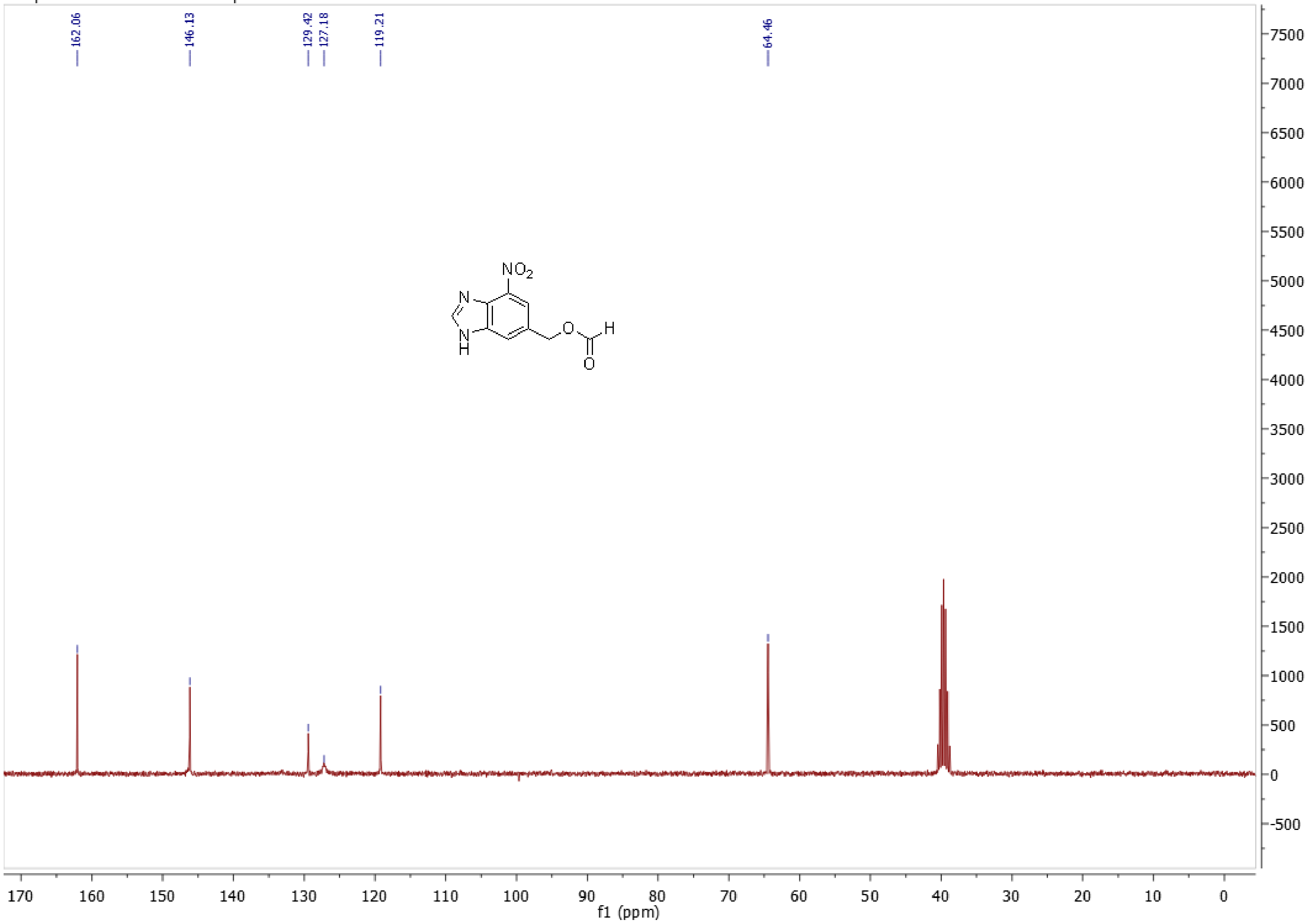

compound 11

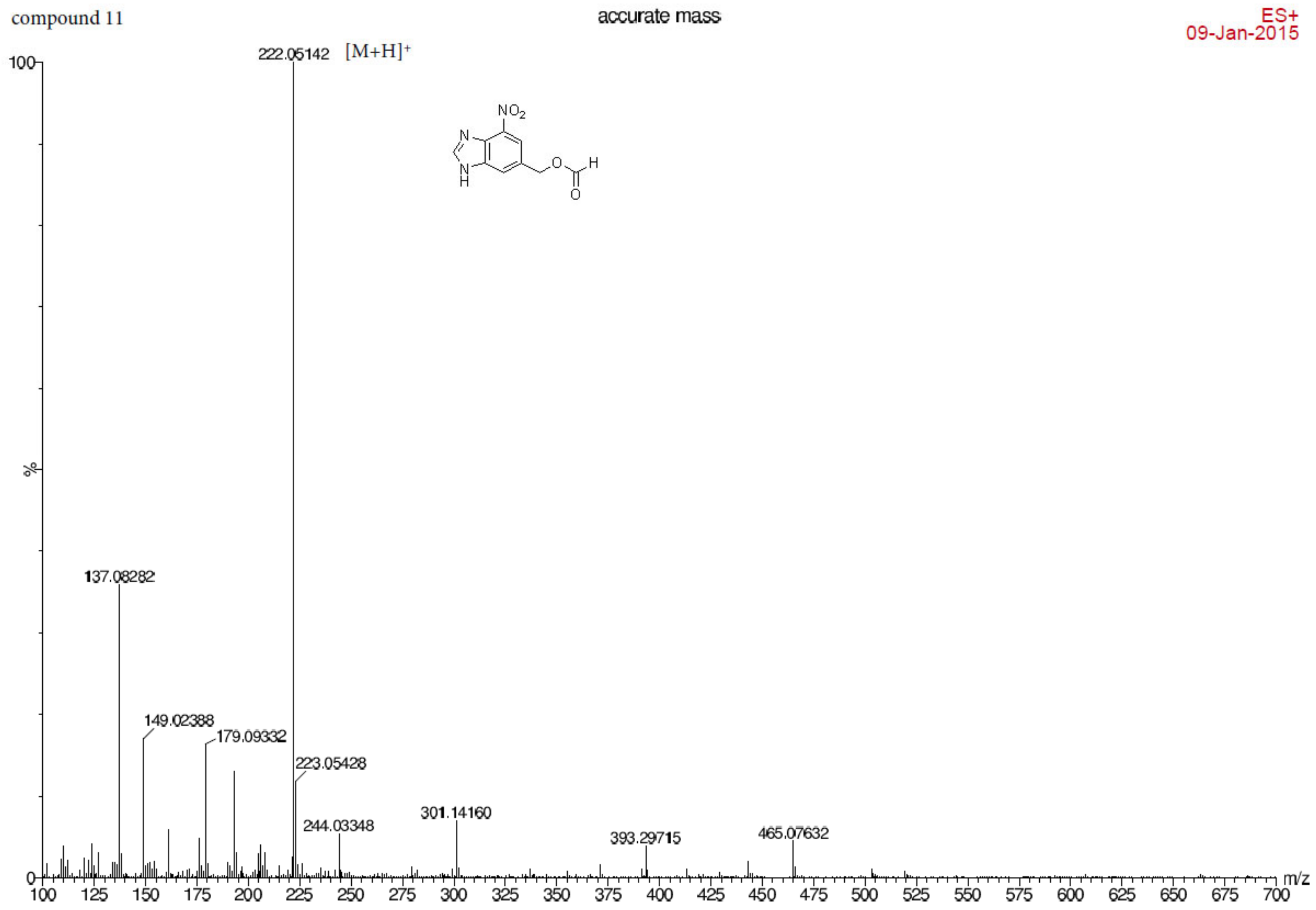
accurate mass
ES+
09-Jan-2015
222.05142 [M+H]+
100
%
0
137.08282
149.02388
179.09332
223.05428
244.03348
301.14160
393.29715
465.07632
NO2
N
N
H
O
H
O
100 125 150 175 200 225 250 275 300 325 350 375 400 425 450 475 500 525 550 575 600 625 650 675 700
m/z

Compound 12 in DMSO-d6 1H spectrum - 300 MHz

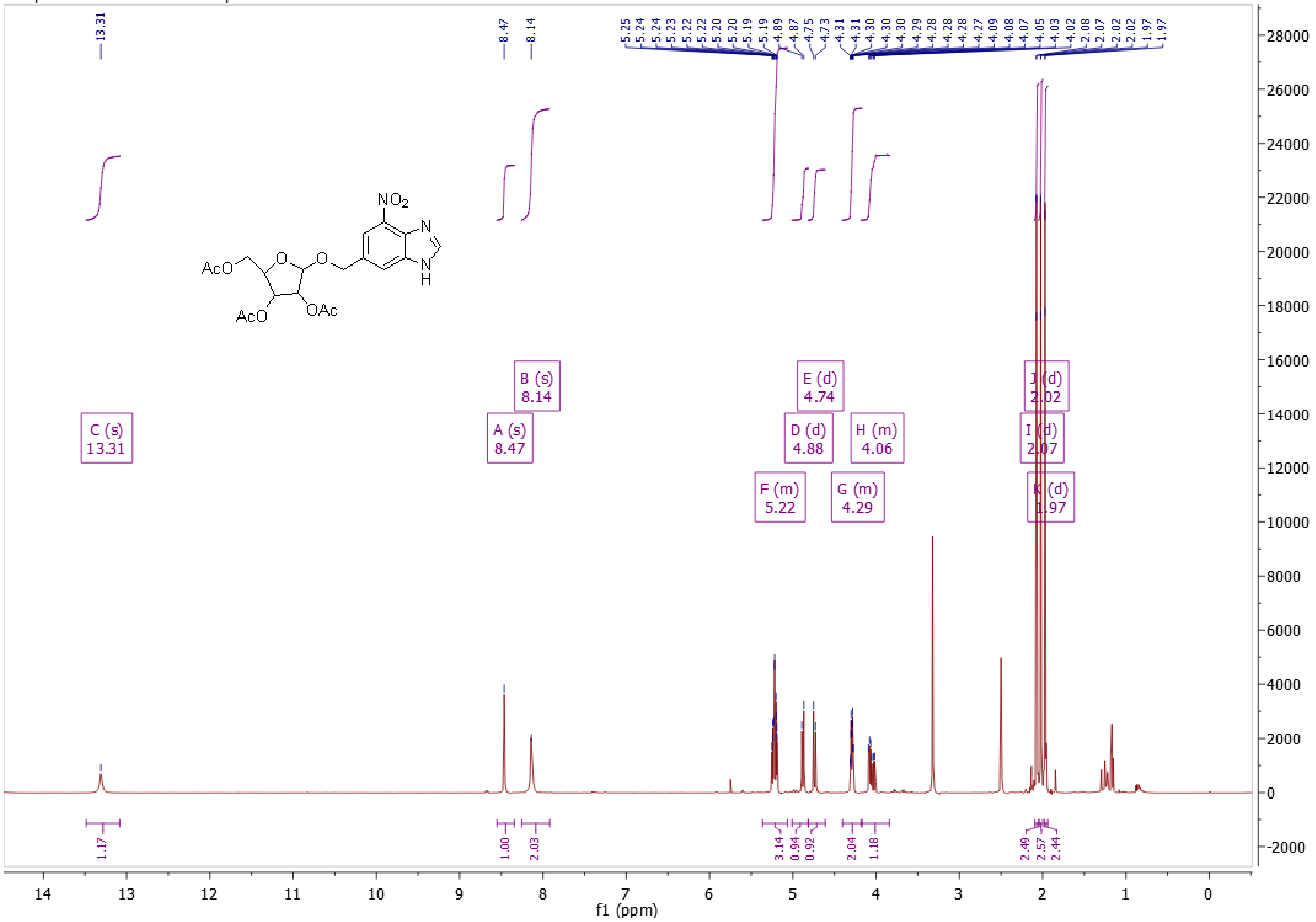

Compound 12 in DMSO-d6 13C spectrum - 75 MHz

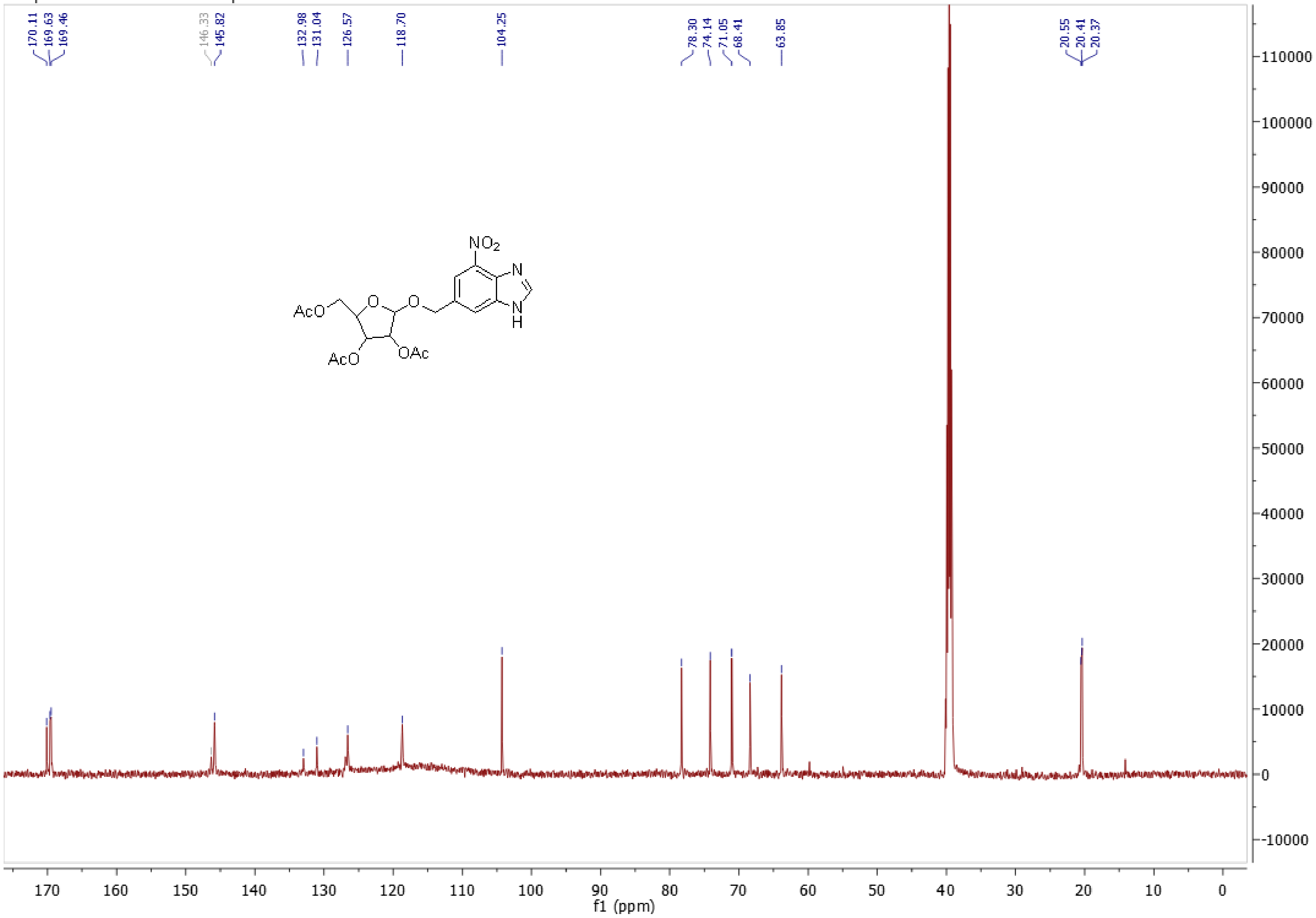

compound 12

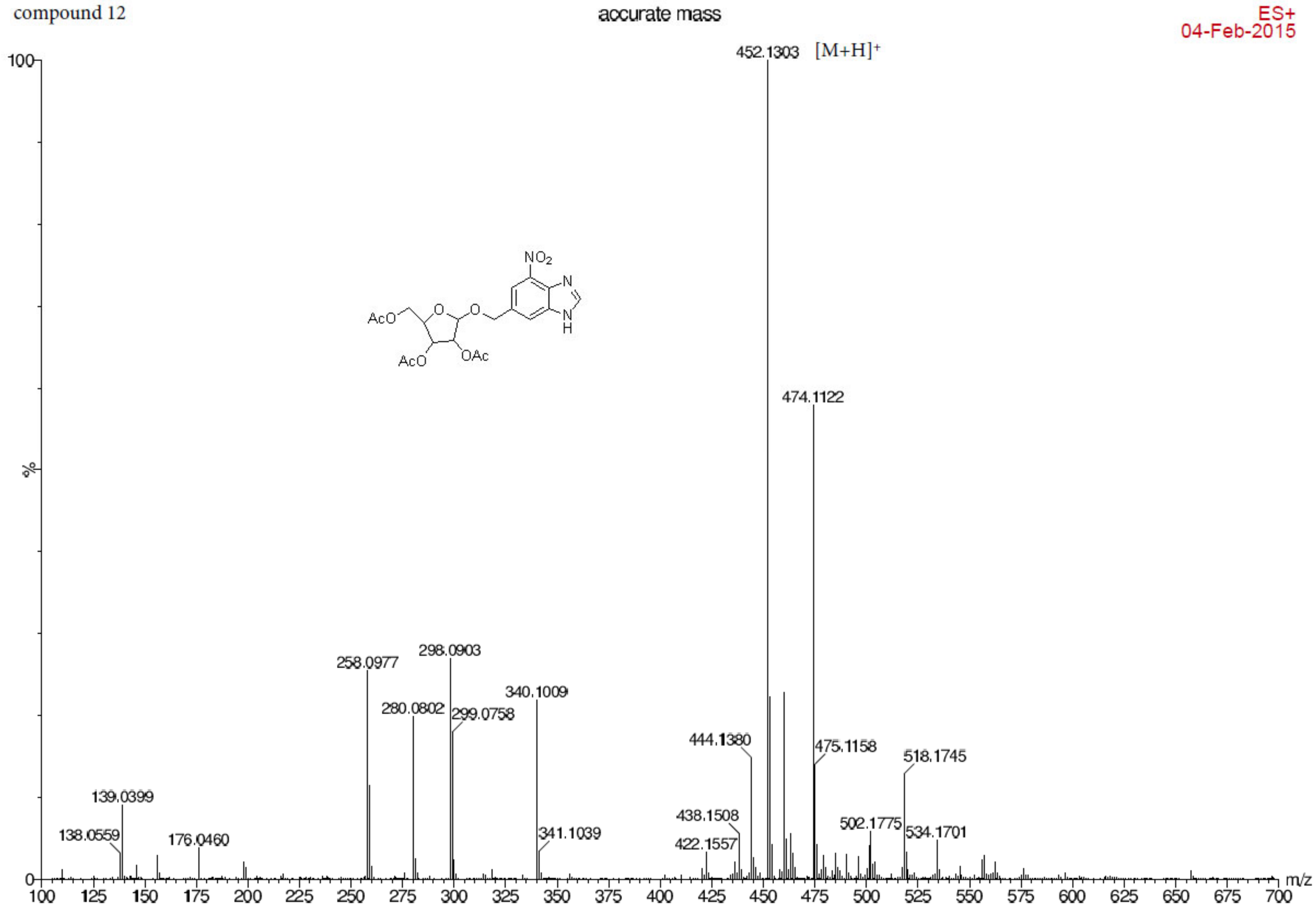
accurate mass
ES+
04-Feb-2015
452.1303 [M+H]+
474.1122
258.0977
298.0903
340.1009
280.0802
299.0758
444.1380
475.1158
518.1745
139.0399
438.1508
502.1775
534.1701
138.0559
176.0460
341.1039
422.1557
NO2
N
N
H
O
O
AcO
AcO
OAc
%
m/z
100
0

Compound 13 in DMSO-d6 1H spectrum - 300 MHz

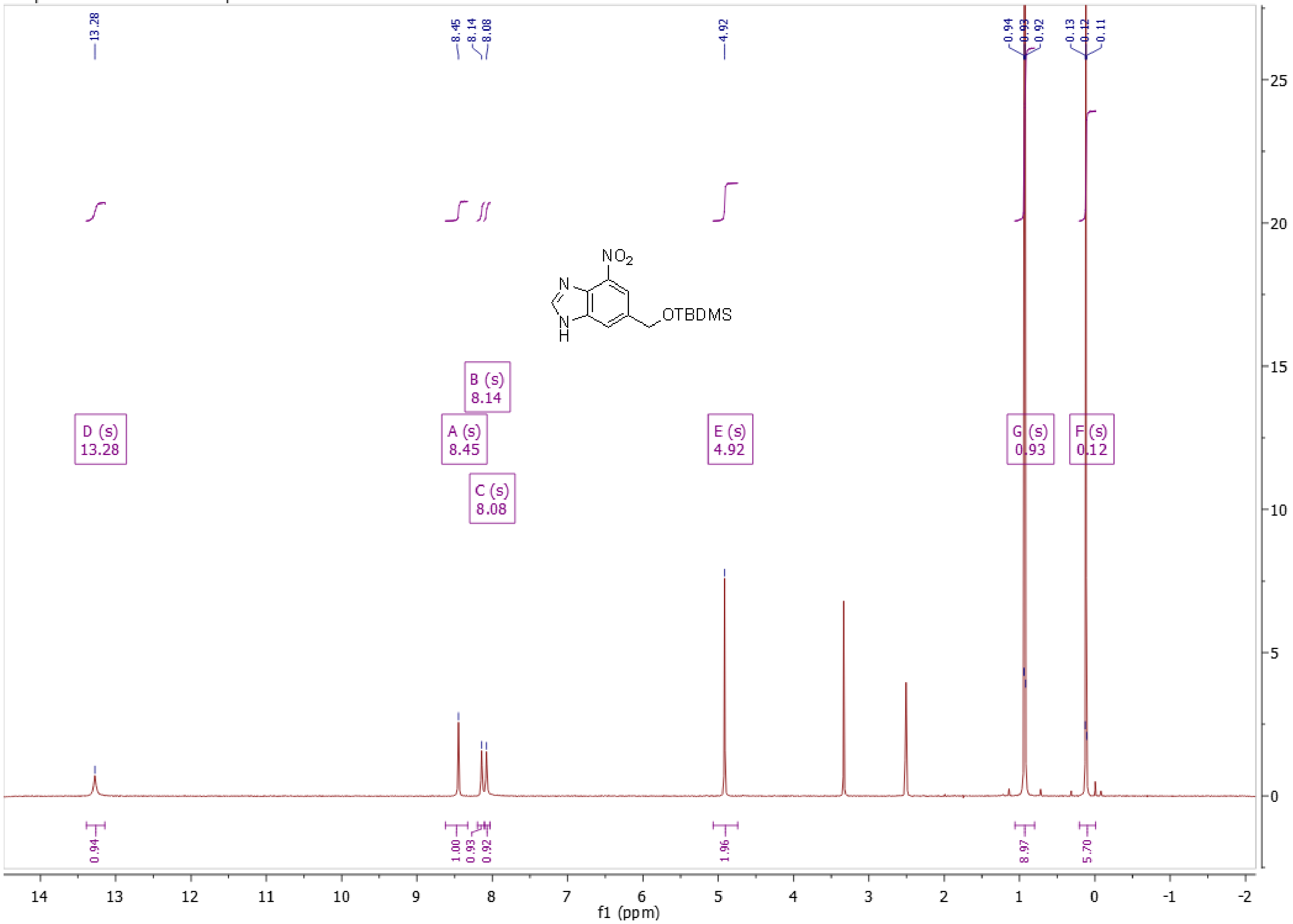

Compound 13 in DMSO-d6 13C spectrum - 75 MHz

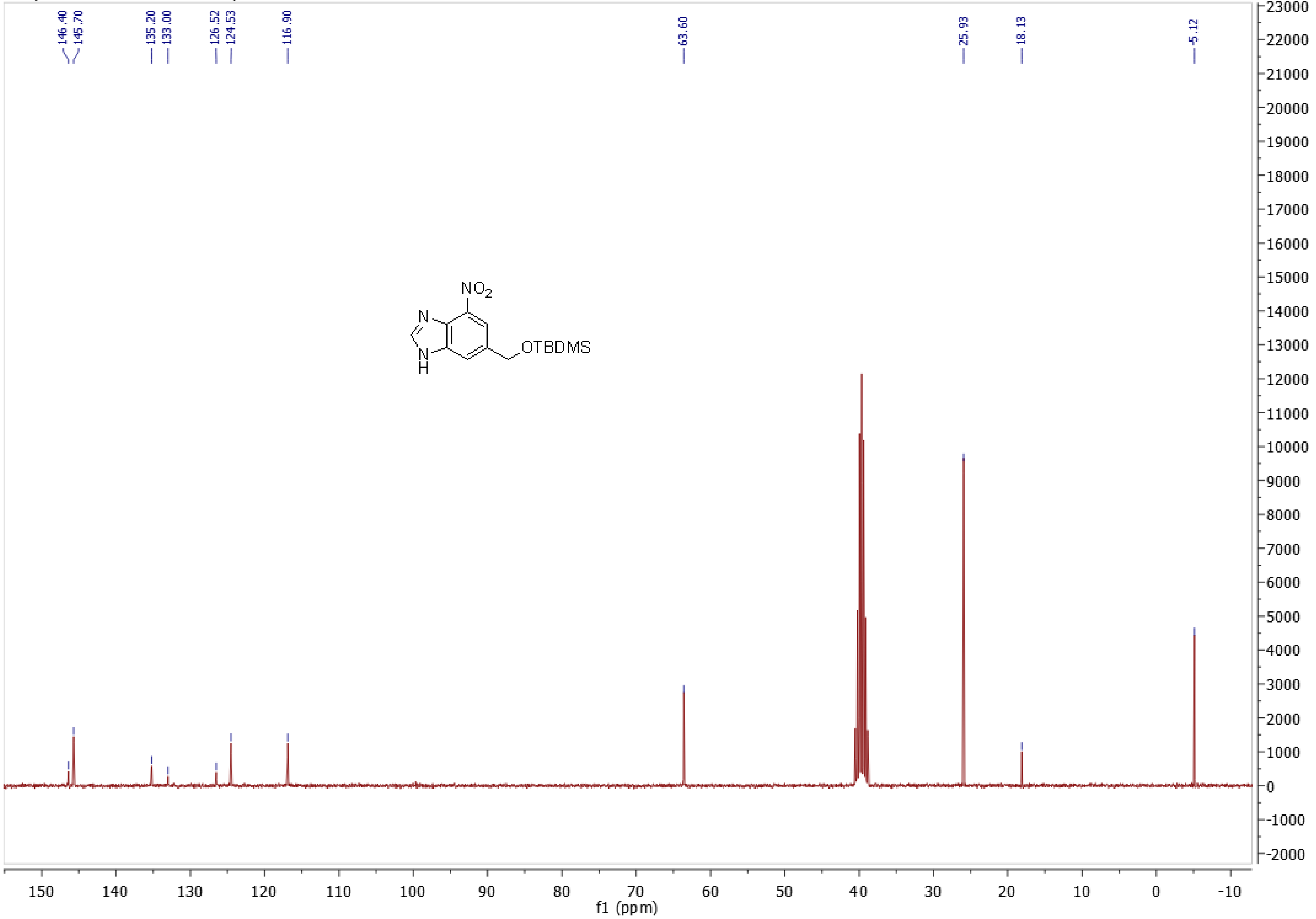

compound 13

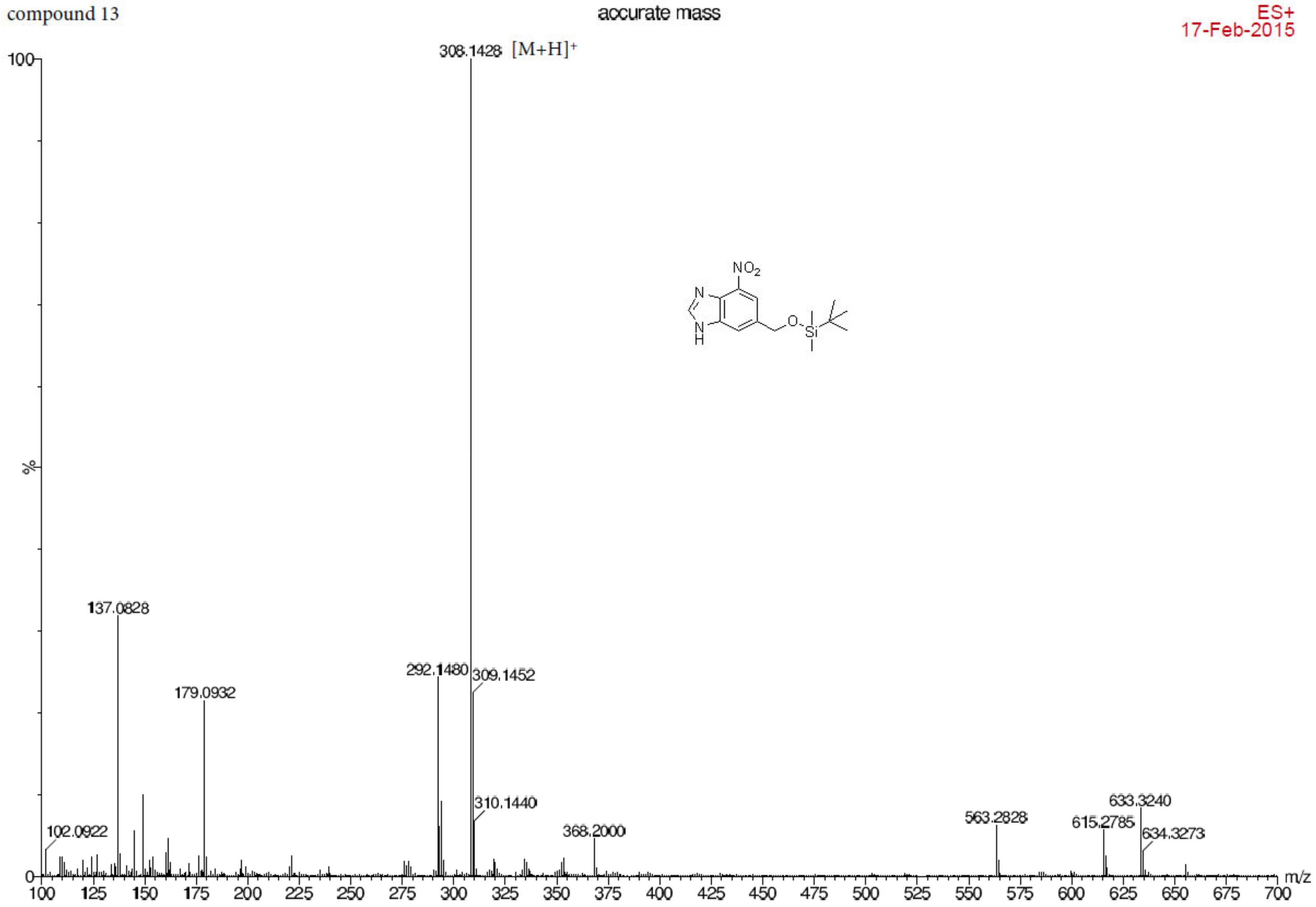
accurate mass
ES+
17-Feb-2015
308.1428 [M+H]+
137.0828
179.0932
292.1480
309.1452
310.1440
102.0922
368.2000
563.2828
615.2785
633.3240
634.3273
%
m/z
NO2
N
N
H
O
Si

Compound 14 in DMSO-d6 1H spectrum - 300 MHz

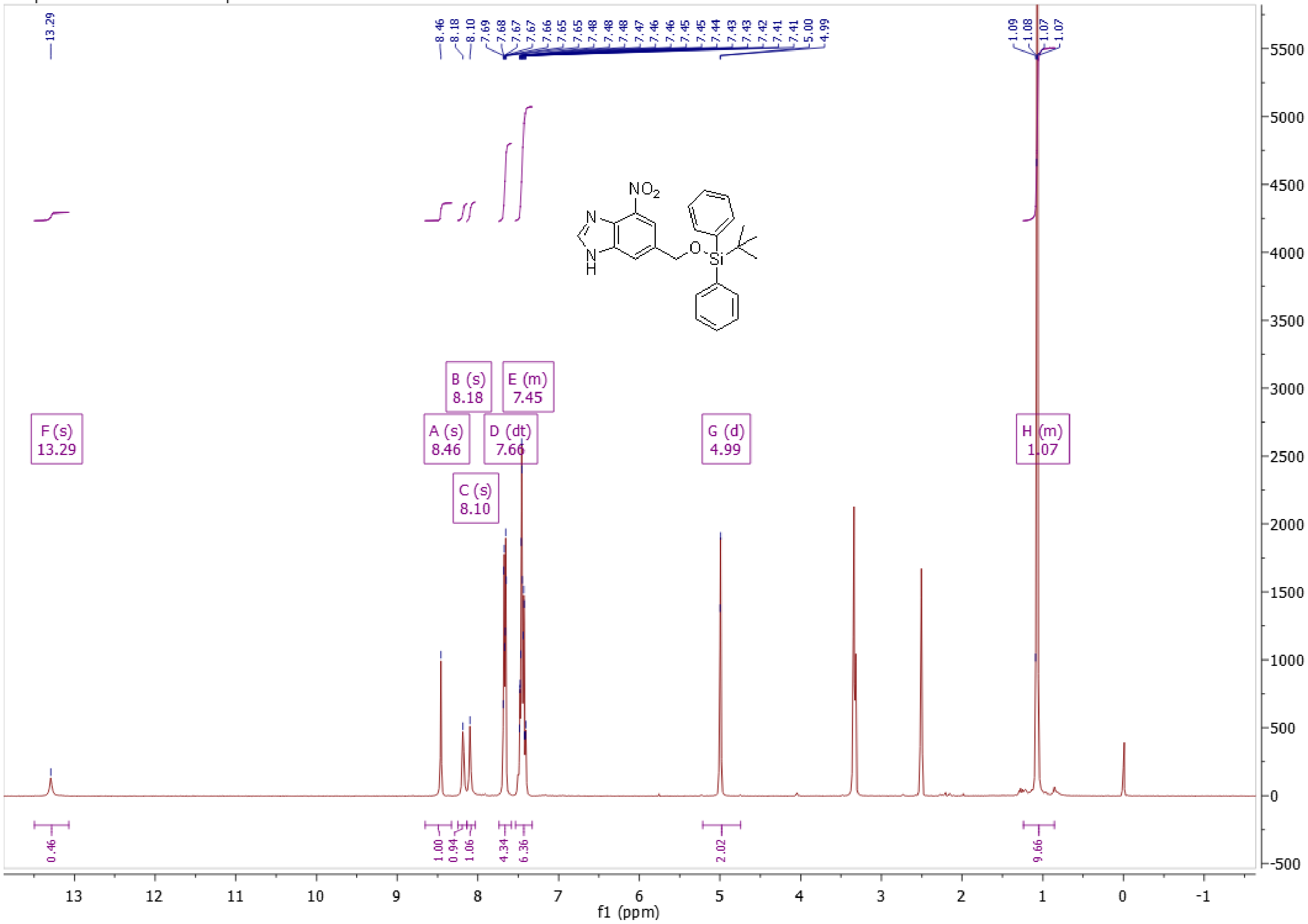

Compound 14 in DMSO-d6 13C spectrum - 75 MHz

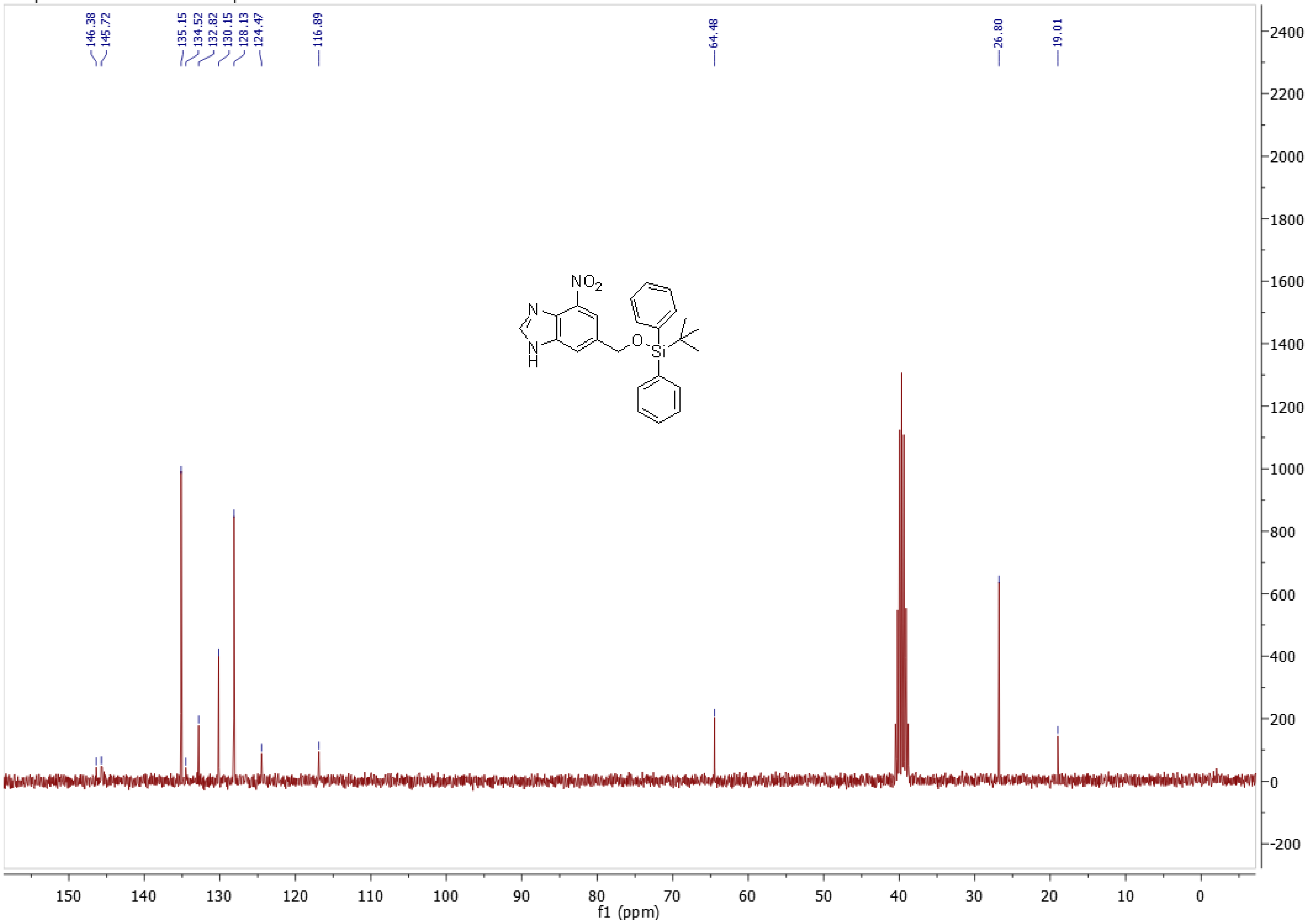

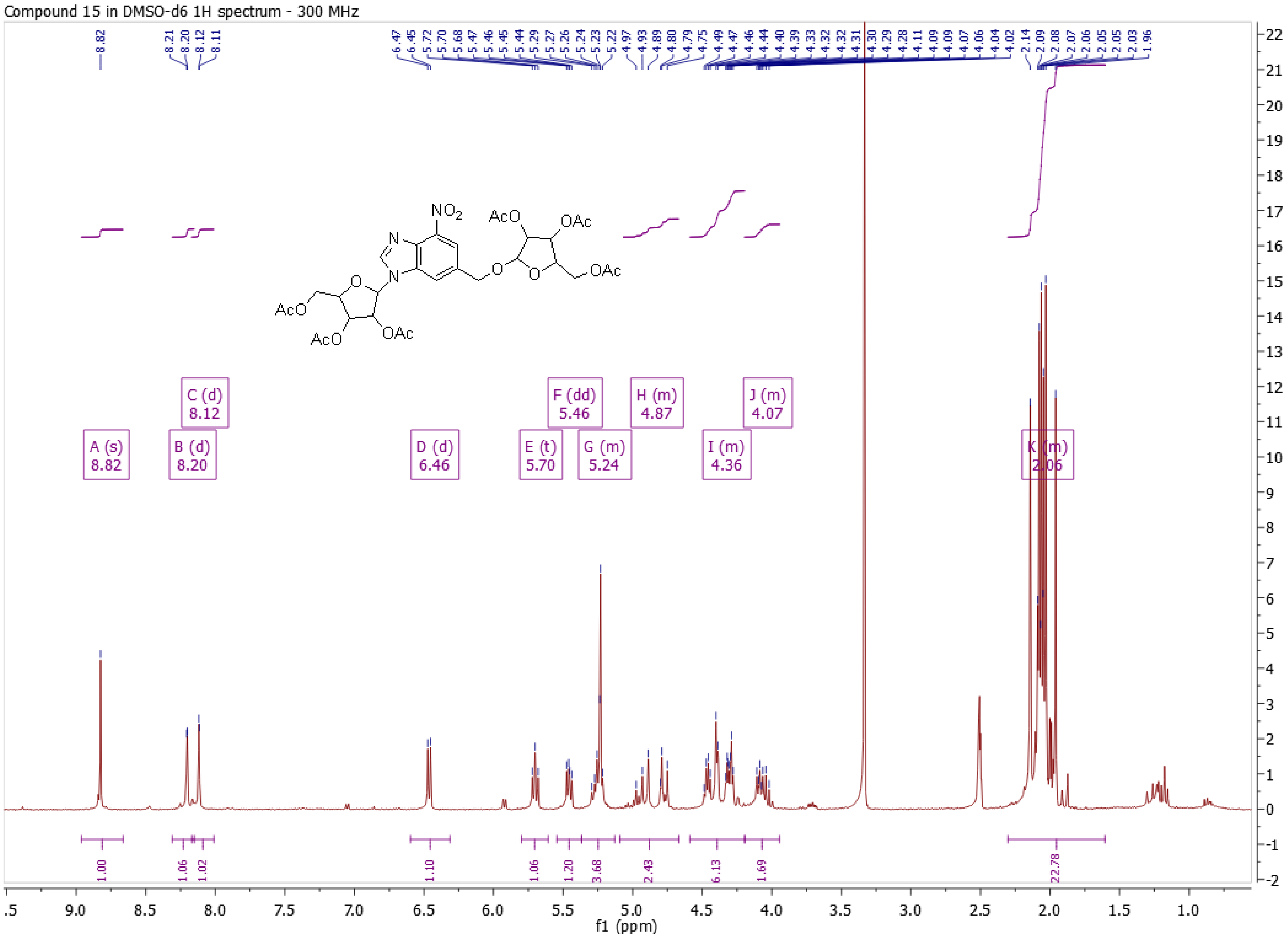
Compound 15 in DMSO-d6 1H spectrum - 300 MHz
A (s) 8.82
B (d) 8.20
C (d) 8.12
D (d) 6.46
E (t) 5.70
F (dd) 5.46
G (m) 5.24
H (m) 4.87
I (m) 4.36
J (m) 4.07
K (m) 2.06
1.00
1.06
1.02
1.10
1.06
1.20
3.68
2.43
6.13
1.69
22.78
f1 (ppm)
NO2
AcO
OAc
OAc
AcO
AcO
OAc
N
N
O
O
O

Compound 15 in DMSO-d6 13C spectrum - 75 MHz

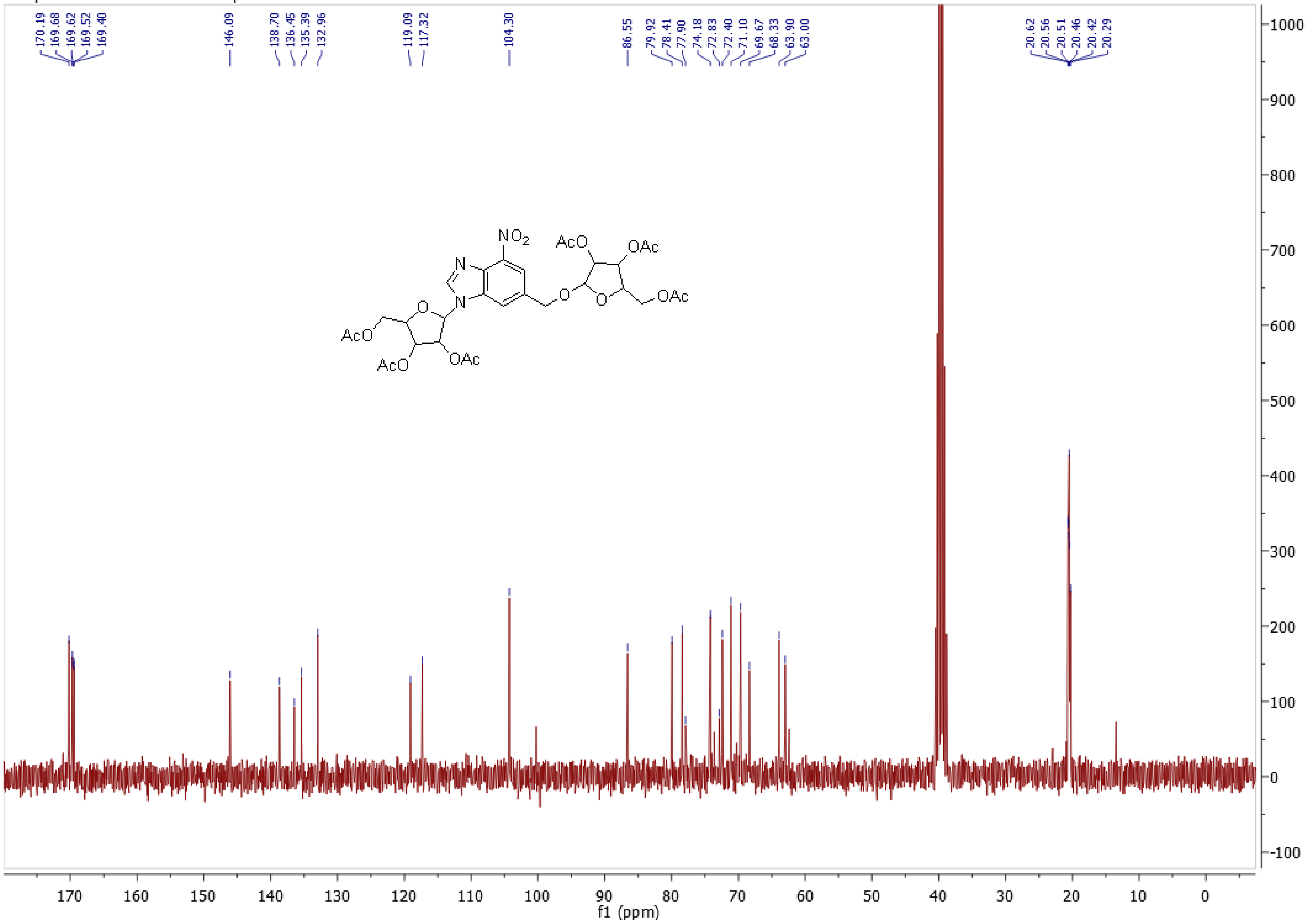

Compound 16 in CDCl3 1H spectrum - 300 MHz

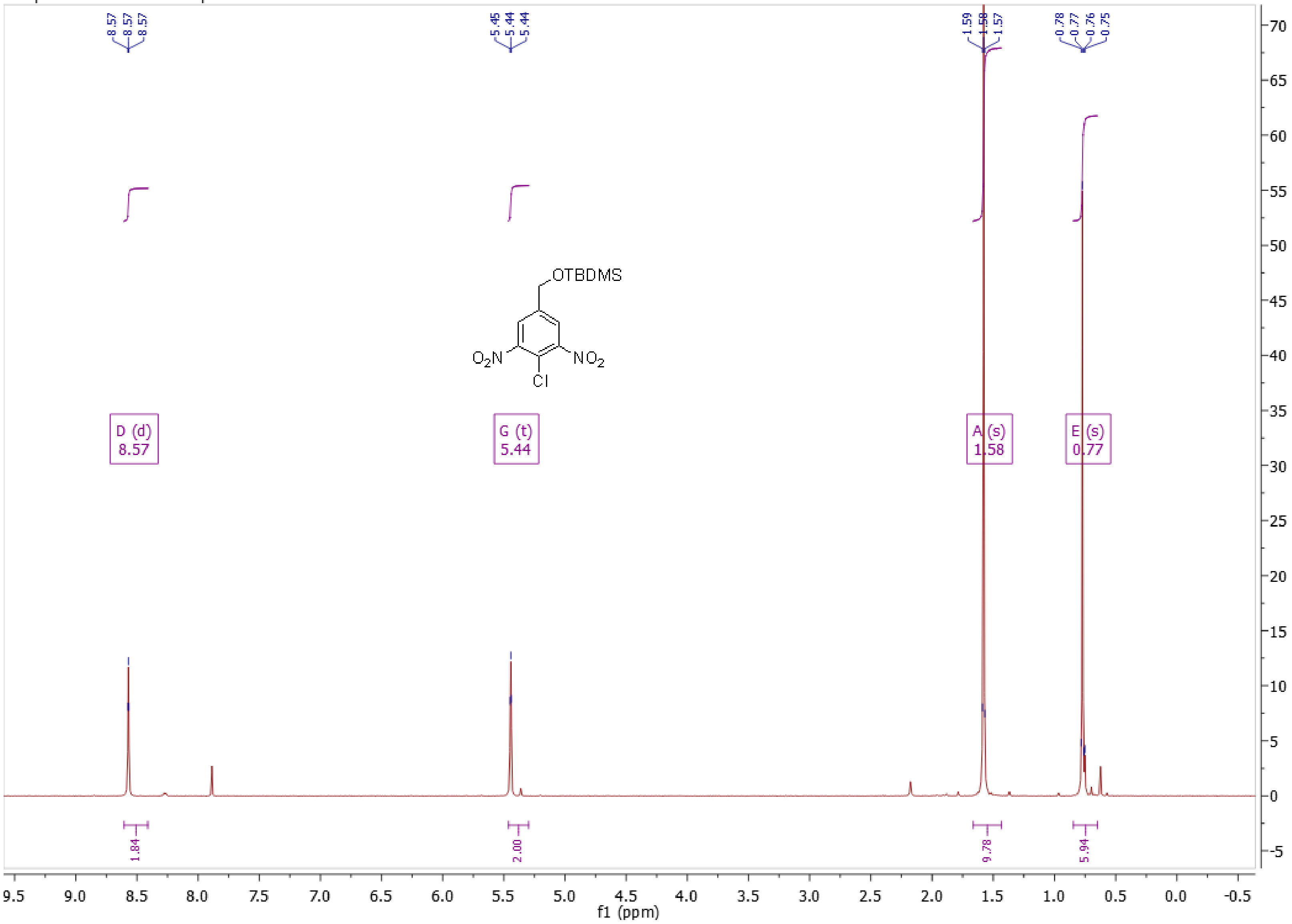

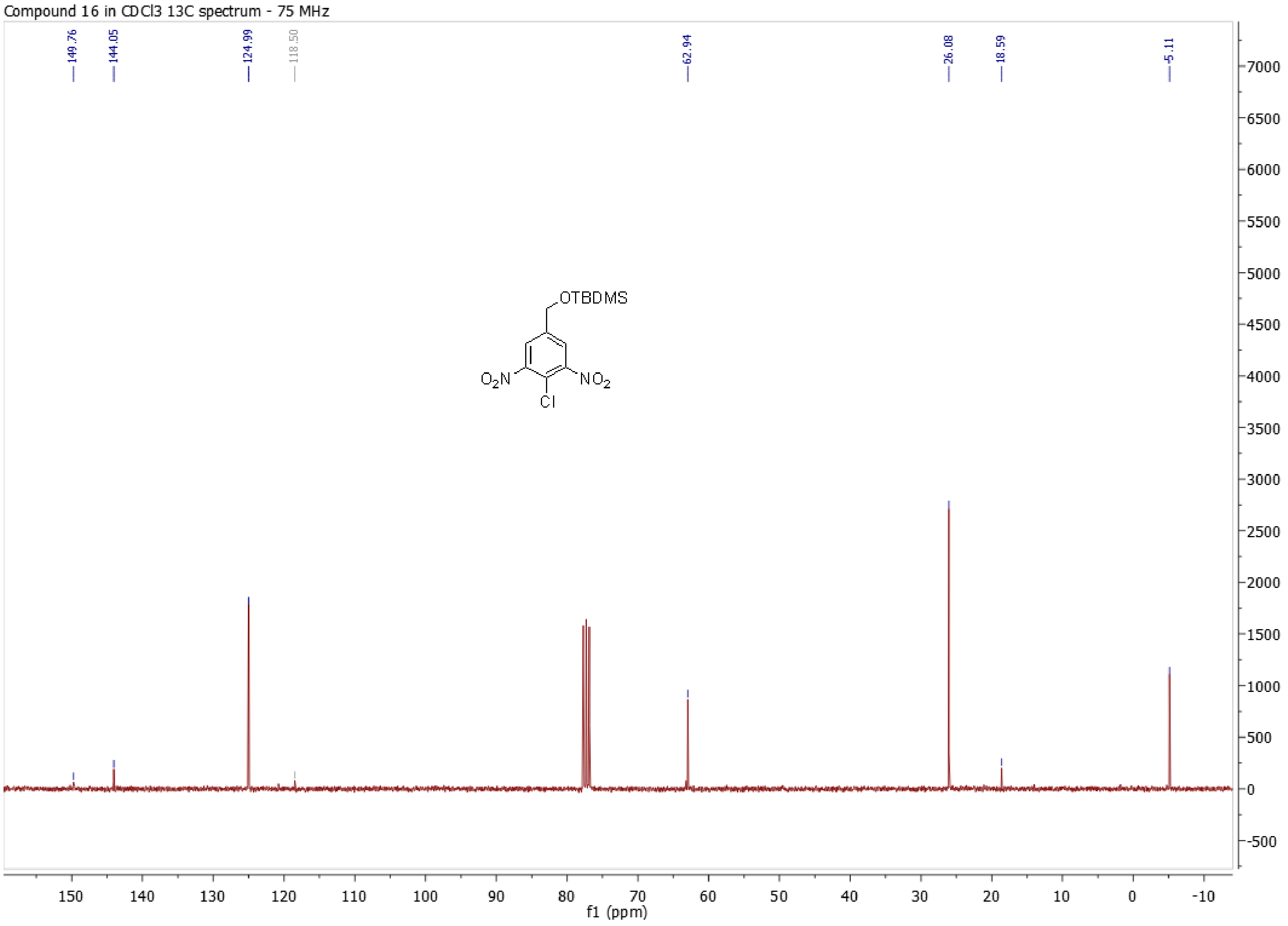
Compound 16 in CDCl3 13C spectrum - 75 MHz
149.76
144.05
124.99
118.50
62.94
26.08
18.59
-5.11
OTBDMS
O2N
NO2
Cl
7000
6500
6000
5500
5000
4500
4000
3500
3000
2500
2000
1500
1000
500
0
-500
150
140
130
120
110
100
90
80
70
60
50
40
30
20
10
0
-10
f1 (ppm)

Compound 17 in DMSO-d6 1H spectrum - 300 MHz

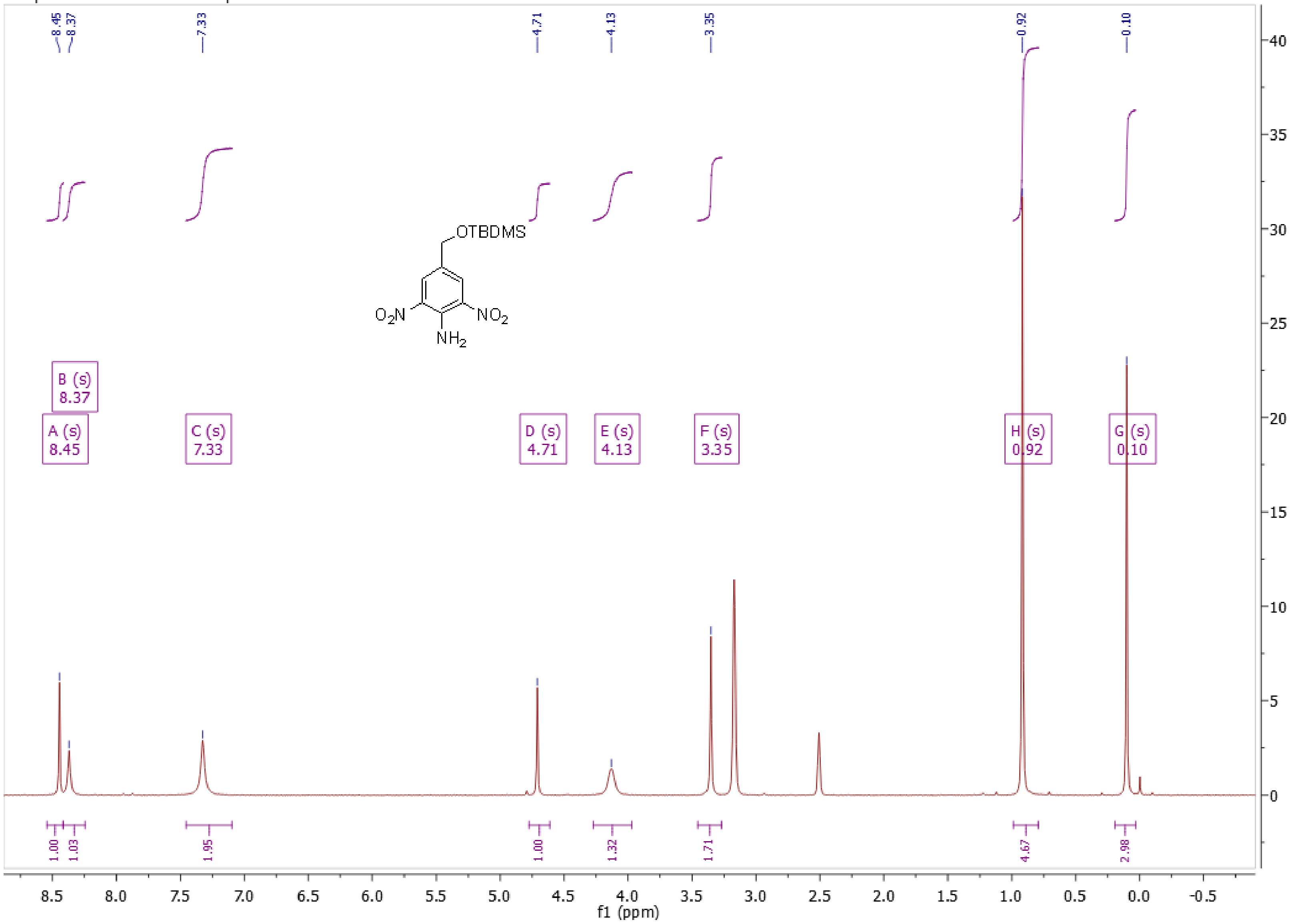

Compound 17 in DMSO-d6 13C spectrum - 75 MHz

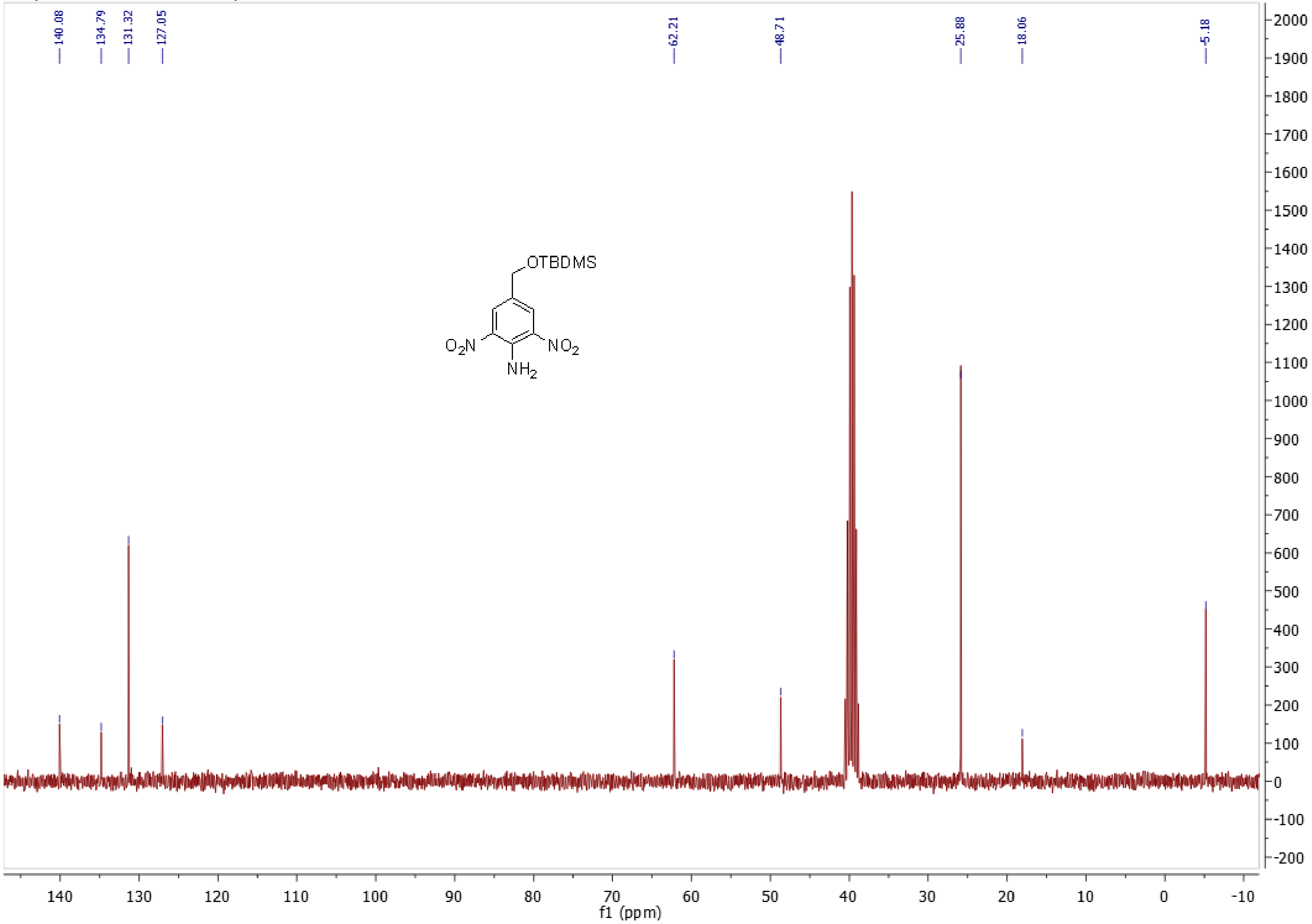

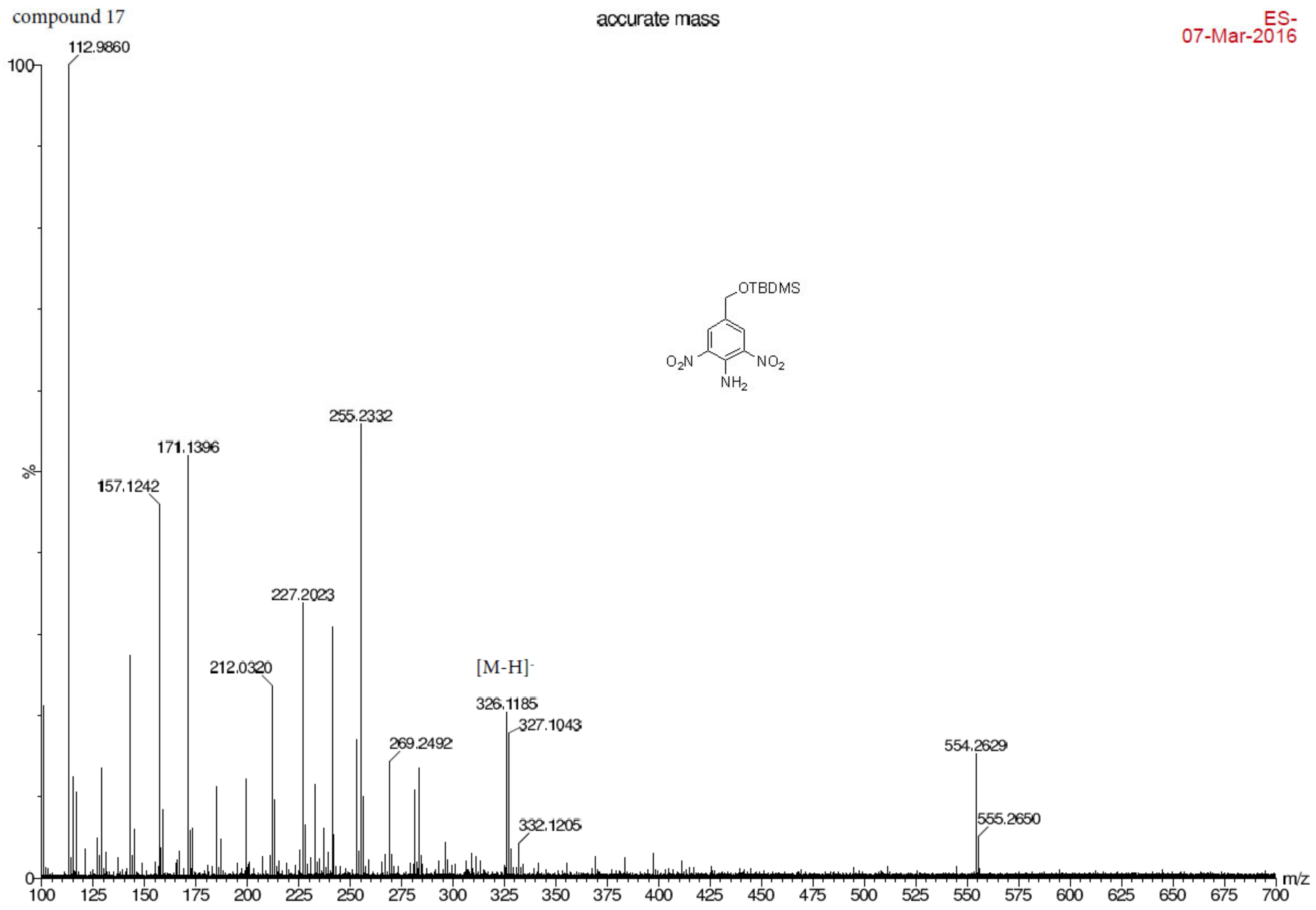

compound 17
accurate mass
ES-
07-Mar-2016
112.9860
157.1242
171.1396
255.2332
227.2023
212.0320
[M-H]-
326.1185
327.1043
269.2492
332.1205
554.2629
555.2650
OTBDMS
O2N
NO2
NH2
%
m/z

Compound 18 in DMSO-d6 1H spectrum - 300 MHz

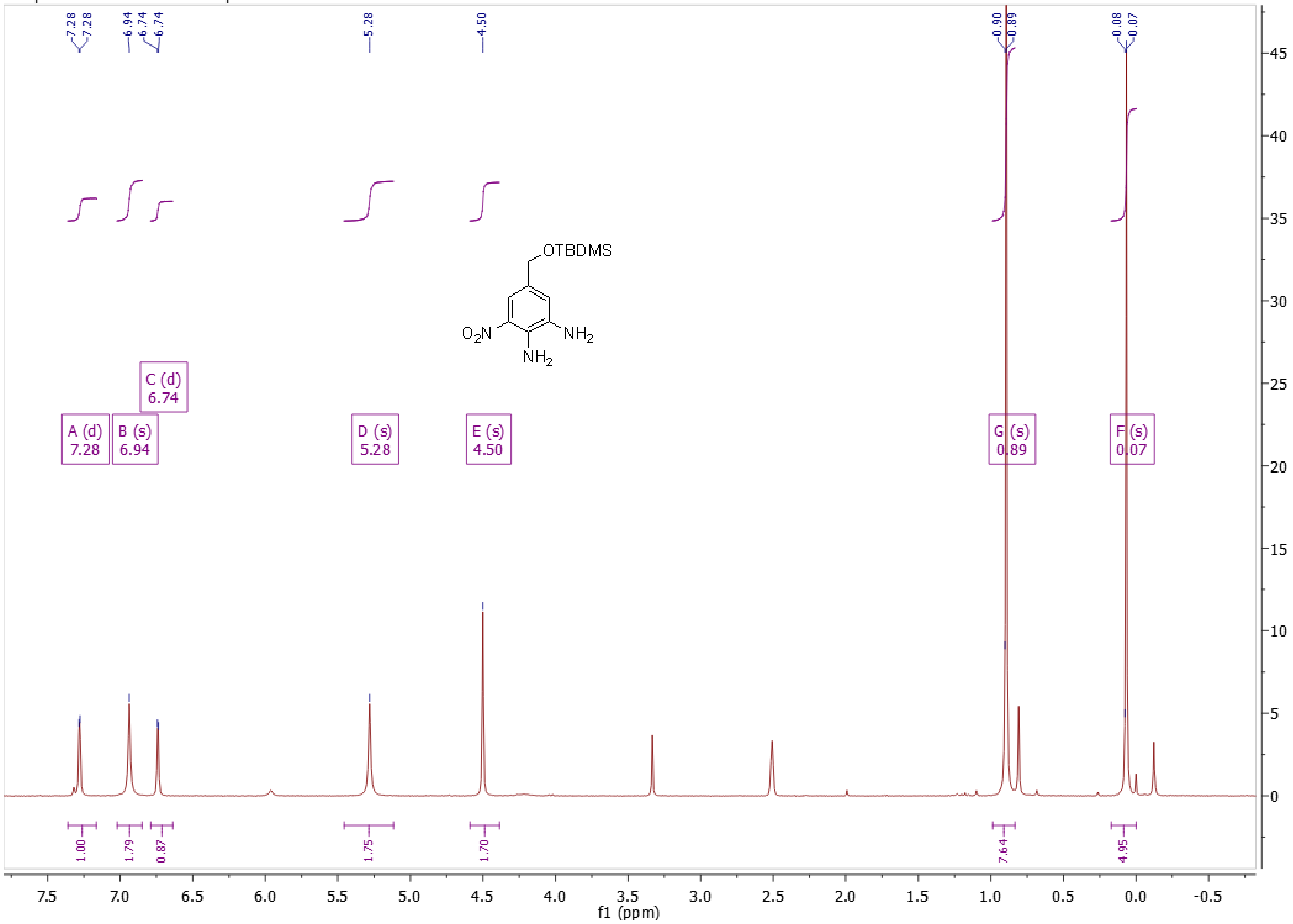

Compound 18 in DMSO-d6 13C spectrum - 75 MHz

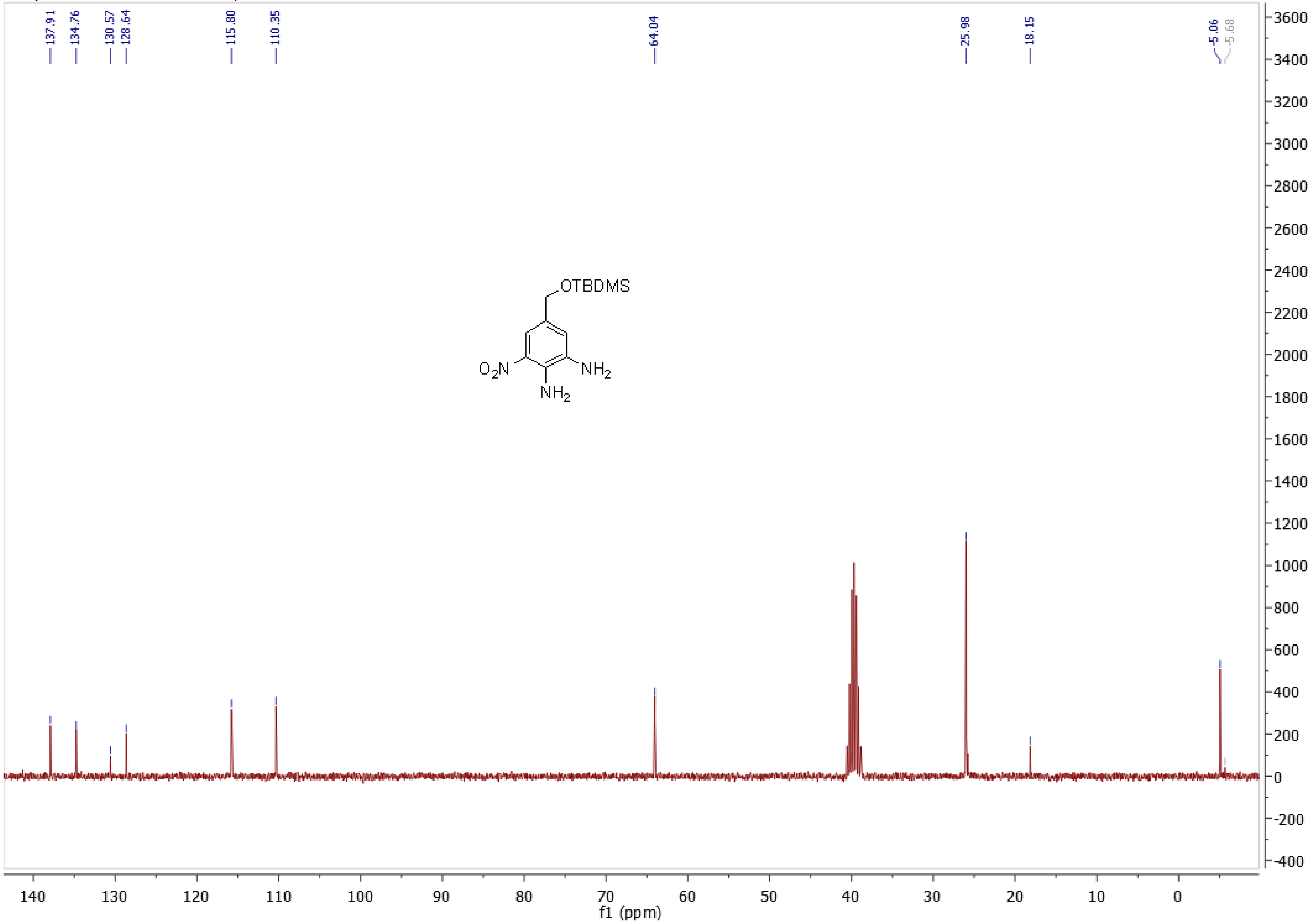

compound 18

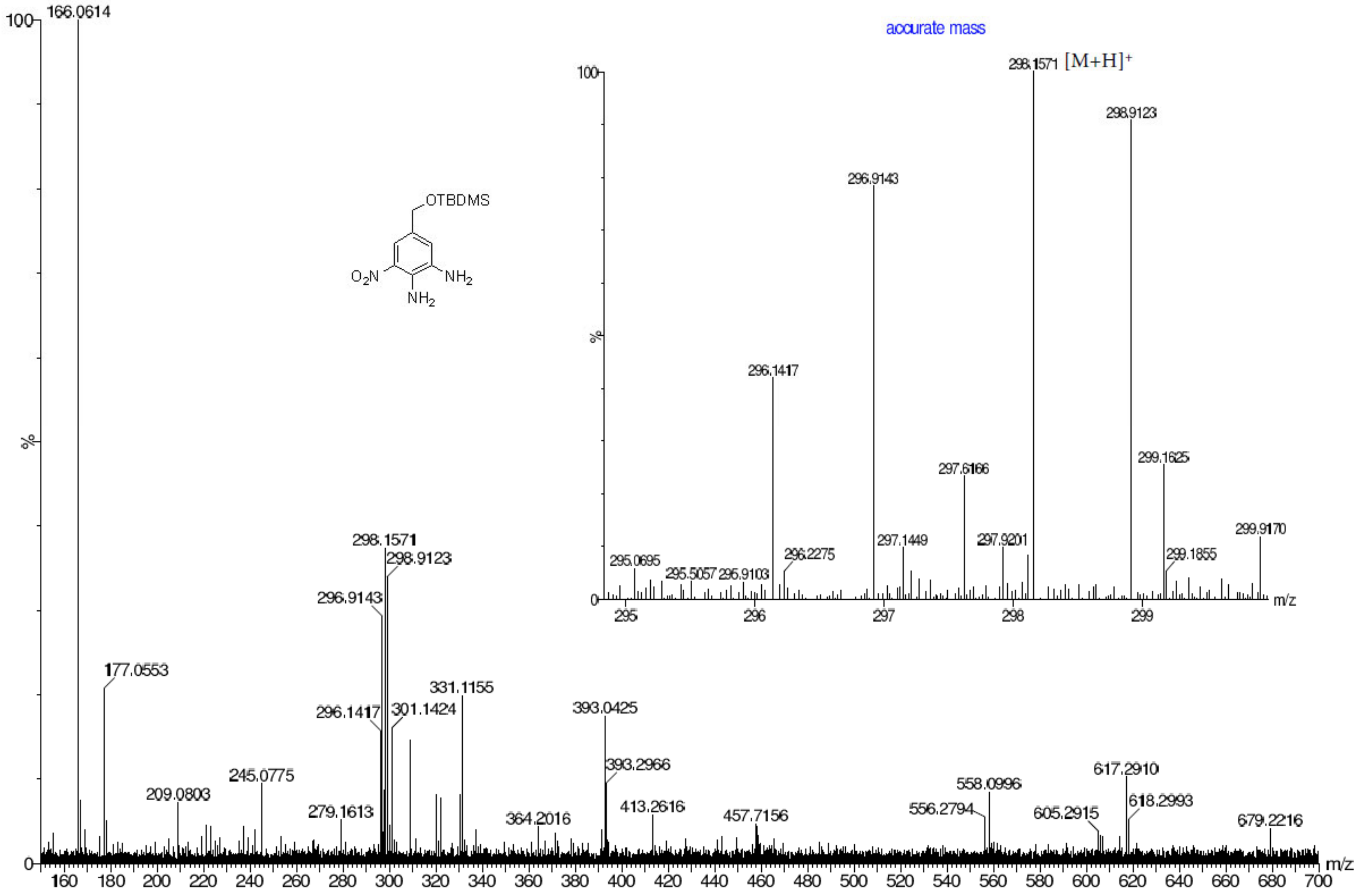
accurate mass
ES+
07-Mar-2016
100
166.0614
177.0553
209.0803
245.0775
279.1613
296.1417
296.9143
298.1571
298.9123
301.1424
331.1155
364.2016
393.0425
393.2966
413.2616
457.7156
556.2794
558.0996
605.2915
617.2910
618.2993
679.2216
%
0
m/z
OTBDMS
O2N
NH2
NH2
accurate mass
[M+H]+
295.0695
295.5057
296.9103
296.2275
297.1449
297.6166
297.9201
299.1625
299.1855
299.9170

Compound 19 in DMSO-d6 1H spectrum - 300 MHz

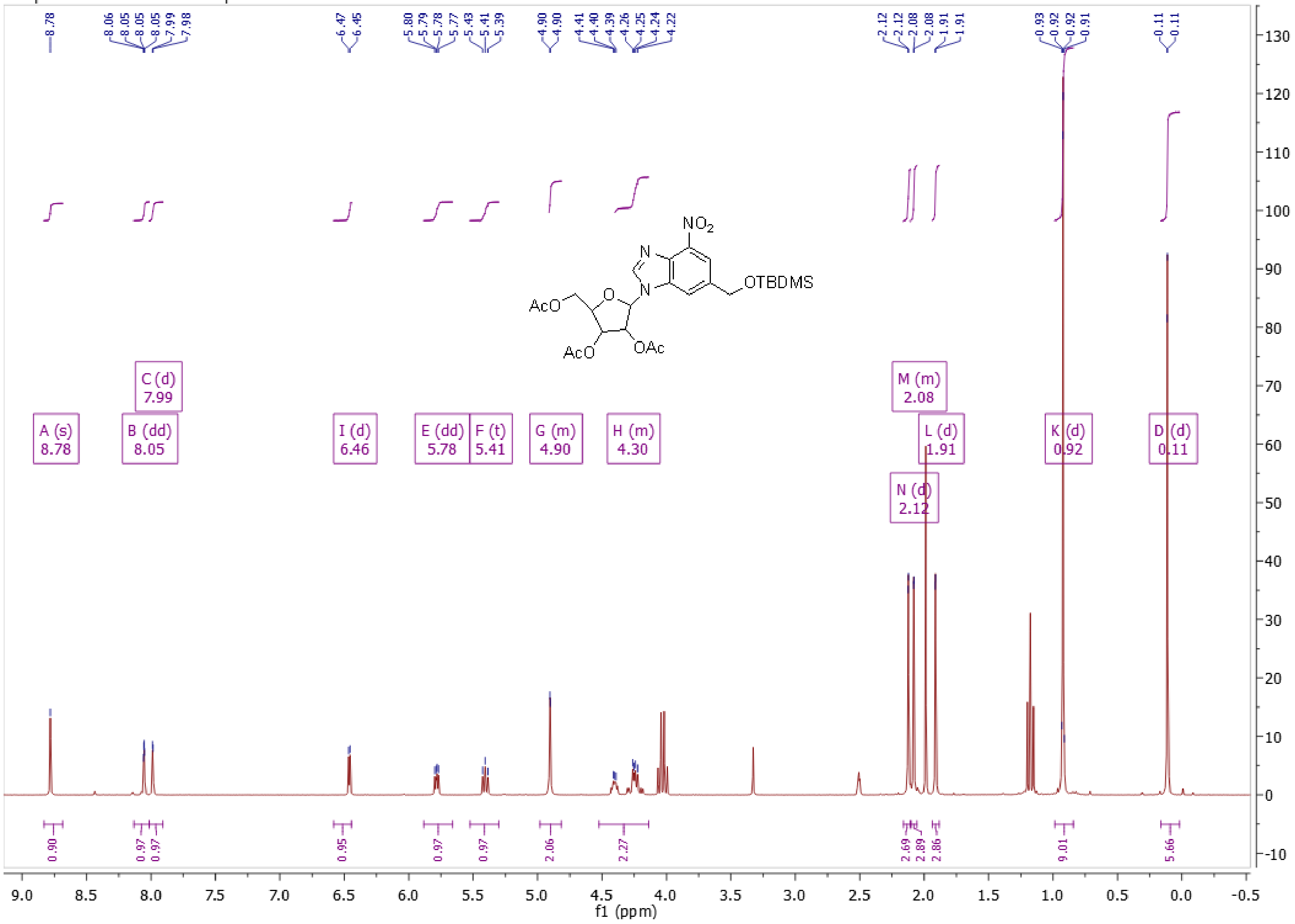

Compound 19 in DMSO-d6 13C spectrum - 75 MHz

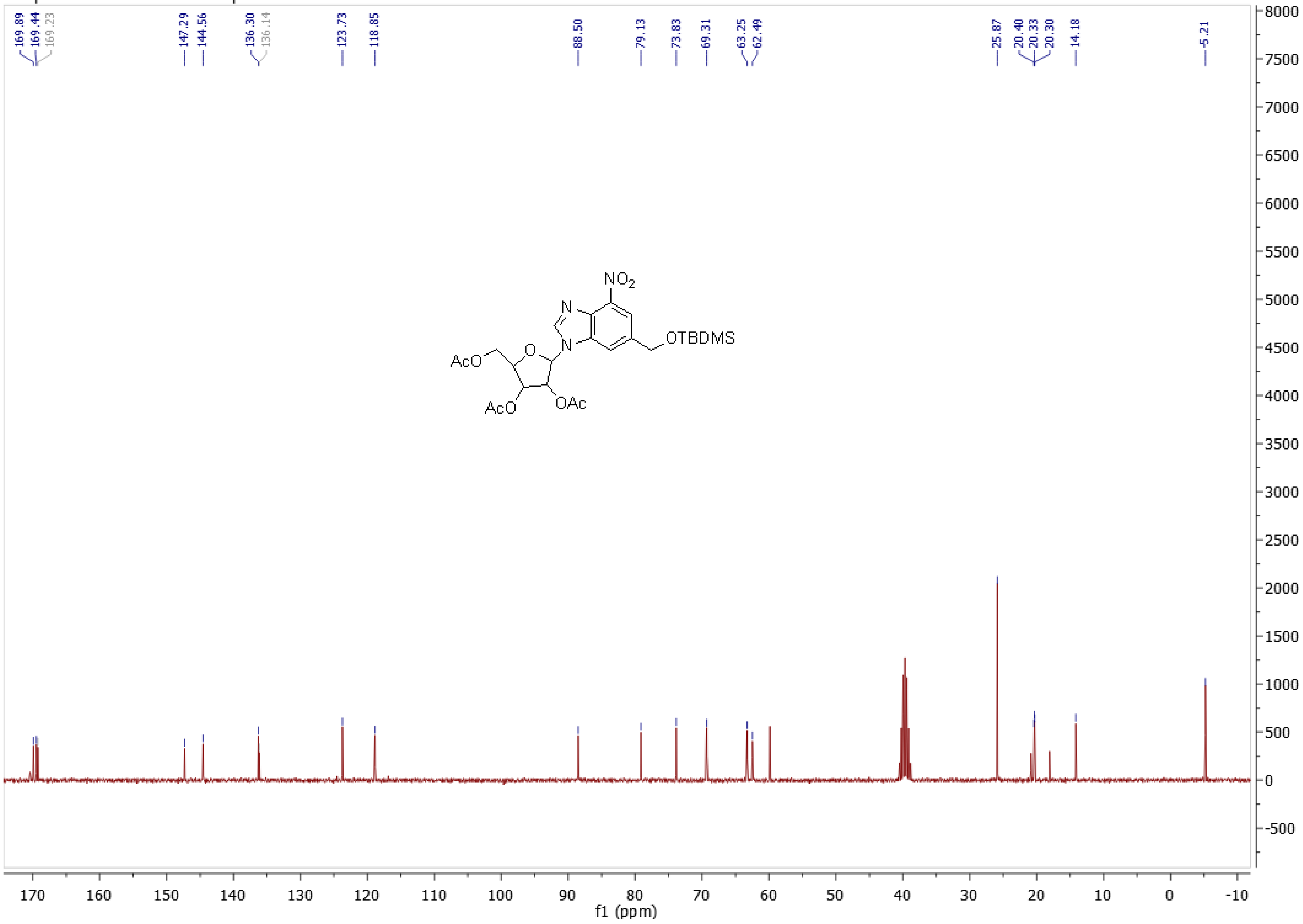

compound 19

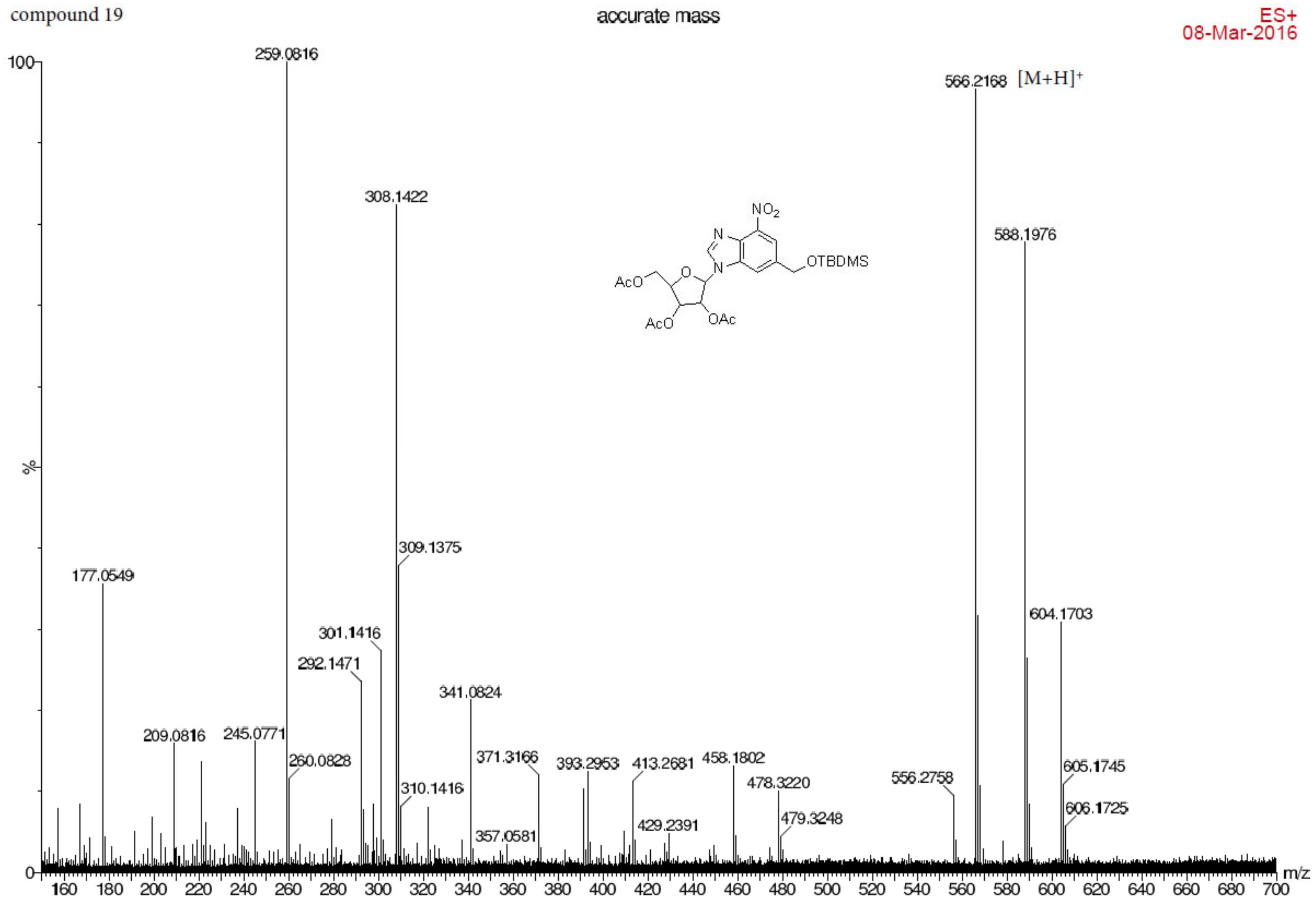
accurate mass
ES+
08-Mar-2016
100
%
0
259.0816
566.2168 [M+H]+
308.1422
588.1976
309.1375
177.0549
604.1703
301.1416
292.1471
341.0824
209.0816
245.0771
260.0828
371.3166
393.2953
413.2681
458.1802
478.3220
556.2758
605.1745
310.1416
606.1725
479.3248
429.2391
357.0581
NO2
N
N
O
OTBDMS
AcO
AcO
OAc
m/z
160
180
200
220
240
260
280
300
320
340
360
380
400
420
440
460
480
500
520
540
560
580
600
620
640
660
680
700

Compound 20 in DMSO-d6 1H spectrum - 300 MHz

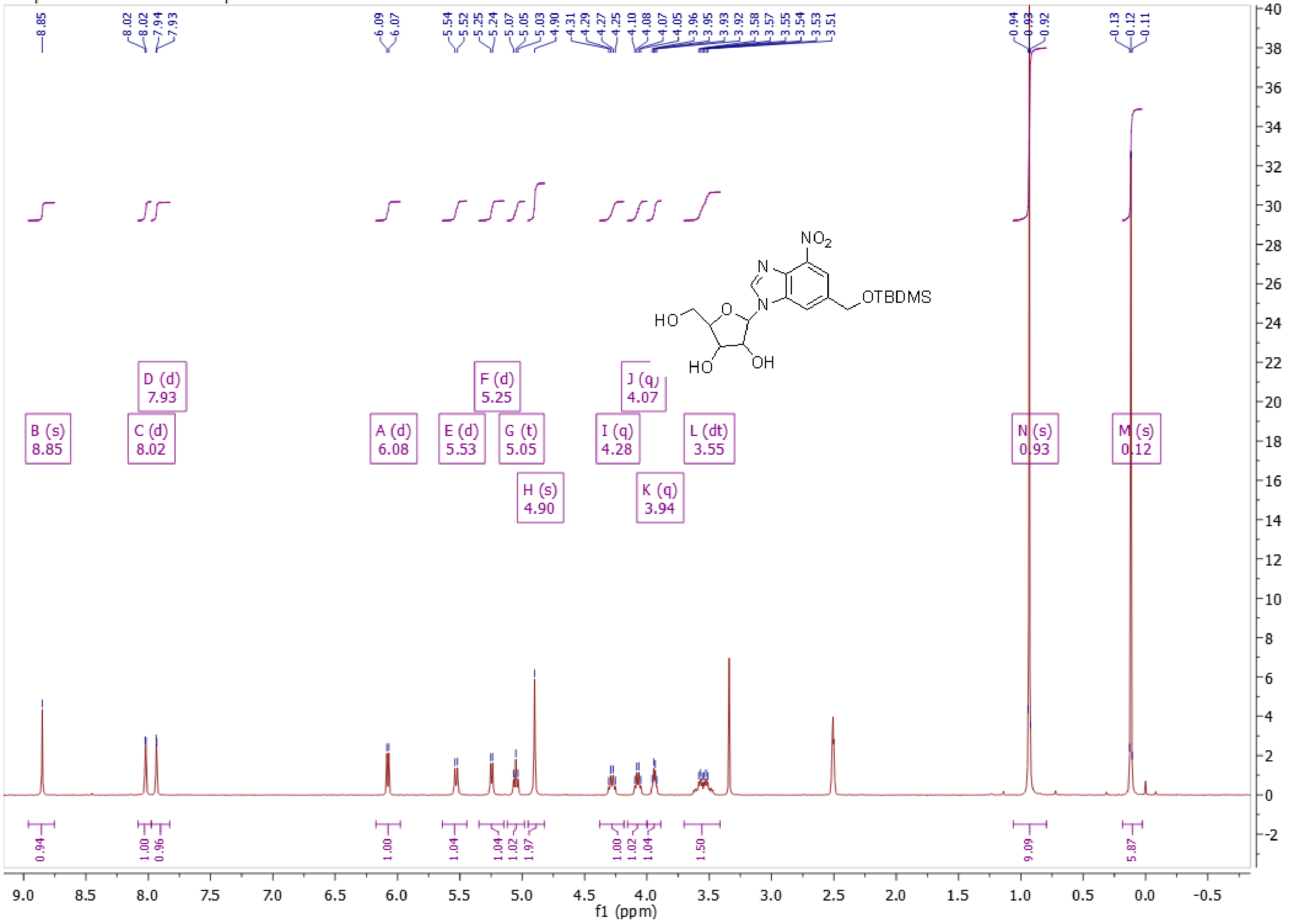

Compound 20 in DMSO-d6 13C spectrum - 75 MHz

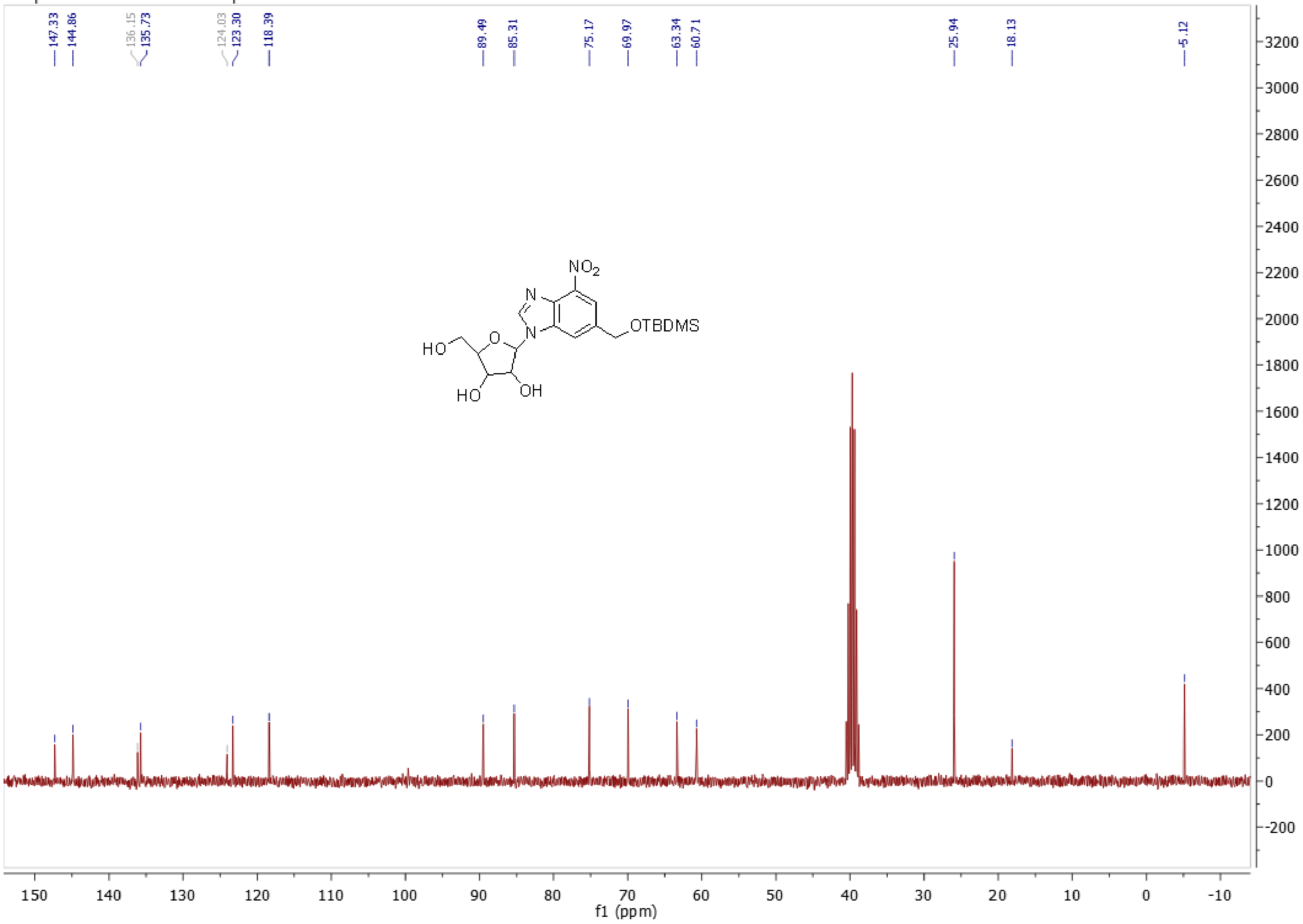

compound 20

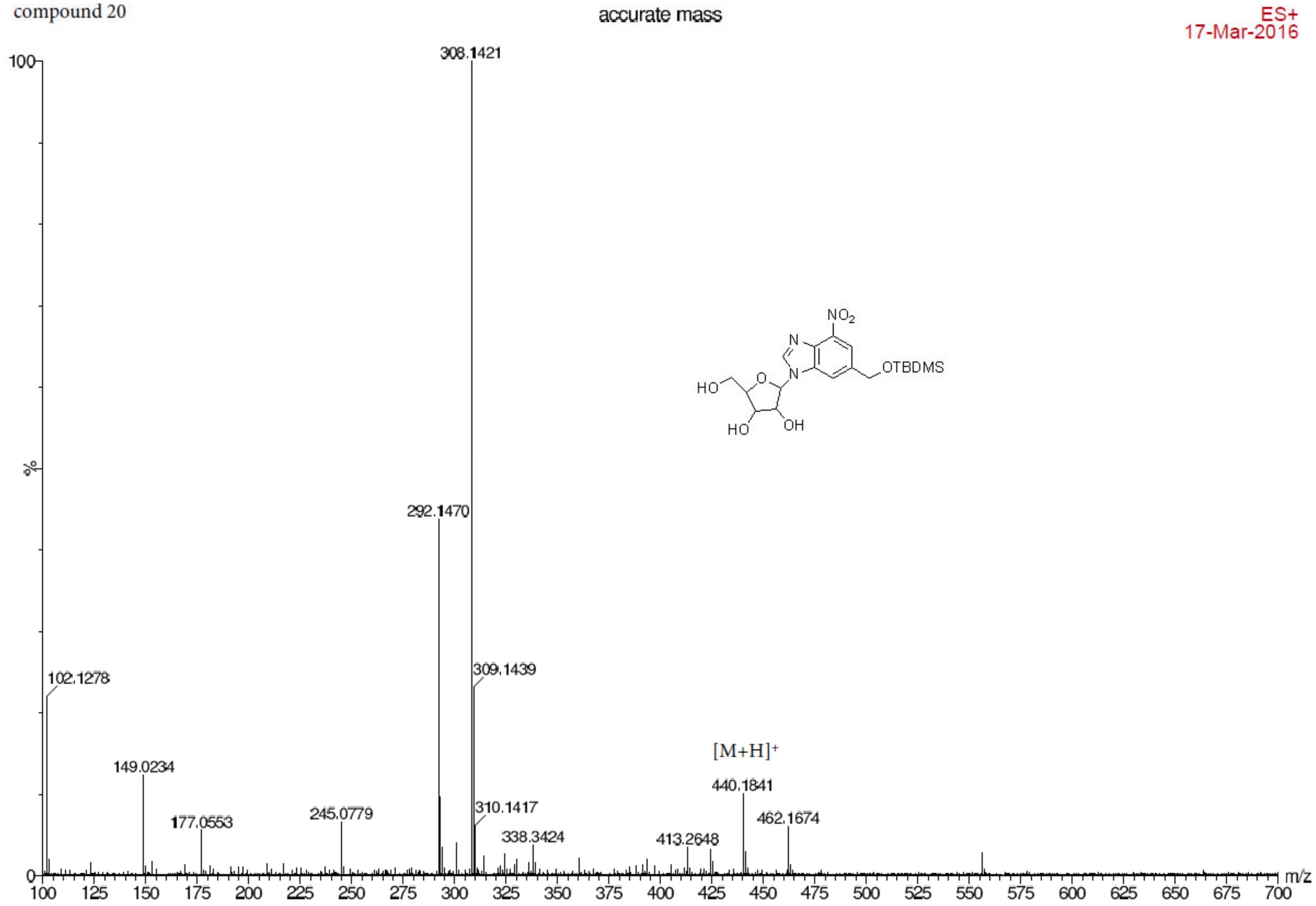

accurate mass
ES+
17-Mar-2016
308.1421
292.1470
309.1439
102.1278
149.0234
177.0553
245.0779
310.1417
338.3424
413.2648
[M+H]+
440.1841
462.1674
%
m/z
NO2
N
N
O
HO
HO
OH
OTBDMS

Compound 21 in DMSO-d6 1H spectrum - 300 MHz

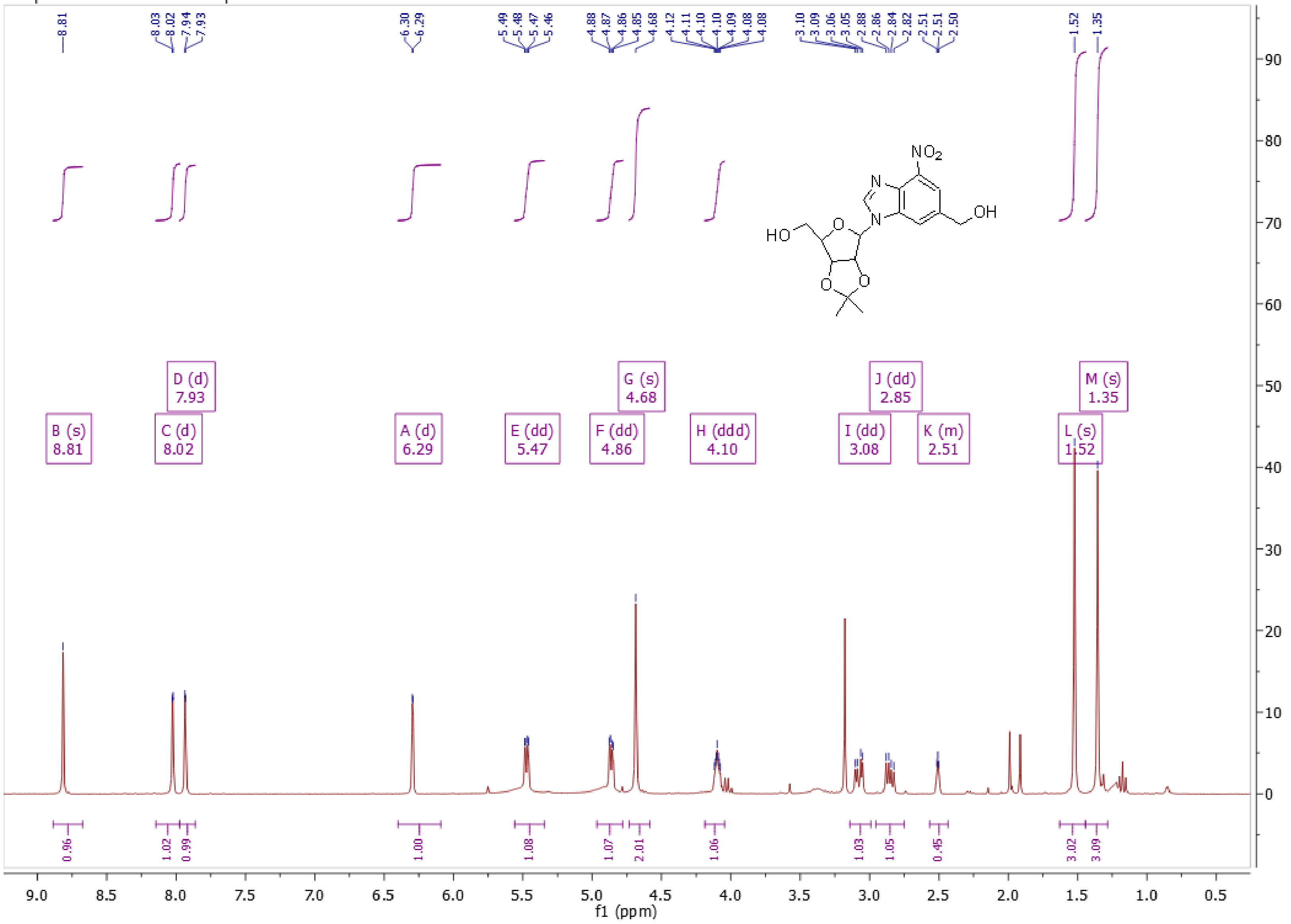

Compound 21 in DMSO-d6 13C spectrum - 75 MHz

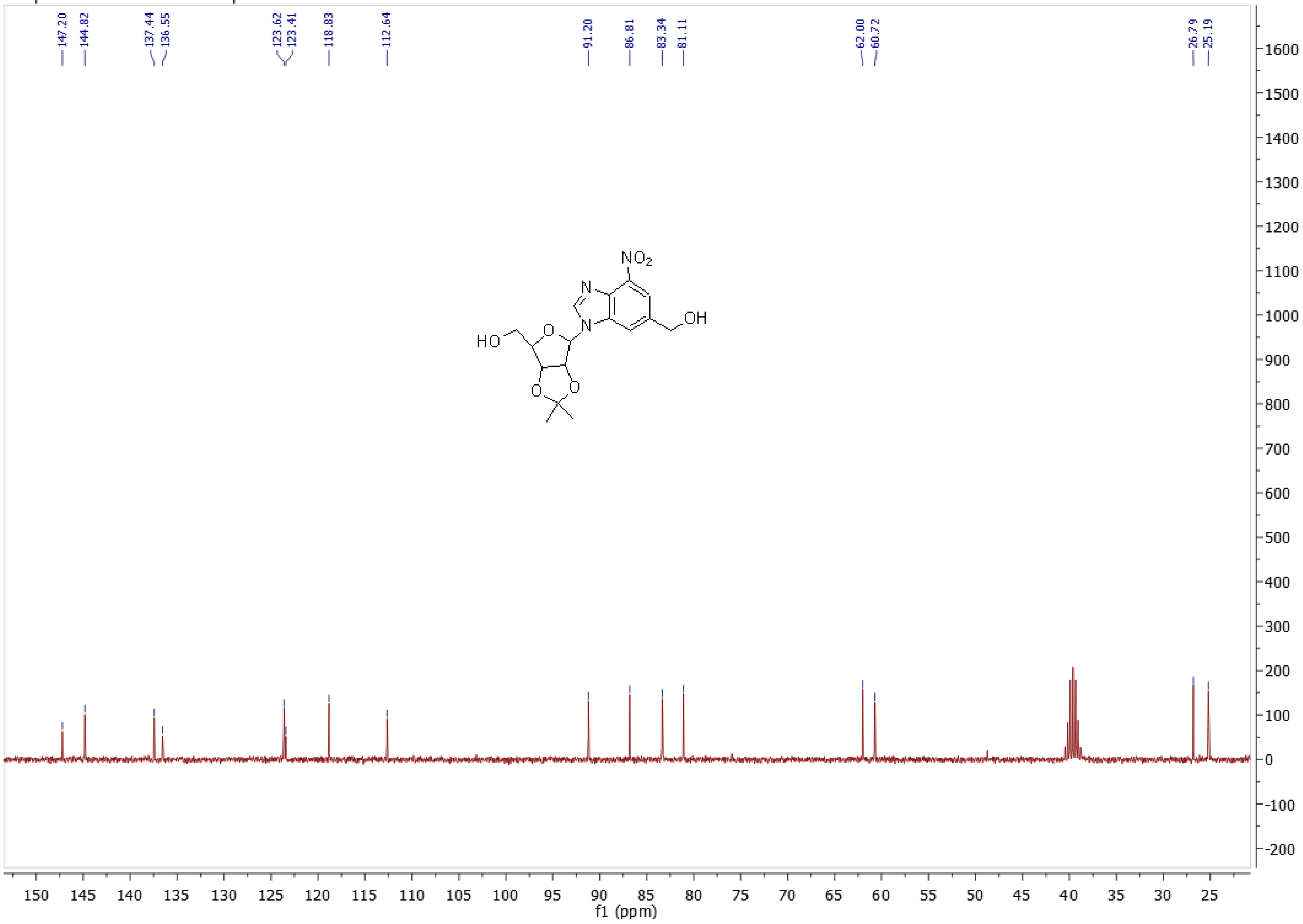

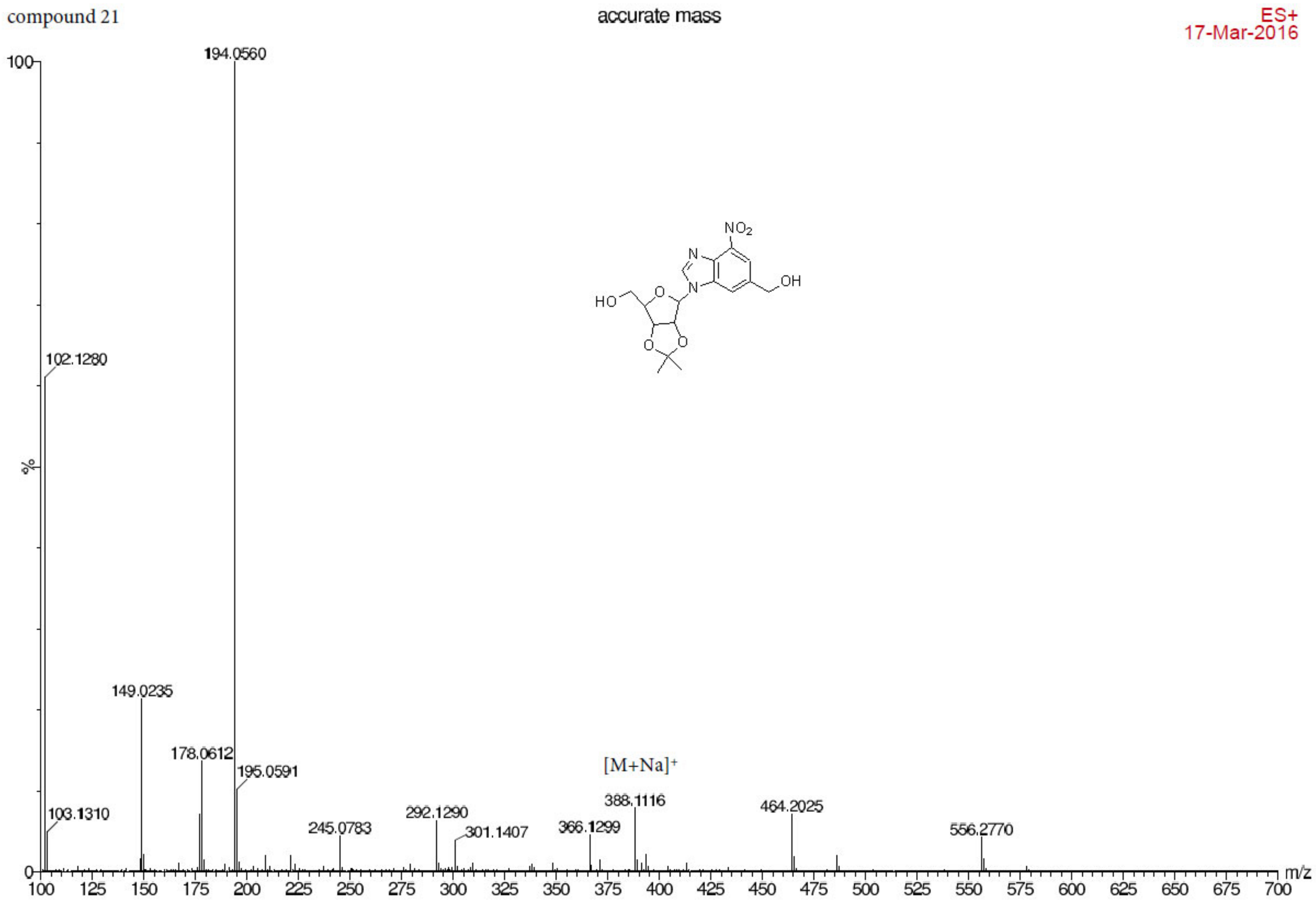

compound 21
accurate mass
ES+
17-Mar-2016
194.0560
102.1280
149.0235
178.0612
195.0591
103.1310
245.0783
292.1290
301.1407
366.1299
[M+Na]+
388.1116
464.2025
556.2770
%
m/z
NO2
N
N
O
HO
OH
O
O

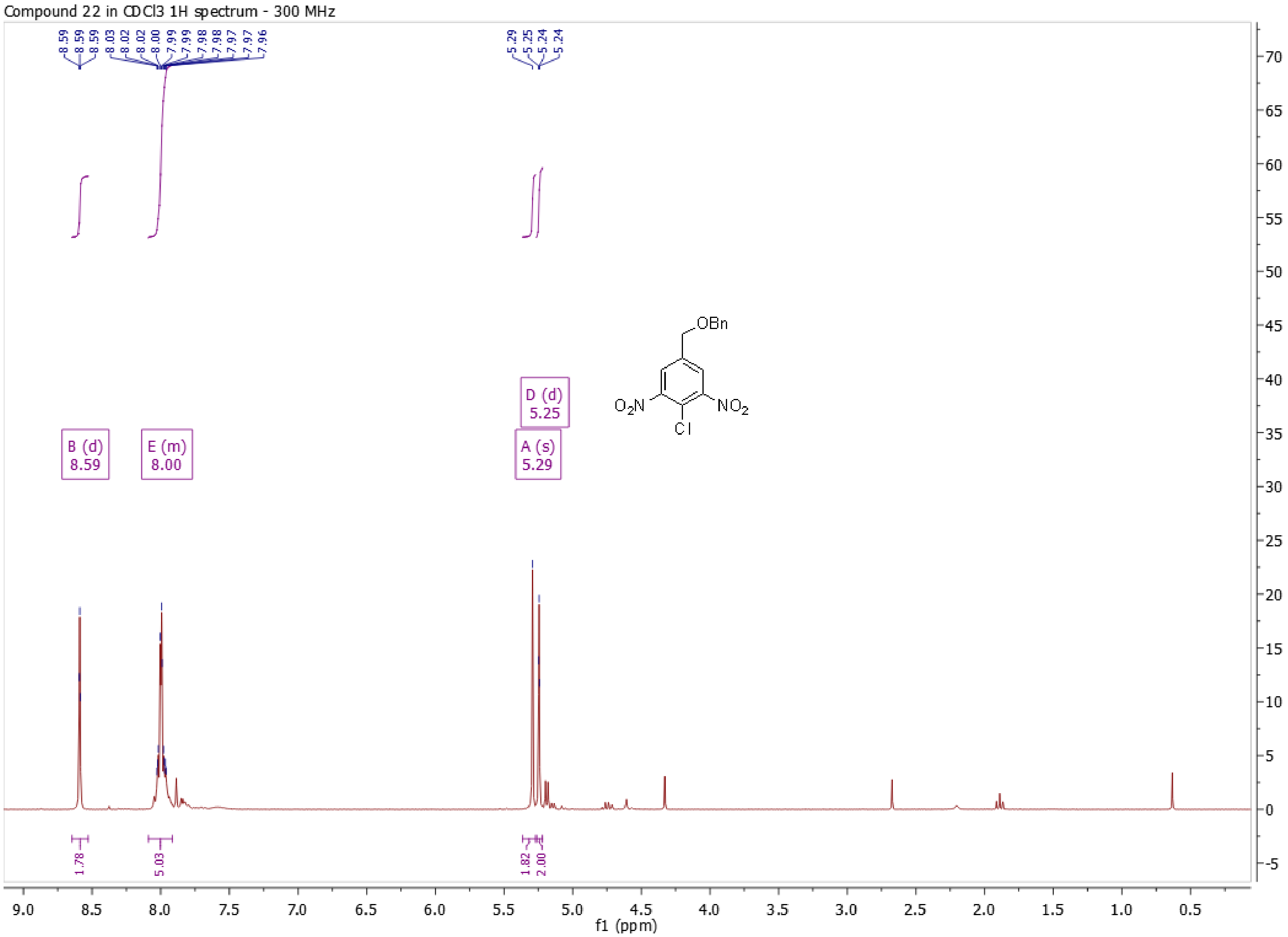

Compound 22 in CDCl3 1H spectrum - 300 MHz
8.59
8.59
8.59
8.03
8.02
8.02
8.00
7.99
7.99
7.98
7.98
7.97
7.97
7.96
5.29
5.25
5.24
5.24
OBn
O_2N
NO_2
Cl
D (d)
5.25
B (d)
8.59
E (m)
8.00
A (s)
5.29
1.78
5.03
1.82
2.00
f1 (ppm)

Compound 22 in CDCl3 13C spectrum - 75 MHz

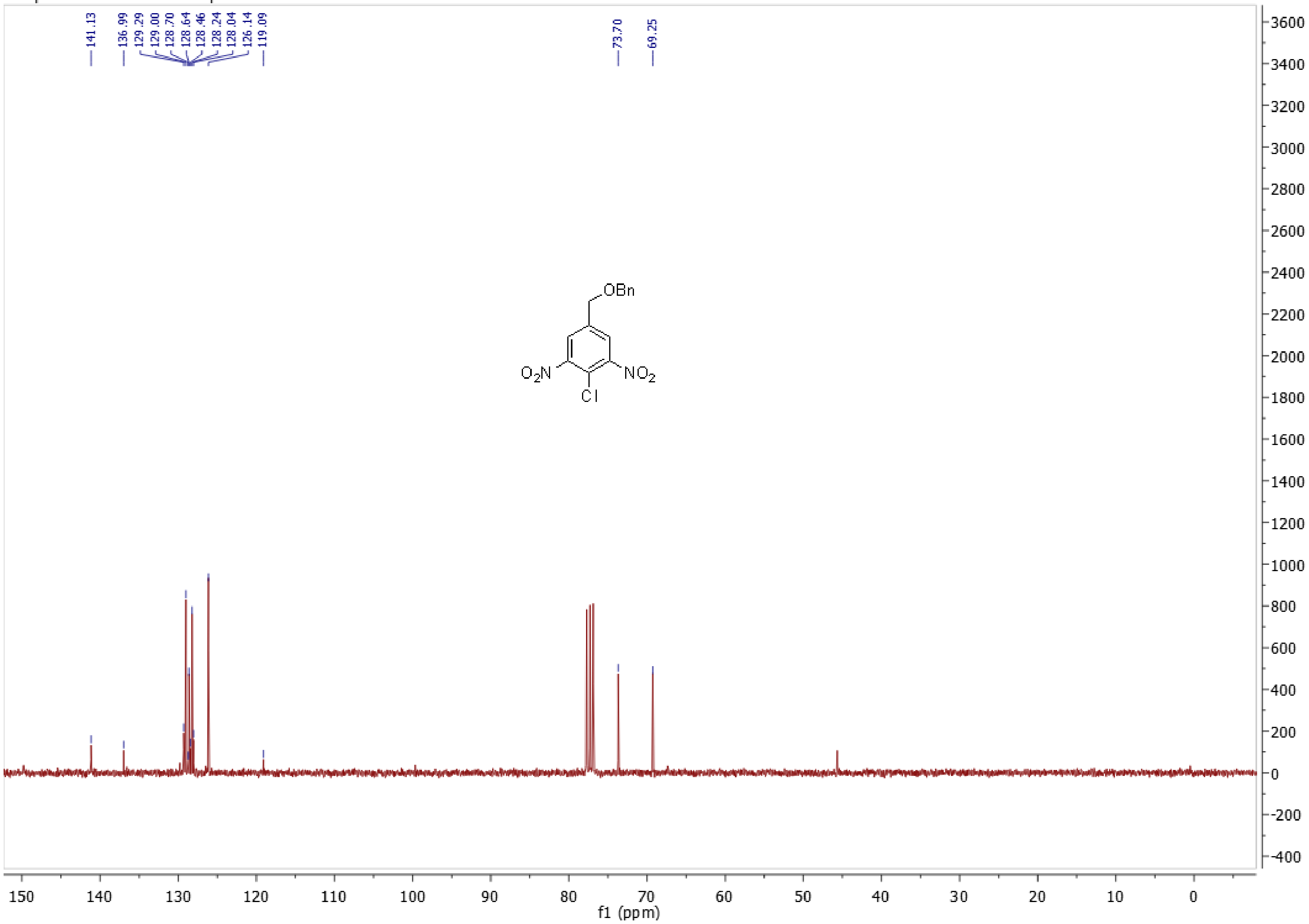

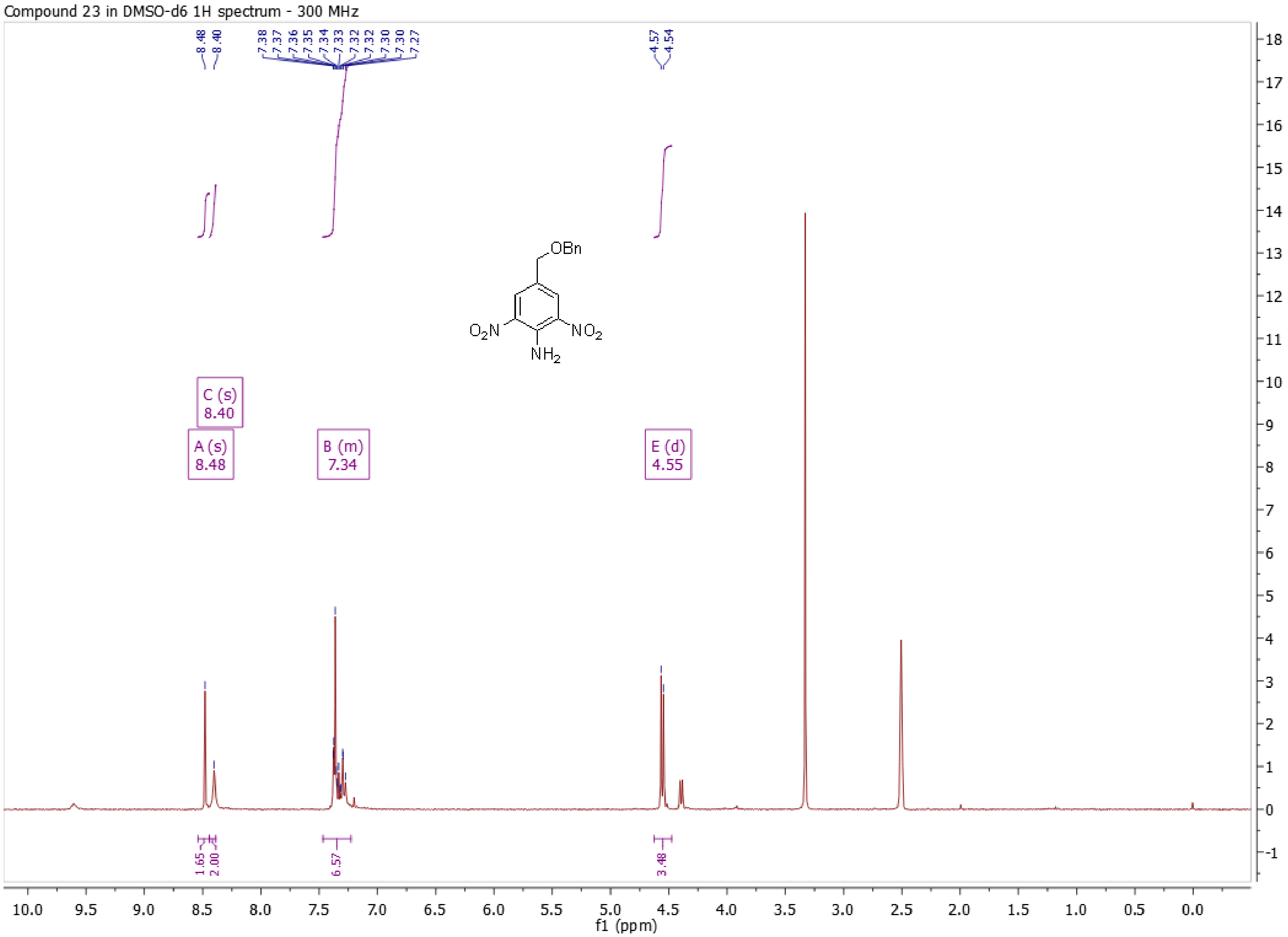

Compound 23 in DMSO-d6 1H spectrum - 300 MHz
8.48
8.40
7.38
7.37
7.36
7.35
7.34
7.33
7.32
7.32
7.30
7.30
7.27
4.57
4.54
OBn
O_2N
NO_2
NH_2
C (s)
8.40
A (s)
8.48
B (m)
7.34
E (d)
4.55
1.65
2.00
6.57
3.48
f1 (ppm)

Compound 23 in DMSO-d6 13C spectrum - 75 MHz

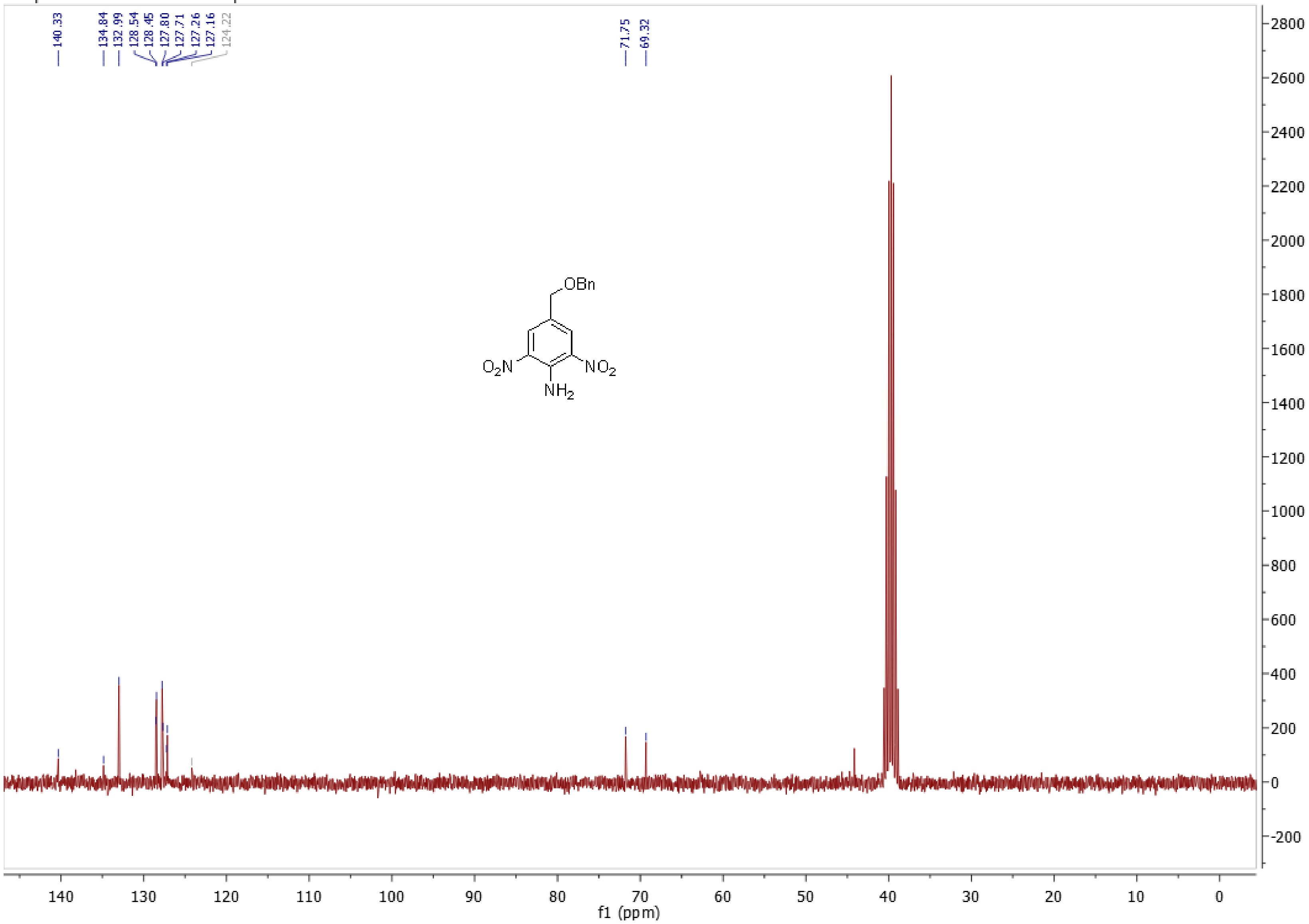

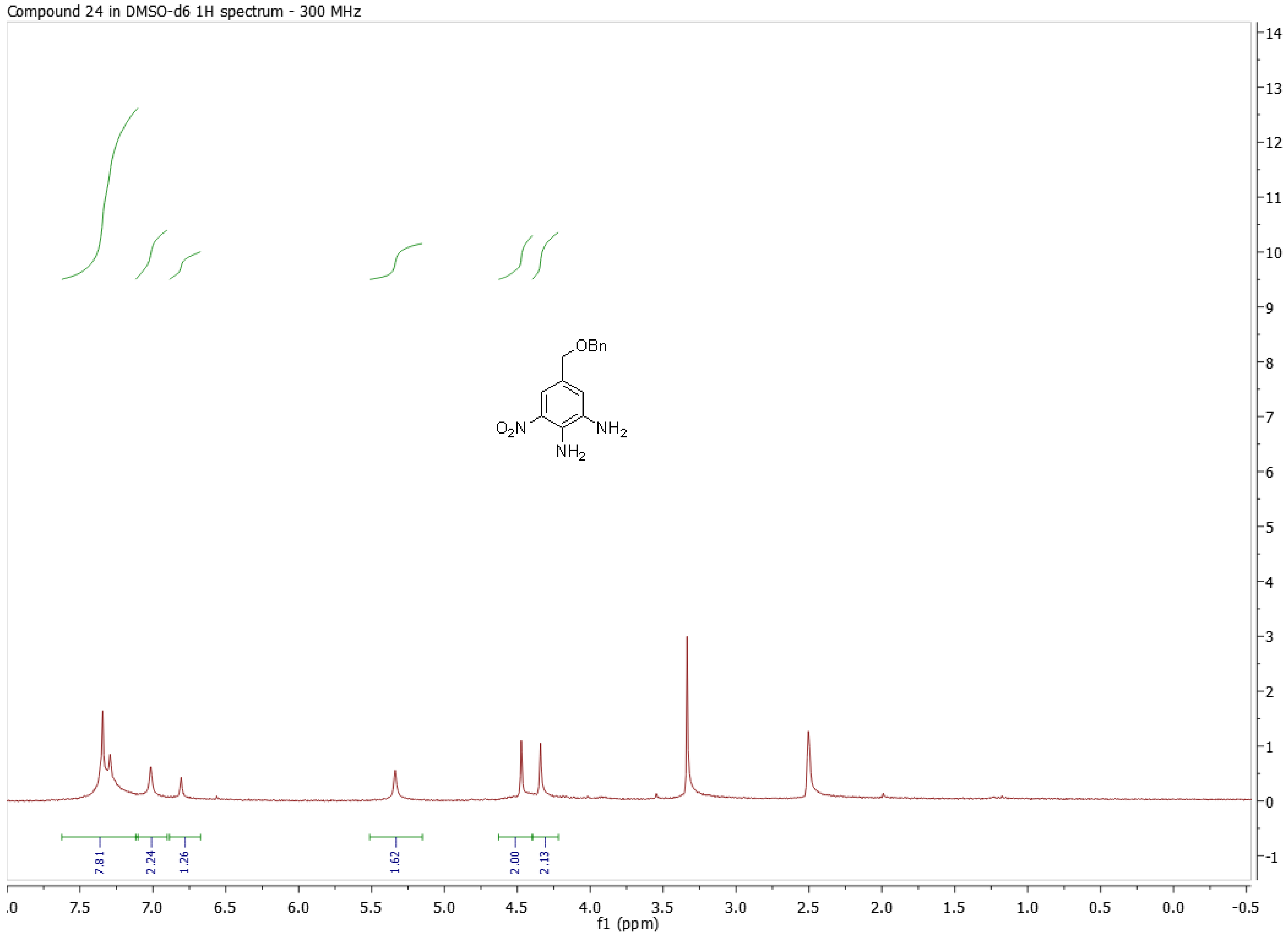
Compound 24 in DMSO-d6 1H spectrum - 300 MHz
OBn
O2N
NH2
NH2
7.81
2.24
1.26
1.62
2.00
2.13
f1 (ppm)

compound 24

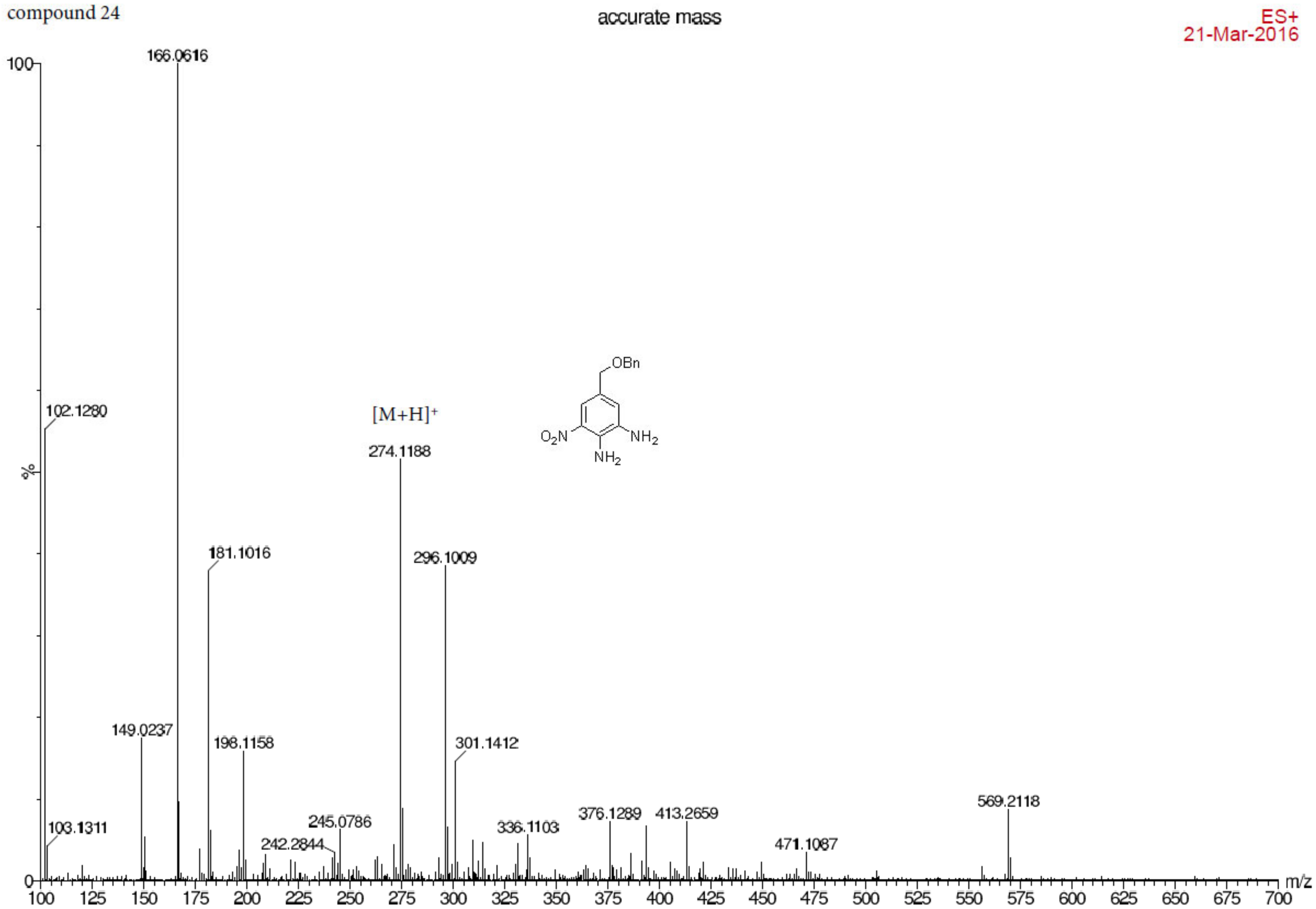
accurate mass
ES+
21-Mar-2016
100
%
0
166.0616
102.1280
103.1311
149.0237
181.1016
198.1158
242.2844
245.0786
[M+H]+
274.1188
296.1009
301.1412
336.1103
376.1289
413.2659
471.1087
569.2118
OBn
O2N
NH2
NH2
m/z
100 125 150 175 200 225 250 275 300 325 350 375 400 425 450 475 500 525 550 575 600 625 650 675 700

Compound 25 in DMSO-d6 1H spectrum - 300 MHz

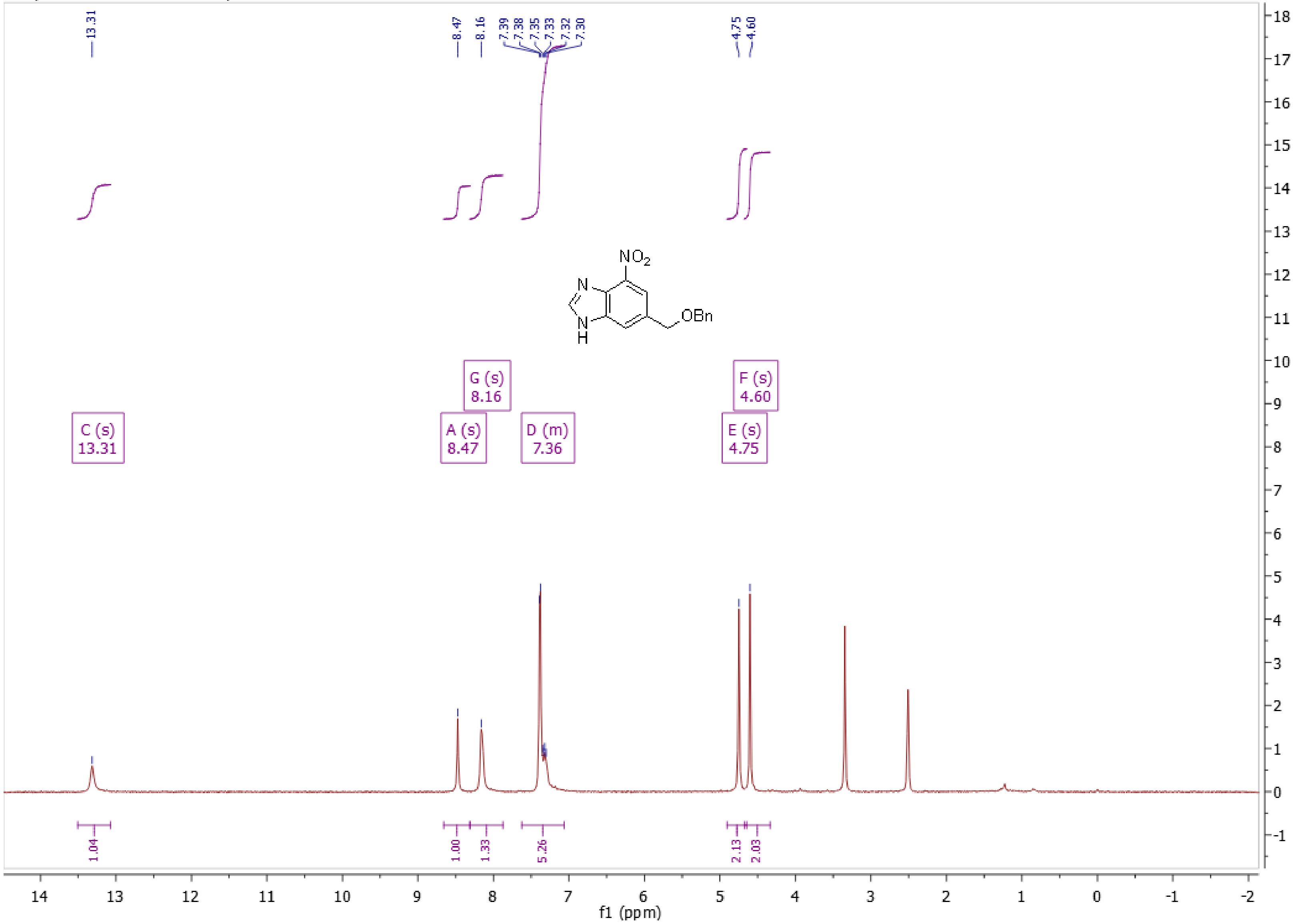

Compound 25 in DMSO-d6 13C spectrum - 75 MHz

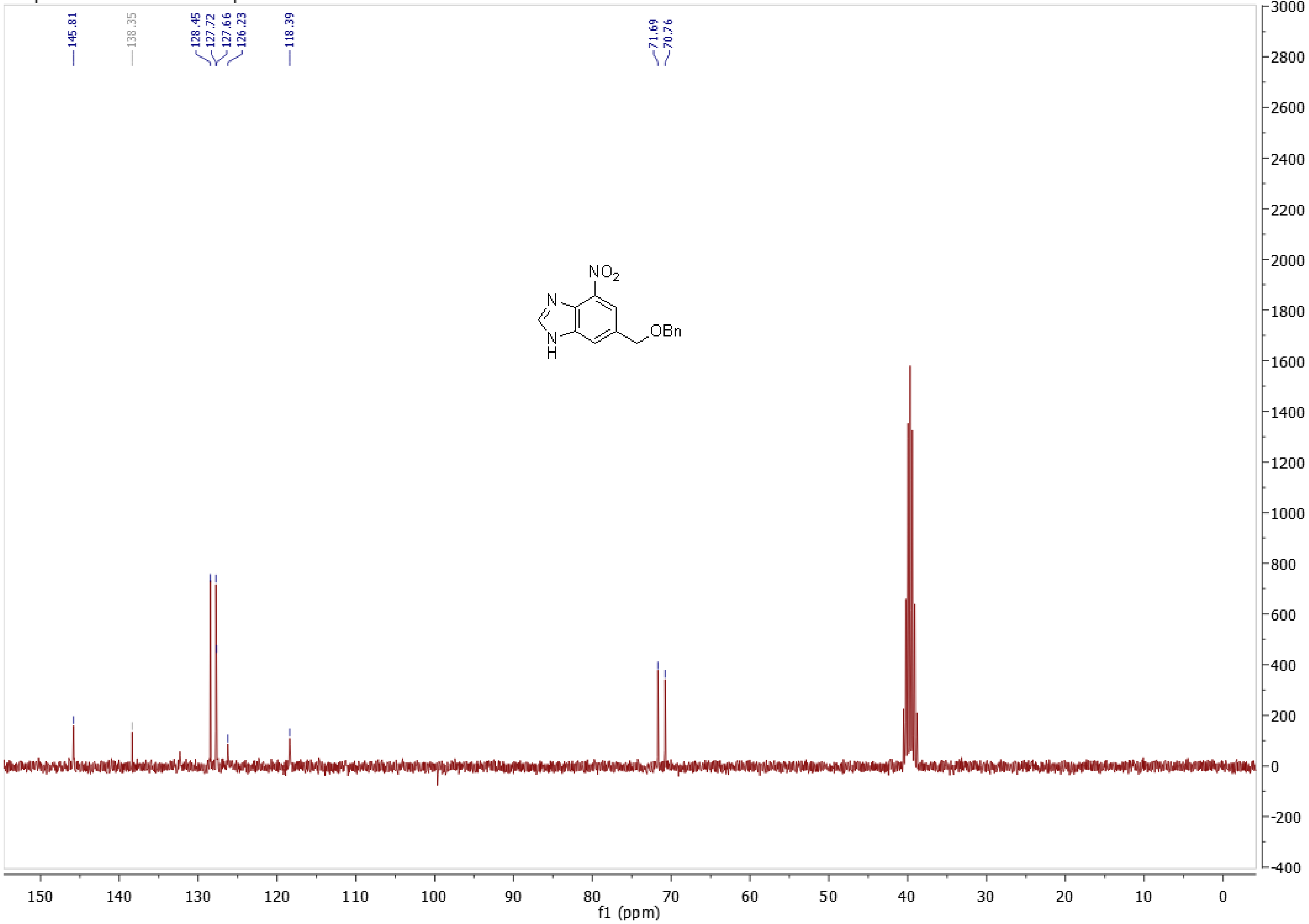

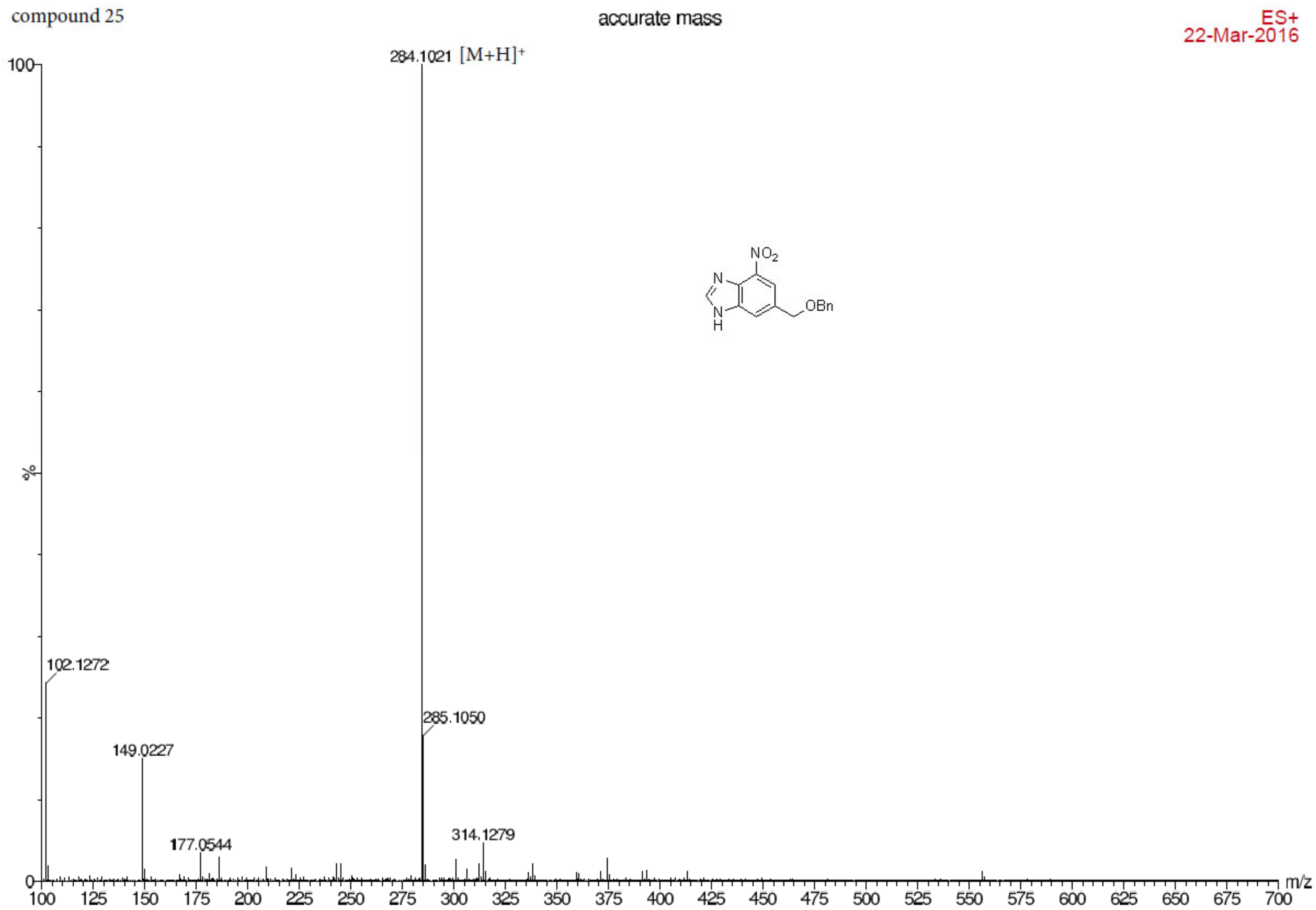
compound 25
accurate mass
ES+
22-Mar-2016
284.1021 [M+H]+
102.1272
149.0227
177.0544
285.1050
314.1279
NO2
N
N
H
OBn
%
m/z

Compound 26 in DMSO-d6 1H spectrum - 300 MHz

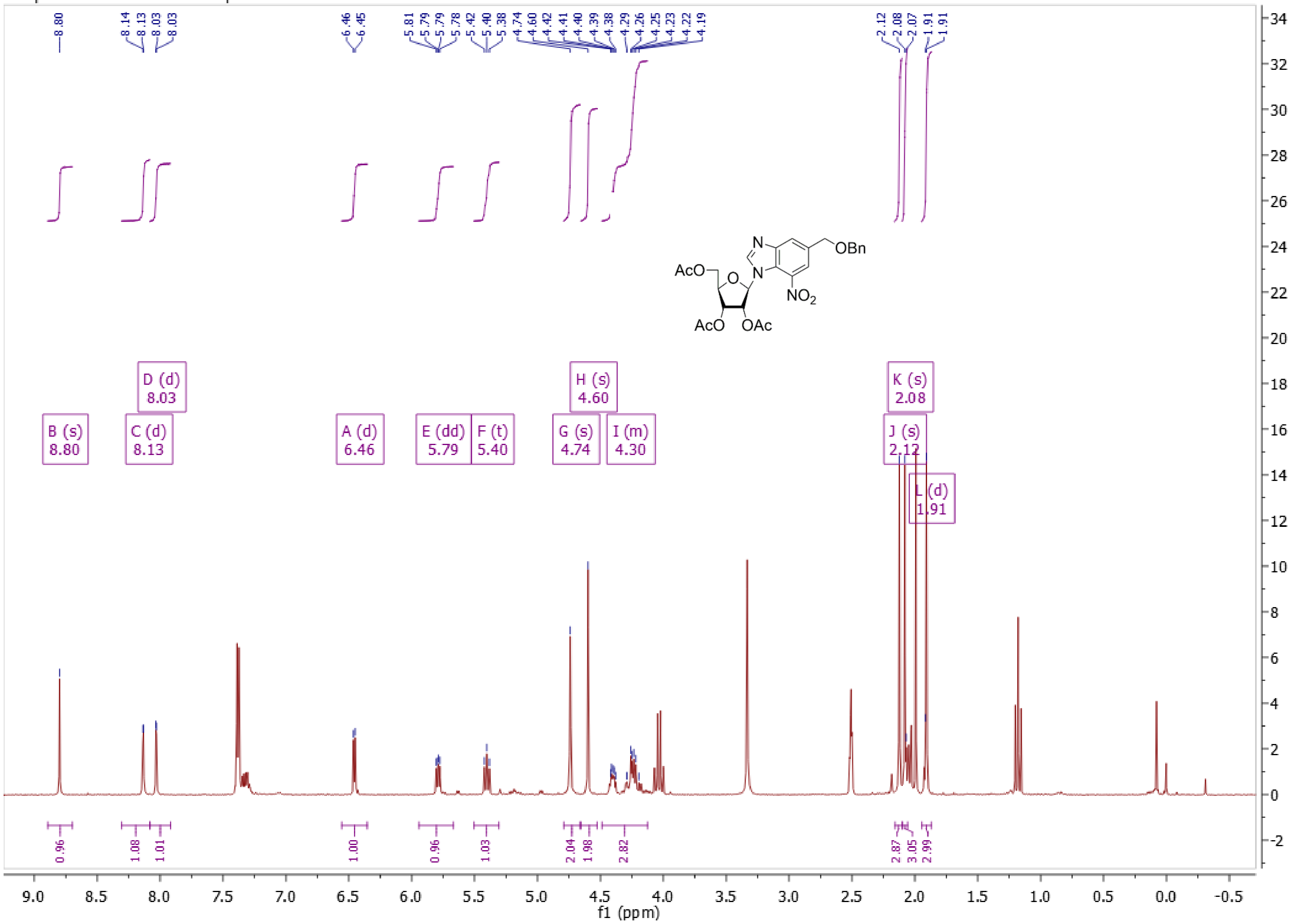

Compound 26 in DMSO-d6 13C spectrum - 75 MHz

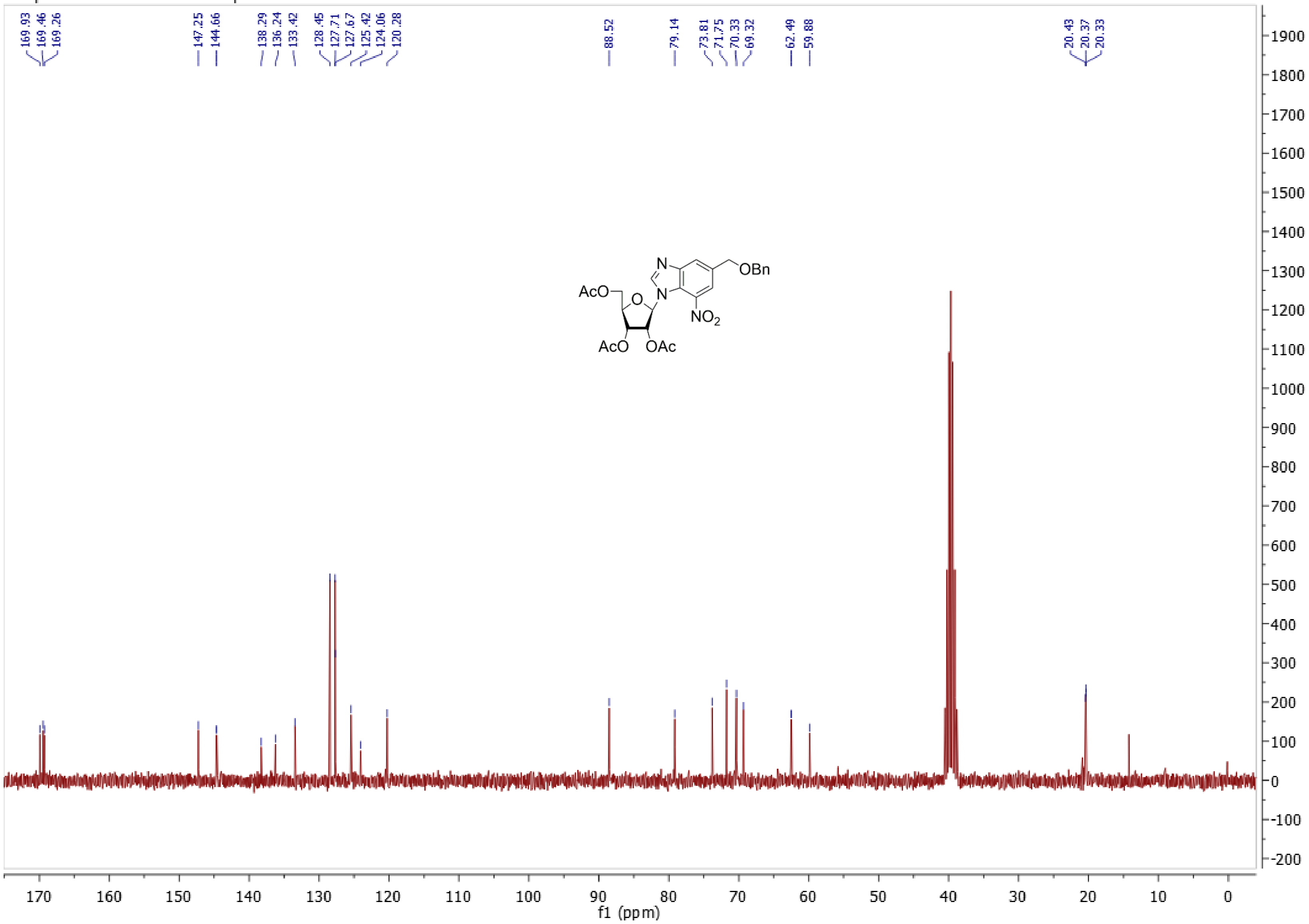

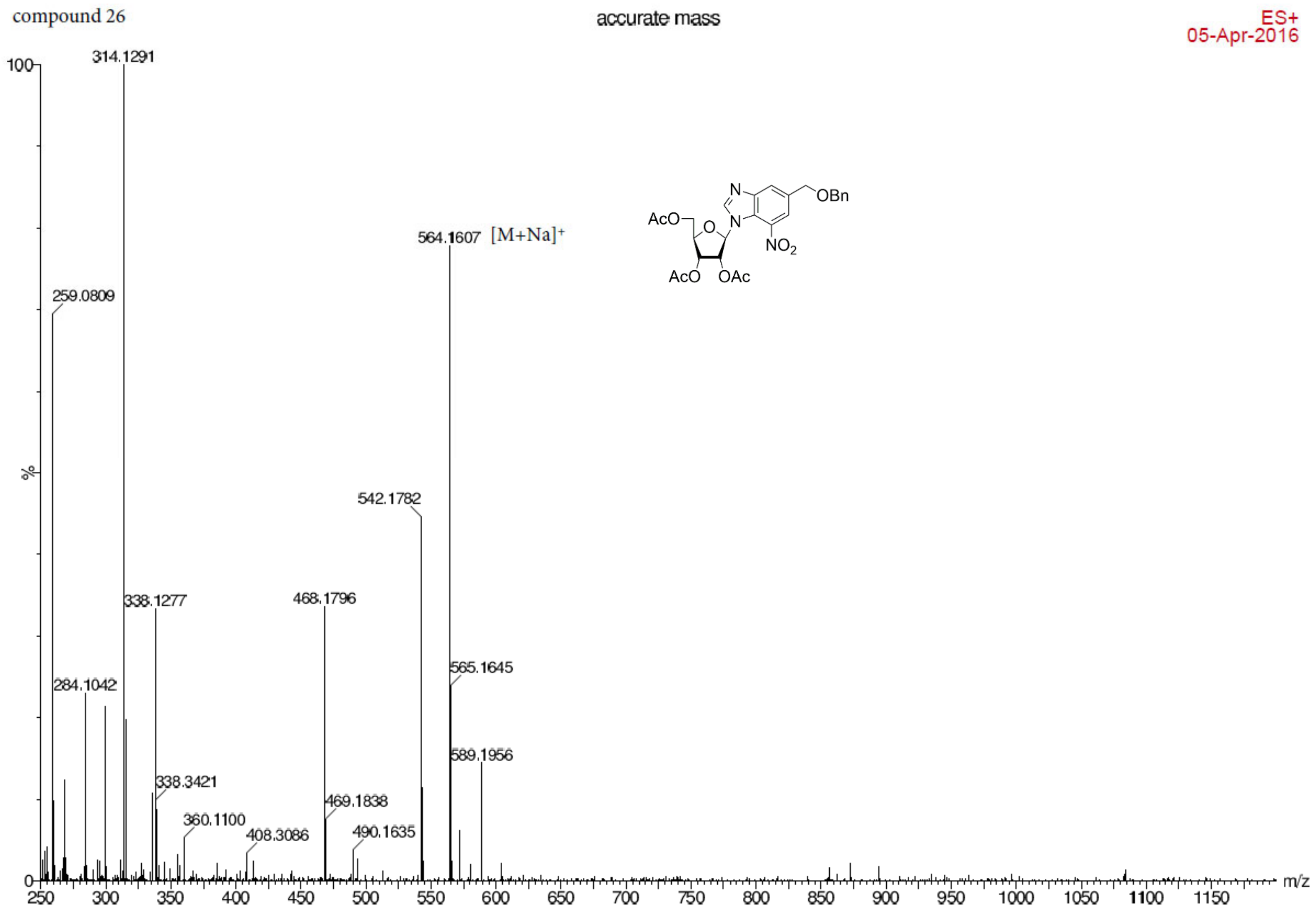
compound 26
accurate mass
ES+
05-Apr-2016
100
%
0
314.1291
564.1607 [M+Na]+
259.0809
542.1782
338.1277
468.1796
565.1645
284.1042
589.1956
338.3421
469.1838
360.1100
490.1635
408.3086
250
300
350
400
450
500
550
600
650
700
750
800
850
900
950
1000
1050
1100
1150
m/z
AcO
OBn
NO2
AcO
OAc
N
O

Compound 27 in DMSO-d6 1H spectrum - 300 MHz

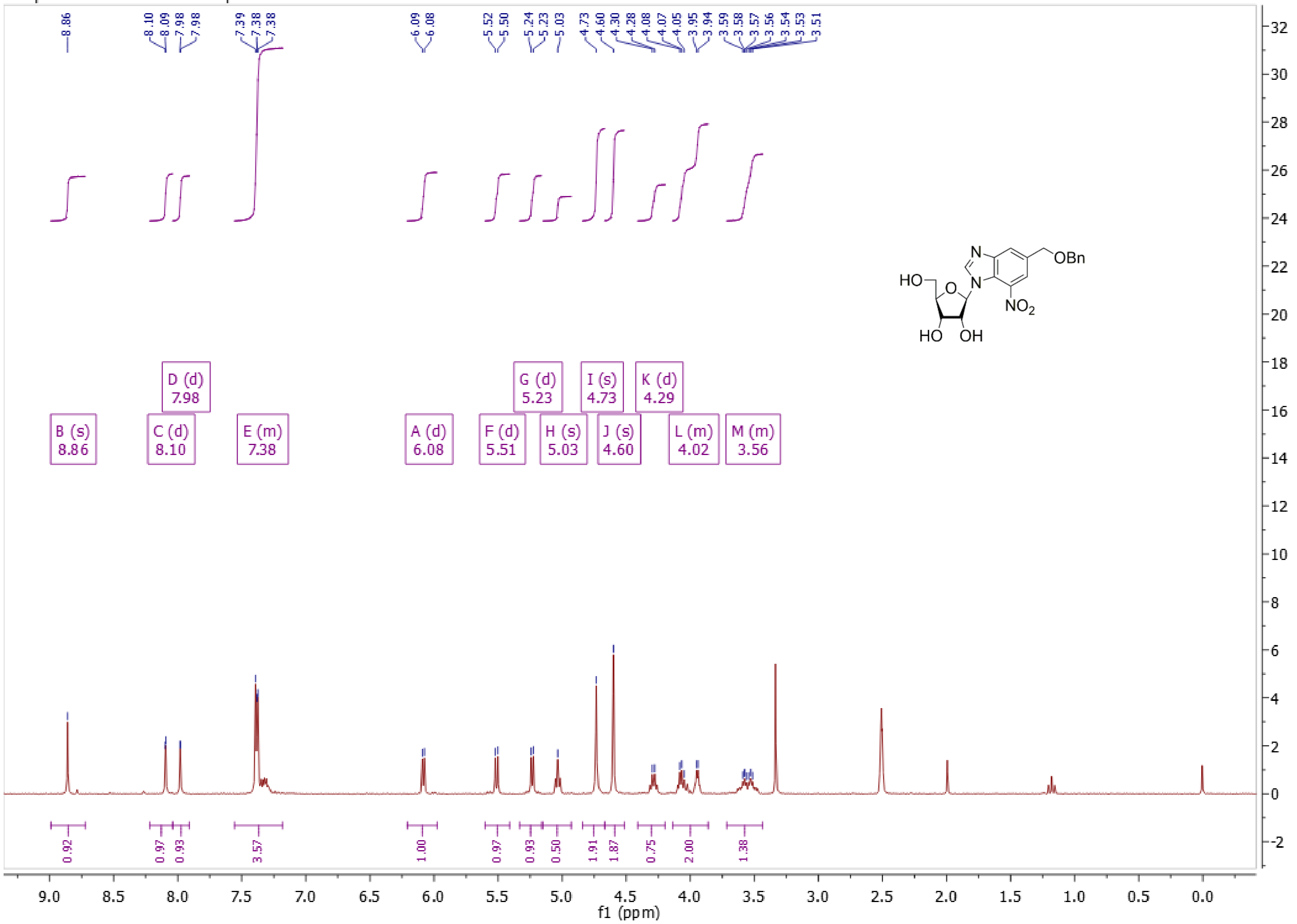

Compound 27 in DMSO-d6 13C spectrum - 75 MHz

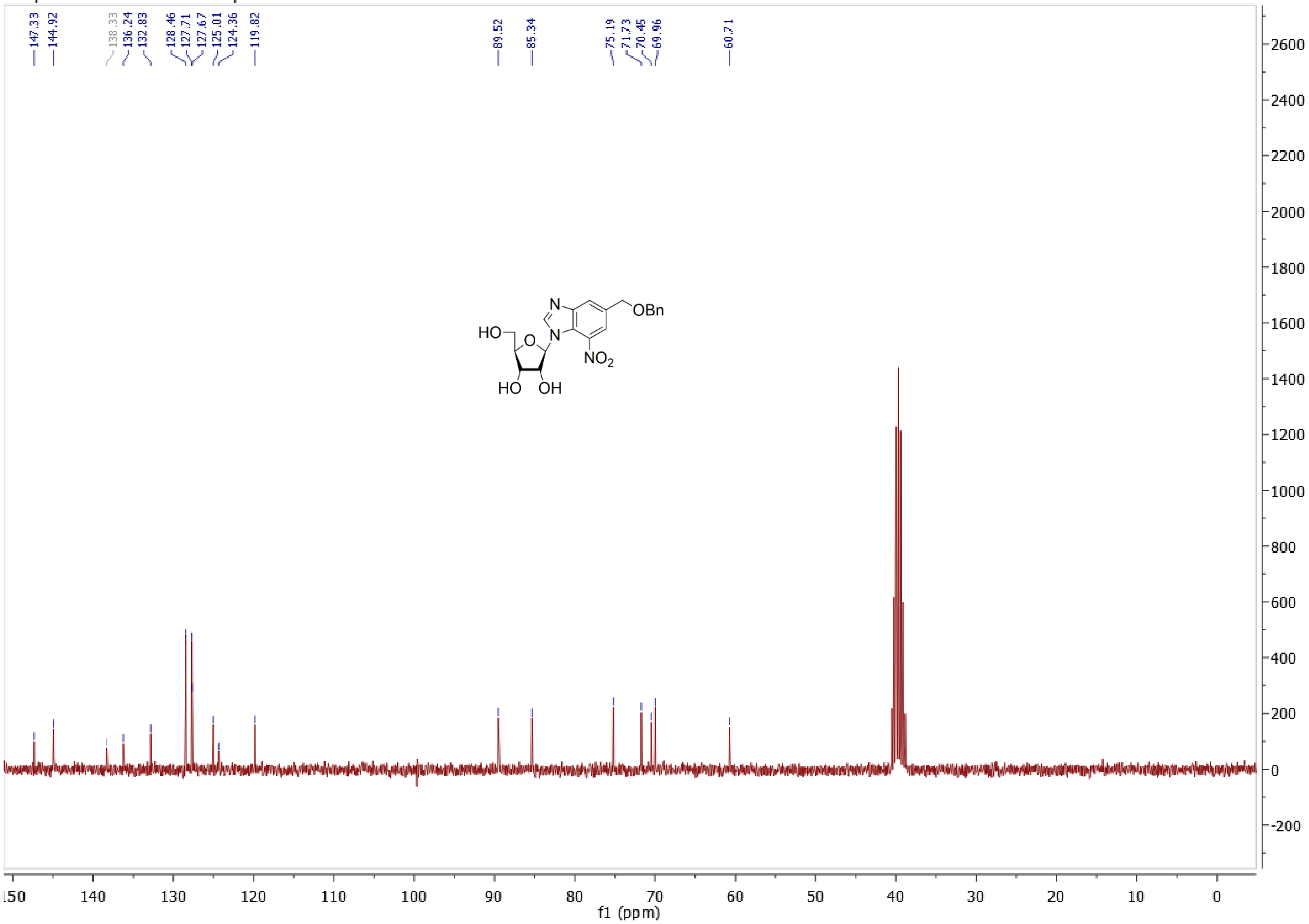

compound 27

accurate mass

ES+
05-Apr-2016

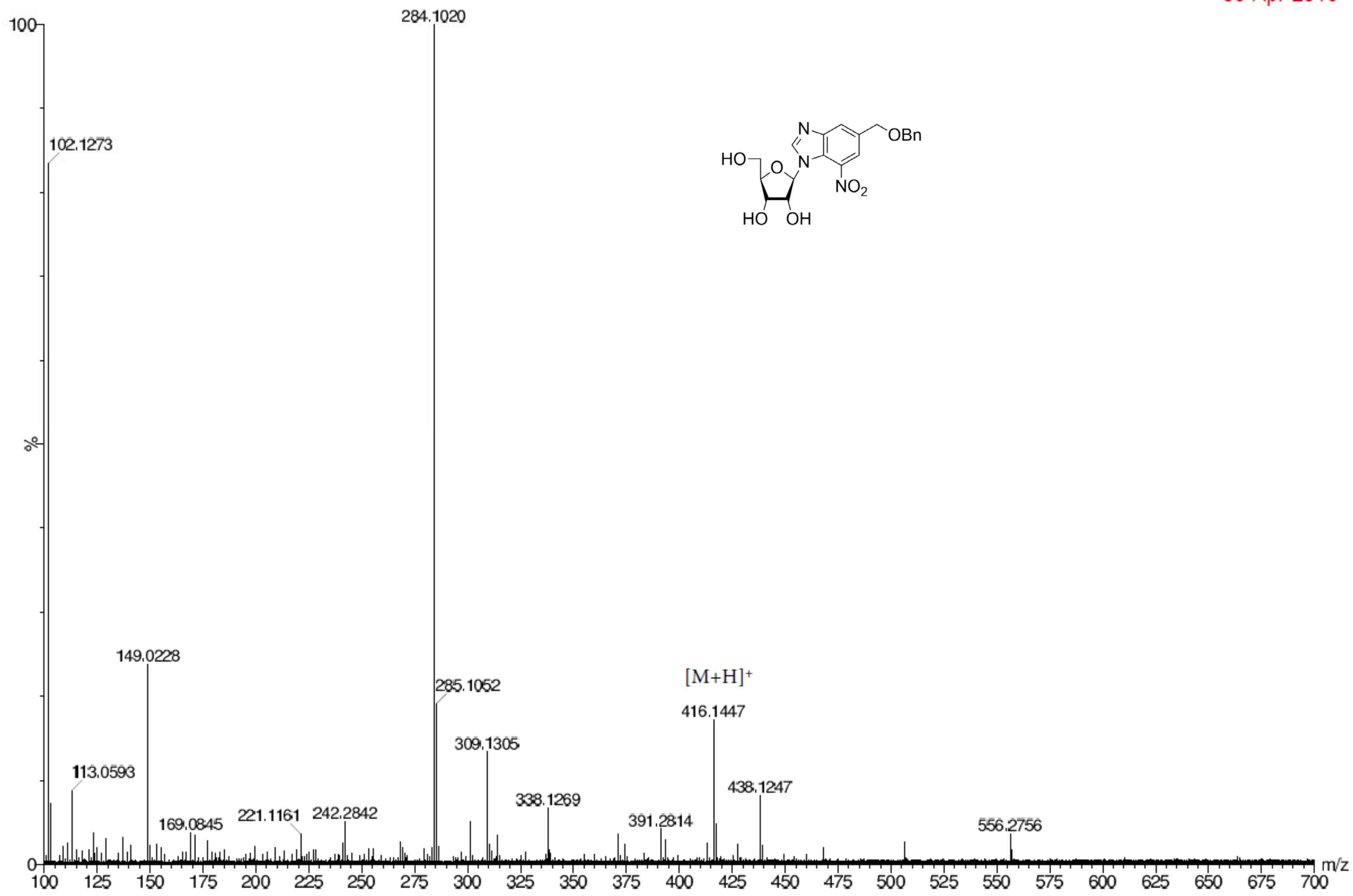

Compound 28 in CDCl3 1H spectrum - 300 MHz

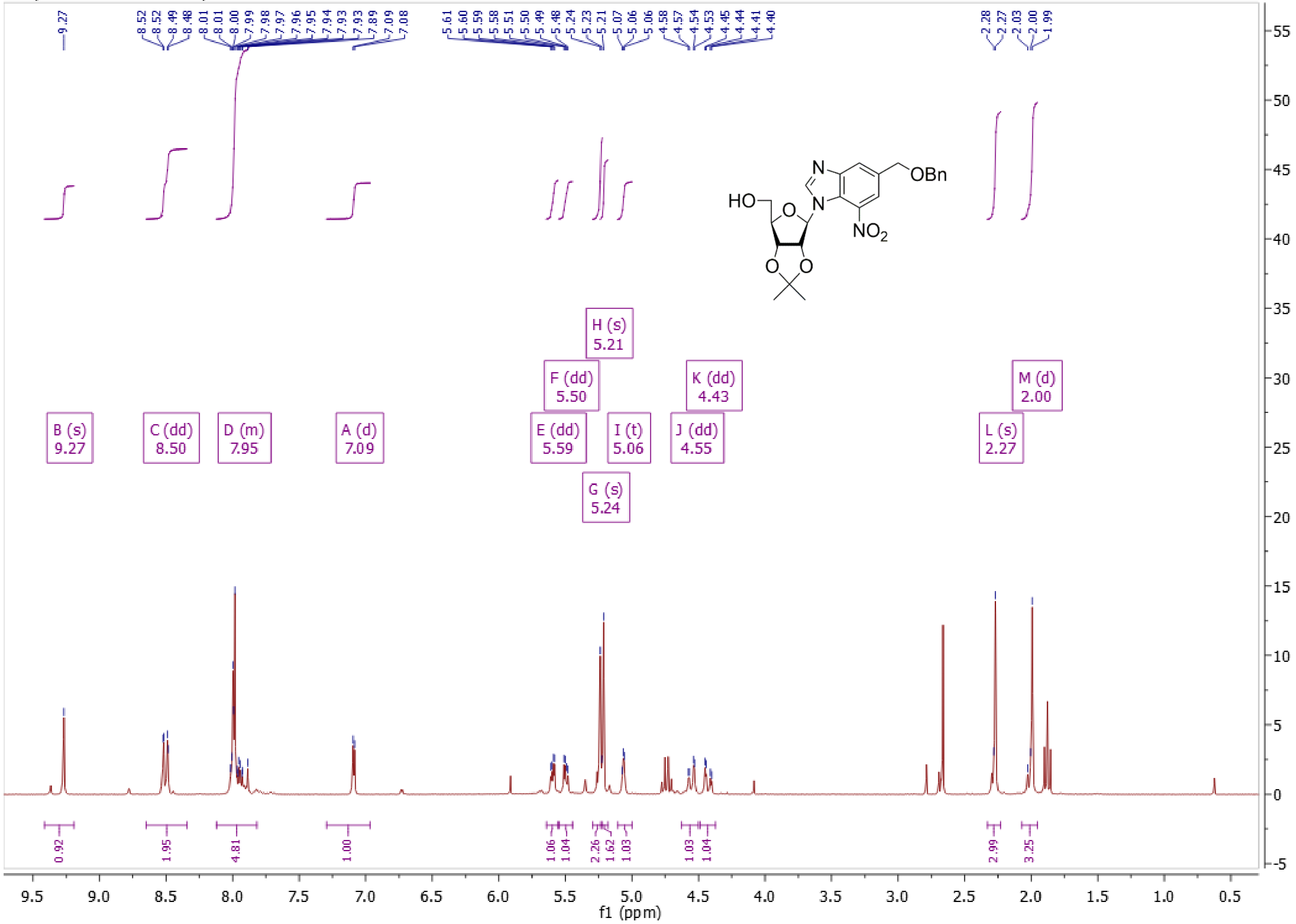

Compound 28 in CDCl3 13C spectrum - 75 MHz

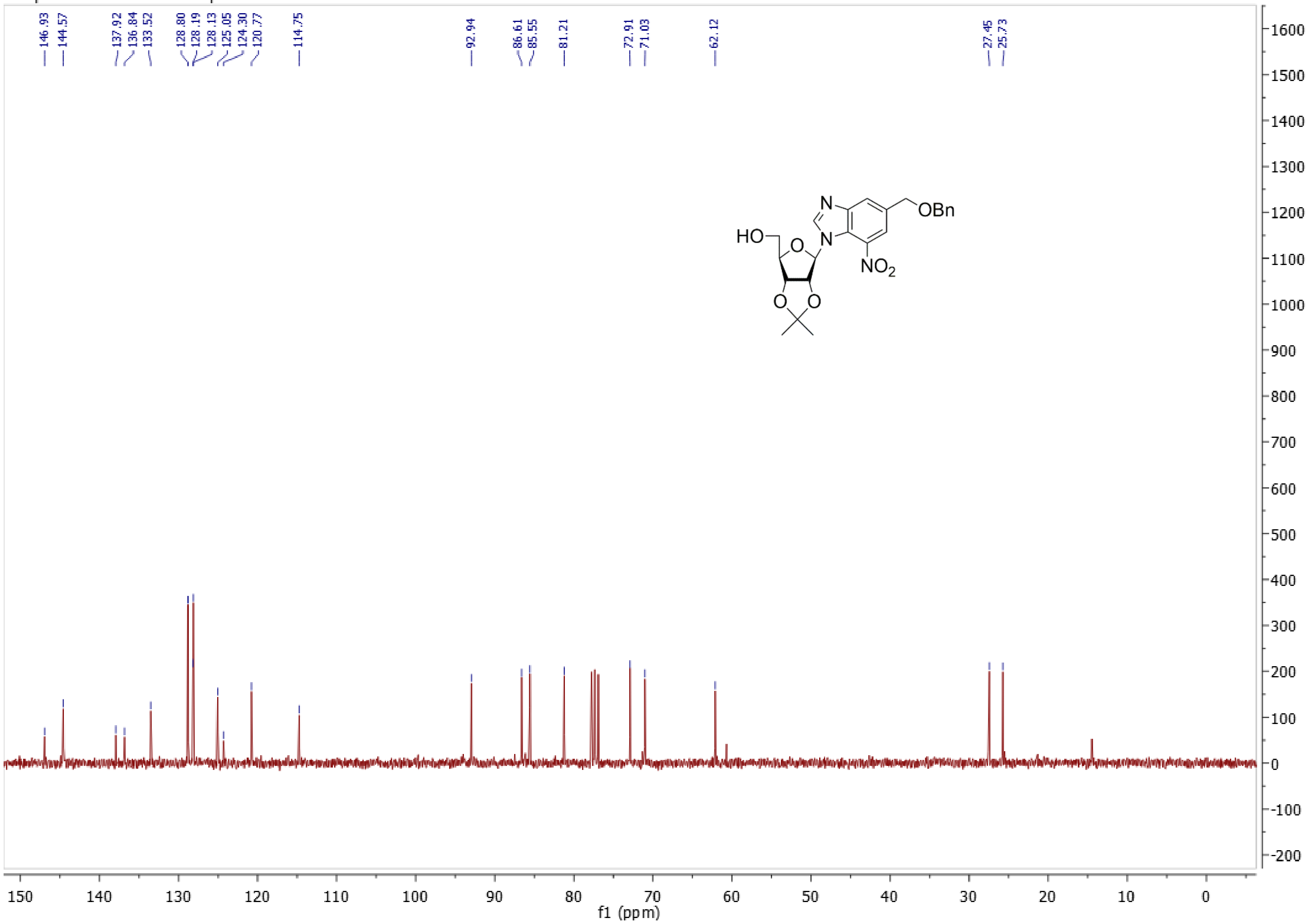

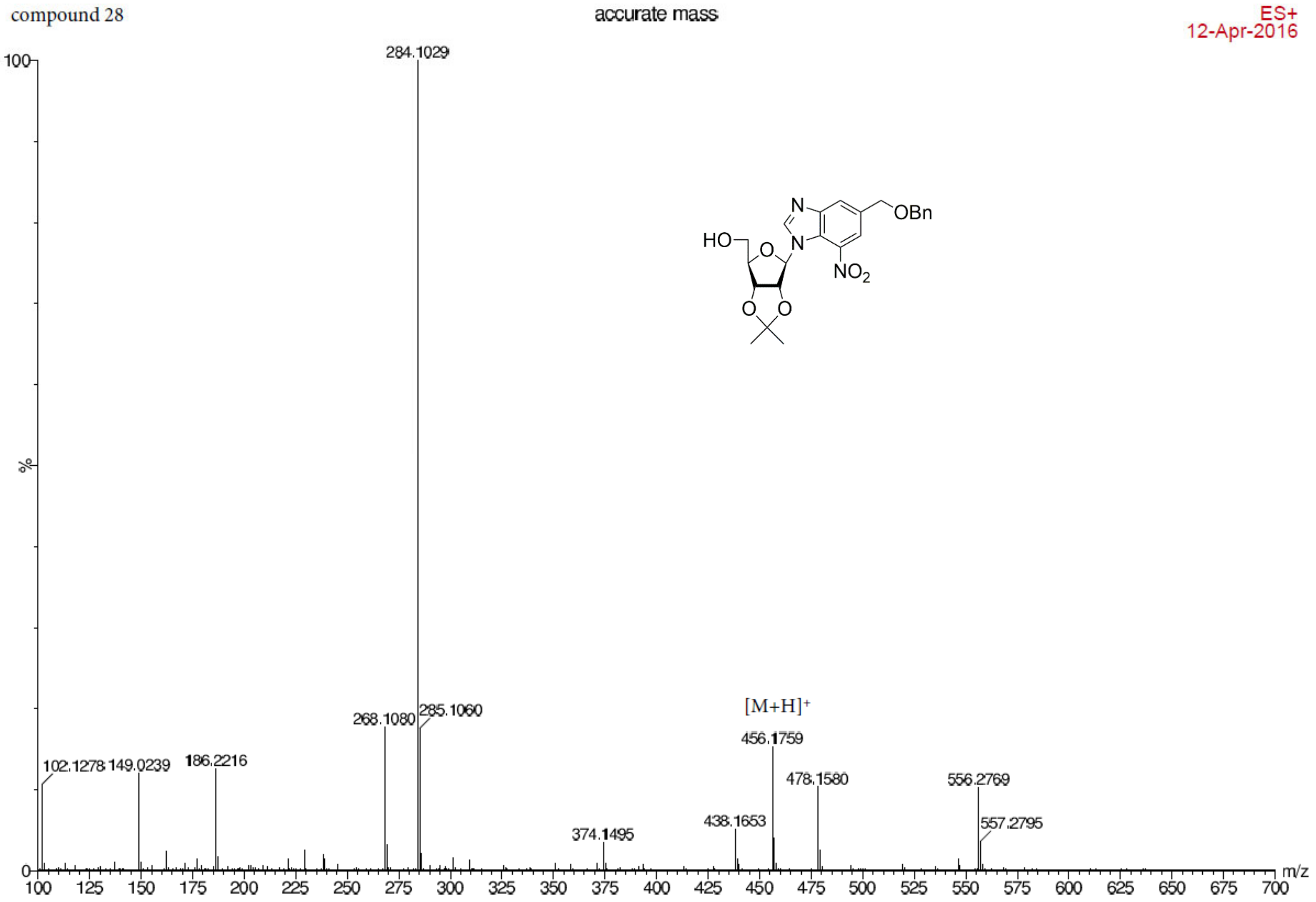
compound 28
accurate mass
ES+
12-Apr-2016
100
%
0
284.1029
268.1080
285.1060
102.1278
149.0239
186.2216
374.1495
438.1653
[M+H]+
456.1759
478.1580
556.2769
557.2795
100 125 150 175 200 225 250 275 300 325 350 375 400 425 450 475 500 525 550 575 600 625 650 675 700
m/z
HO
O
N
N
OBn
NO2
O
O

Compound 29 in DMSO-d6 1H spectrum - 300 MHz

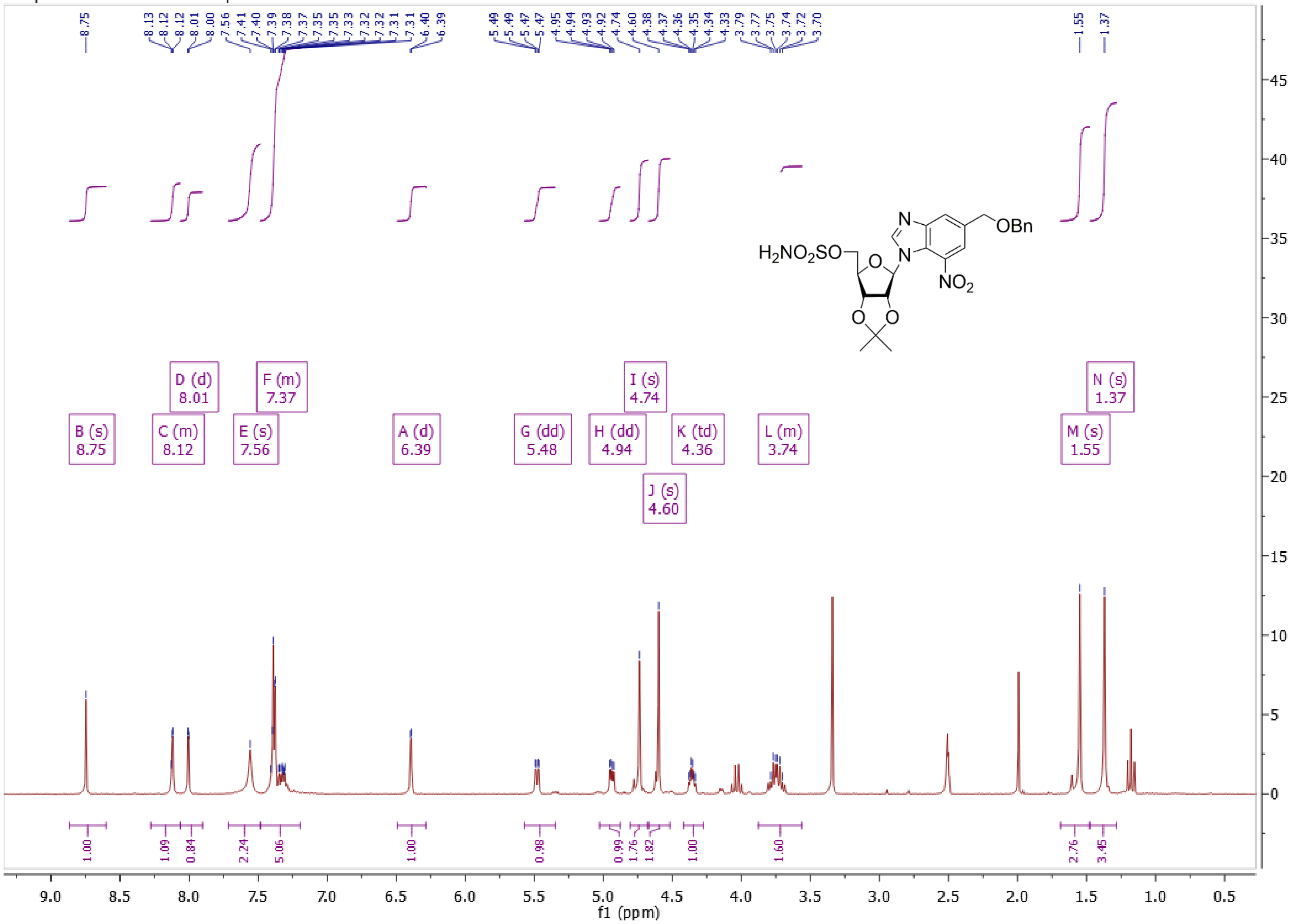

Compound 29 inDMSO-d6 13C spectrum - 75 MHz

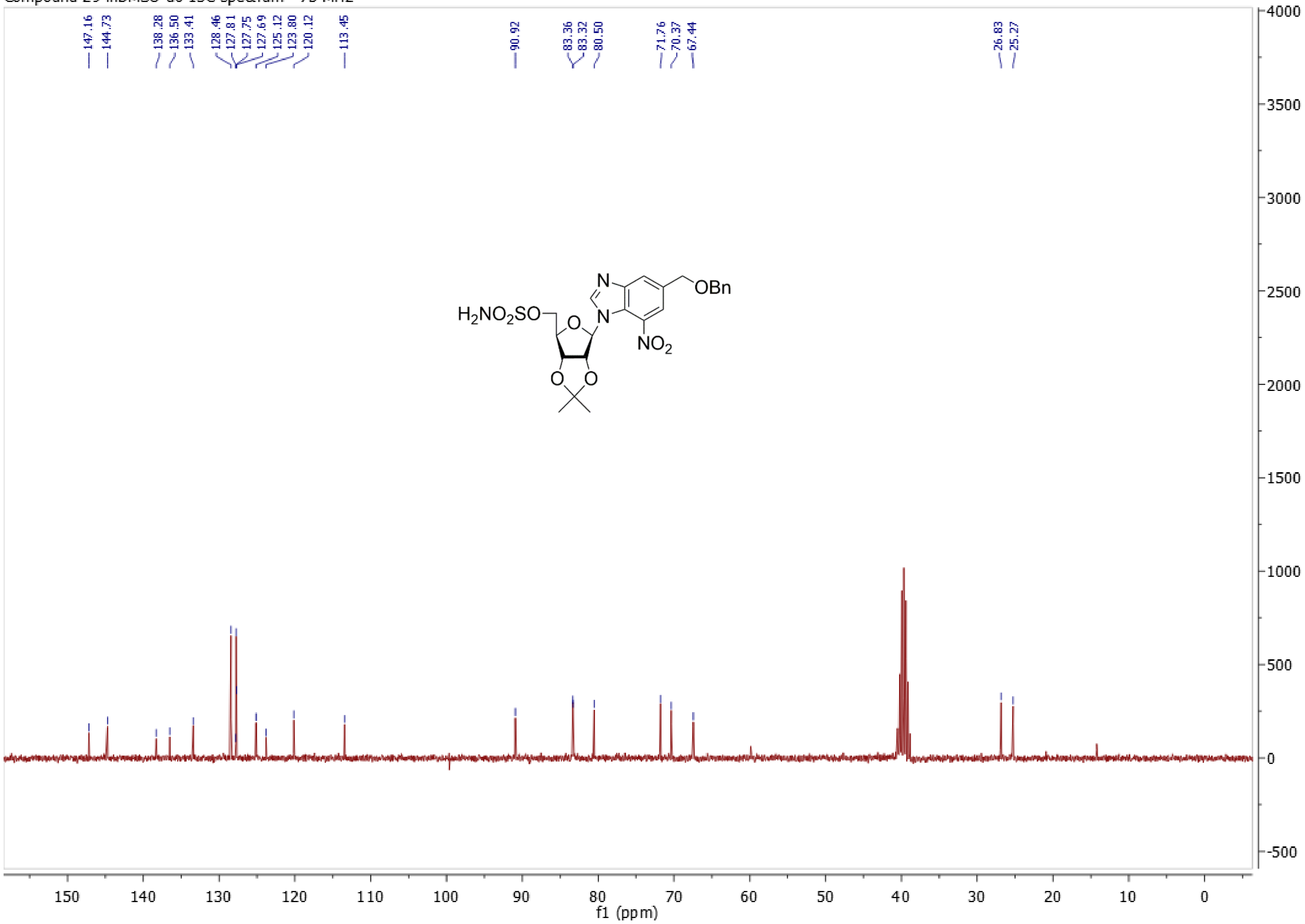

compound 29

accurate mass

ES+
12-Apr-2016

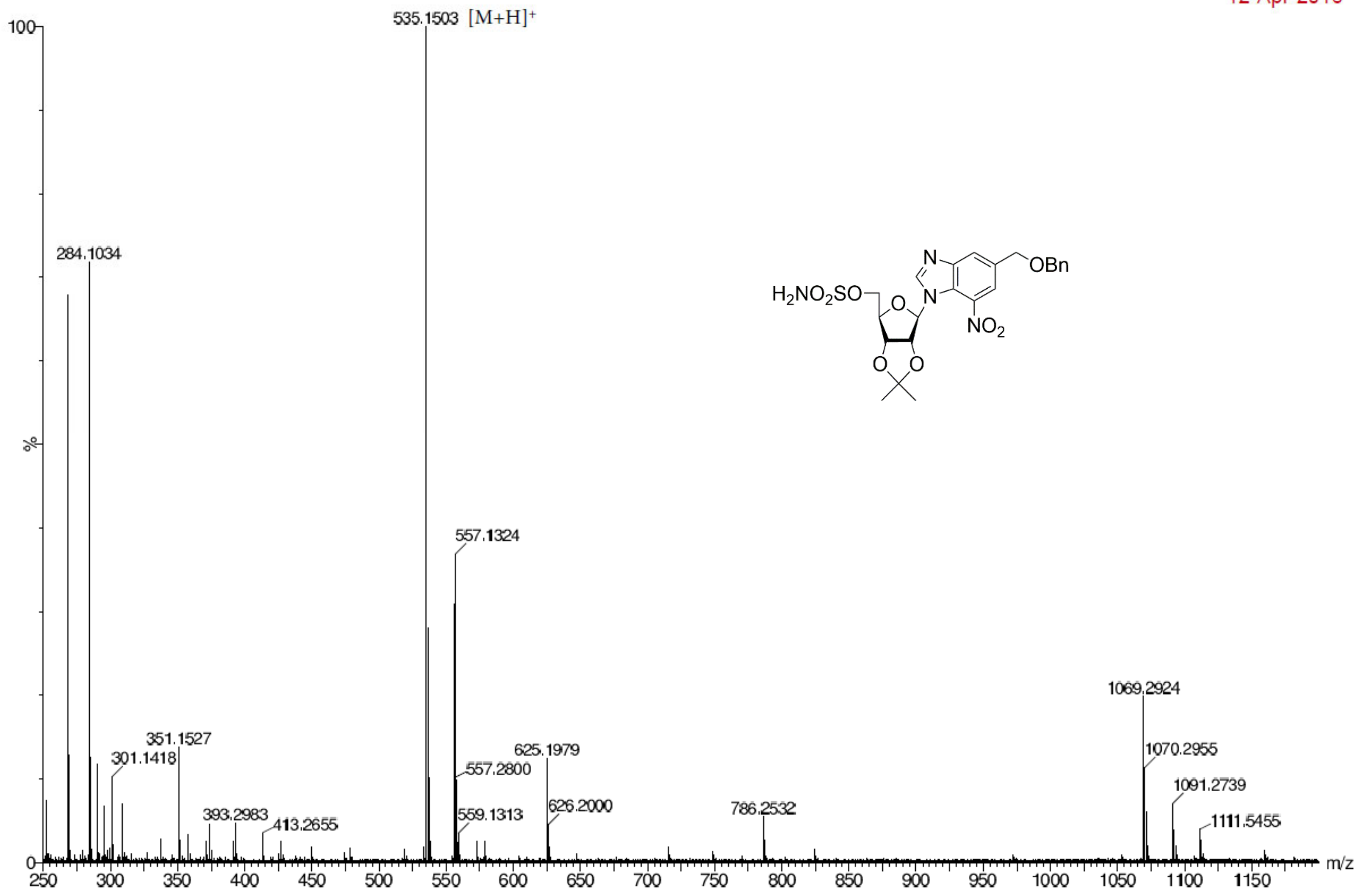

Compound 30a in CDCl3 1H spectrum - 300 MHz

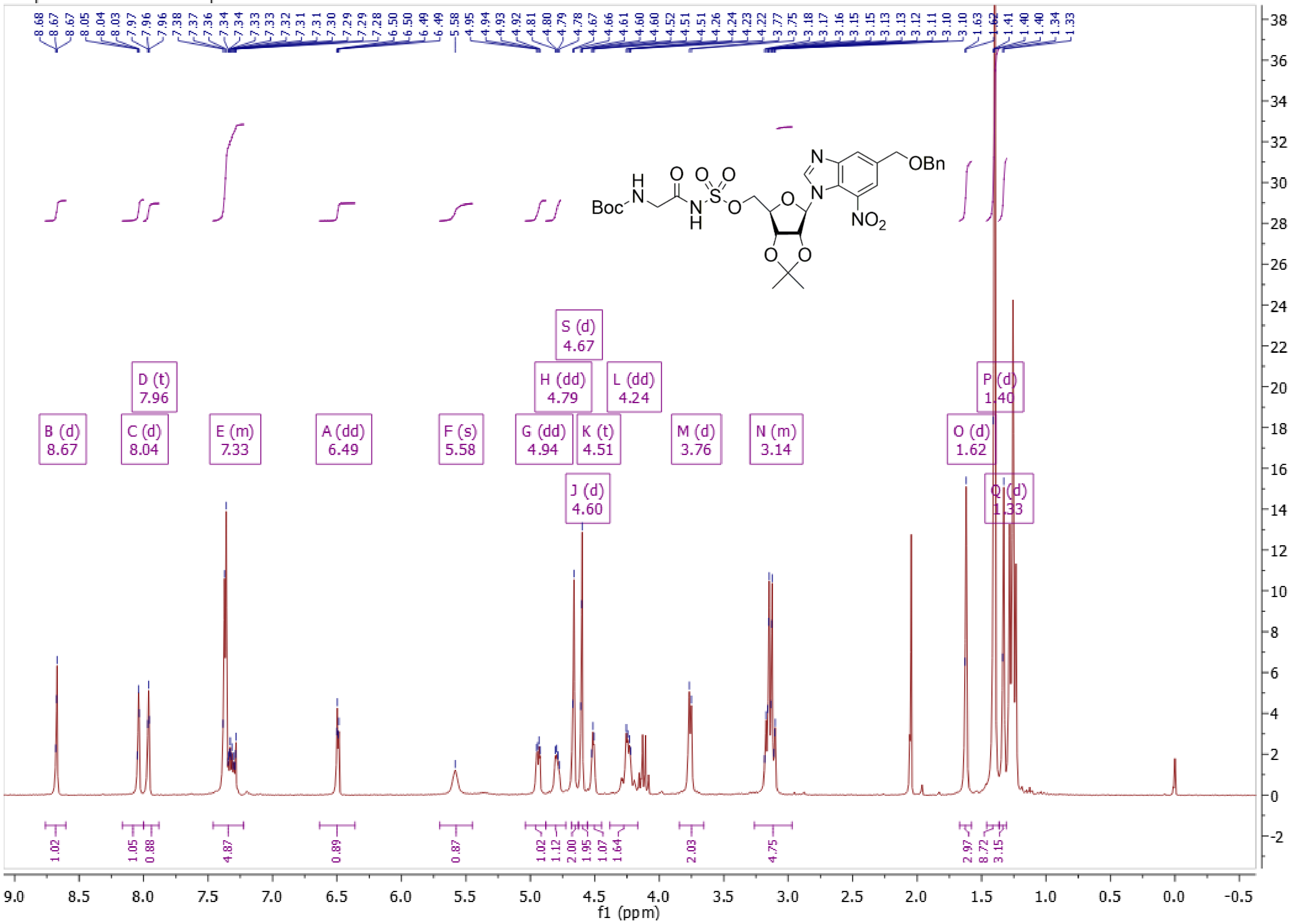

Compound 30a inDMSO-d6 13C spectrum - 75 MHz

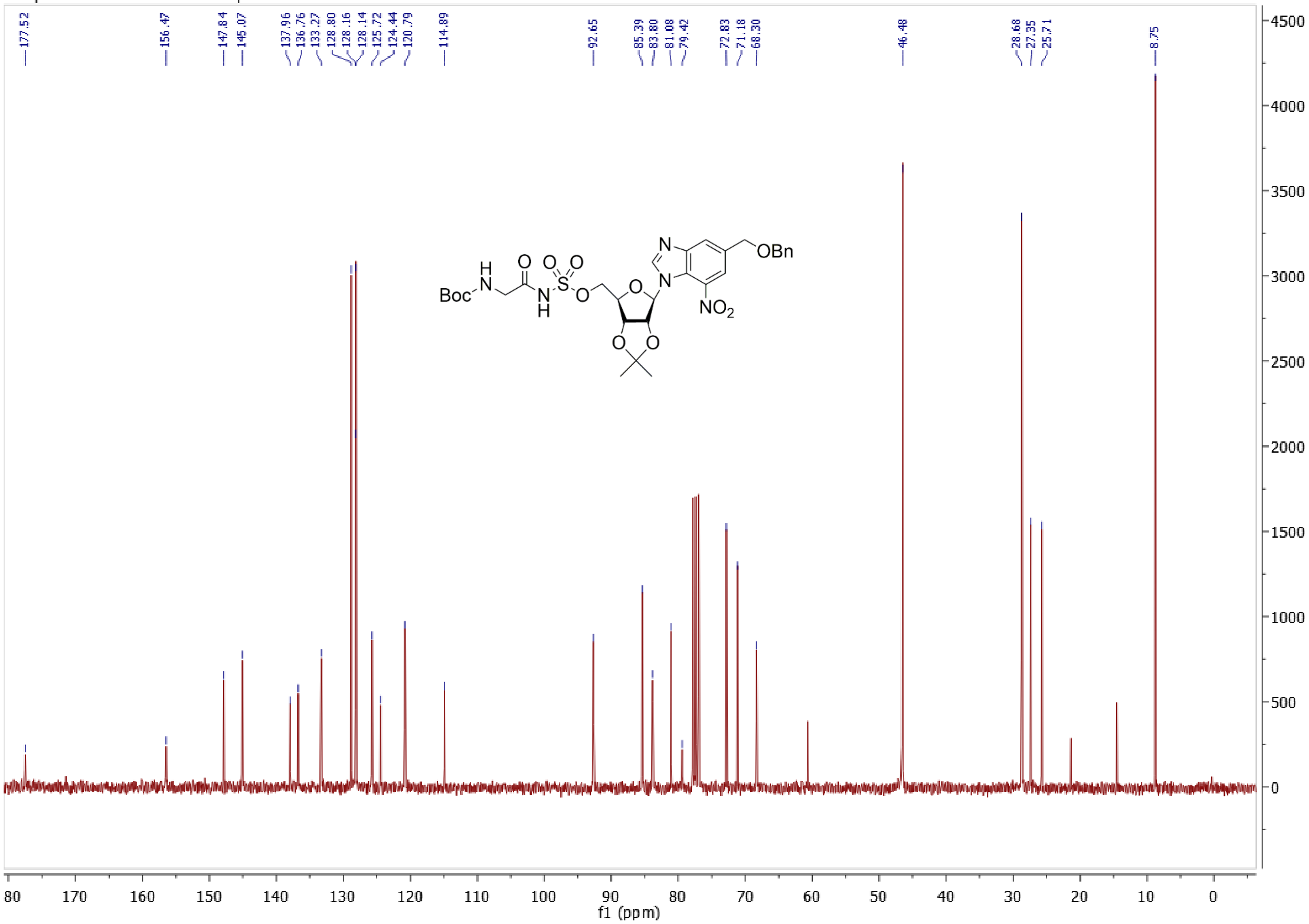

compound 30a

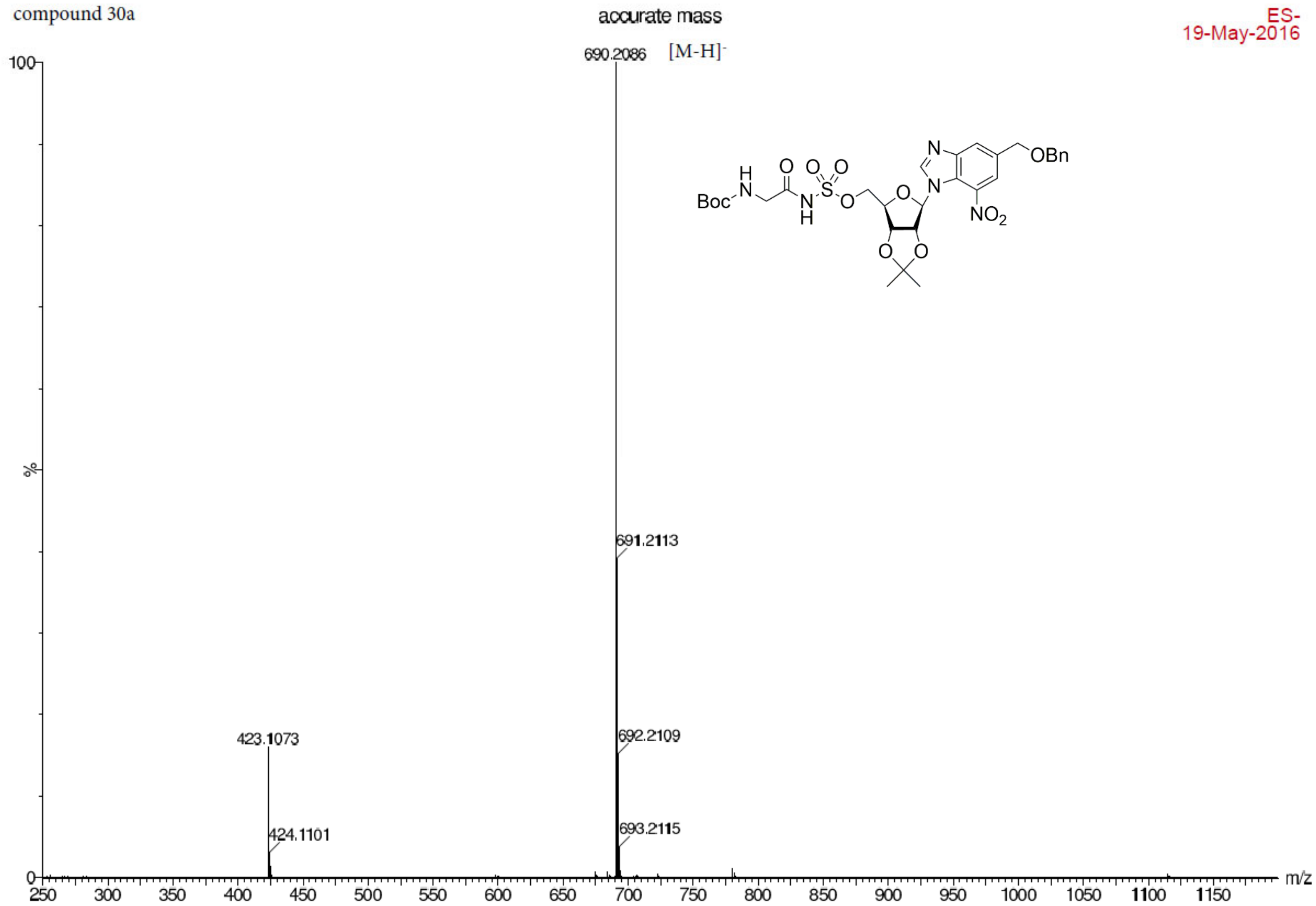

Compound 30b in DMSO-d6 1H spectrum - 300 MHz

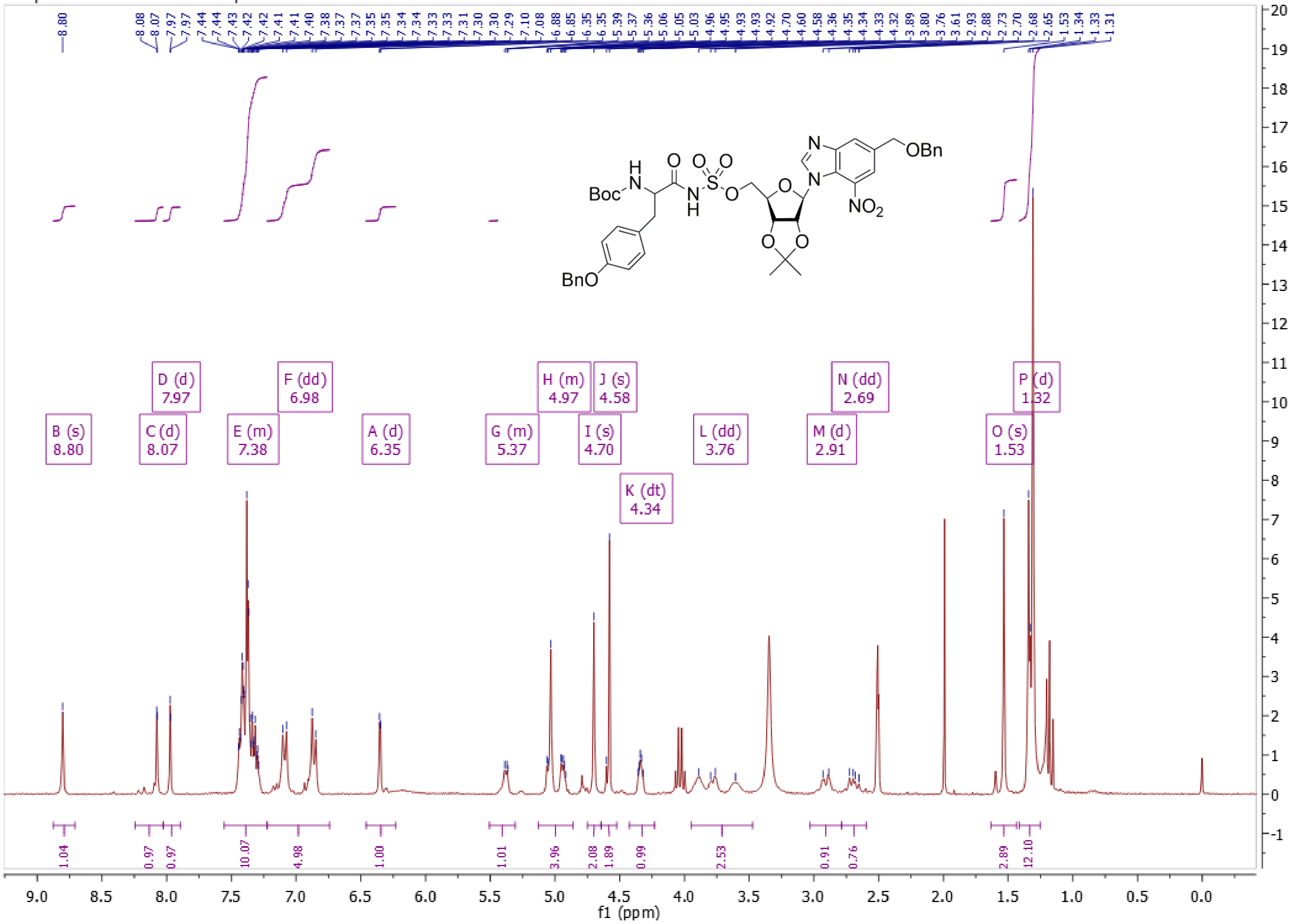

Compound 30b inDMSO-d6 13C spectrum - 75 MHz

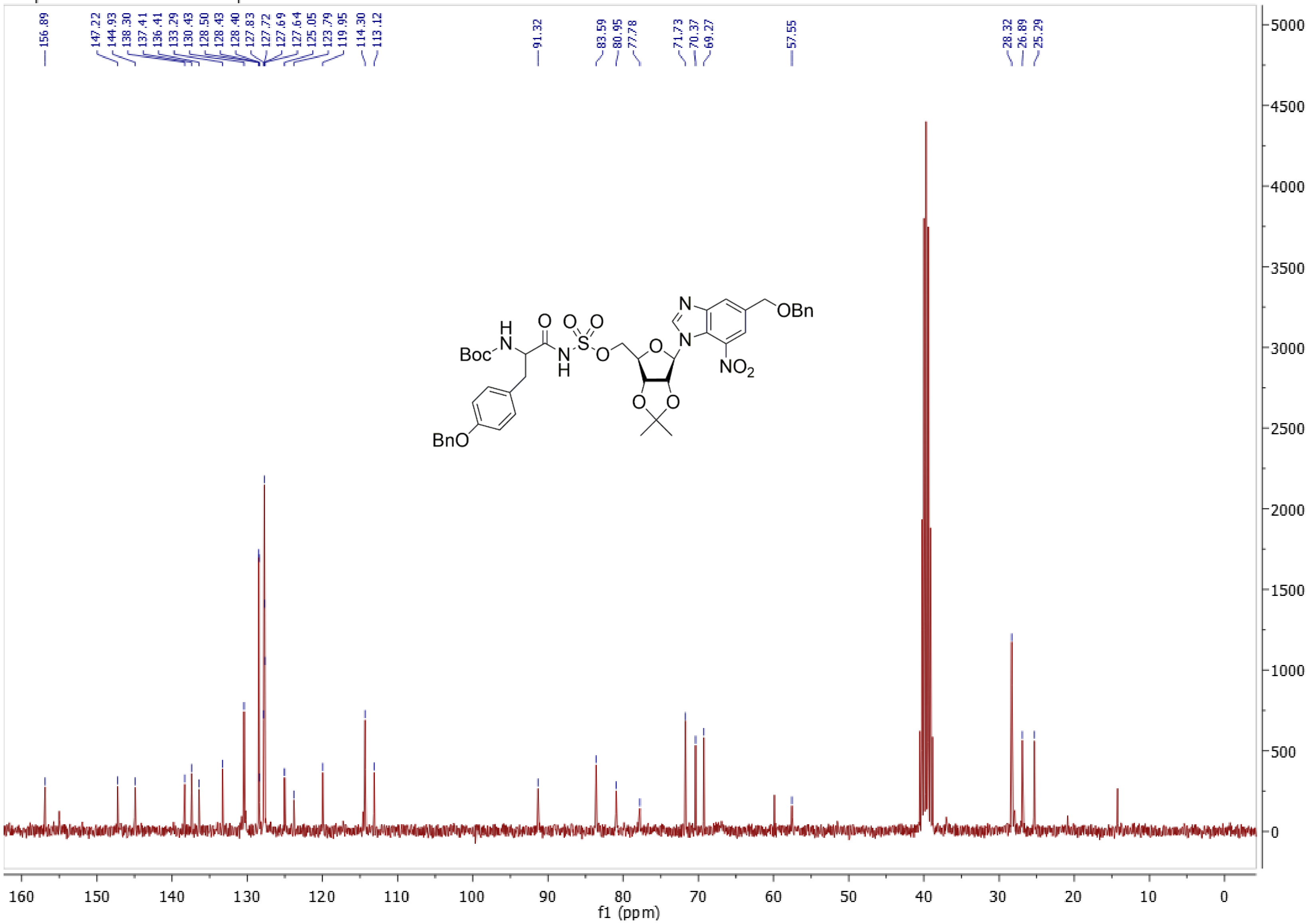

compound 30b

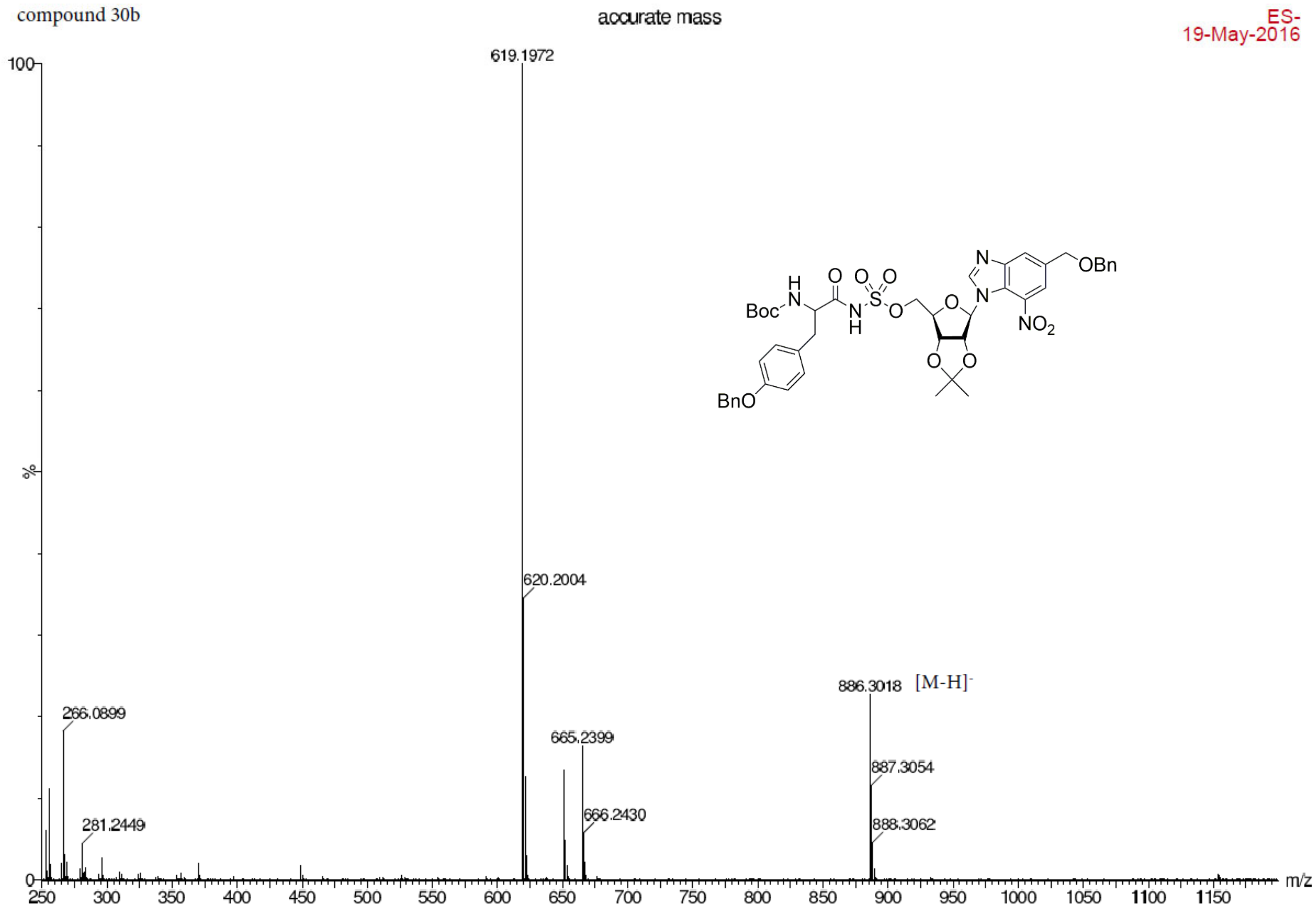

Compound 30c in DMSO-d6 1H spectrum - 300 MHz

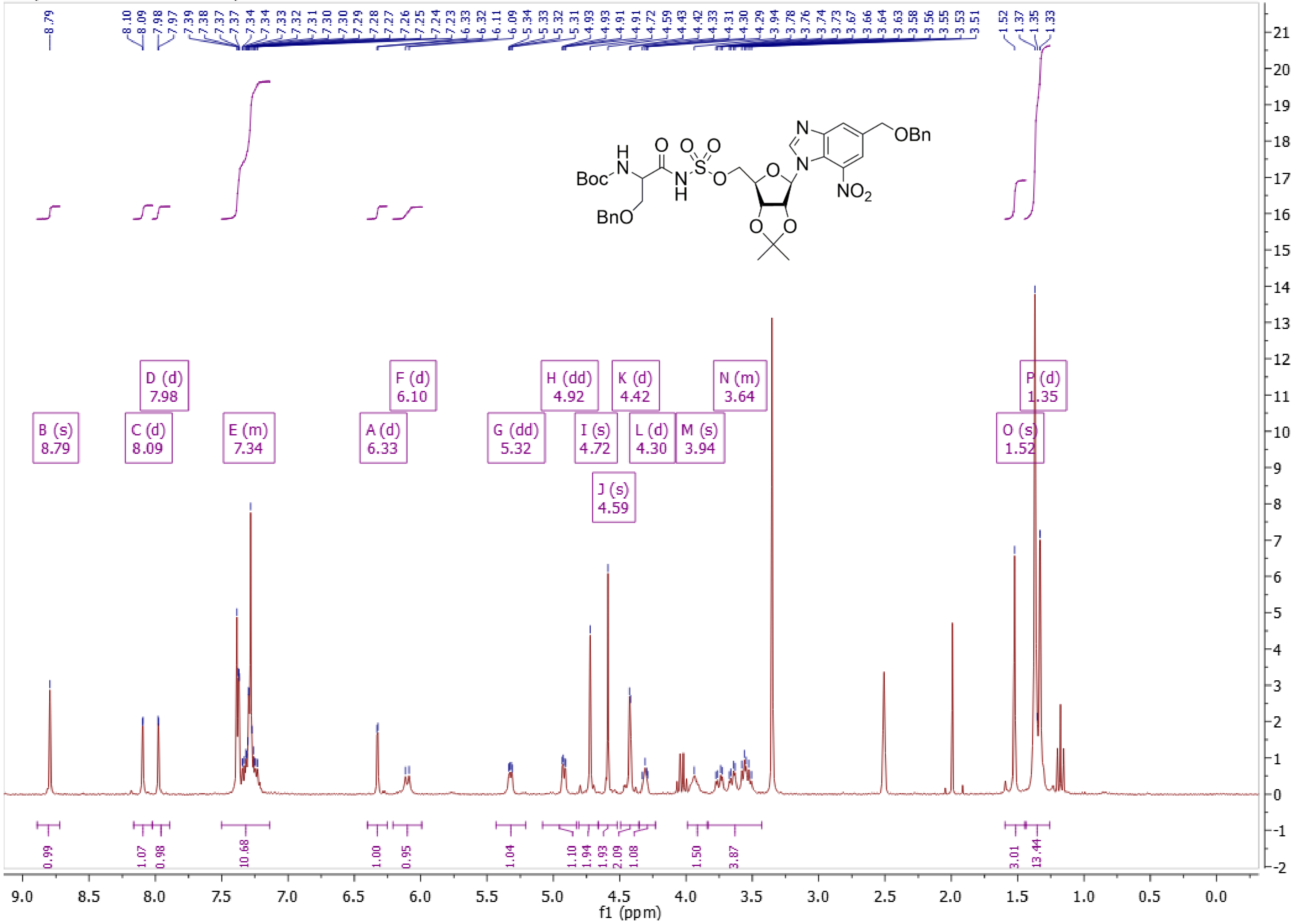

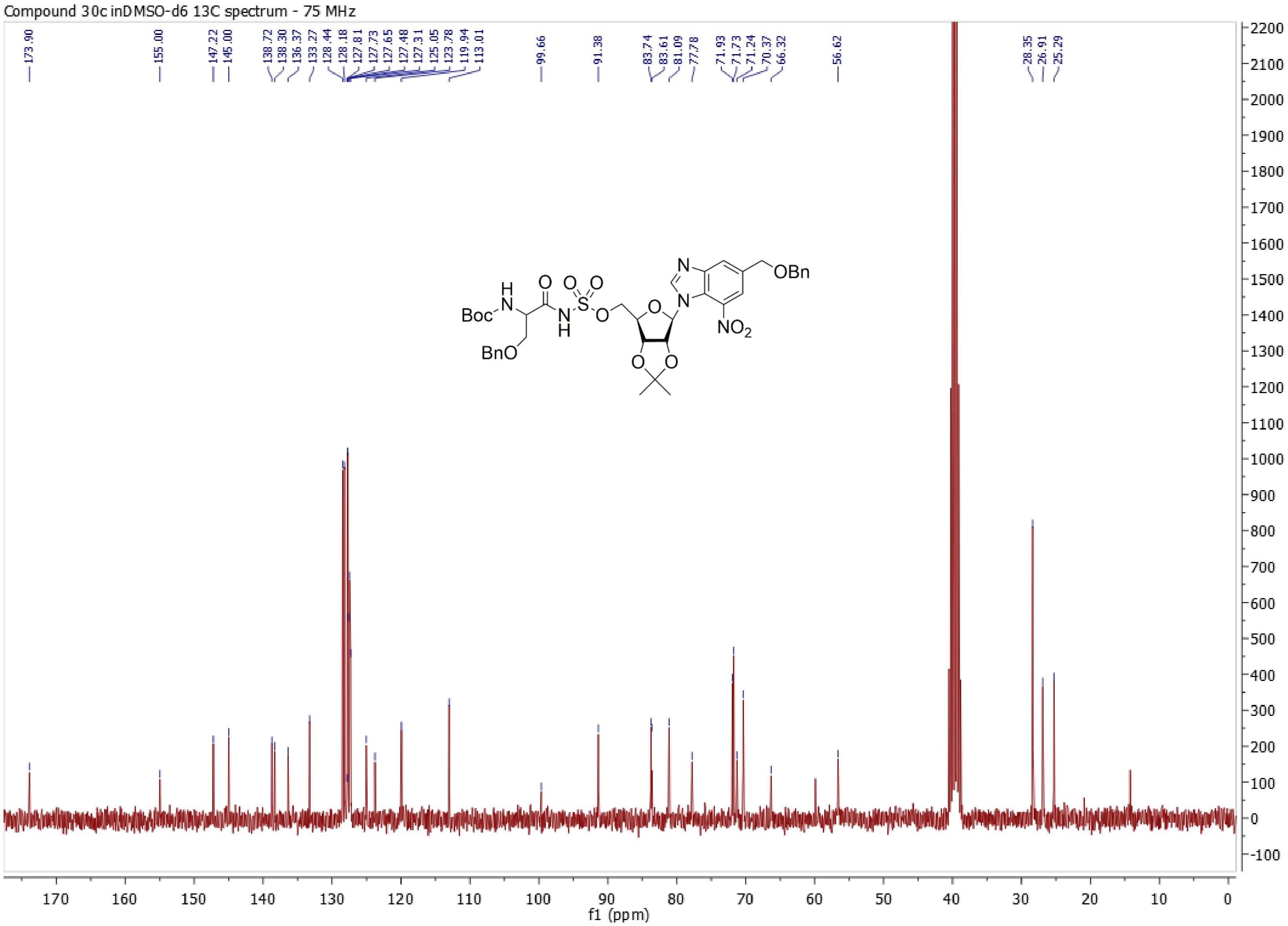
Compound 30c inDMSO-d6 13C spectrum - 75 MHz
173.90
155.00
147.22
145.00
138.72
138.30
136.37
133.27
128.44
128.18
127.81
127.73
127.65
127.48
127.31
125.05
123.78
119.94
113.01
99.66
91.38
83.74
83.61
81.09
77.78
71.93
71.73
71.24
70.37
66.32
56.62
28.35
26.91
25.29
f1 (ppm)
Boc
BnO
OBn
NO2

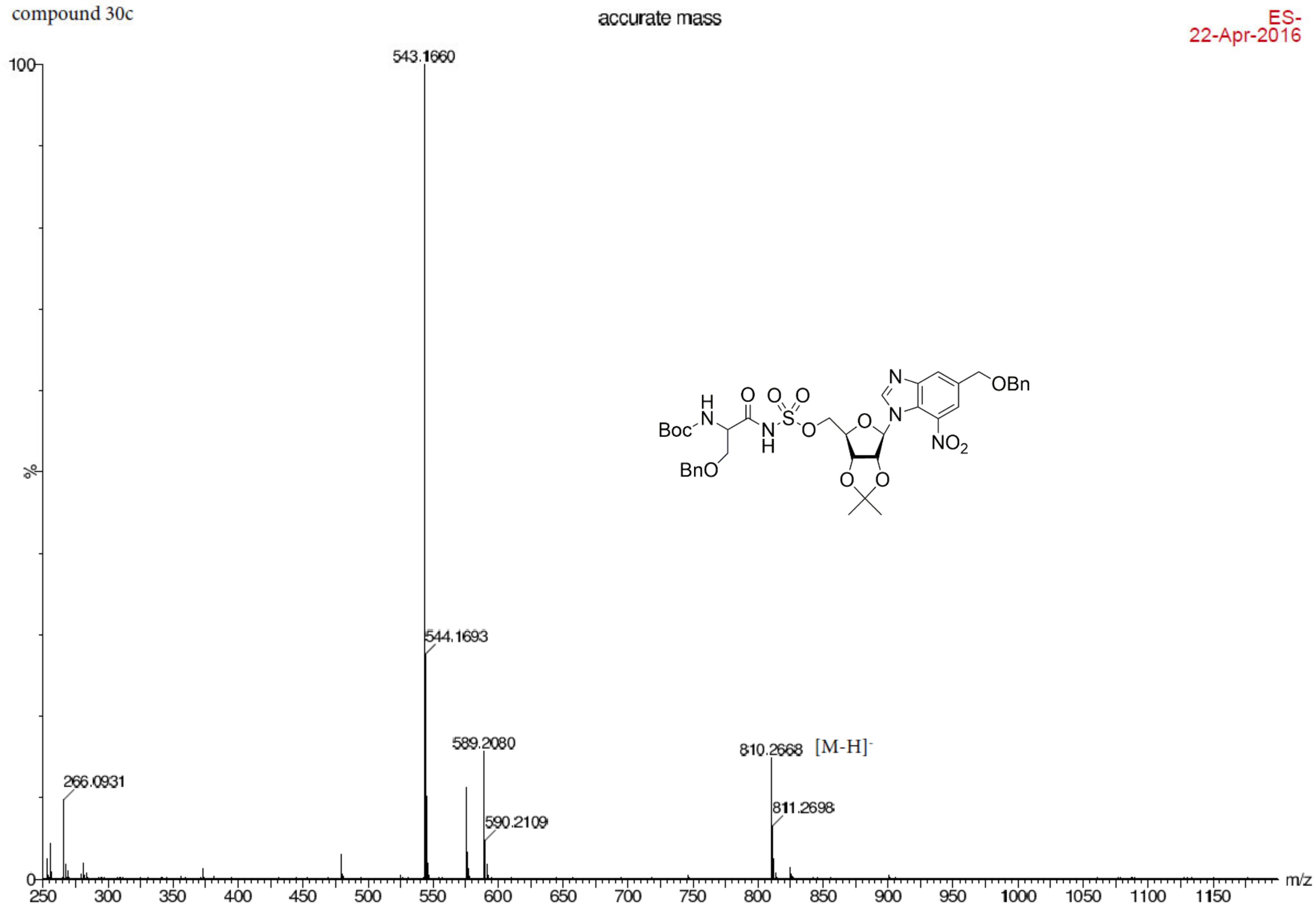

compound 30c
accurate mass
ES-
22-Apr-2016
100
%
0
543.1660
544.1693
589.2080
590.2109
266.0931
810.2668 [M-H]-
811.2698
250
300
350
400
450
500
550
600
650
700
750
800
850
900
950
1000
1050
1100
1150
m/z
Boc
H
N
O
O
O
N
S
O
H
BnO
O
O
O
N
N
OBn
NO2

Compound 30d in DMSO-d6 1H spectrum - 300 MHz

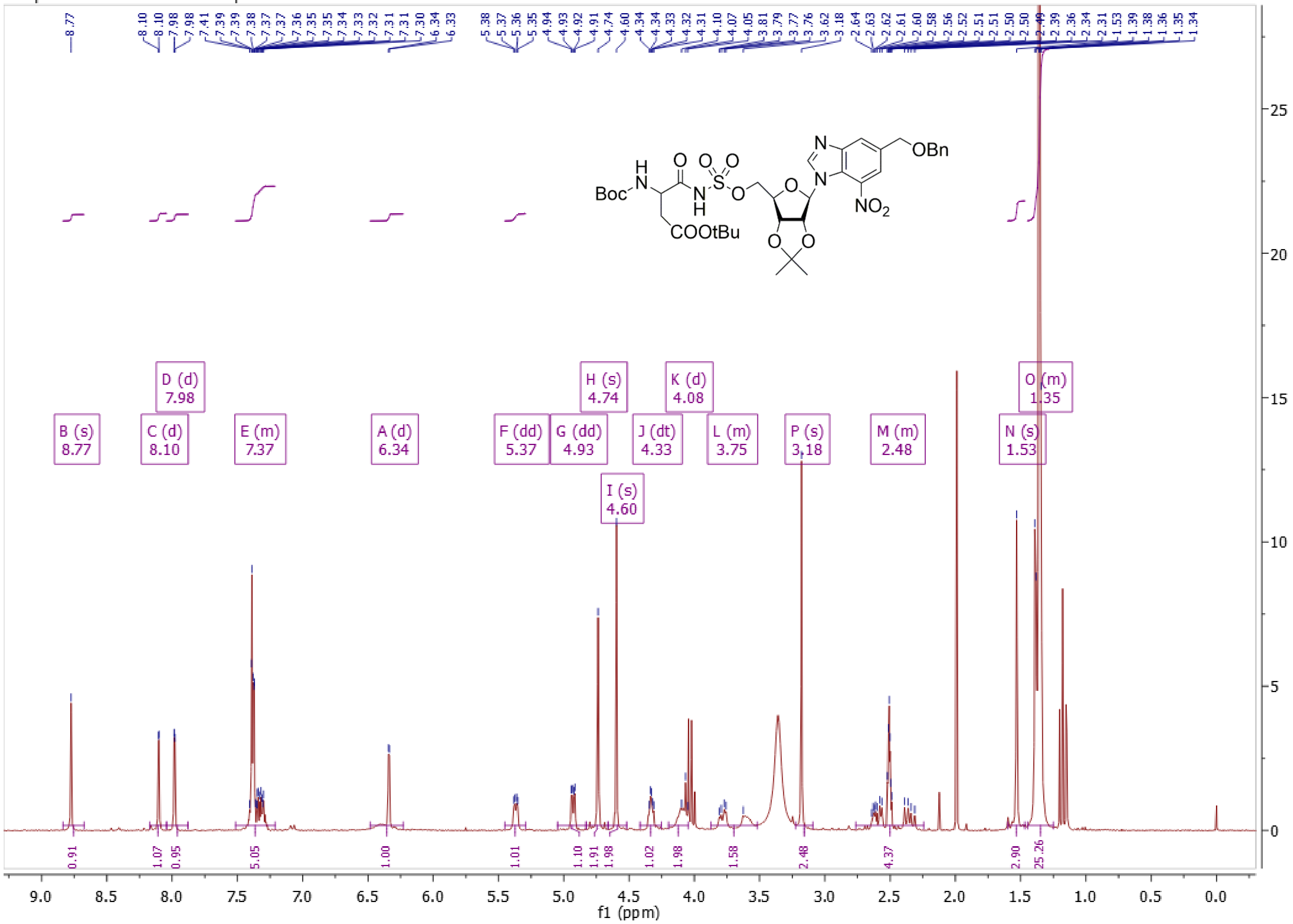

Compound 30d inDMSO-d6 13C spectrum - 75 MHz

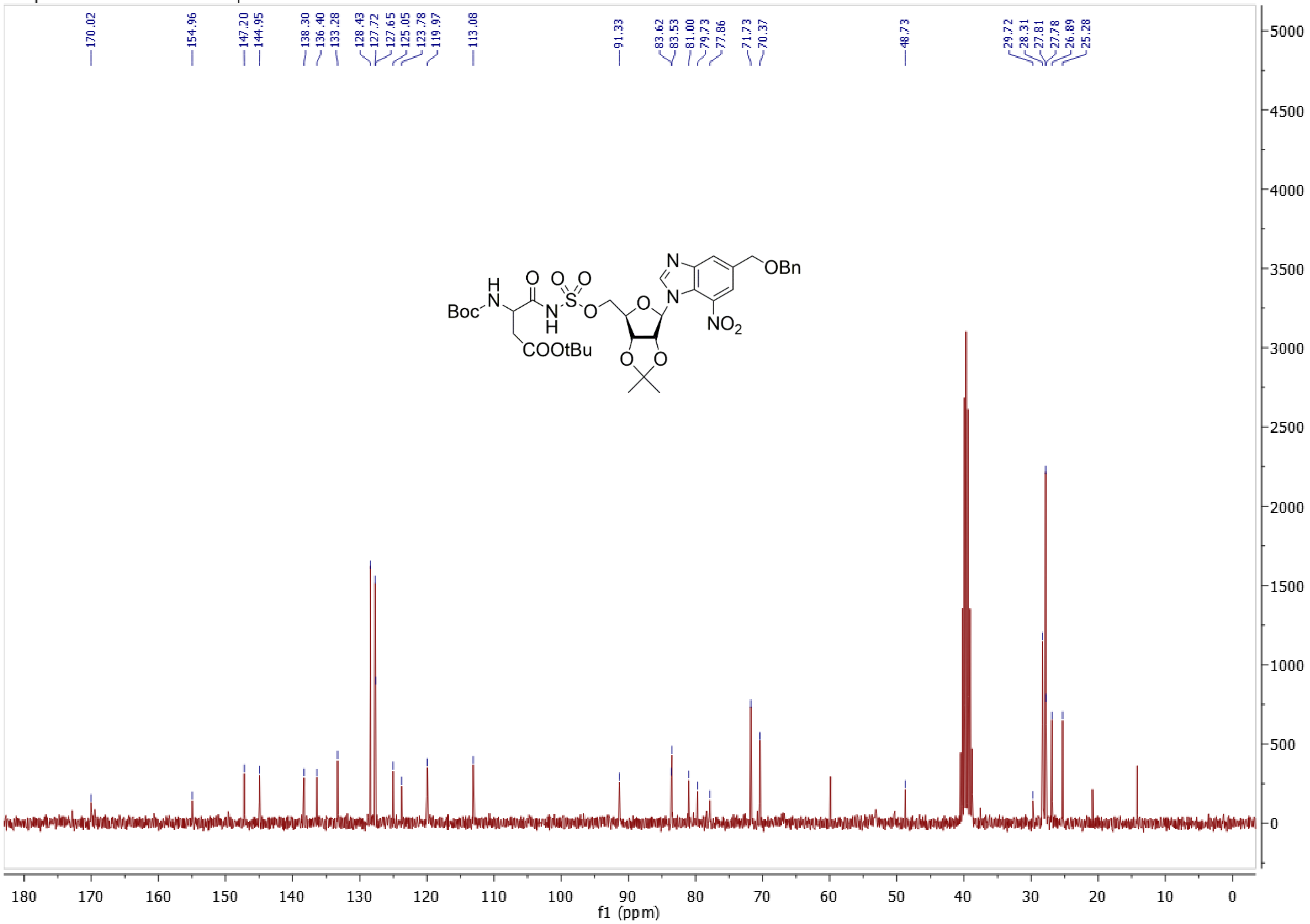

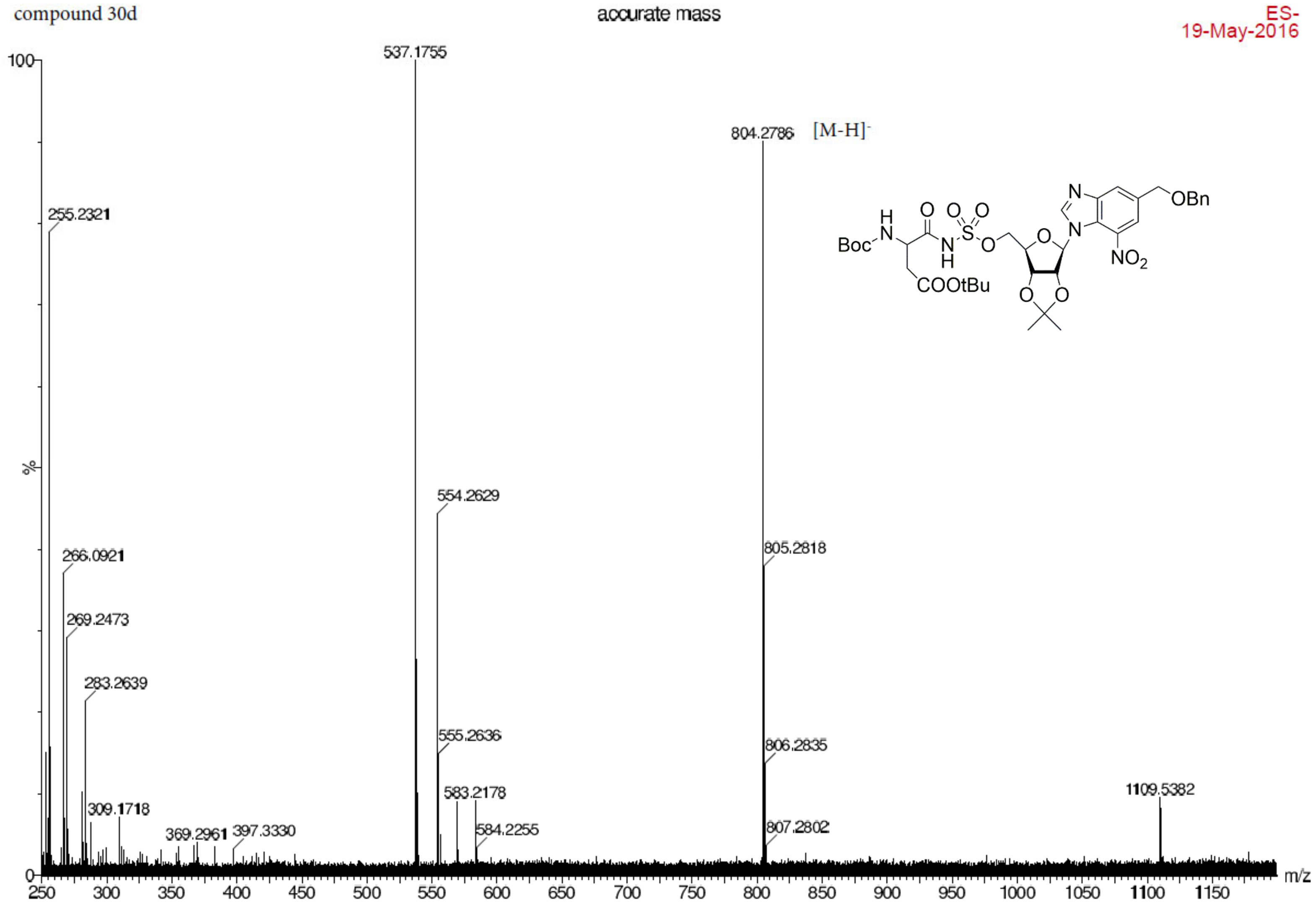
compound 30d
accurate mass
ES-
19-May-2016
537.1755
804.2786 [M-H]-
255.2321
554.2629
266.0921
805.2818
269.2473
283.2639
555.2636
806.2835
583.2178
1109.5382
309.1718
369.2961
397.3330
584.2255
807.2802
%
m/z
Boc
COOtBu
OBn
NO2

Compound 30e in DMSO-d6 1H spectrum - 300 MHz

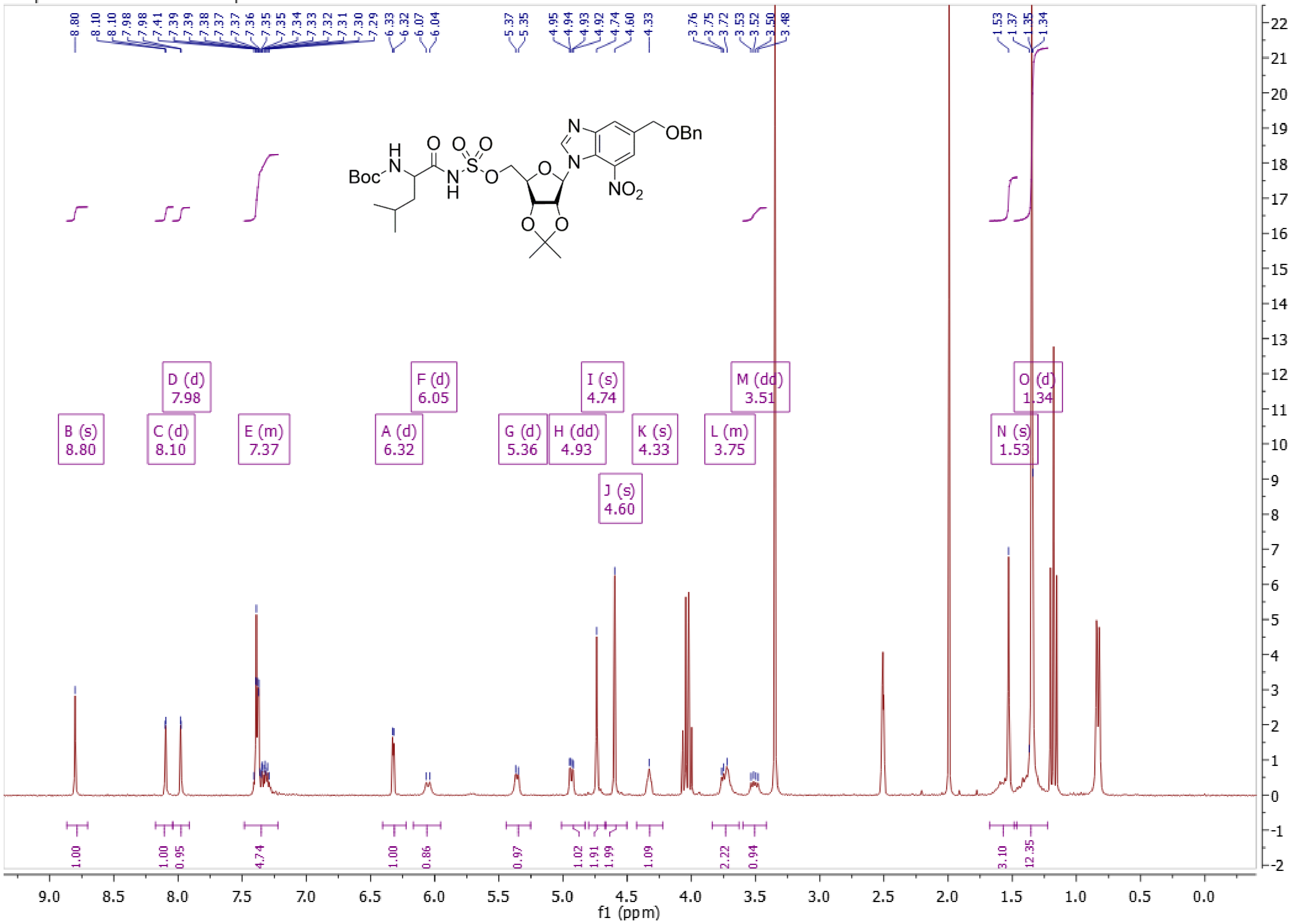

Compound 30e inDMSO-d6 13C spectrum - 75 MHz

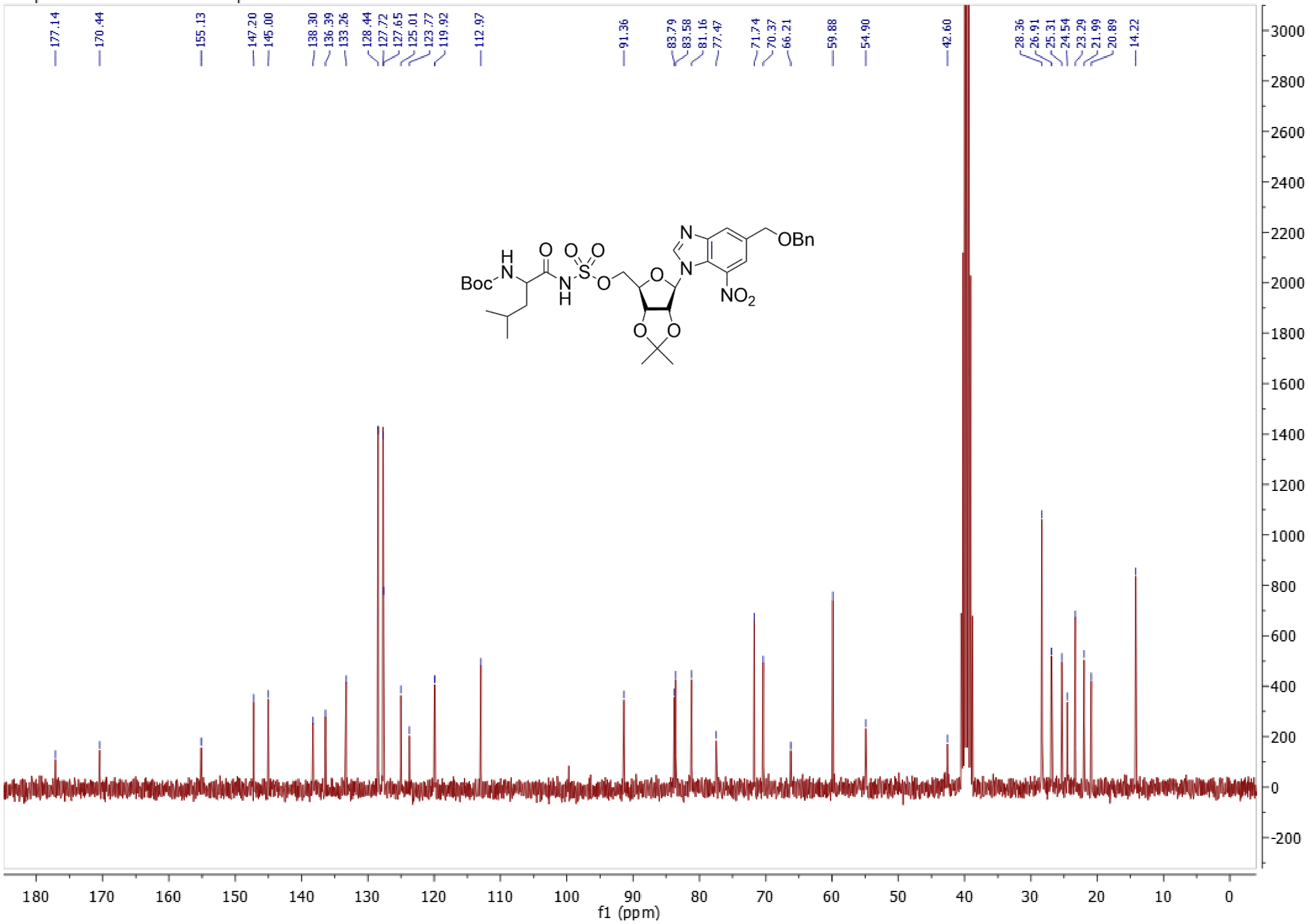

compound 30e

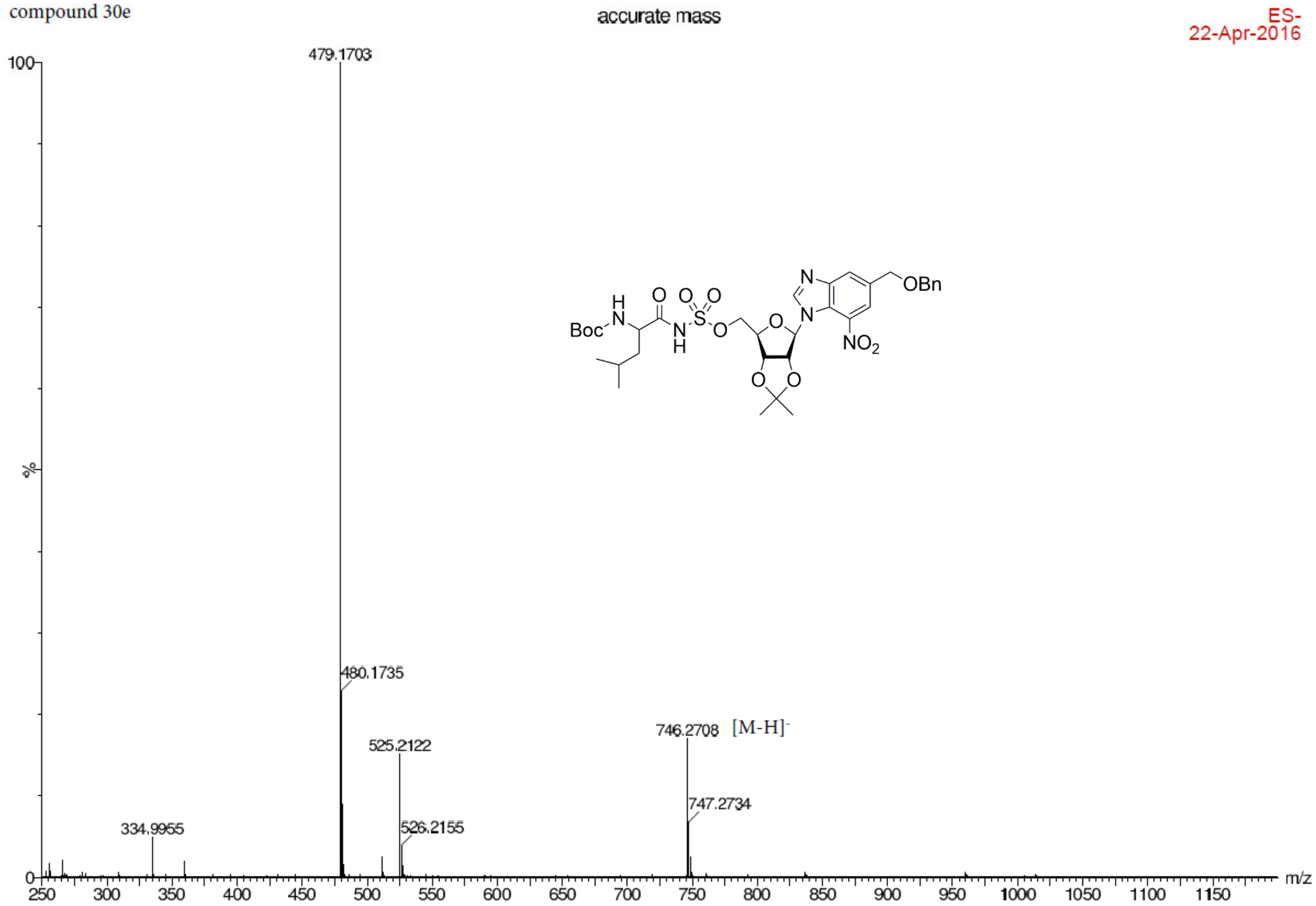

Compound 30f inDMSO-d6 13C spectrum - 75 MHz

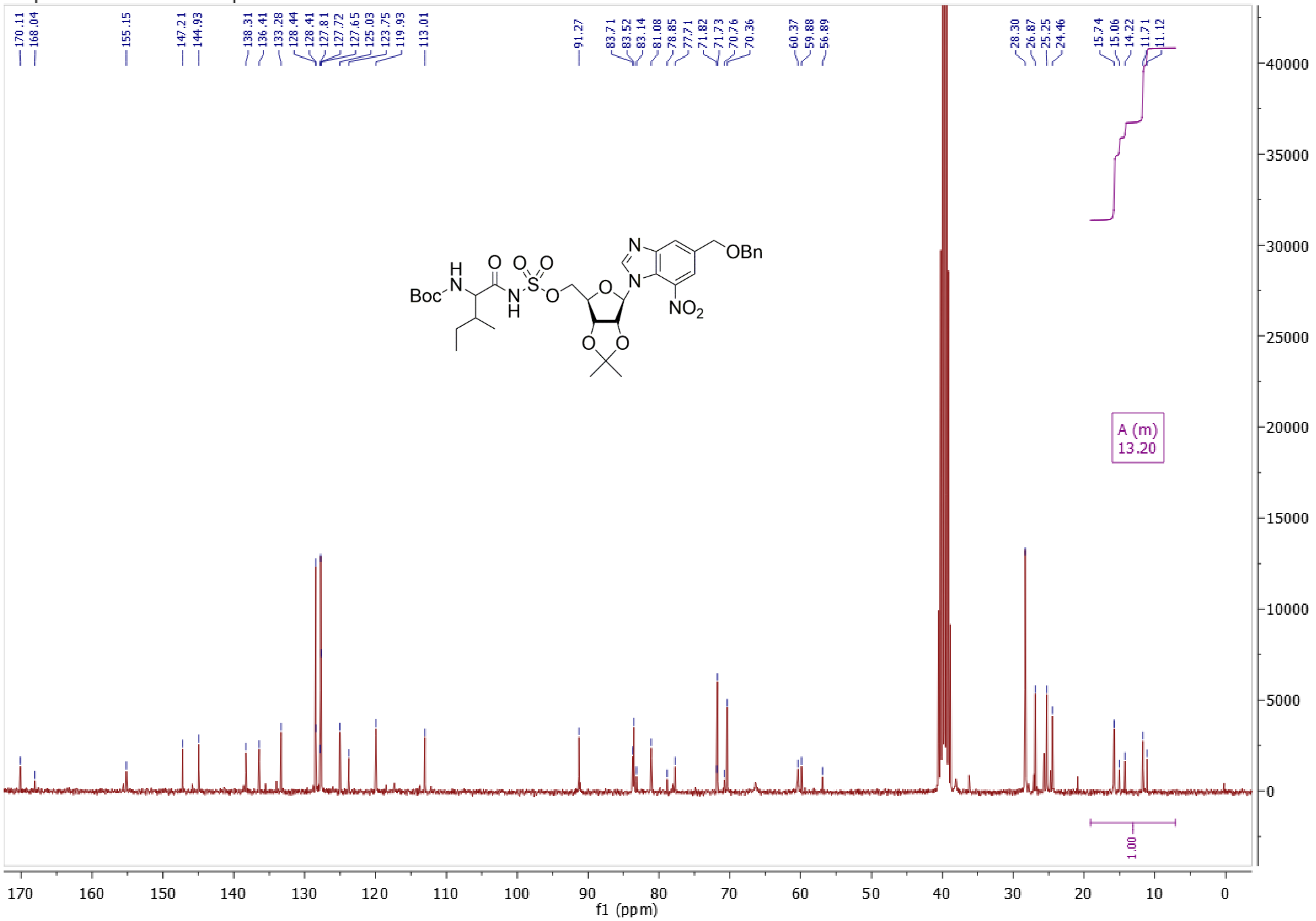

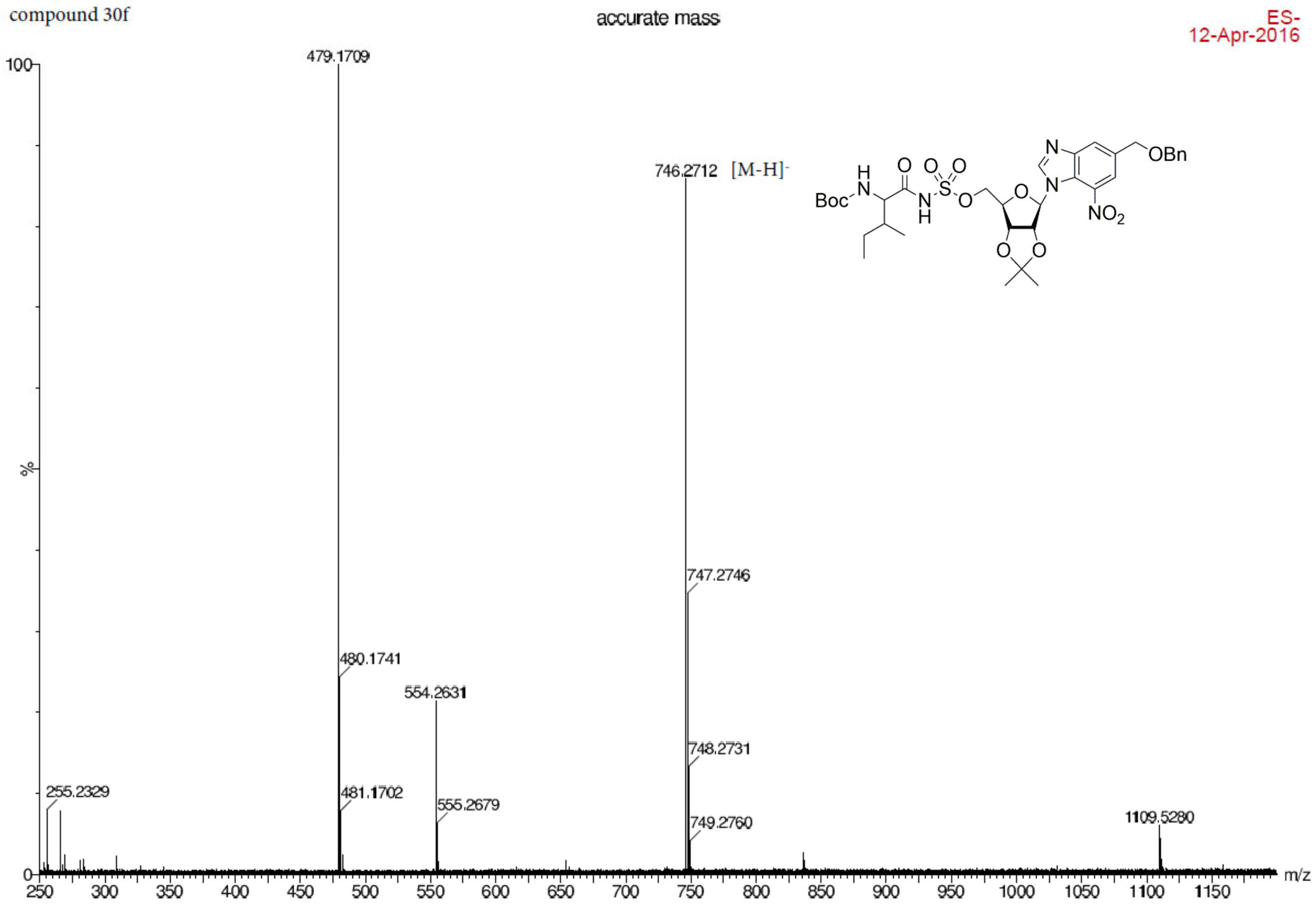
compound 30f
accurate mass
ES-
12-Apr-2016
100
%
0
479.1709
746.2712 [M-H]-
747.2746
480.1741
554.2631
748.2731
255.2329
481.1702
555.2679
749.2760
1109.5280
250
300
350
400
450
500
550
600
650
700
750
800
850
900
950
1000
1050
1100
1150
m/z
Boc
H
N
O
O
O
S
N
H
O
O
N
N
OBn
NO2
O
O

Compound 31a in MeOD 1H spectrum - 300 MHz

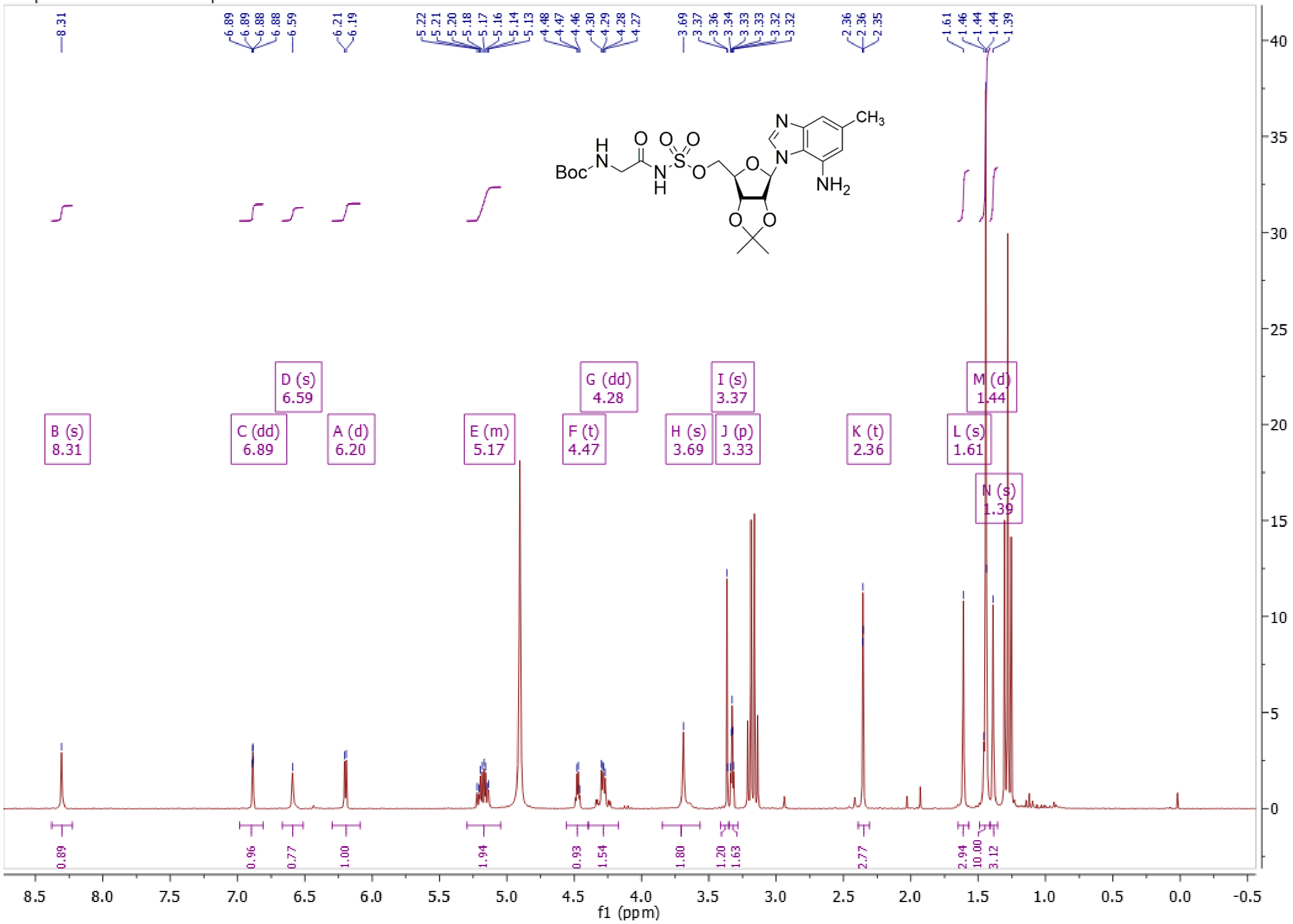

Compound 31a in MeOD 13C spectrum - 75 MHz

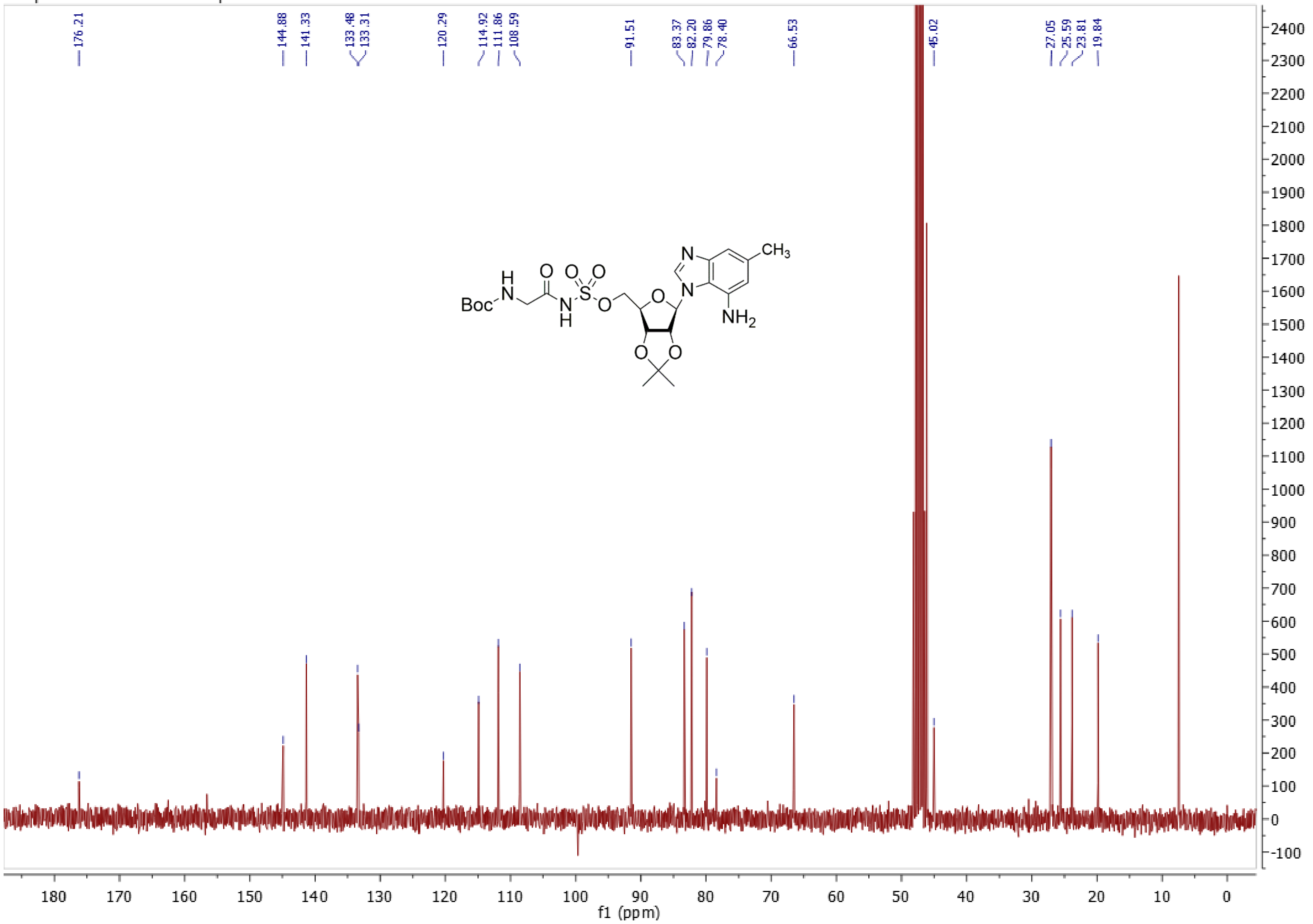

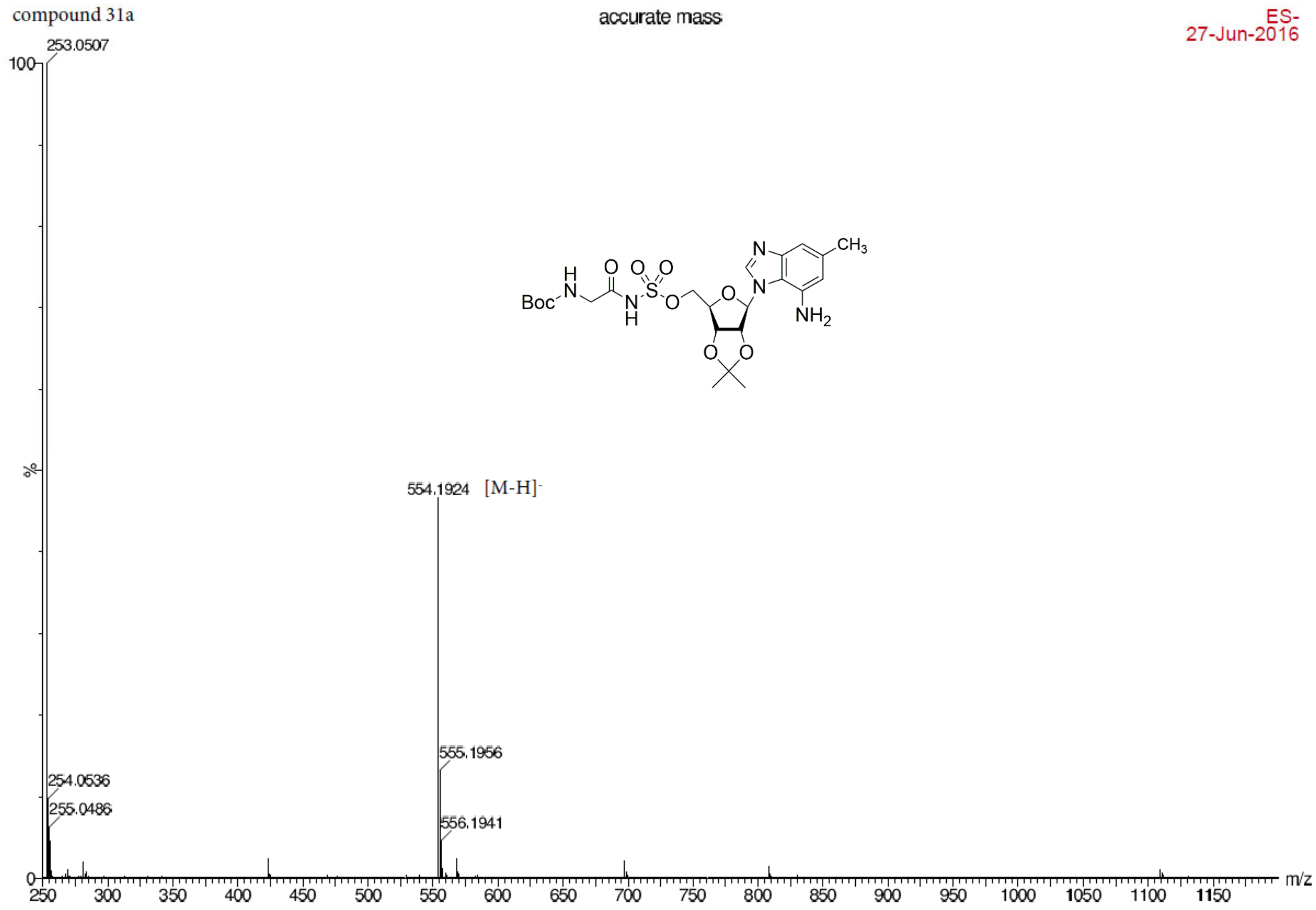

compound 31a
accurate mass
ES-
27-Jun-2016
253.0507
100
%
554.1924 [M-H]-
555.1956
254.0536
255.0486
556.1941
0
250
300
350
400
450
500
550
600
650
700
750
800
850
900
950
1000
1050
1100
1150
m/z
Boc
H
N
O
O
O
S
N
H
O
O
N
N
CH3
NH2
O
O

Compound 31b in DMSO-d6 1H spectrum - 300 MHz

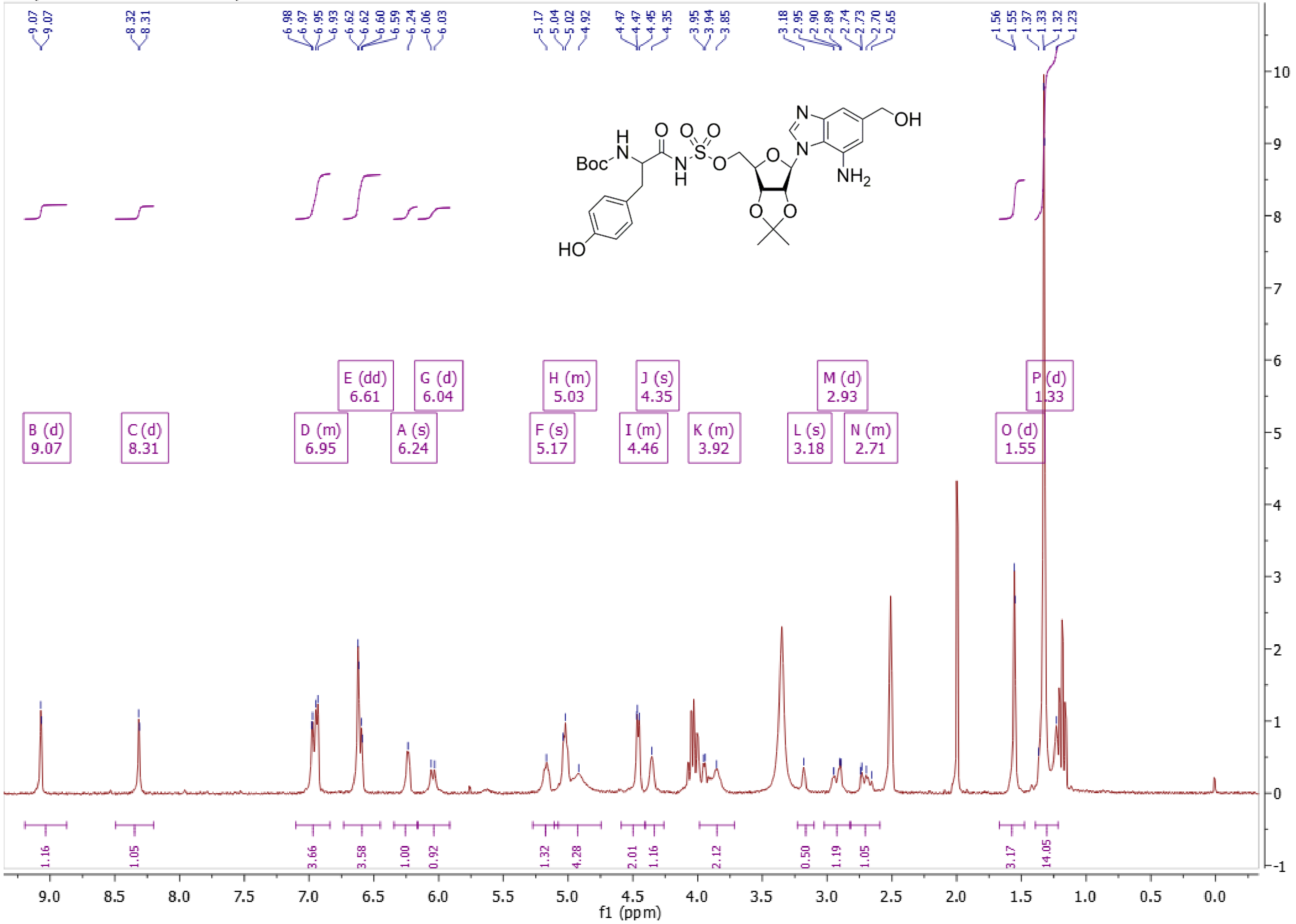

Compound 31b in DMSO-d6 13C spectrum - 75 MHz

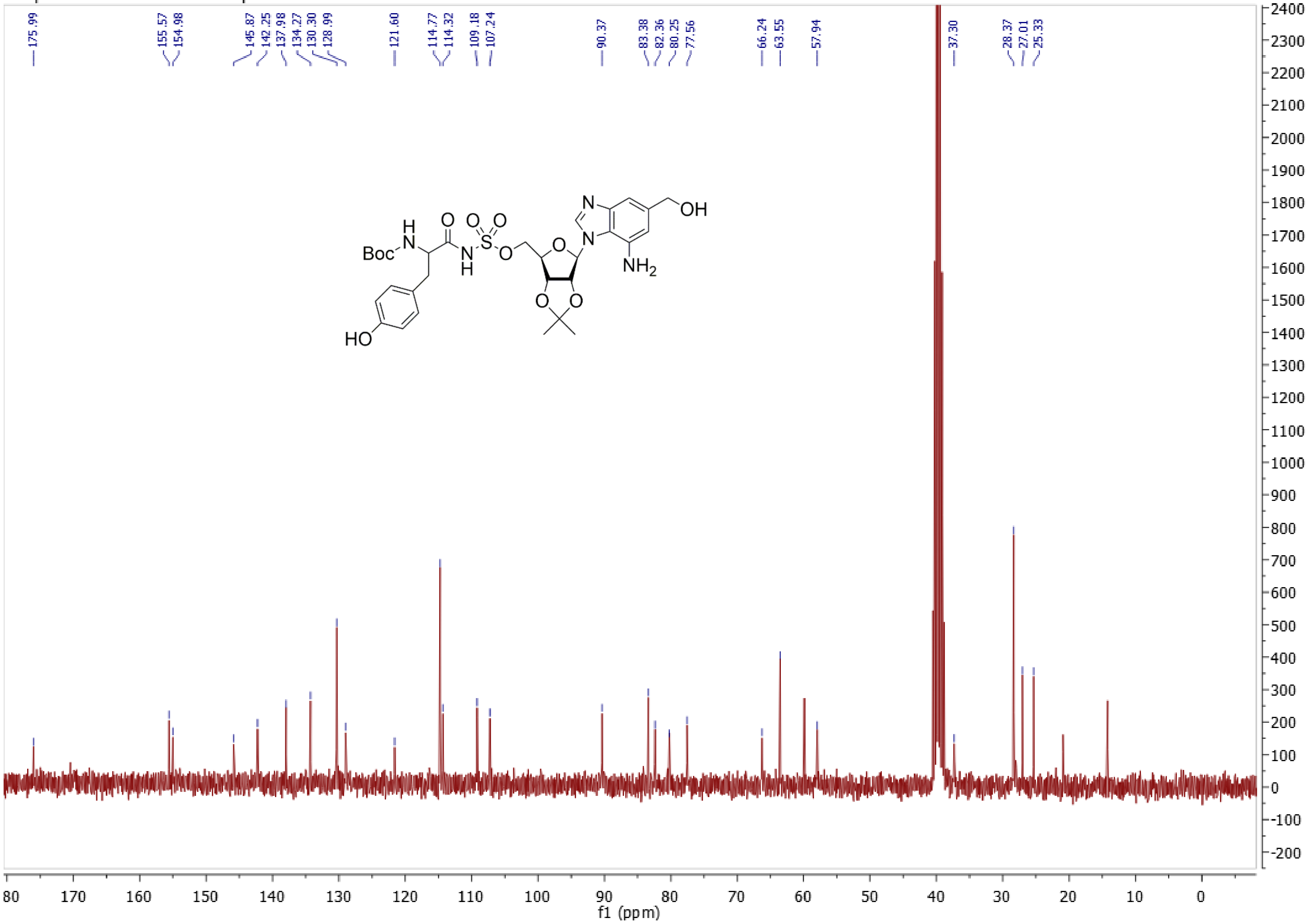

compound 31b

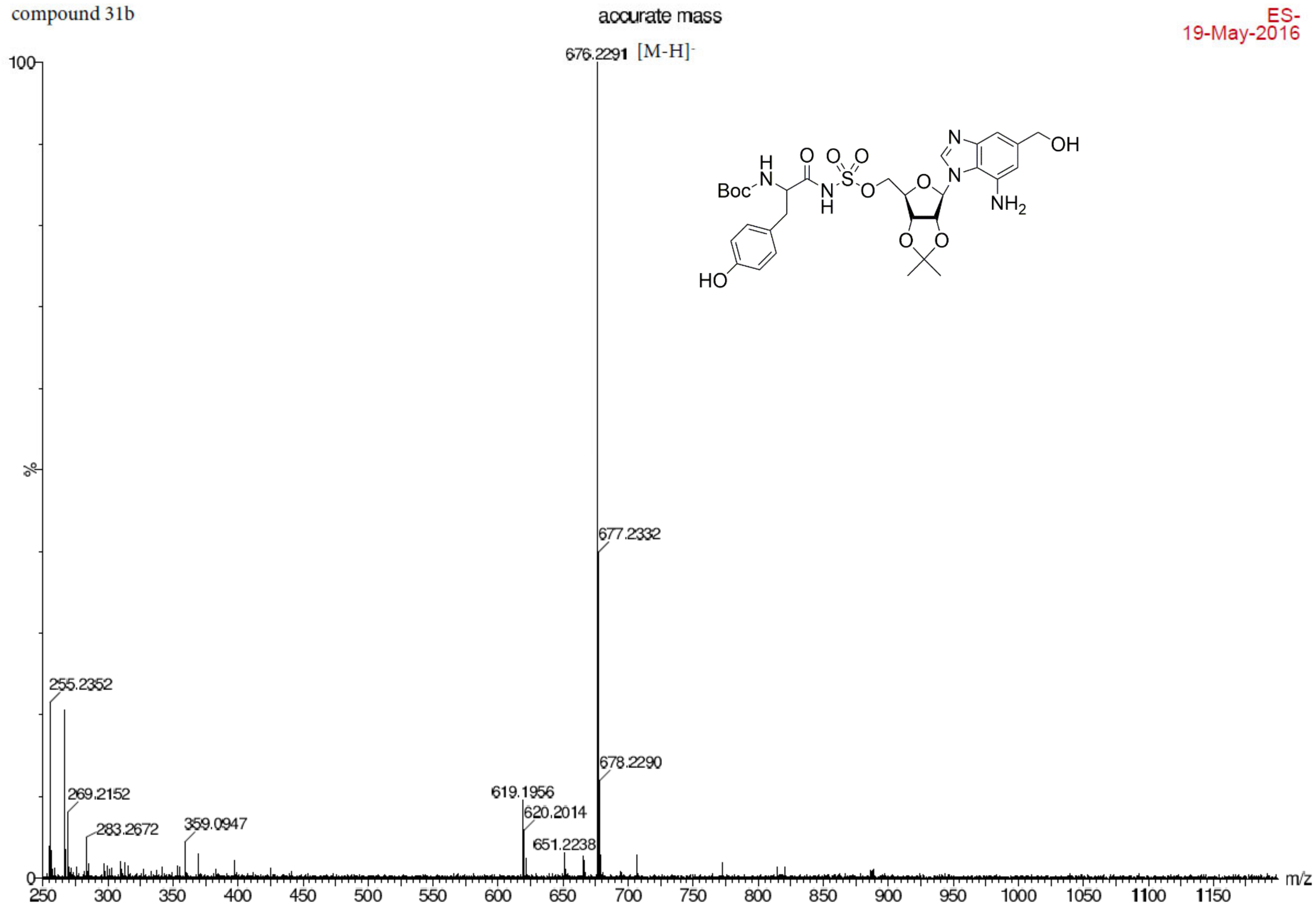

Compound 31c in DMSO-d6 1H spectrum - 300 MHz

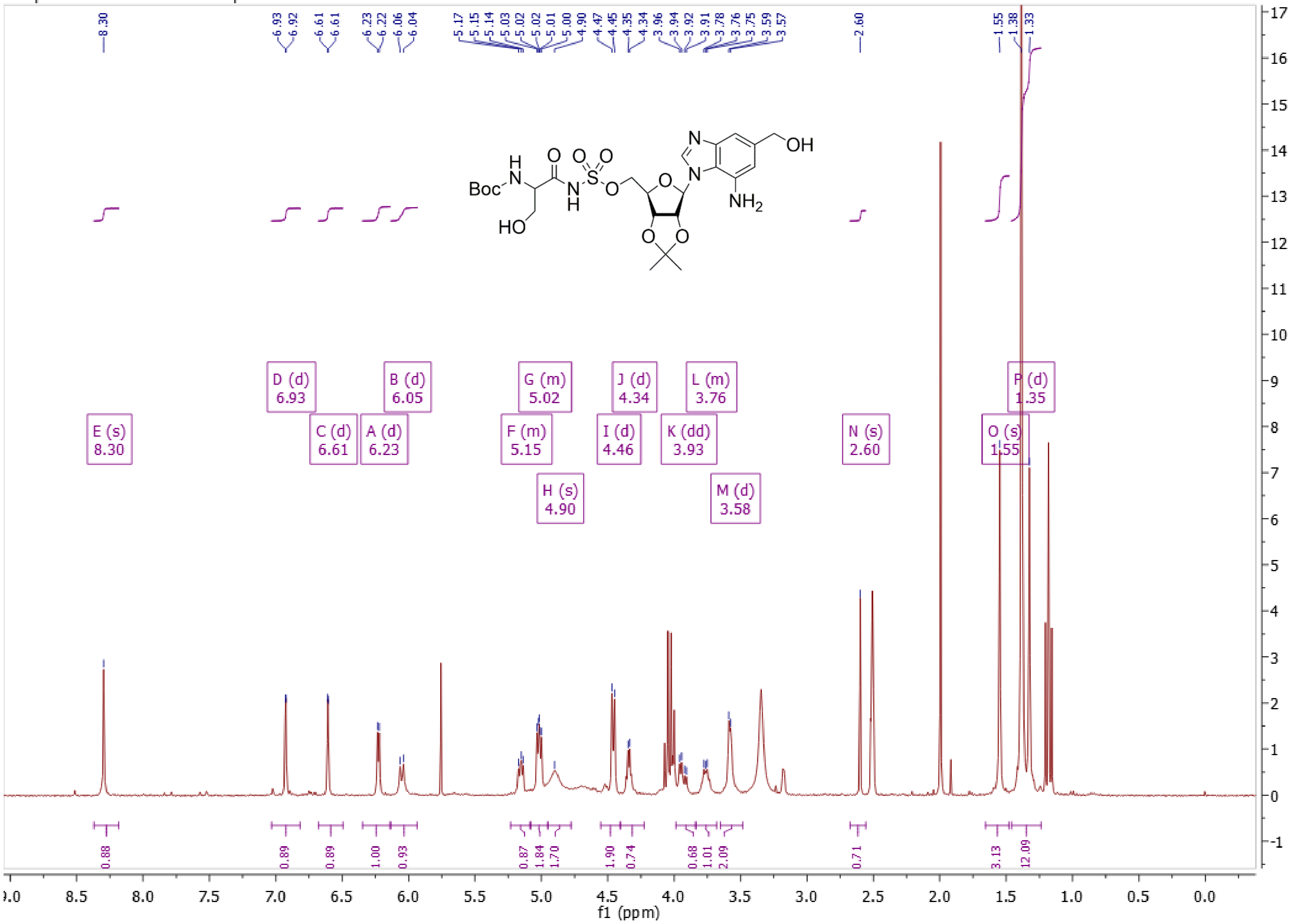

Compound 31c in DMSO-d6 13C spectrum - 75 MHz

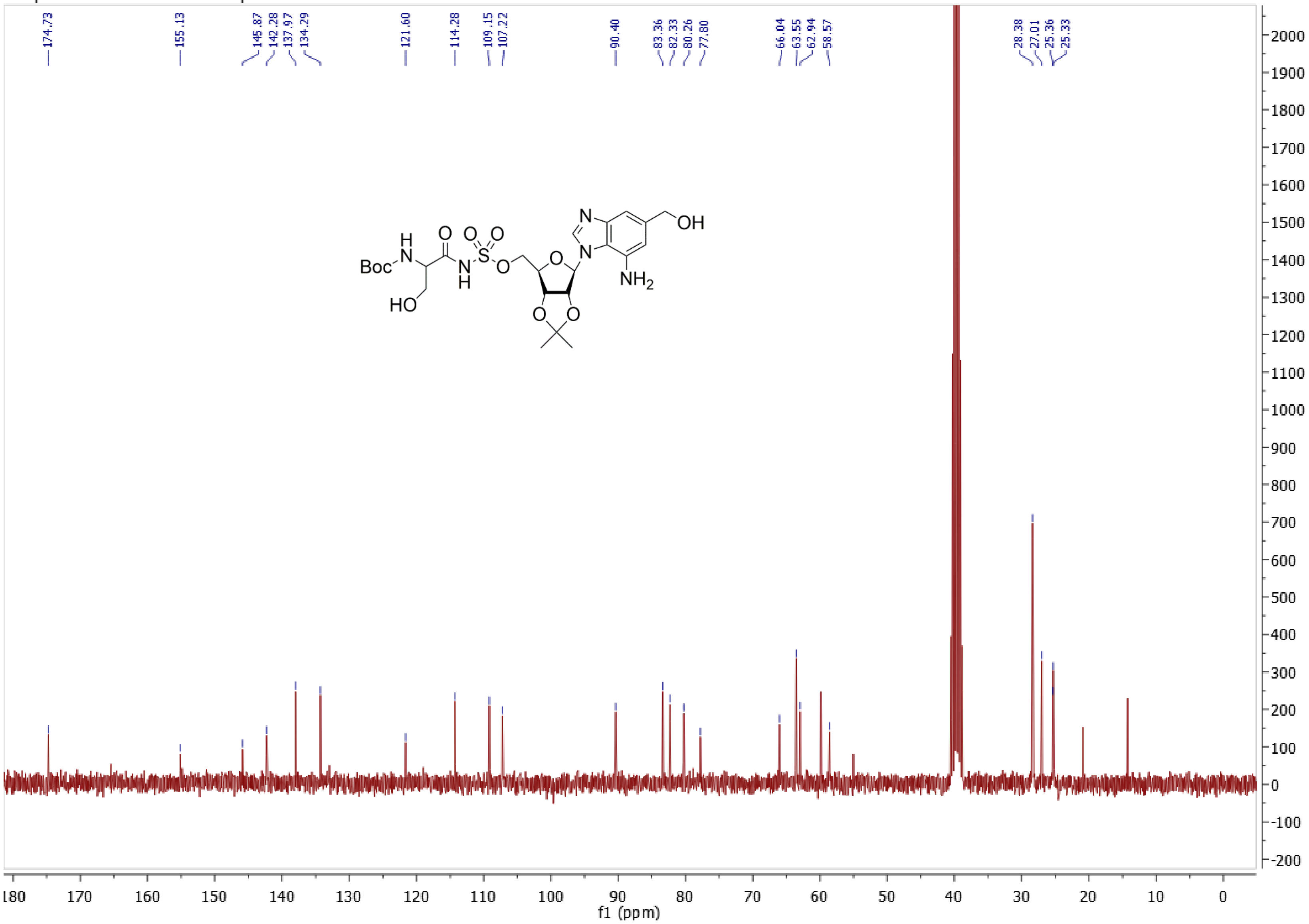

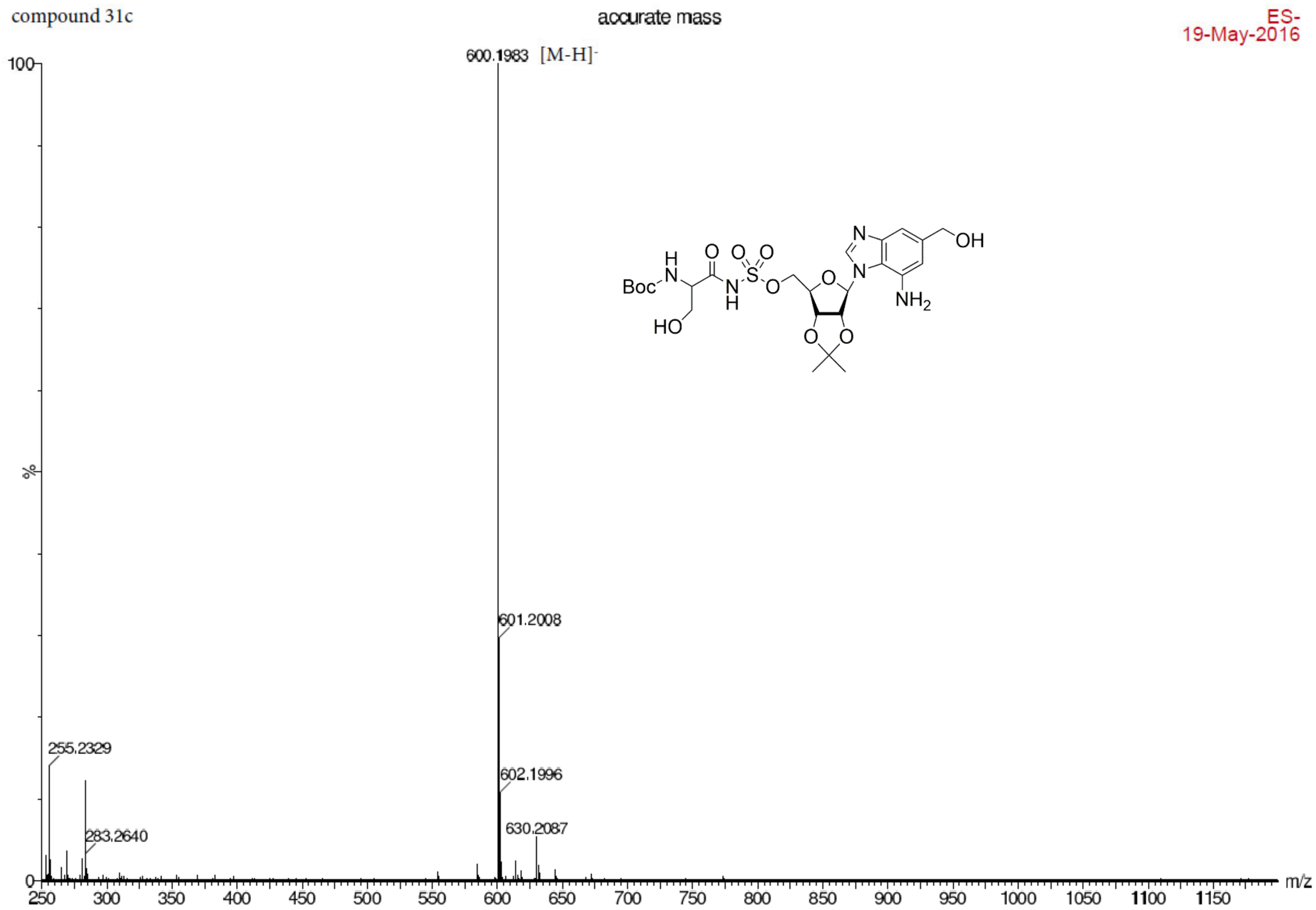
compound 31c
accurate mass
ES-
19-May-2016
600.1983 [M-H]-
601.2008
602.1996
630.2087
255.2329
283.2640
100
%
0
250
300
350
400
450
500
550
600
650
700
750
800
850
900
950
1000
1050
1100
1150
m/z
Boc
H
N
O
O
O
S
N
H
O
O
HO
N
N
OH
NH2
O
O

Compound 31d in DMSO-d6 1H spectrum - 300 MHz

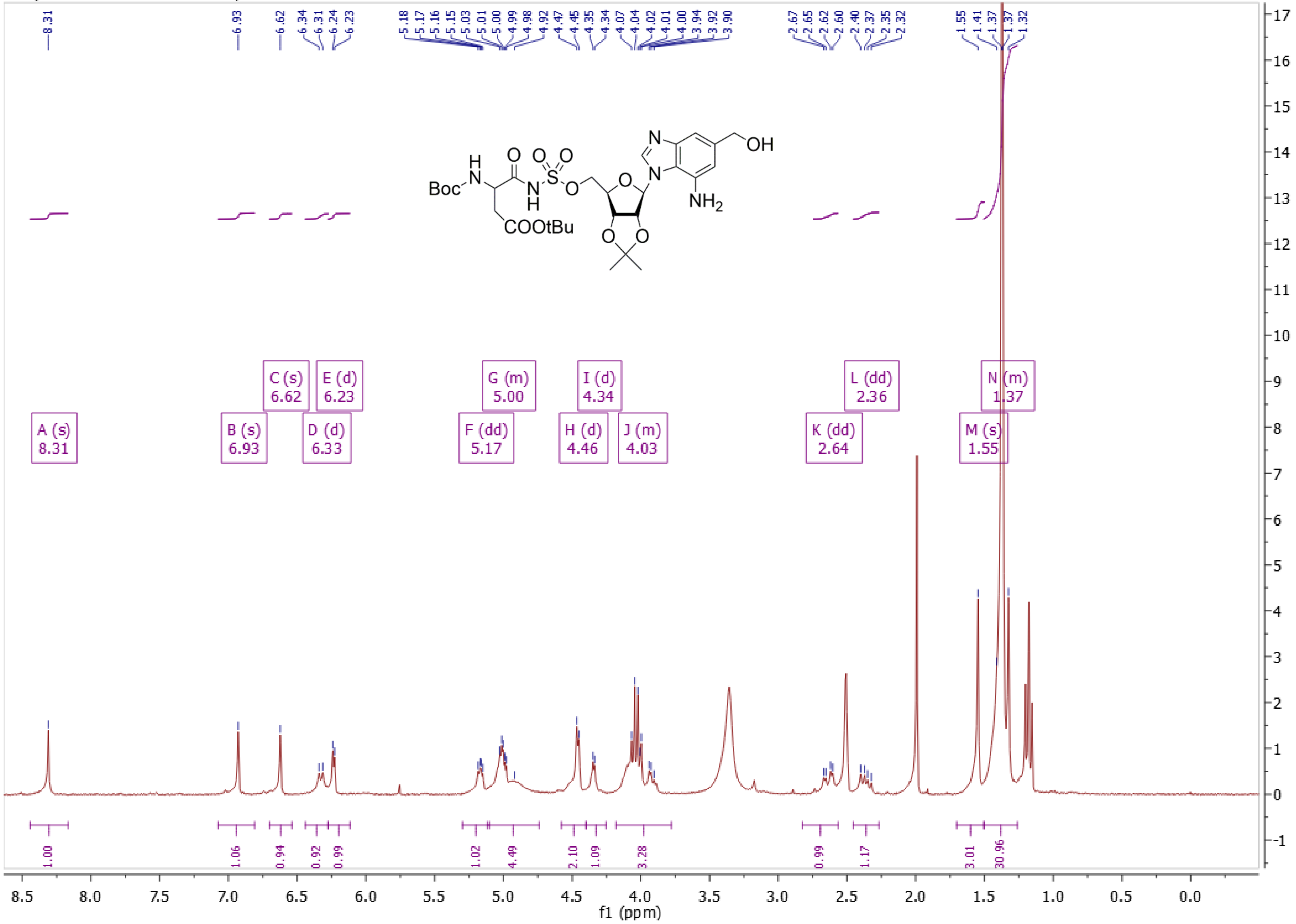

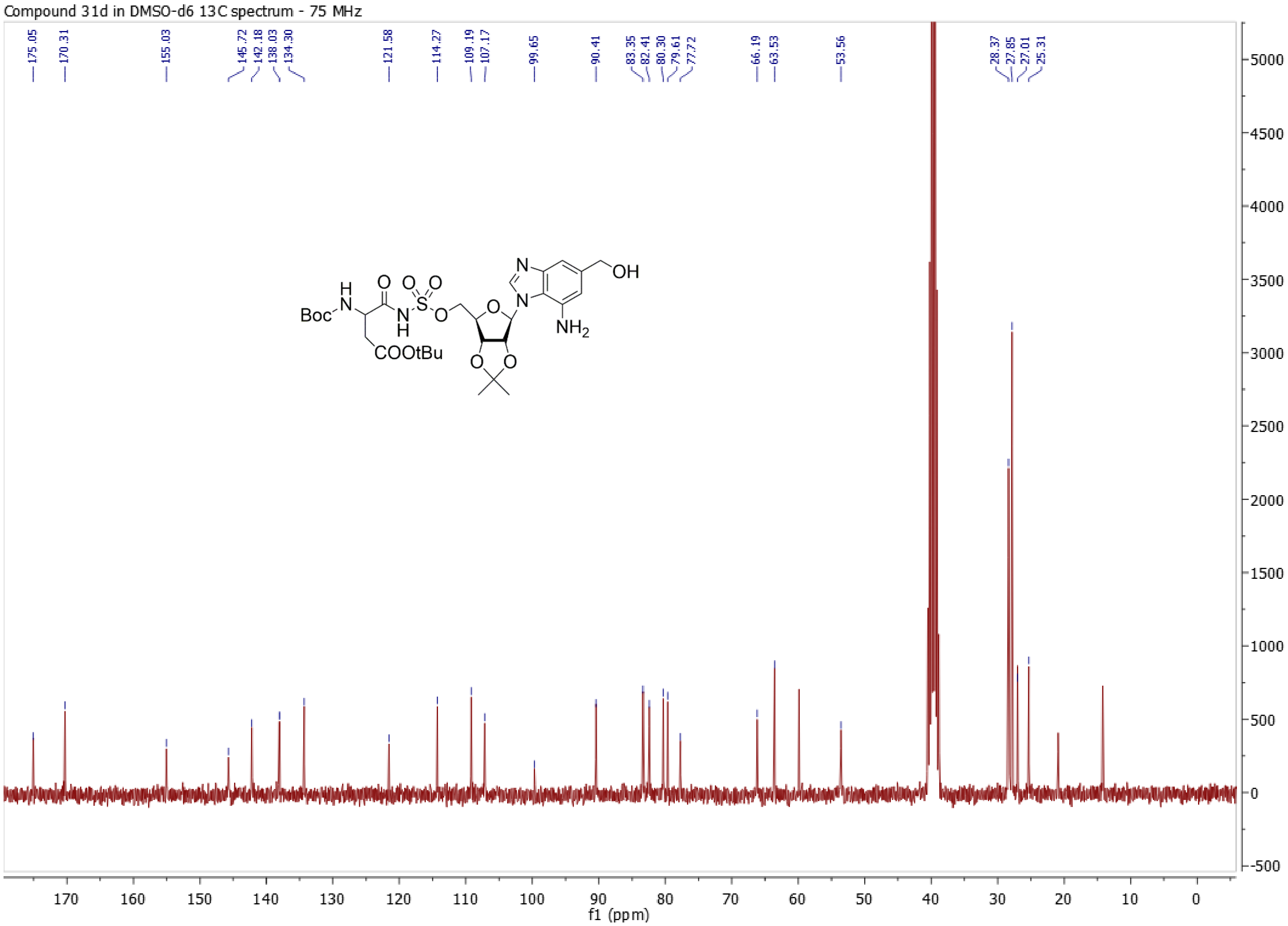
Compound 31d in DMSO-d6 13C spectrum - 75 MHz
175.05
170.31
155.03
145.72
142.18
138.03
134.30
121.58
114.27
109.19
107.17
99.65
90.41
83.35
82.41
80.30
79.61
77.72
66.19
63.53
53.56
28.37
27.85
27.01
25.31
Boc
H
N
O
O
O
S
N
H
O
COOtBu
O
N
N
OH
NH2
O
O
f1 (ppm)

compound 31d

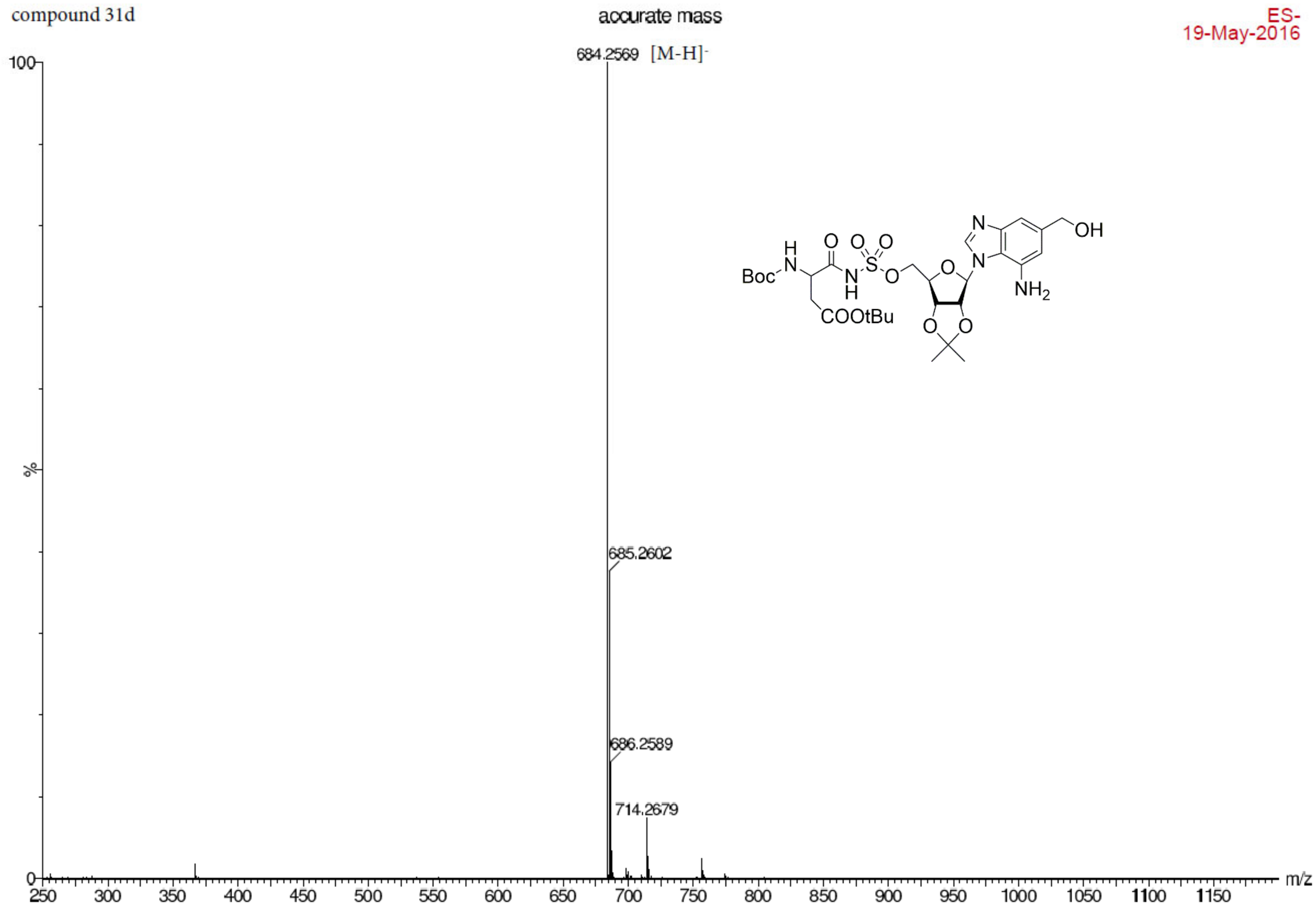

Compound 31e in DMSO-d6 1H spectrum - 300 MHz

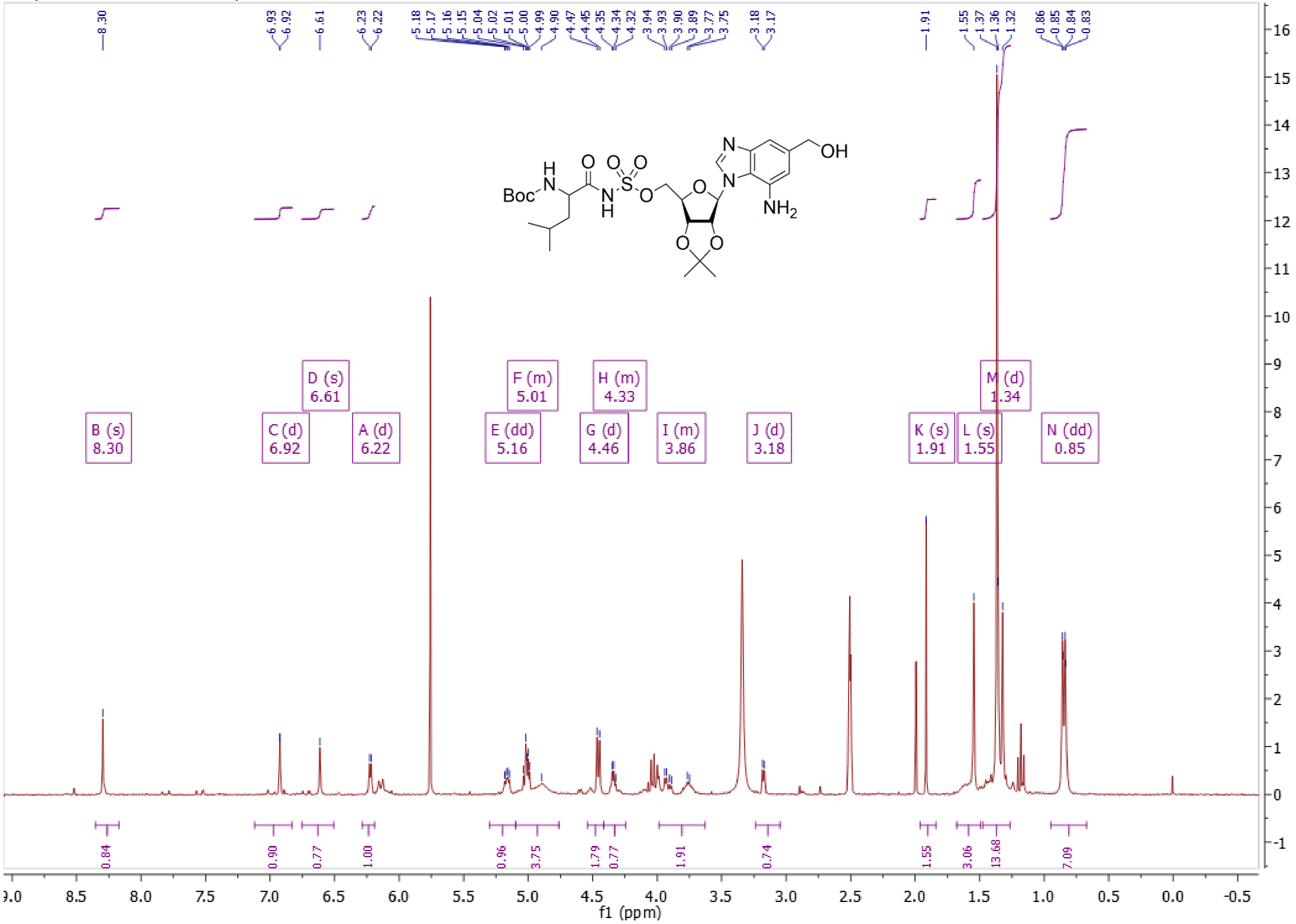

Compound 31e in DMSO-d6 13C spectrum - 75 MHz

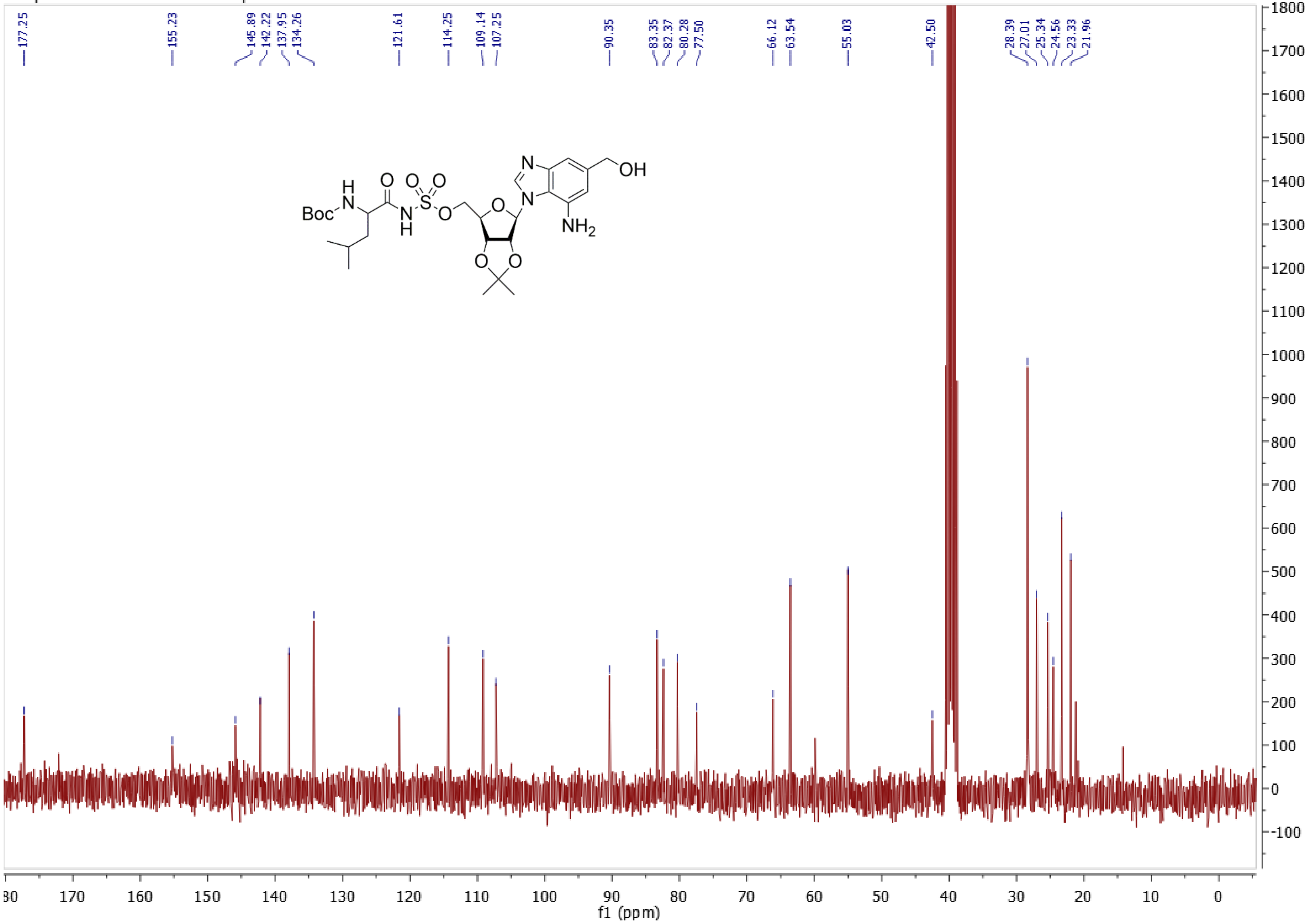

compound 31e

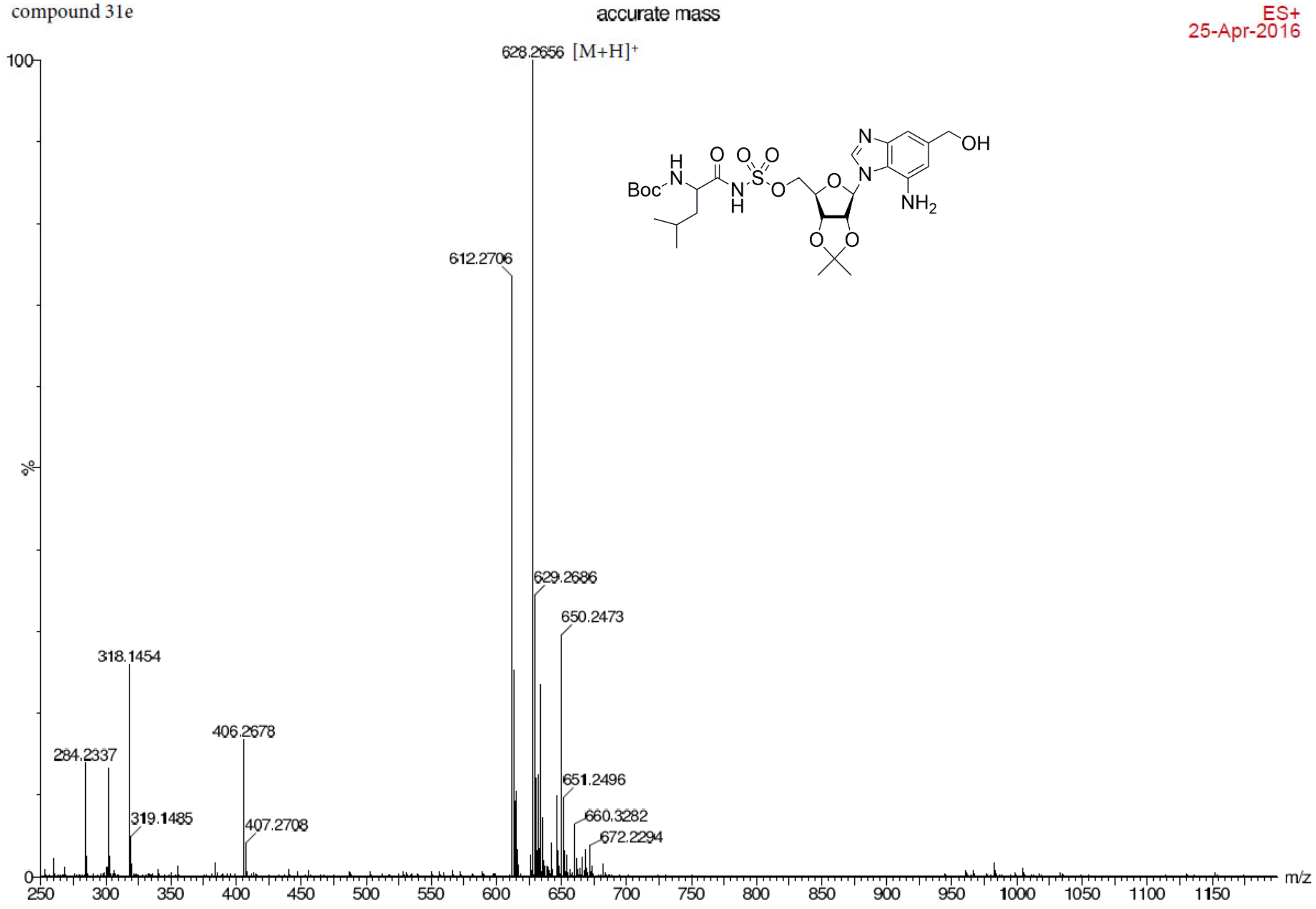

Compound 31f in DMSO-d6 1H spectrum - 300 MHz

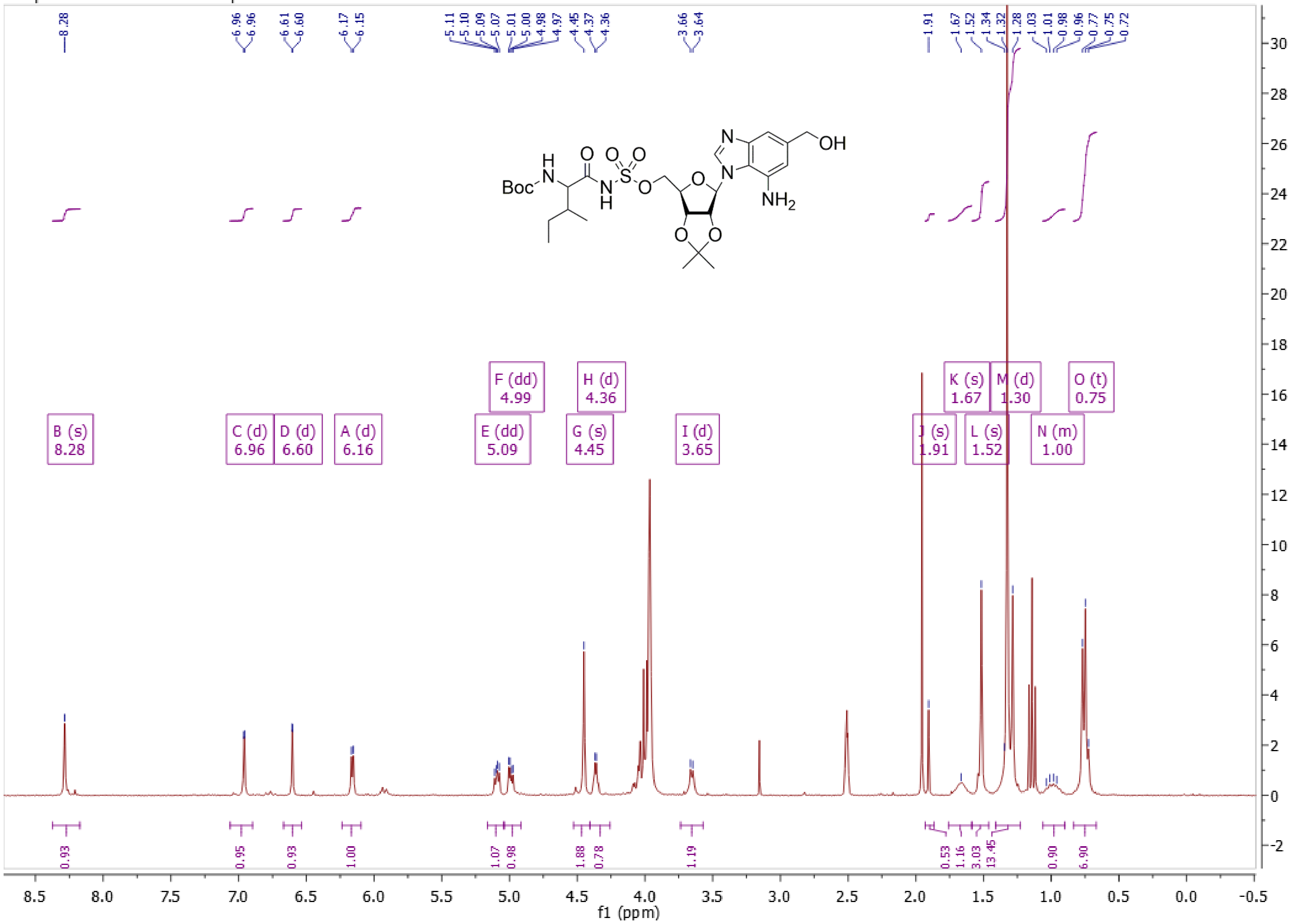

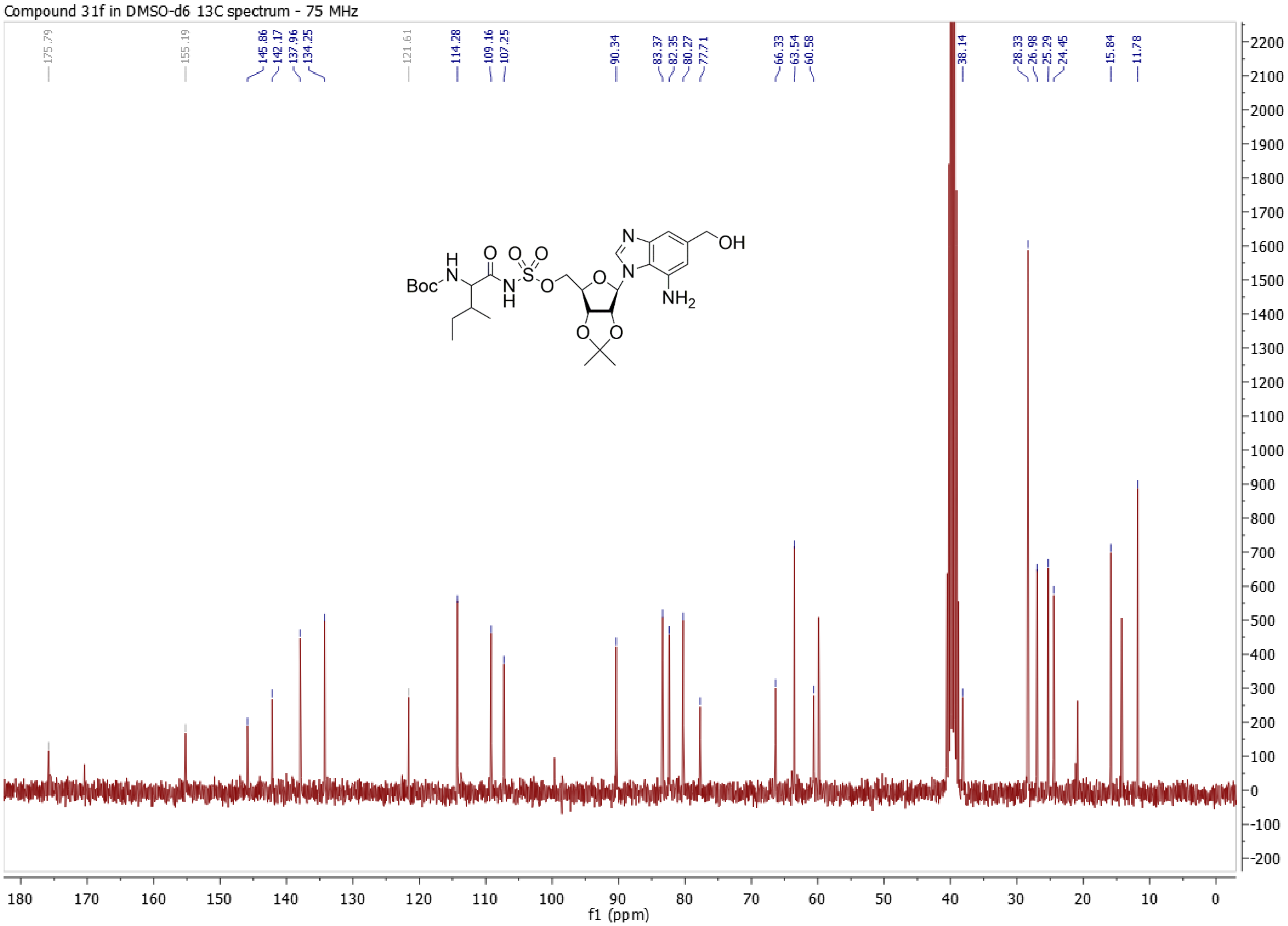
Compound 31f in DMSO-d6 13C spectrum - 75 MHz
175.79
155.19
145.86
142.17
137.96
134.25
121.61
114.28
109.16
107.25
90.34
83.37
82.35
80.27
77.71
66.33
63.54
60.58
38.14
28.33
26.98
25.29
24.45
15.84
11.78
Boc
H
N
O
O
O
S
N
H
O
O
N
N
OH
NH2
O
O
f1 (ppm)

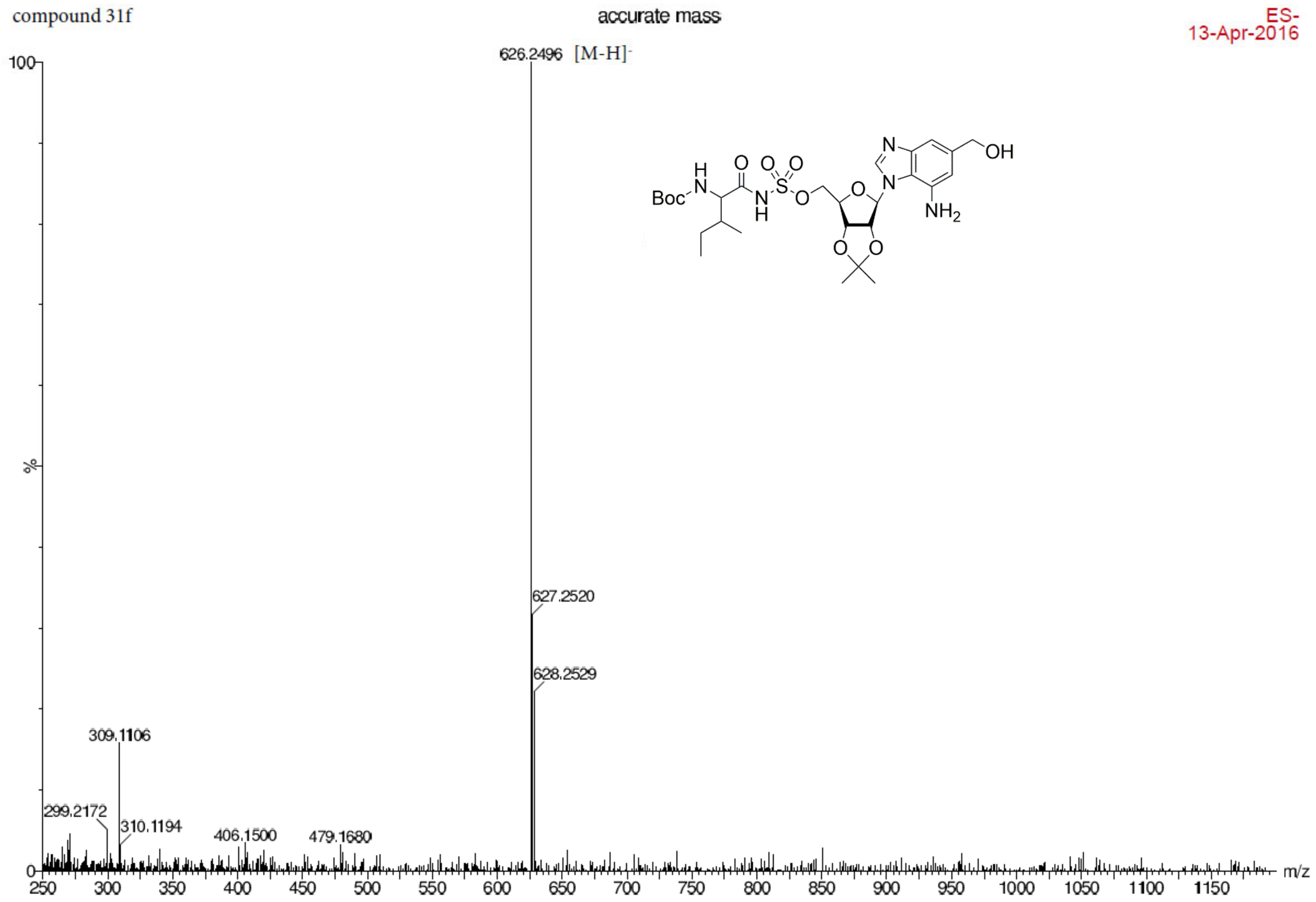
compound 31f
accurate mass
ES-
13-Apr-2016
626.2496 [M-H]-
627.2520
628.2529
309.1106
299.2172
310.1194
406.1500
479.1680
%
m/z
Boc
NH2
OH

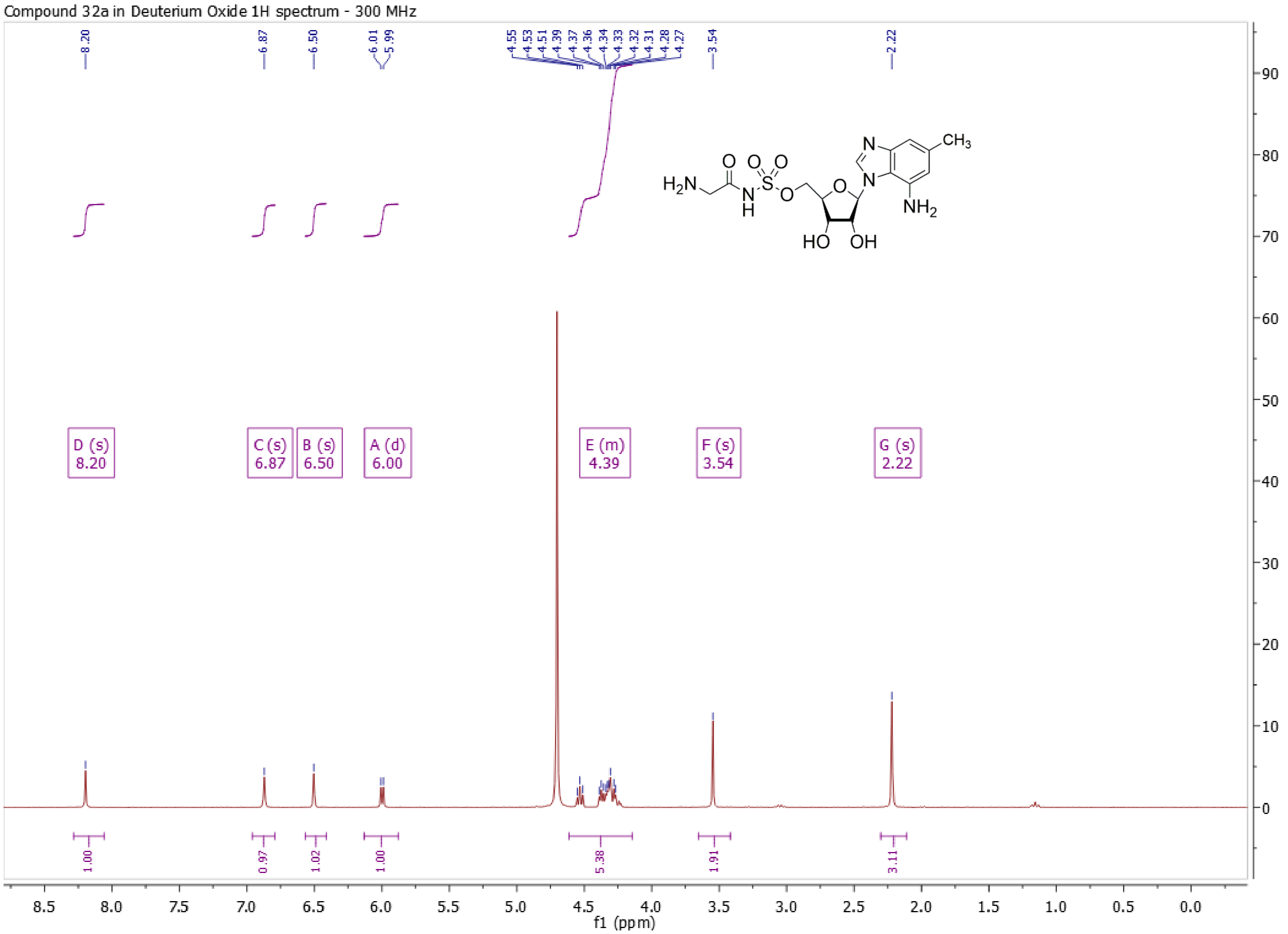

Compound 32a in Deuterium Oxide 1H spectrum - 300 MHz
8.20
6.87
6.50
6.01
5.99
4.55
4.53
4.51
4.39
4.37
4.36
4.34
4.33
4.32
4.31
4.28
4.27
3.54
2.22
D (s) 8.20
C (s) 6.87
B (s) 6.50
A (d) 6.00
E (m) 4.39
F (s) 3.54
G (s) 2.22
1.00
0.97
1.02
1.00
5.38
1.91
3.11
H2N
O
O
O
S
N
H
O
O
HO
OH
N
N
CH3
NH2
8.5 8.0 7.5 7.0 6.5 6.0 5.5 5.0 4.5 4.0 3.5 3.0 2.5 2.0 1.5 1.0 0.5 0.0
f1 (ppm)
90 80 70 60 50 40 30 20 10 0

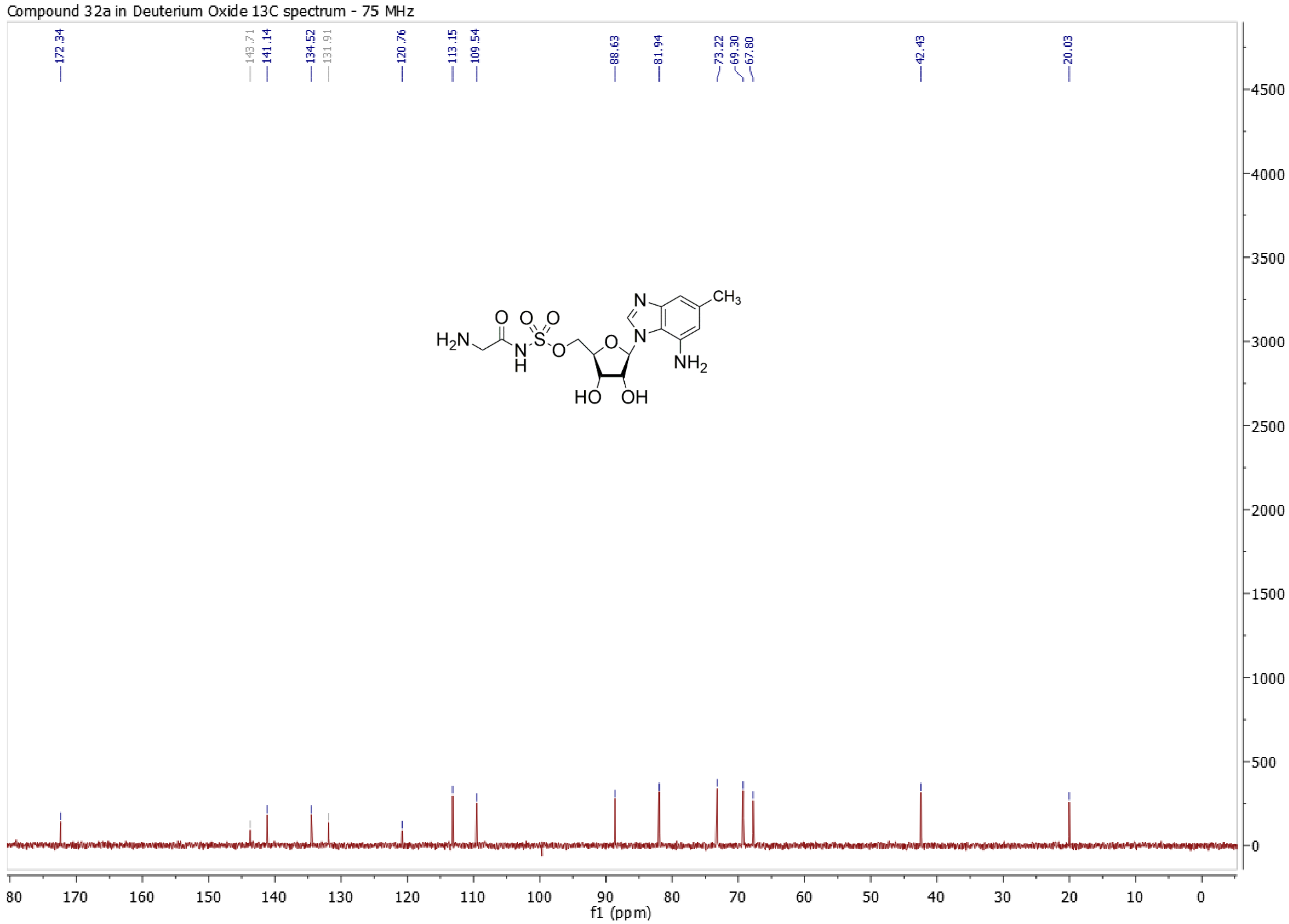

Compound 32a in Deuterium Oxide 13C spectrum - 75 MHz
172.34
143.71
141.14
134.52
131.91
120.76
113.15
109.54
88.63
81.94
73.22
69.30
67.80
42.43
20.03
H2N
O
O
O
N
H
S
O
O
N
N
CH3
NH2
HO
OH
4500
4000
3500
3000
2500
2000
1500
1000
500
0
80
170
160
150
140
130
120
110
100
90
80
70
60
50
40
30
20
10
0
f1 (ppm)

compound 32a

accurate mass

ES+
27-Jun-2016

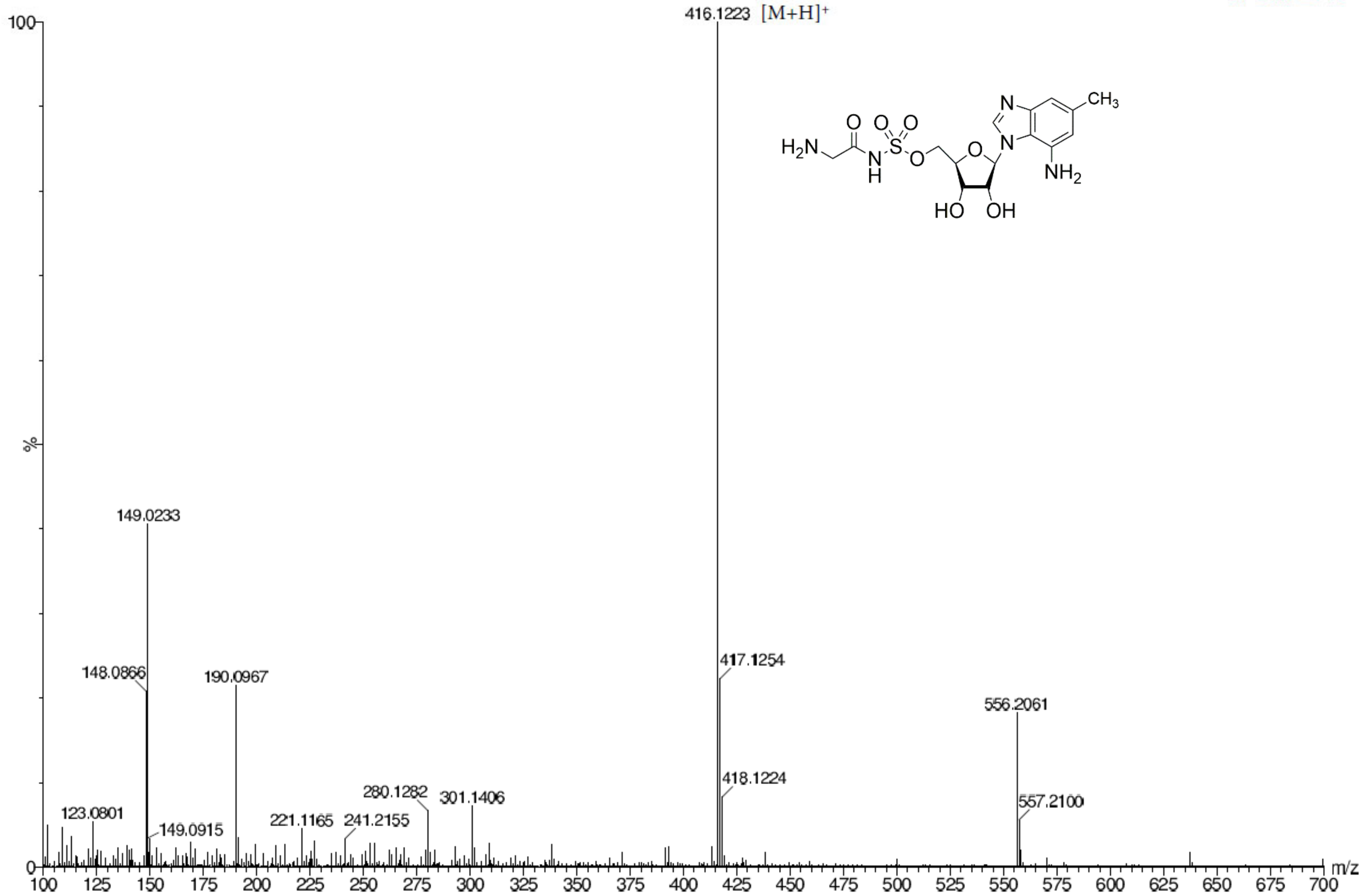

Compound 32b in Deuterium Oxide 13C spectrum - 75 MHz

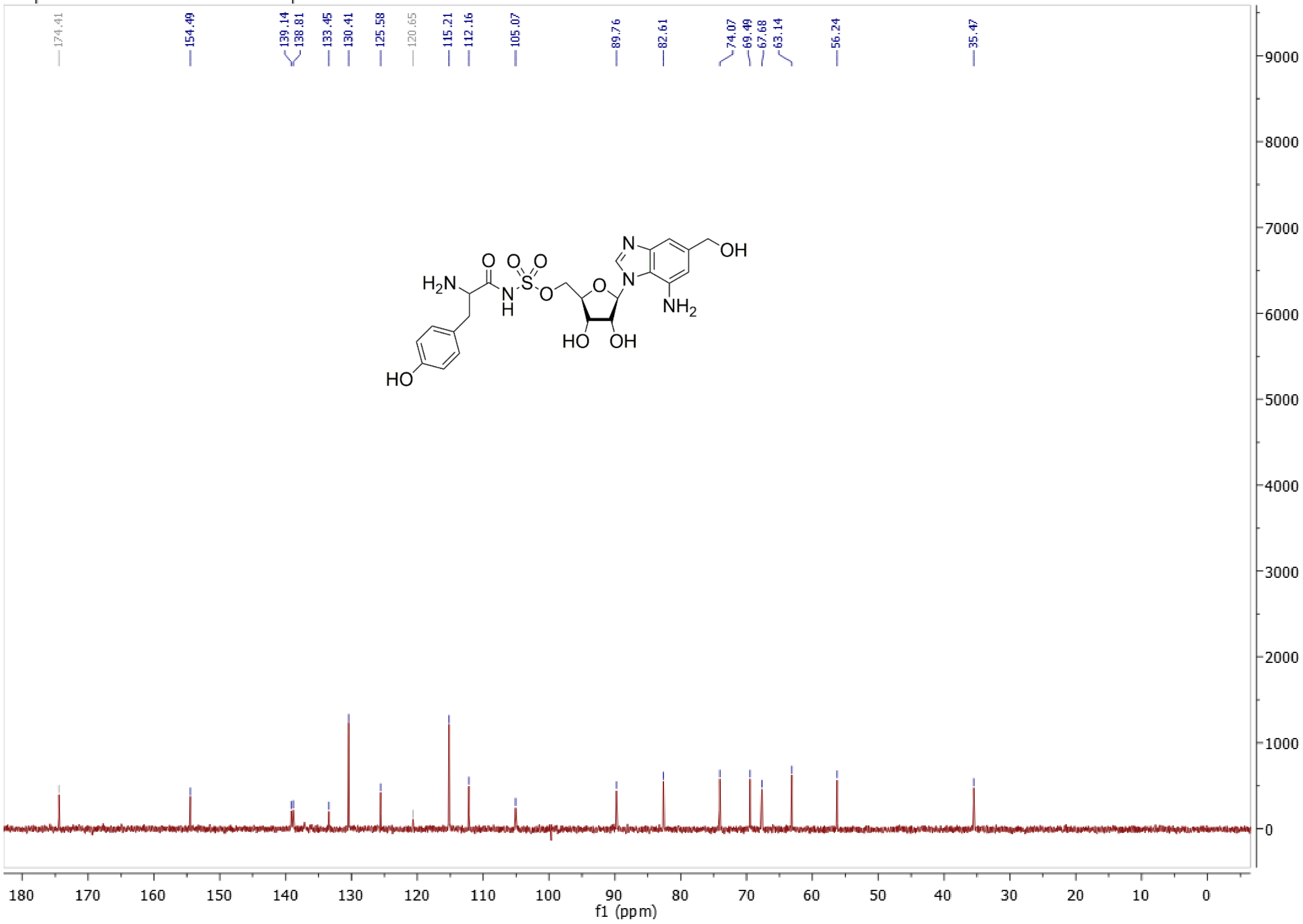

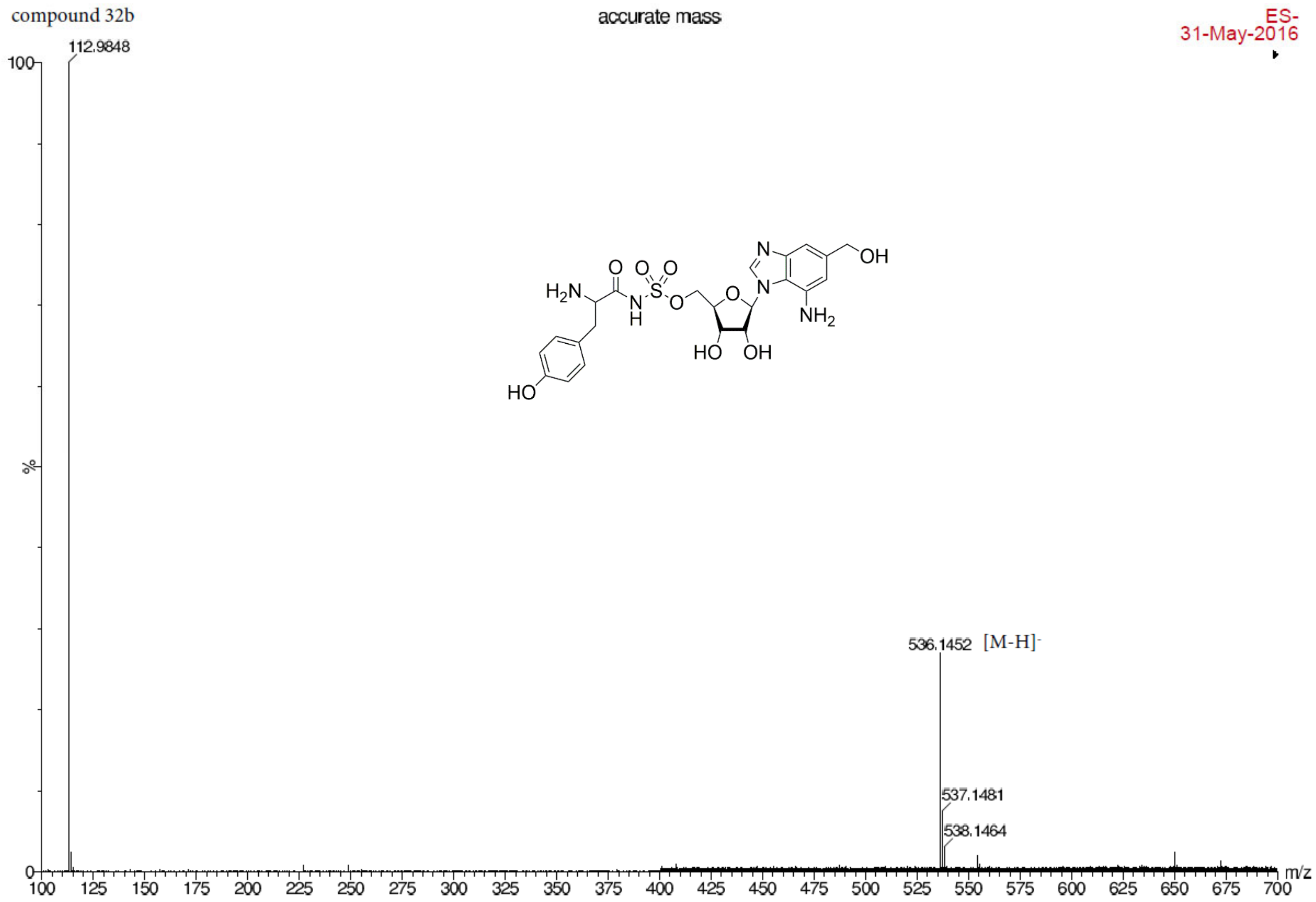
compound 32b
accurate mass
ES-
31-May-2016
112.9848
536.1452 [M-H]-
537.1481
538.1464
%
m/z
100
0
100 125 150 175 200 225 250 275 300 325 350 375 400 425 450 475 500 525 550 575 600 625 650 675 700

Compound 32c in Deuterium Oxide 1H spectrum - 300 MHz

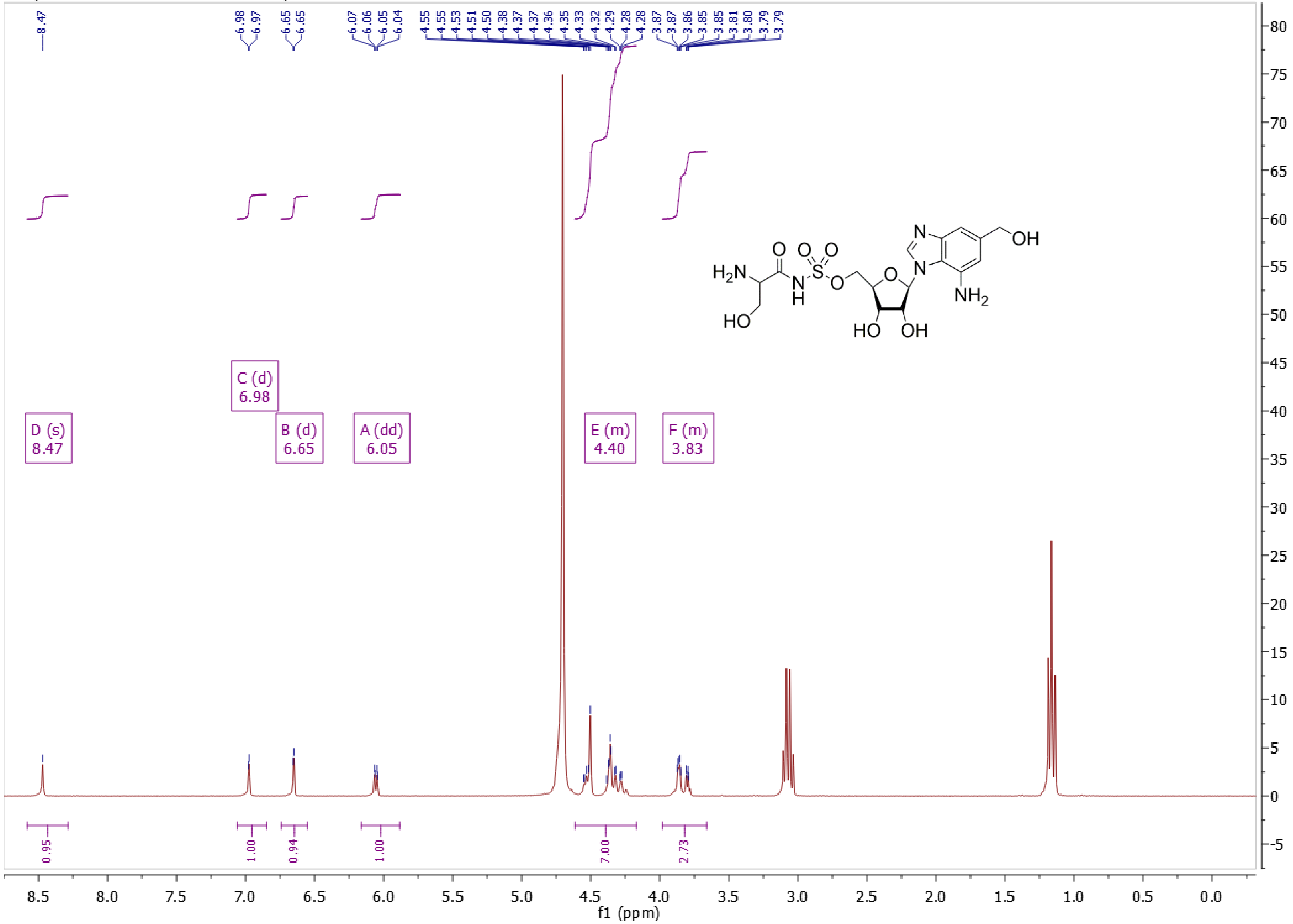

Compound 32c in Deuterium Oxide 13C spectrum - 75 MHz

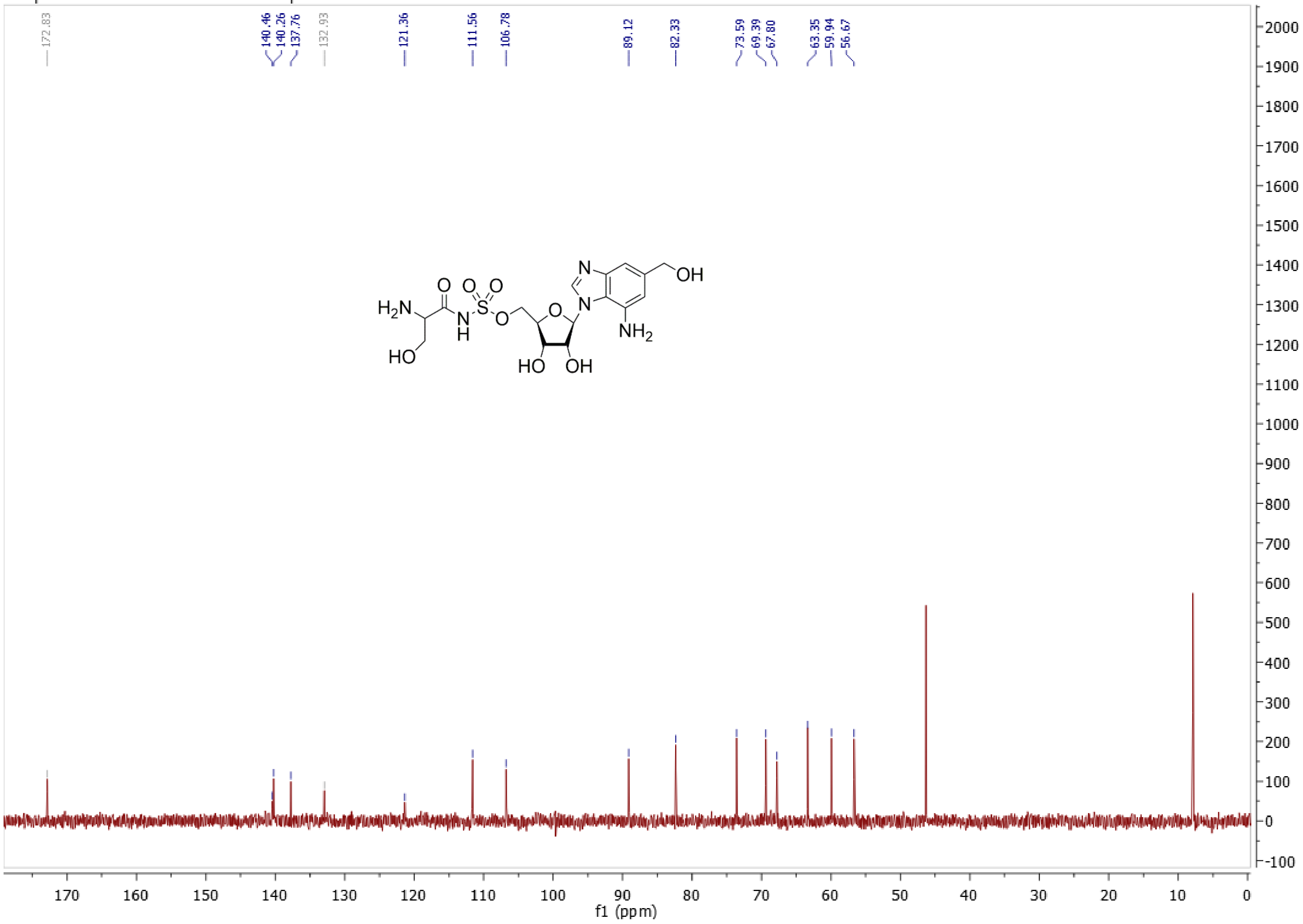

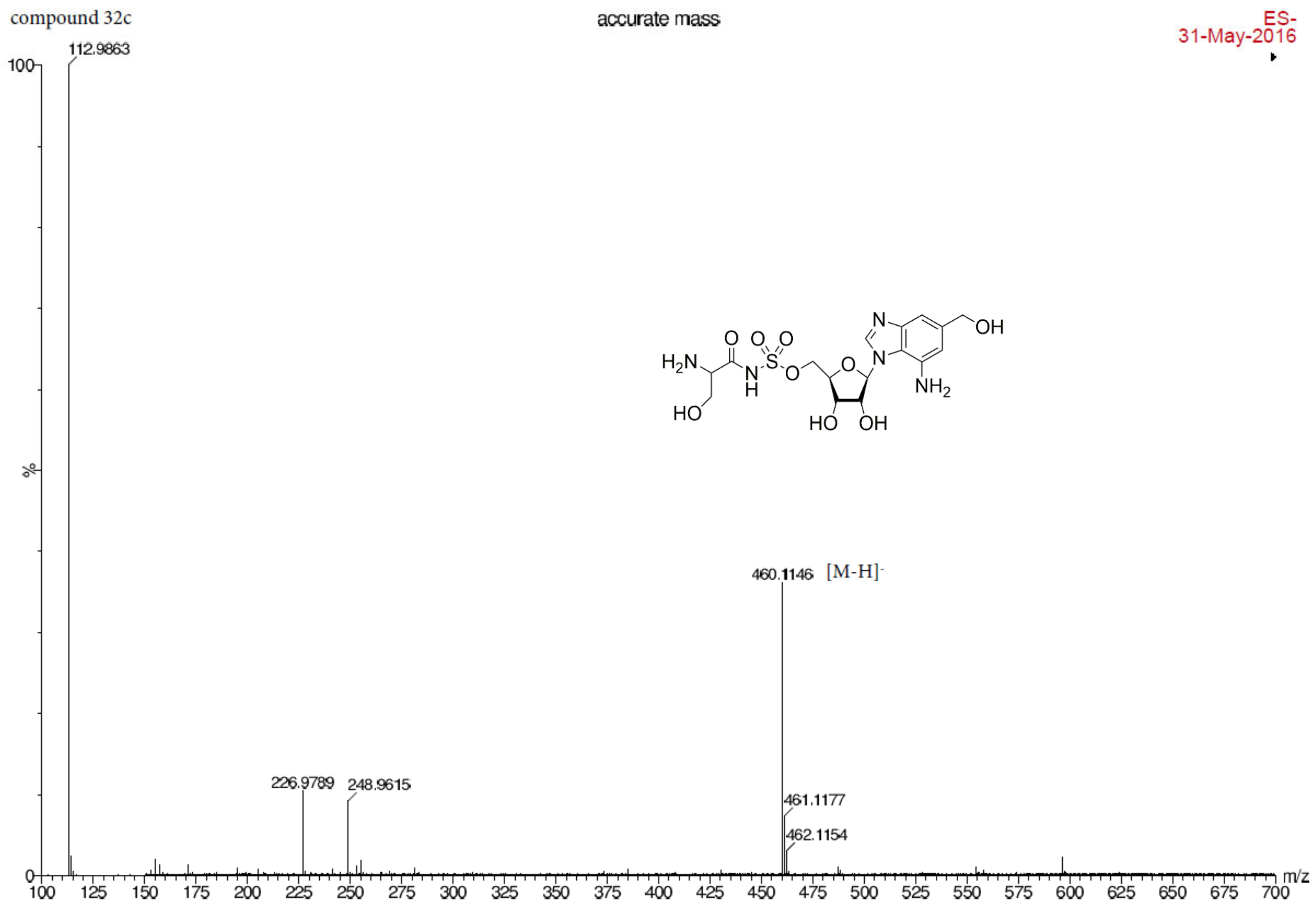
compound 32c
accurate mass
ES-
31-May-2016
112.9863
100
%
0
460.1146 [M-H]-
226.9789
248.9615
461.1177
462.1154
100 125 150 175 200 225 250 275 300 325 350 375 400 425 450 475 500 525 550 575 600 625 650 675 700
m/z
H2N
O
O O
S
N
H
O
O
N
N
OH
NH2
HO
HO OH

Compound 32d in Deuterium Oxide 1H spectrum - 300 MHz

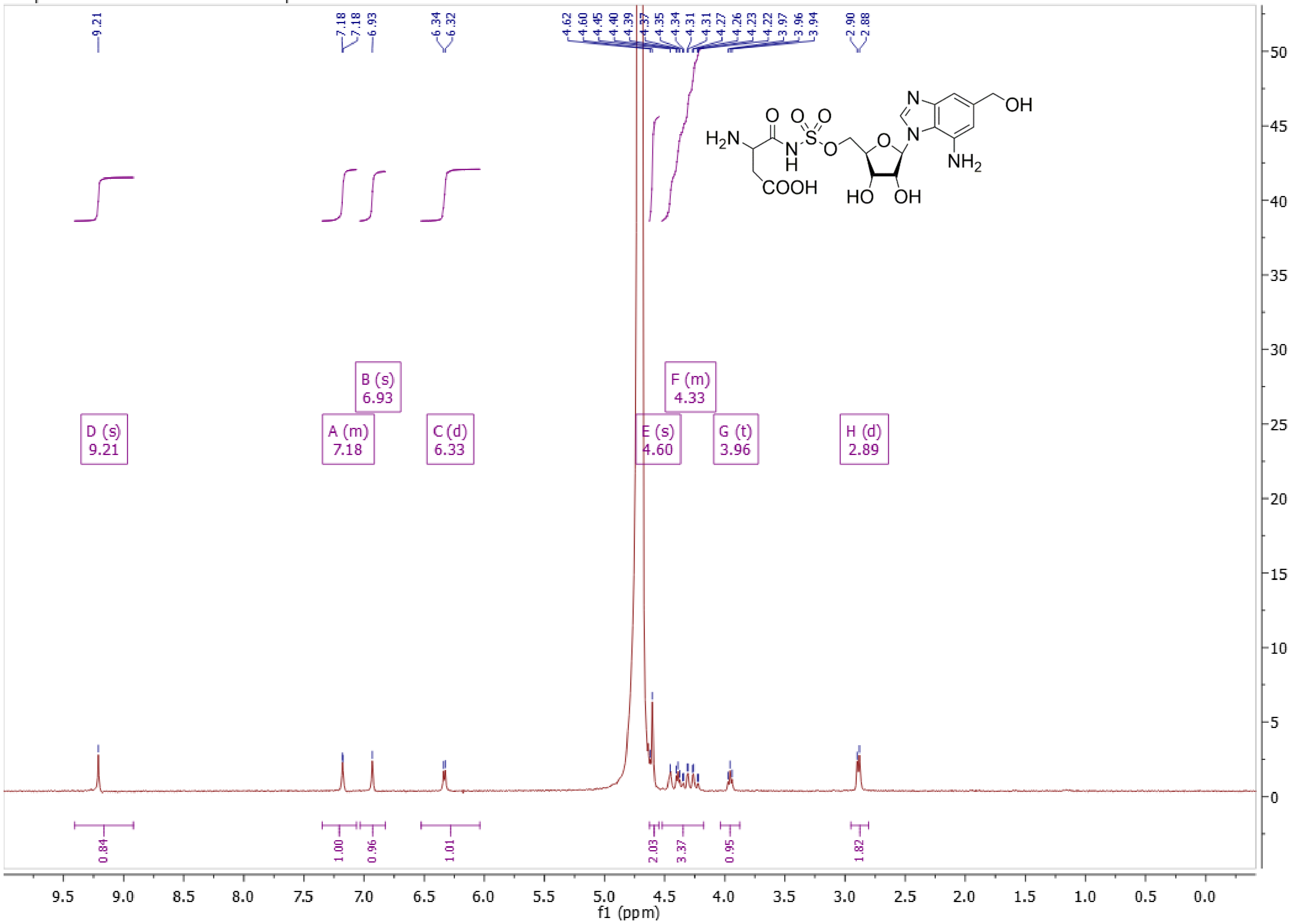

Compound 32d in Deuterium Oxide 13C spectrum - 75 MHz

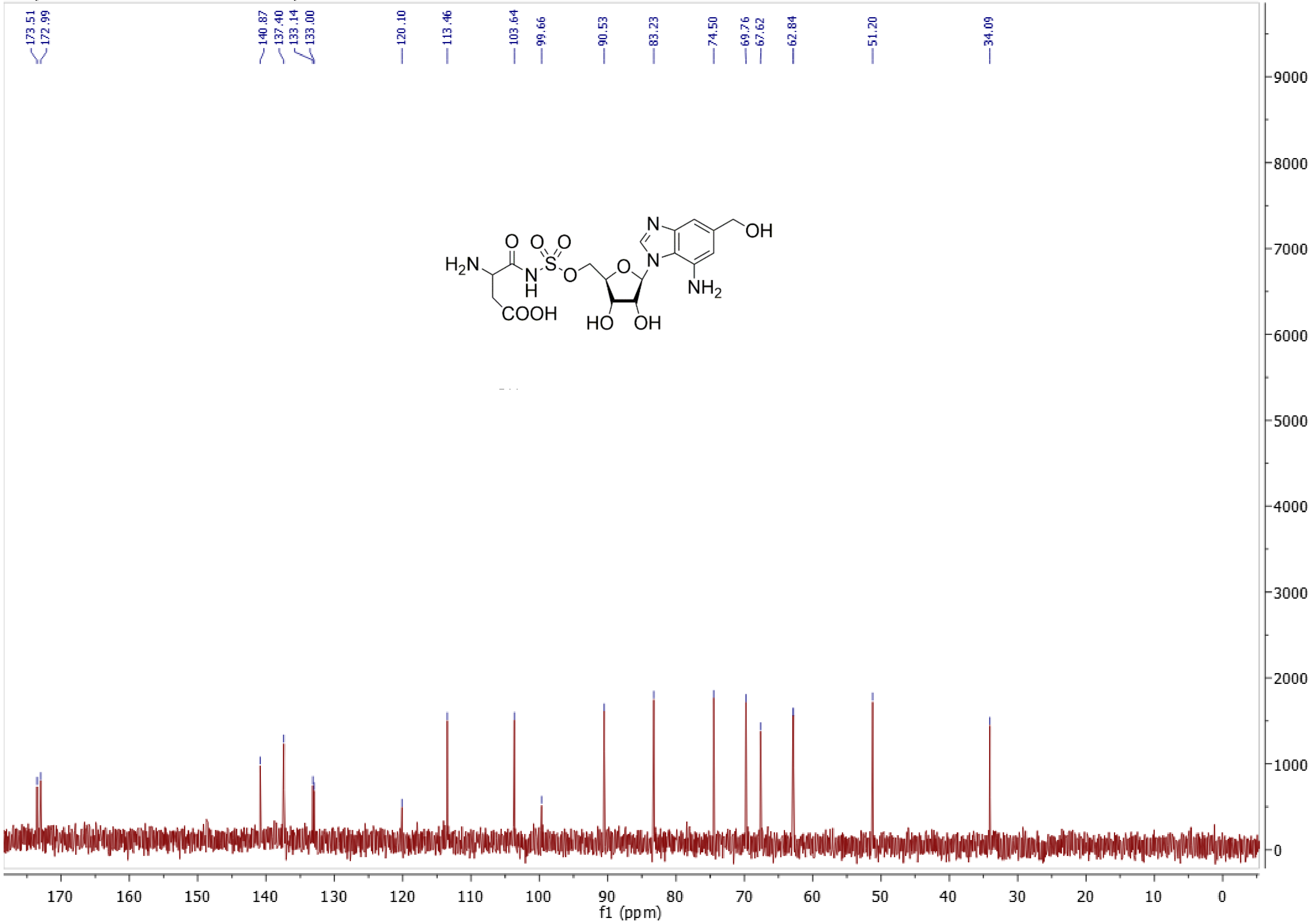

Compound 32d

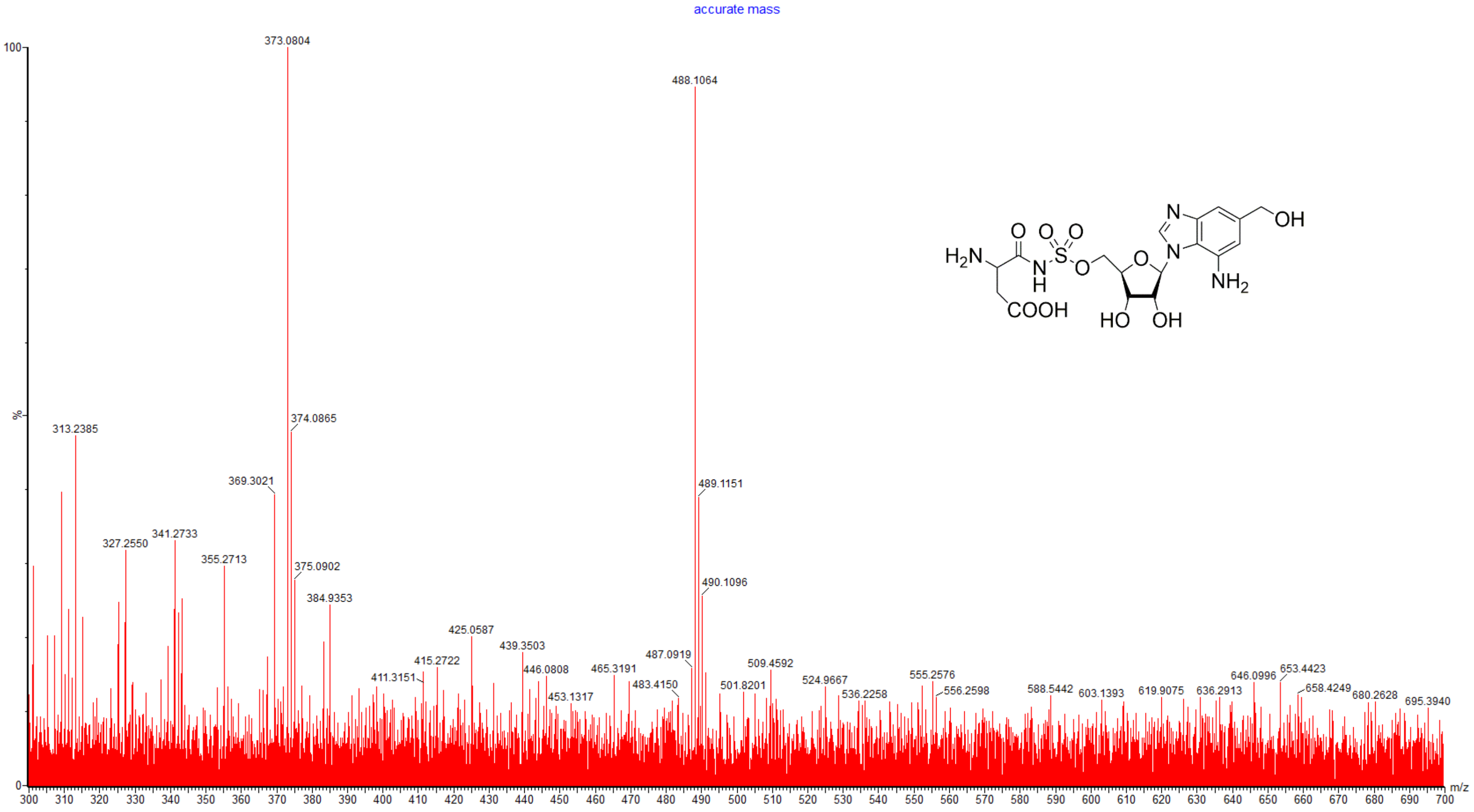

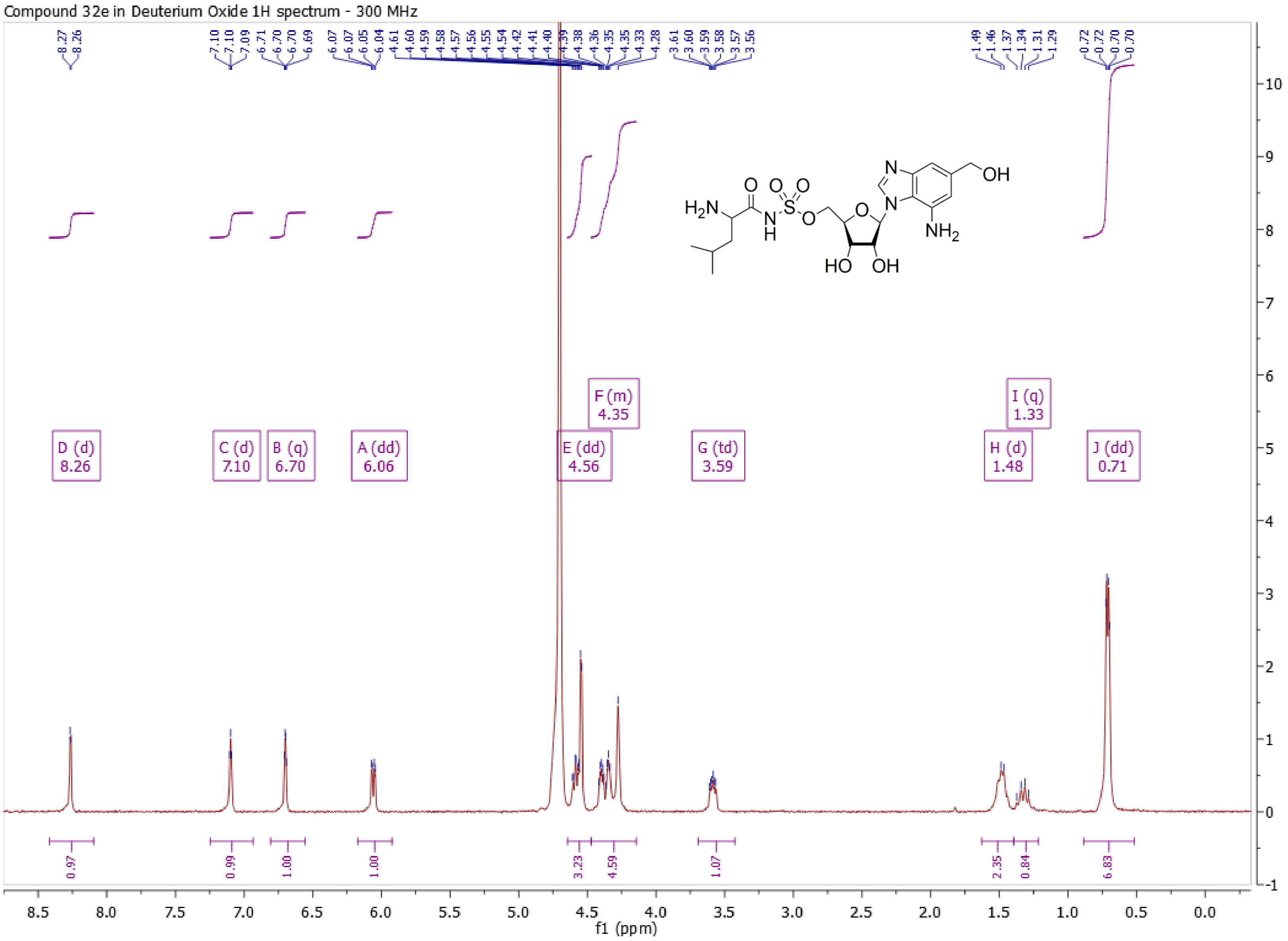
Compound 32e in Deuterium Oxide 1H spectrum - 300 MHz
D (d) 8.26
C (d) 7.10
B (q) 6.70
A (dd) 6.06
E (dd) 4.56
F (m) 4.35
G (td) 3.59
H (d) 1.48
I (q) 1.33
J (dd) 0.71
f1 (ppm)
H2N
O
O
O
N
H
S
O
O
N
N
OH
NH2
HO
OH

Compound 32e in Deuterium Oxide 13C spectrum - 75 MHz

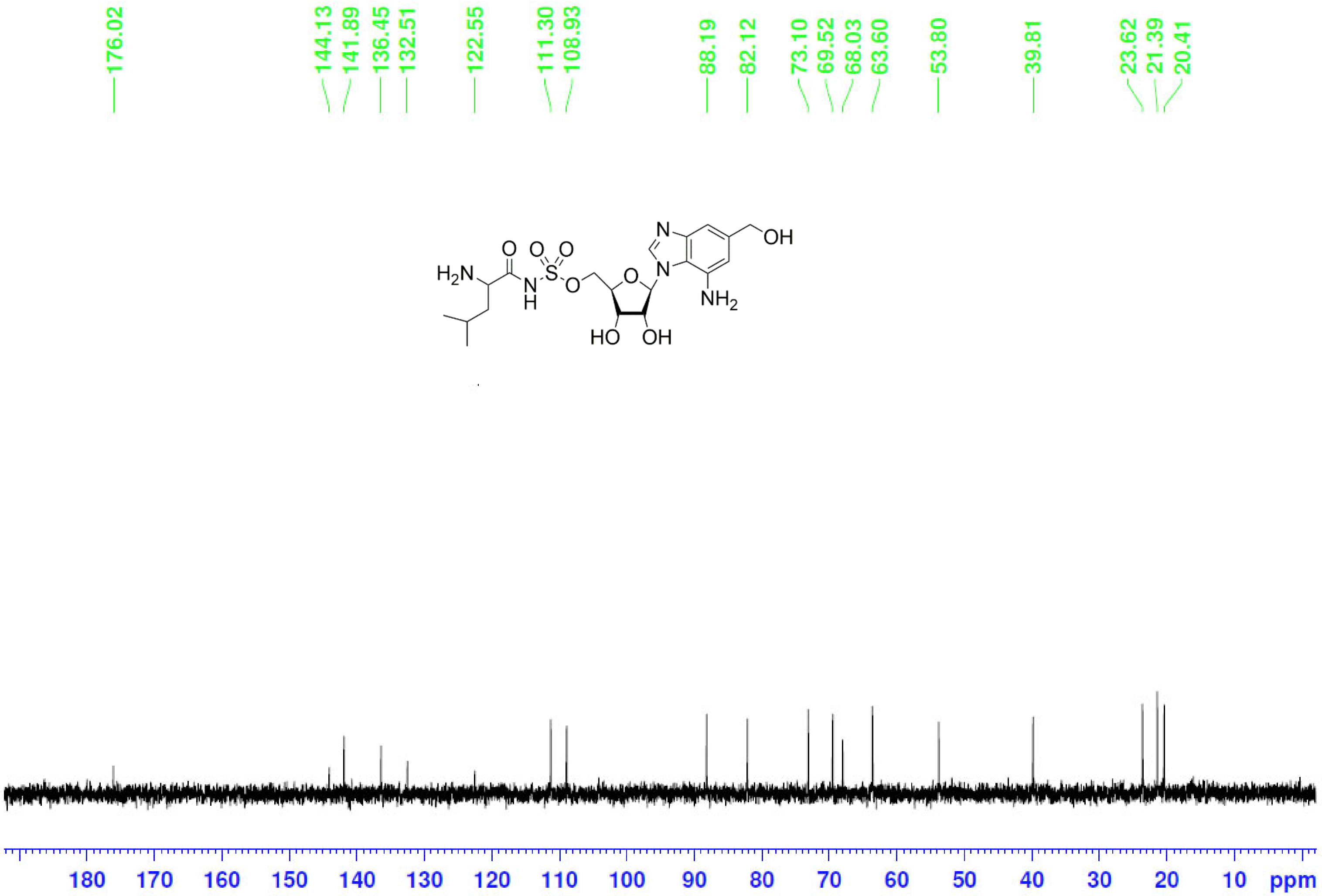

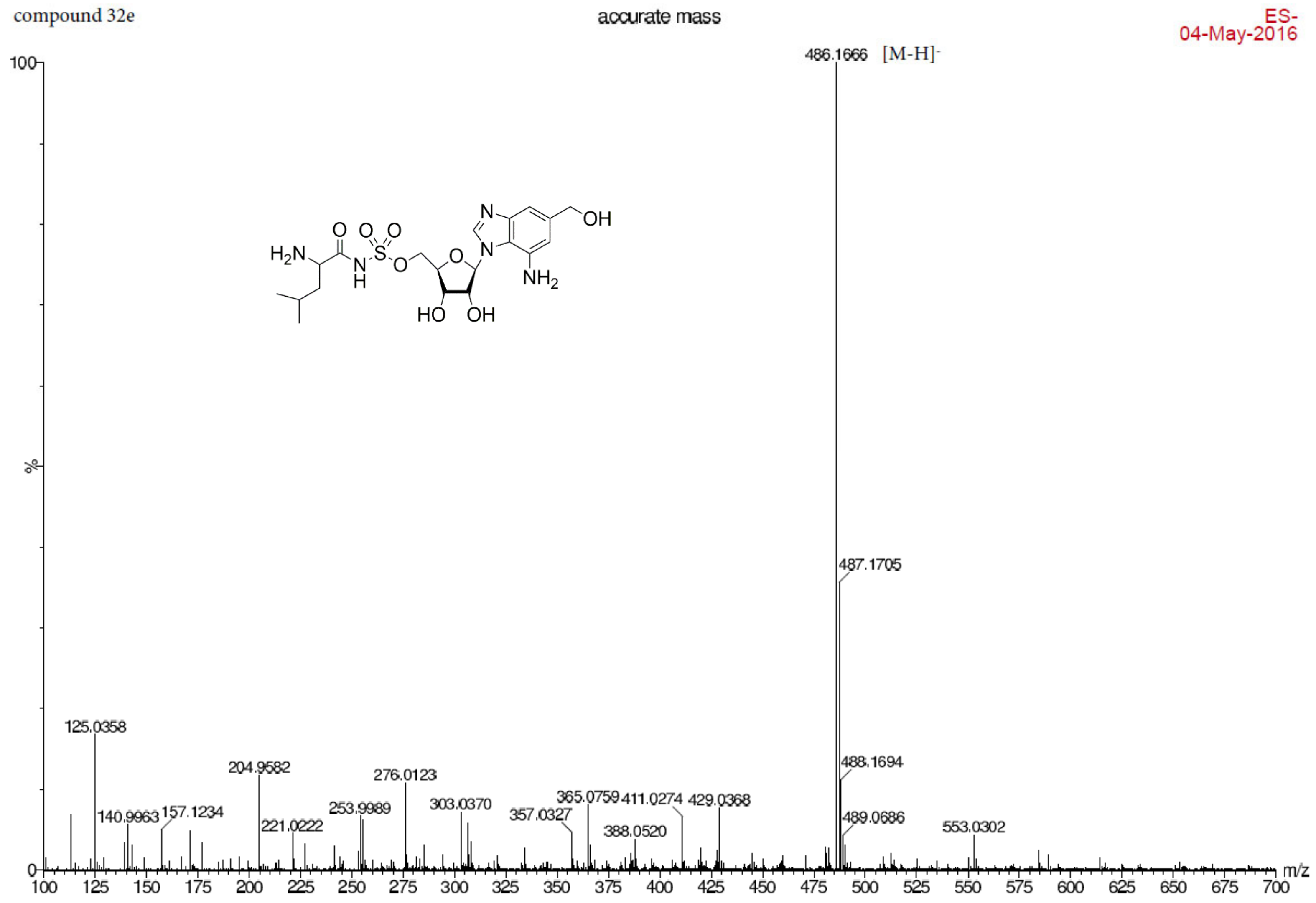
compound 32e
accurate mass
ES-
04-May-2016
486.1666 [M-H]-
487.1705
488.1694
489.0686
553.0302
125.0358
140.9963
157.1234
204.9582
221.0222
253.9989
276.0123
303.0370
357.0327
365.0759
388.0520
411.0274
429.0368
%
m/z
H2N
NH2
OH
HO
N
O
S

Compound 32f in Deuterium Oxide 13C spectrum - 75 MHz

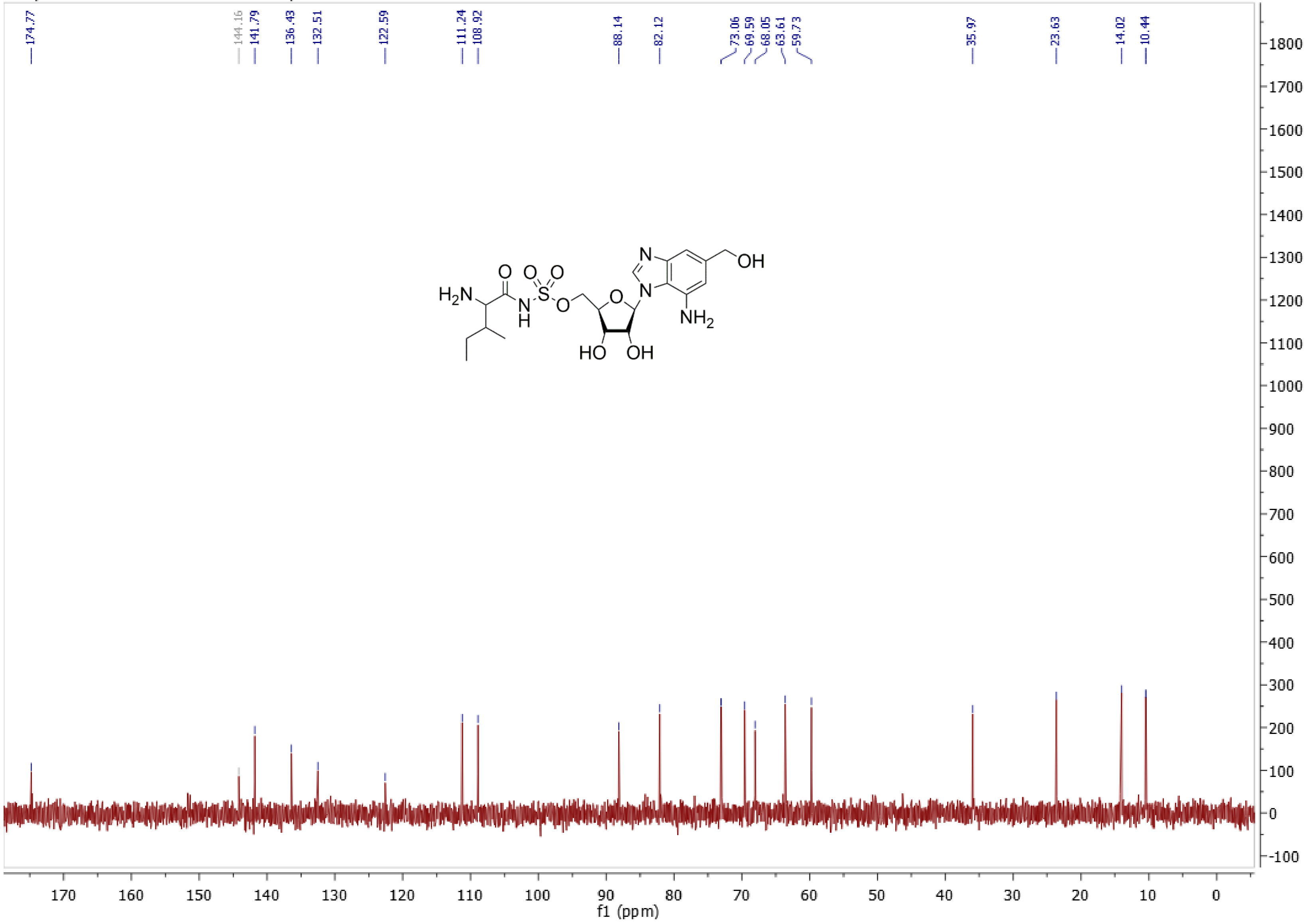

compound 32f

accurate mass

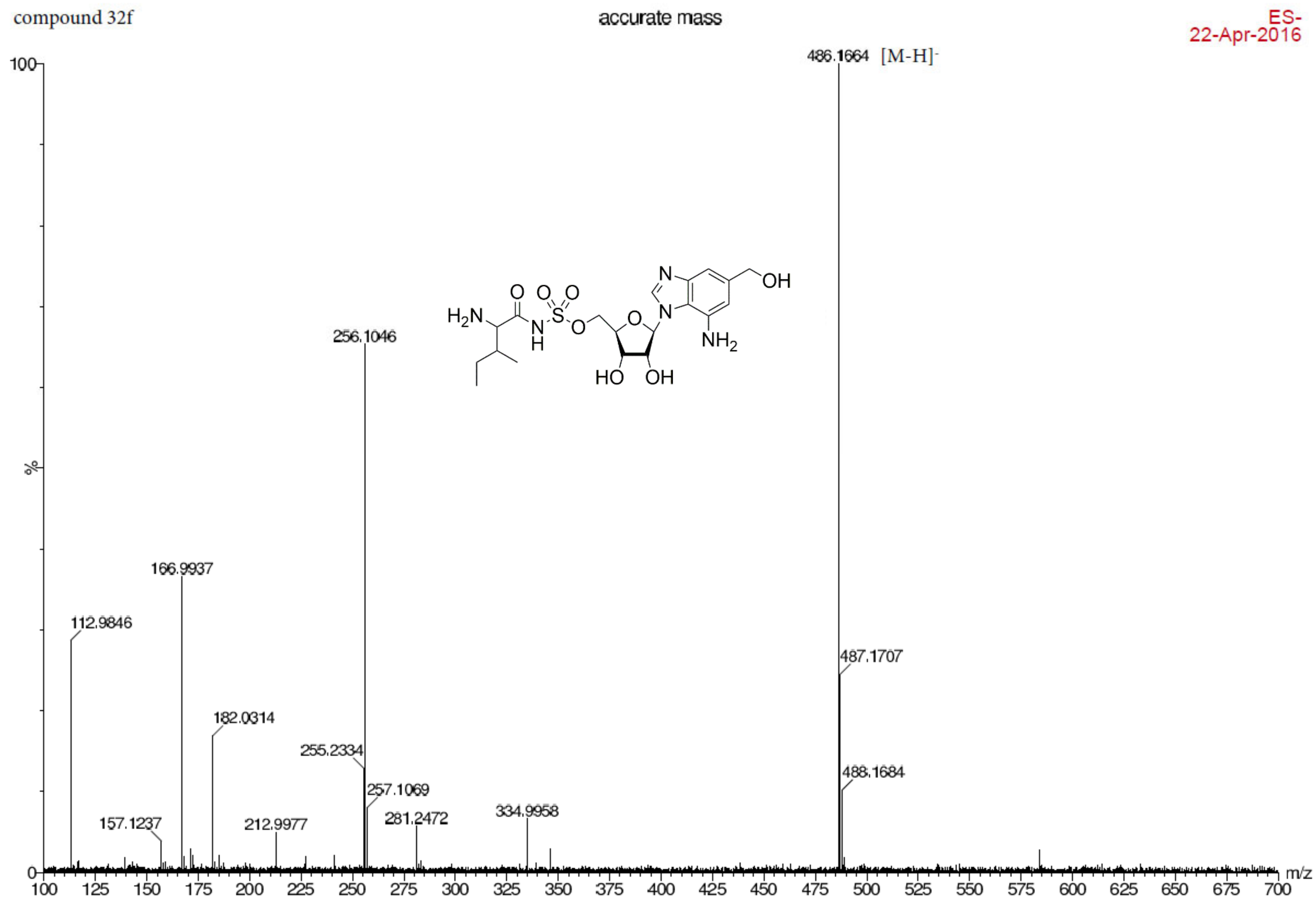

Supplement: Supplementary file 1 [file molecules-25-04751-s001.pdf]
